# Supplementary material for: Global Transcriptional and Epigenetic Reconfiguration during Chemical Reprogramming of Human Retinal Pigment Epithelial Cells into Photoreceptor-like Cells
Source: Cells. 2022 Oct 6;11(19):3146. doi: 10.3390/cells11193146 (PMC9564162; doi:10.3390/cells11193146)
Supplement: Supplementary file 1 [file cells-11-03146-s001.zip › Supplementary Figures S1-S6.pdf]

Supplementary Materials

Supplementary Figures S1–S6

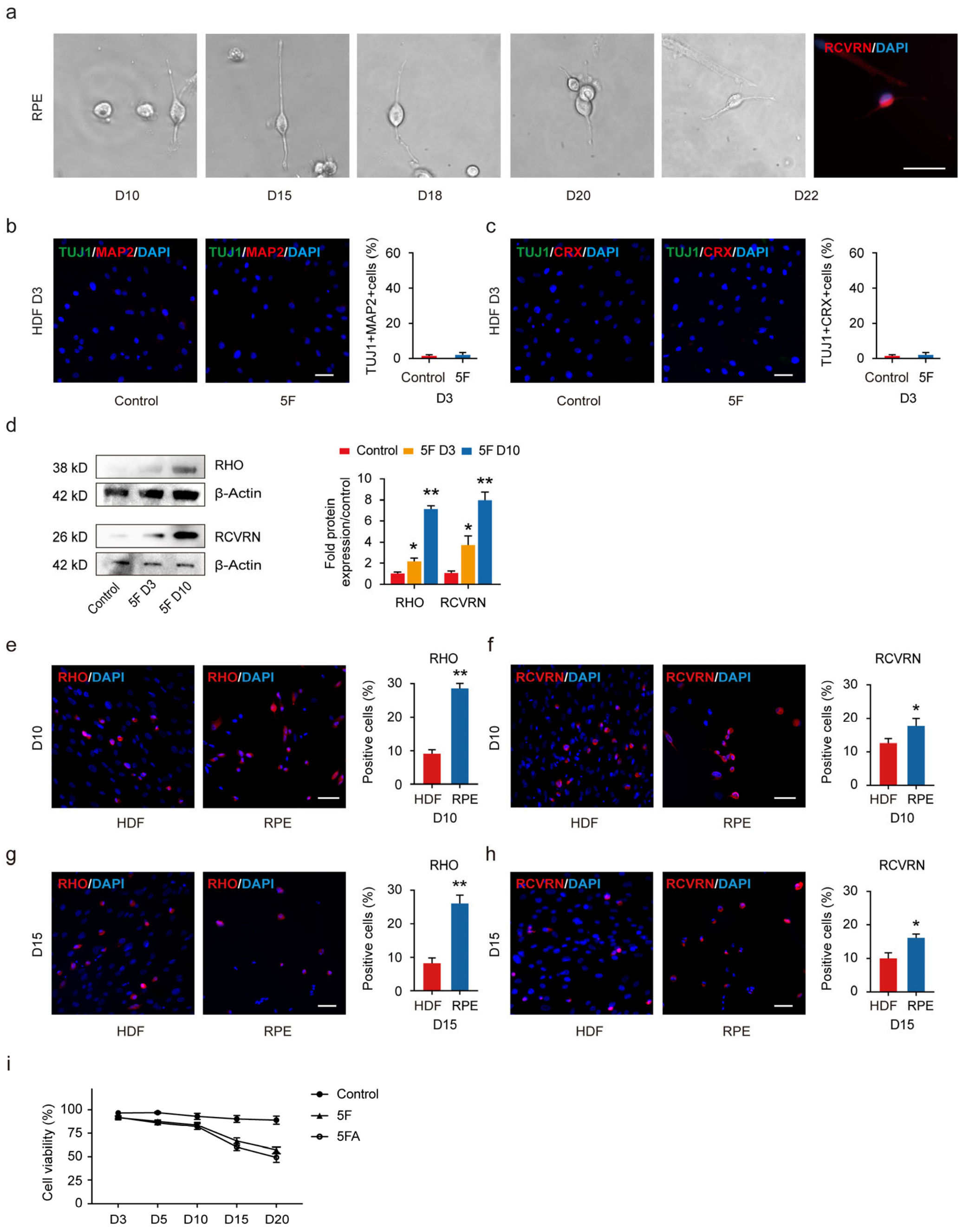

Supplementary Figure S1. 5F induced direct reprogramming of RPE cells into CiPCs. (**a**) Bright-field images of R-CiPCs at different extended induction time points. Immunostaining showed expression of photoreceptor marker RCVRN on Day (D) 22. Scale bar: 50 μm. (**b**,**c**) Representative images of HDF treated with 5F and stained for neuronal markers TUJ1, MAP2 (**b**) and TUJ1, CRX (**c**) at D3. Bar charts indicating the quantification of TUJ1 and MAP2 positive cells or TUJ1 and CRX positive cells among DAPI positive cells at D3, respectively. Nuclei were stained with DAPI. Scale bars: 50 μm. n=four for each group. Statistical comparison was performed by a two-tailed, unpaired t-test. Error bars indicate SD. (**d**) Western blot result for

the expression of RHO and RCVRN during CiPCs induction. n=three repetitions. Statistical significance was evaluated by one-way ANOVA with a Tukey's post hoc. (**e**,**f**) Representative immunostaining images of HDF and RPE cells treated with 5F stained for photoreceptor marker RHO (**e**) and RCVRN (**f**) at D10. Bar plot showing RHO and RCVRN positive cells among all DAPI positive cells. (**g**,**h**) Representative immunostaining images of HDF and RPE cells treated with 5F and stained for RHO (**g**) and RCVRN (**h**) at D15. Bar plot showing RHO and RCVRN positive cells among all DAPI positive cells. Nuclei were stained with DAPI. Scale bars: 50 μm. n=four repetitions. Statistical comparison was performed by a two-tailed, unpaired t-test. *$p < 0.05$, **$p < 0.01$. Error bars indicate SD. (**i**) Cell viability was estimated by trypan blue exclusion assay. n=three repetitions, data are shown as mean ± SD.

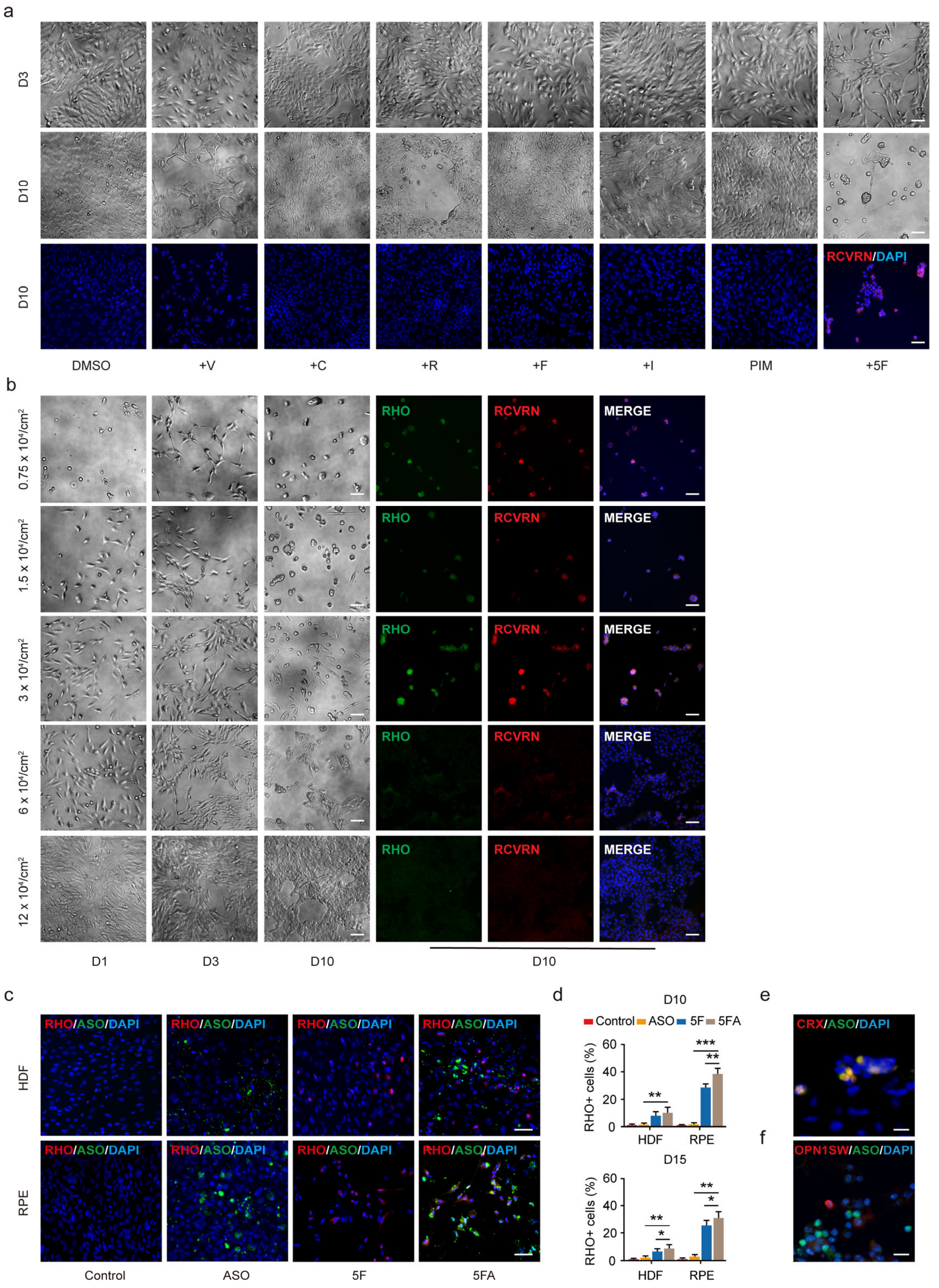

Supplementary Figure S2. 5F in combination with PTBP1 ASO induced direct conversion of RPE cells into CiPCs. (**a**) Bright-field images of RPE cells treated with dimethyl sulfoxide (DMSO), individual factor, PIM only or 5F at reprogramming D3 (up row) and D10 (middle row). Immunostaining showed expression of photoreceptor marker RCVRN related to each condition at D10 (below row). Scale bars: 50 μm. (**b**) Bright-field images of RPE cells started from different seeding densities during the reprogramming process at D1, D3, and D10 (left 3

columns), and immunostaining of photoreceptor markers RHO and RCVRN related to each starting density at D10 (right 3 columns). Starting densities are indicated on the left side of the figures. Scale bars: 50 μm. (**c**) Immunofluorescence staining of RHO on cells treated with ASO, 5F or 5FA at D10. Nuclei were stained with DAPI. n=four repetitions each group. Scale bars: 50 μm. (**d**) Quantifications of RHO positive staining cells among all DAPI positive cells in different conditions at D10 and D15. Statistical significance was evaluated by two-way ANOVA with a Bonferroni post hoc. $*p < 0.05$, $**p < 0.01$, $***p < 0.001$. Error bars indicate SD. (**e,f**) Immunofluorescence staining of CRX (**e**) and OPN1SW (**f**) on cells treated with 5FA. Nuclei were stained with DAPI. Scale bars: 25 μm.

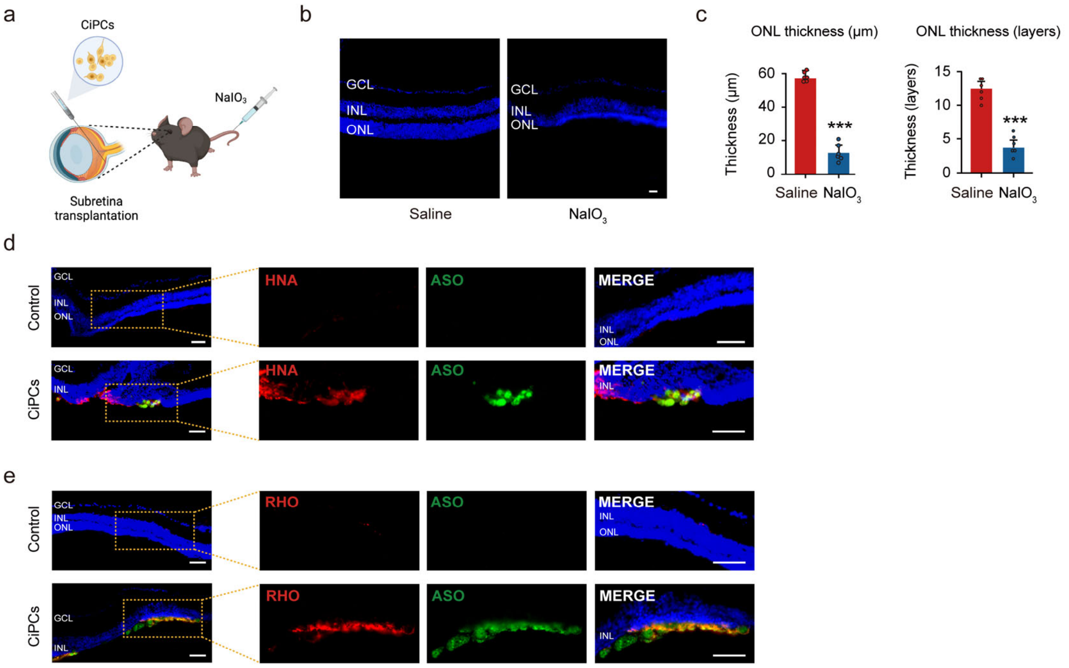

Supplementary Figure S3. Subretinal transplantation of CiPCs into a retinal degeneration mouse model. (**a**) Schematic of $NaIO_3$ induced retinal degeneration mouse model construction and subretinal transplantation of CiPCs. Intravenous injection of $NaIO_3$ causes RPE damage, leading to degeneration of photoreceptors. (**b**) Representative immunohistochemistry images of the retinal layers 4 weeks after saline (left) or $NaIO_3$ injection (right). Scale bar is 50 μm. (**c**) Bar plot showing the thickness of ONL in μm (left) or cell layers (right). n=six eyes for each group. Statistical significance was evaluated by a two-tailed, unpaired t-test. ***$p < 0.001$. Error bars indicate SD. (**d**) Representative immunostaining images of CiPCs survived into the subretinal space, stained for human nuclear antigen (HNA). (**e**) Representative immunostaining images of CiPCs stained for RHO 4 weeks after subretinal injection. The dashed line boxes are magnified on the right side. GCL, ganglion cell layer; INL, inner nuclear layer; ONL, outer nuclear layer. Nuclei were stained with DAPI. Scale bars: 50 μm.

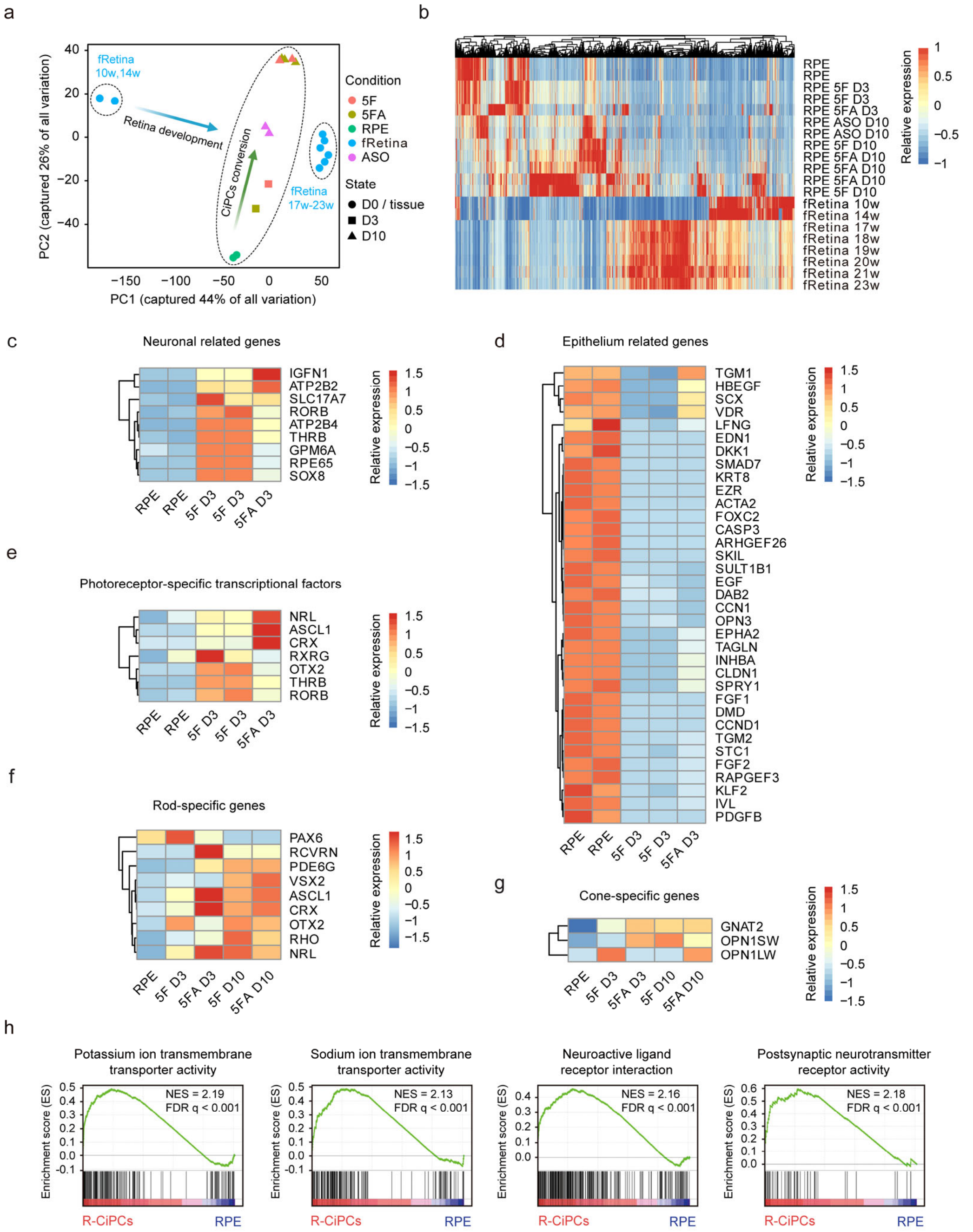

Supplementary Figure S4. 5F effectively reshaped the transcriptional profile. (**a**) PCA on the RNA-seq for the RPE cell samples with different treatments and fRetina tissue at different developing times. (**b**) Heatmap showing the gene expression profile conversion during R-CiPCs induction and fRetina development. W, week. (**c**) Heatmap of RNA-seq data for the expression of neuronal-related genes at the early stage (D3) of reprogramming. (**d**) Heatmap of RNA-seq data for the expression of epithelium-related genes at D3. (**e**) Heatmap of RNA-seq data for the expression of photoreceptor-specific transcriptional factors at D3. (**f**) Heatmap of RNA-seq data for the expression of rod-specific genes during R-CiPCs conversion (D3 and D10). (**g**) Heatmap of RNA-seq data for the expression of cone-specific genes during R-CiPCs conversion (D3 and D10). (**h**) GSEA plots evaluating the changes in the indicated gene signatures induced by the 5F treatment at D10. NES, normalized enrichment score. FDR, false discovery rate.

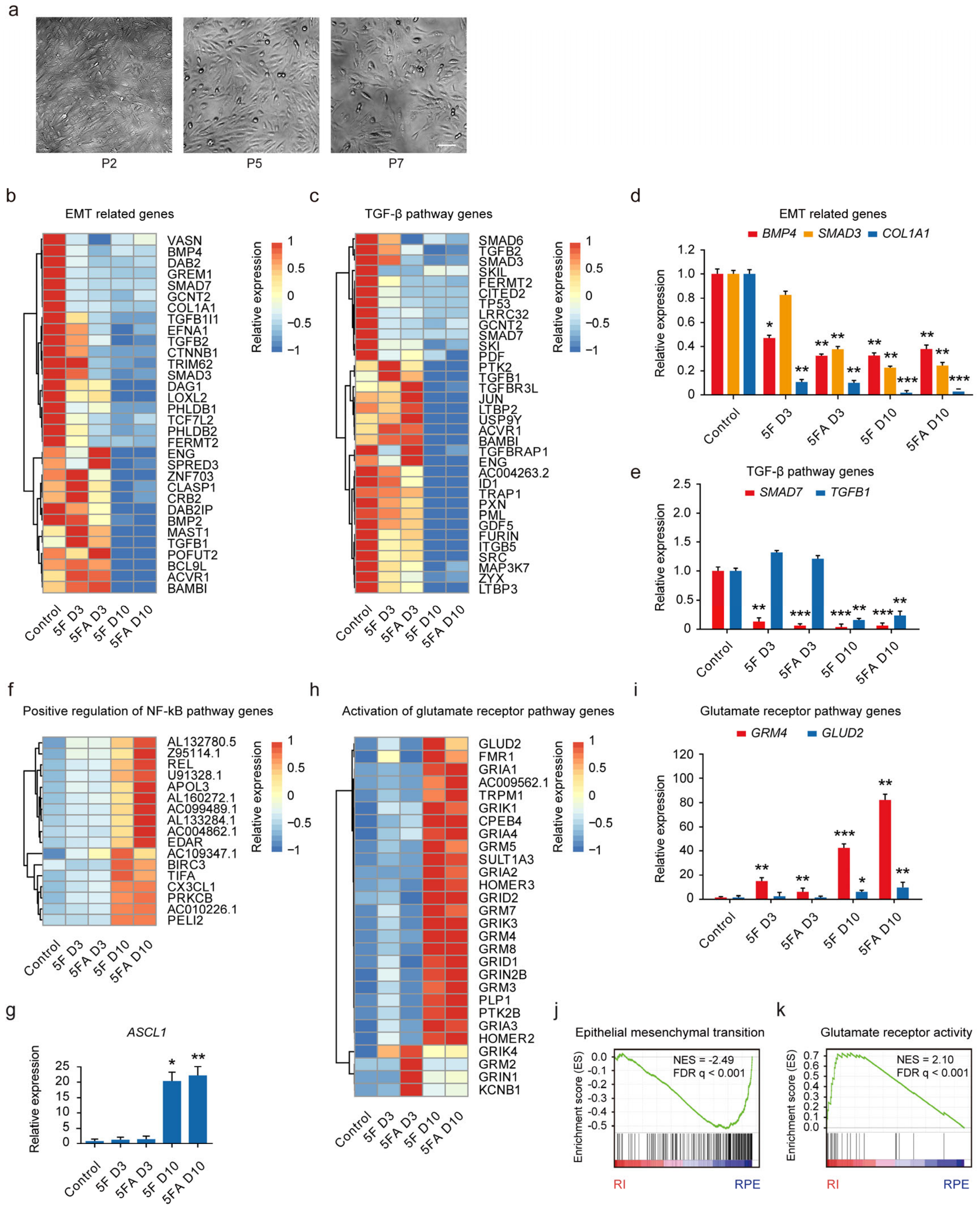

Supplementary Figure S5. Pathways influenced by the treatment of 5F. (**a**) Bright-field images of RPE cells at passages (P) 2, 5, and 7. Scale bar: 50 μm. (**b**) Heatmap showing the DEGs related to epithelial-mesenchymal transition (EMT) due to 5F treatment. (**c**) Heatmap showing the DEGs related to TGF-β signal pathway due to 5F treatment. (**d**) Bar plot showing the qPCR result of mRNA expression level of EMT related genes *BMP4*, *SMAD3* and *COL1A1*. (**e**) Bar plot showing the qPCR result of mRNA expression of TGF-β pathway genes *SMAD7* and *TGFB1*. (**f**) Heatmap showing the upregulated DEGs related to positive regulation of NF-kB pathway during the CiPCs induction. (**g**) Bar plot showing the mRNA expression level of *ASCL1* during CiPCs induction. (**h**) Heatmap showing the DEGs related to activation of

glutamate receptor pathway due to 5F treatment. (**i**) Bar plot showing the mRNA expression level of *GRM4* and *GLUD2* during CiPCs induction. Statistical significance was evaluated by one-way ANOVA with a Tukey's post hoc. *$p < 0.05$, **$p < 0.01$, ***$p < 0.001$. Error bars indicate SD. (**j**,**k**) GSEA plots evaluating the changes in the indicated gene signatures induced by the 5F treatment at D3. NES, normalized enrichment score. FDR, false discovery rate.

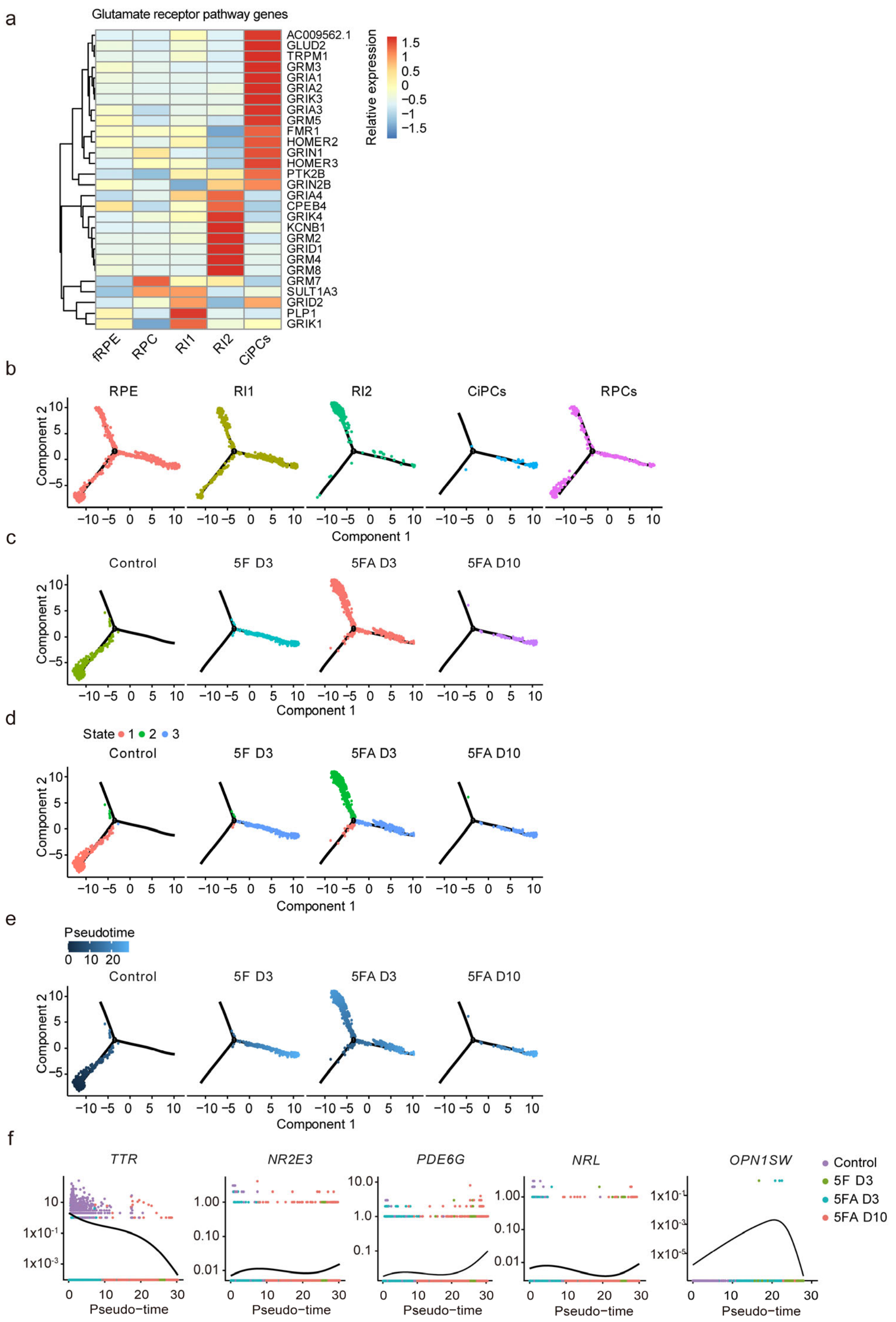

Supplementary Figure S6. Trajectory and pseudo-time analysis identify dynamic changes along with the commitment to CiPCs. (**a**) Heatmap showing the expression of glutamate receptor pathway genes in different cell types in the D10 sample. (**b**-**e**) Single-cell pseudo-time trajectory analysis was performed by Monocle2. Cells on the tree are colored by cell type and treatment condition. (**b**) Monocle pseudo-time trajectory of the CiPCs conversion process. Cells are labeled by the cell subtype. (**c**) Monocle pseudo-time trajectory of the CiPCs conversion process. Cells are labeled by the sample treatment condition. (**d**) Monocle pseudo-time trajectory of the CiPCs conversion process. Cells are labeled by the state inferred by Monocle2. (**e**) Monocle pseudo-time trajectory of the CiPCs conversion process. Cells are labeled by the

pseudo-time value. (**f**) Kinetic curves for RPE lineage gene *TTR*, rod photoreceptor lineage genes *NR2E3*, *PDE6G*, *NRL* and cone photoreceptor lineage gene *OPN1SW*.

Supplementary Video S1. Live-cell time-lapse imaging shows that RPE cells gained neuron-like morphological changes on the first day of reprogramming.
