# Supplementary material for: Global Transcriptional and Epigenetic Reconfiguration during Chemical Reprogramming of Human Retinal Pigment Epithelial Cells into Photoreceptor-like Cells
Source: Cells. 2022 Oct 6;11(19):3146. doi: 10.3390/cells11193146 (PMC9564162; doi:10.3390/cells11193146)
Supplement: Supplementary file 1 [file cells-11-03146-s001.zip › Supplementary Table S1.pdf]

Supplementary Materials

Supplementary Table S1:

Differently expressed genes (DEGs) in D3 intermediates compared with RPE control

Gene	Base mean	Log2 fold change	lfcSE	Stat	p value	p adjust
AJAP1	169.2997	2.482038271	0.249197	9.960135	2.28E-23	2.42E-22
SPSB1	7948.463	-2.650574188	0.057836	-45.8292	0	0
NPPB	7331.942	9.750439274	0.170737	57.10798	0	0
TMEM51-A	131.2015	-4.562880965	0.399644	-11.4174	3.42E-30	4.42E-29
TMEM51	2192.678	-3.140217295	0.098429	-31.9033	2.40E-223	2.24E-221
FHAD1	79.98011	-2.333544903	0.367788	-6.34481	2.23E-10	1.40E-09
HSPB7	858.0682	3.388077161	0.134758	25.142	1.73E-139	9.82E-138
EPHA2	15385.98	2.72578528	0.043927	62.05326	0	0
LINC02783	203.7787	2.564366956	0.258745	9.910802	3.74E-23	3.96E-22
PADI1	116.3206	2.079145871	0.293616	7.081175	1.43E-12	1.00E-11
ALPL	73.71242	-2.586800922	0.386124	-6.6994	2.09E-11	1.39E-10
HTR1D	76.06735	3.35949326	0.367502	9.141431	6.16E-20	5.86E-19
MAN1C1	1025.403	-2.113082058	0.122817	-17.2051	2.43E-66	6.39E-65
THEMIS2	370.1297	-3.163044989	0.223115	-14.1767	1.28E-45	2.36E-44
TINAGL1	2004.555	2.001306174	0.093812	21.33309	5.60E-101	2.23E-99
MYCL	118.548	2.300463564	0.315507	7.291321	3.07E-13	2.22E-12
EDN2	3048.859	4.332419029	0.078553	55.15305	0	0
CLDN19	61.39263	-2.325115956	0.395373	-5.88081	4.08E-09	2.37E-08
DMBX1	361.1474	-2.500873824	0.42138	-5.93497	2.94E-09	1.73E-08
ELAVL4	21.26323	-2.130572901	0.451355	-4.72039	2.35E-06	1.10E-05
SLC1A7	56.87534	-2.135942751	0.399694	-5.34394	9.09E-08	4.80E-07
LRP8	1767.07	-2.215543563	0.101753	-21.7738	4.11E-105	1.71E-103
AL603840.	26.12094	-2.109328365	0.44797	-4.70864	2.49E-06	1.16E-05
PLPP3	2441.537	-3.261650492	0.096708	-33.7269	2.33E-249	2.42E-247
PDE4B	813.3371	-4.280686779	0.379838	-11.2698	1.85E-29	2.34E-28
RPE65	195.8182	-4.175889147	0.334926	-12.4681	1.11E-35	1.65E-34
NEXN	6208.69	3.957775253	0.061381	64.47917	0	0
IFI44L	218.0424	-2.016999434	0.248606	-8.11322	4.93E-16	4.08E-15
AC092807.	113.2824	2.599270321	0.308424	8.427596	3.53E-17	3.04E-16
CCN1	29703.91	3.062405699	0.032852	93.21963	0	0
COL24A1	286.8004	-2.398995293	0.224646	-10.679	1.28E-26	1.50E-25
PKN2-AS1	35.8078	-2.52654433	0.439917	-5.74323	9.29E-09	5.27E-08
TGFBR3	178.5146	-2.102688265	0.266989	-7.87556	3.39E-15	2.69E-14
BCAR3	9613.835	2.371478218	0.045679	51.91581	0	0
F3	14446.14	2.301554957	0.036344	63.32719	0	0
PLPPR4	25.07942	-2.302344268	0.45075	-5.10781	3.26E-07	1.64E-06
KCNA2	46.98957	-3.174976906	0.43625	-7.27789	3.39E-13	2.45E-12
CHI3L2	172.3428	-2.25478575	0.2999	-7.51845	5.54E-14	4.16E-13
KCND3	143.4549	2.161382651	0.276369	7.820643	5.26E-15	4.13E-14

HSD3B1	265.2871	2.625353035	0.209457	12.53411	4.86E-36	7.25E-35
AC239859.	42.61572	-2.713586102	0.433891	-6.25408	4.00E-10	2.47E-09
NBPF17P	23.13951	-2.064552873	0.450786	-4.57989	4.65E-06	2.11E-05
CGN	3414.627	-2.662084108	0.074499	-35.733	1.22E-279	1.48E-277
RORC	454.9126	-5.984882366	0.346477	-17.2735	7.45E-67	1.98E-65
CRCT1	33.25415	-2.429686541	0.44207	-5.49616	3.88E-08	2.11E-07
IVL	375.5448	4.415835225	0.20311	21.74114	8.38E-105	3.47E-103
IL6R	711.1142	-3.556134497	0.178119	-19.9649	1.11E-88	3.85E-87
AL353807.	122.5345	2.097720565	0.281841	7.442926	9.85E-14	7.30E-13
KRT8P45	61.60915	2.051311055	0.361894	5.668271	1.44E-08	8.08E-08
FCRL5	32.24823	-2.669992012	0.446354	-5.98179	2.21E-09	1.31E-08
SPTA1	43.43641	-2.535296826	0.43001	-5.89591	3.73E-09	2.17E-08
ATP1A2	26.93954	-2.015478254	0.446602	-4.51292	6.39E-06	2.86E-05
ATP1A4	26.08515	-2.038808816	0.448066	-4.55025	5.36E-06	2.42E-05
LY9	30.96573	-2.621284331	0.447058	-5.8634	4.53E-09	2.63E-08
FAM78B	786.9	-2.888120979	0.162605	-17.7615	1.40E-70	3.92E-69
ADCY10	25.23147	-2.337617001	0.450443	-5.18959	2.11E-07	1.08E-06
F5	45.20603	-2.384827934	0.42096	-5.66522	1.47E-08	8.22E-08
PRRX1	637.8538	-2.463518186	0.162256	-15.1829	4.59E-52	9.53E-51
TNFSF18	265.5451	5.106274287	0.259669	19.66457	4.34E-86	1.46E-84
TNR	48.41414	-2.887173826	0.429423	-6.72338	1.78E-11	1.18E-10
BRINP2	1684.77	-3.249272745	0.113944	-28.5164	7.34E-179	5.33E-177
CACNA1E	95.90236	-3.858827946	0.401143	-9.61959	6.61E-22	6.72E-21
LINC01344	30.85413	-2.06391835	0.442621	-4.66295	3.12E-06	1.44E-05
COLGALT2	313.2941	-2.334869191	0.215927	-10.8133	2.98E-27	3.55E-26
NIBAN1	7086.731	-3.595651811	0.06262	-57.4202	0	0
IVNS1ABP	11422.98	2.426427952	0.040179	60.39078	0	0
KIF21B	1002.031	-2.596074406	0.13097	-19.8218	1.93E-87	6.57E-86
CACNA1S	22.08697	-2.154646694	0.451338	-4.77391	1.81E-06	8.55E-06
IGFN1	329.8731	-3.642683698	0.251537	-14.4817	1.58E-47	3.03E-46
CHI3L1	2035.474	-2.385154744	0.091335	-26.1144	2.50E-150	1.53E-148
OPTC	92.89286	-2.241072427	0.356751	-6.28189	3.34E-10	2.07E-09
ATP2B4	59840.39	-2.17994295	0.028367	-76.8492	0	0
CNTN2	39.27359	-2.904441393	0.442389	-6.56536	5.19E-11	3.38E-10
CR1	21.07023	-2.117654341	0.451366	-4.69166	2.71E-06	1.26E-05
MIR29B2C	60.6088	-2.152877407	0.396632	-5.4279	5.70E-08	3.06E-07
LAMB3	314.6735	-2.411576016	0.216729	-11.1271	9.26E-29	1.15E-27
G0S2	65.10936	-3.124369437	0.410716	-7.60714	2.80E-14	2.13E-13
SERTAD4-	219.3244	3.86994162	0.25524	15.16198	6.31E-52	1.31E-50
SERTAD4	109.1074	2.4068377	0.307057	7.838404	4.56E-15	3.60E-14
USH2A	66.55098	-2.339219204	0.384765	-6.0796	1.20E-09	7.25E-09
LINC02869	54.87115	2.930041868	0.395424	7.409868	1.26E-13	9.32E-13
HHIPL2	239.9609	-3.222003264	0.273996	-11.7593	6.32E-32	8.54E-31
ITPKB	14121.75	-2.67639836	0.042018	-63.6963	0	0
RHOU	4707.579	-3.00297959	0.069394	-43.2744	0	0
AL136171.	25.06651	-2.315312695	0.45065	-5.13772	2.78E-07	1.41E-06
SIPA1L2	3270.935	-4.362516784	0.102393	-42.6057	0	0

ACTN2	250.7039	-4.14657945	0.305188	-13.587	4.78E-42	8.16E-41
RYR2	72.37843	-3.752684335	0.421813	-8.89655	5.76E-19	5.28E-18
KMO	196.9789	2.746224693	0.265896	10.32819	5.25E-25	5.87E-24
OPN3	472.4791	2.34368971	0.159366	14.7063	5.87E-49	1.15E-47
CHML	1163.391	2.104769375	0.102334	20.56761	5.35E-94	1.98E-92
OR2M3	21.64491	-2.159286026	0.451307	-4.78452	1.71E-06	8.13E-06
ALKAL2	212.1765	3.891194604	0.246877	15.76164	5.71E-56	1.27E-54
MYT1L	47.83757	-3.197056946	0.435799	-7.33608	2.20E-13	1.60E-12
AC013460.	23.66756	-2.274620734	0.450819	-5.04553	4.52E-07	2.25E-06
AC099344.	23.34573	-2.250017676	0.450978	-4.9892	6.06E-07	2.99E-06
TRIB2	2631.068	-3.007249992	0.098354	-30.5759	2.56E-205	2.15E-203
LRATD1	3981.099	-4.176912938	0.089946	-46.4379	0	0
MYCN	343.034	2.506972895	0.191759	13.07354	4.66E-39	7.46E-38
VSNL1	115.2992	-2.629677189	0.333816	-7.87763	3.34E-15	2.65E-14
APOB	25.44872	-2.360137764	0.450218	-5.24222	1.59E-07	8.19E-07
TOGARAM	136.2862	-3.250471507	0.338664	-9.59794	8.16E-22	8.27E-21
PCARE	40.37874	-2.972754444	0.440706	-6.74544	1.53E-11	1.02E-10
ALK	38.45794	-2.598021197	0.438735	-5.92162	3.19E-09	1.87E-08
CAPN13	19.47777	-2.032226382	0.451282	-4.50322	6.69E-06	2.98E-05
CYP1B1	12544.1	-2.286849162	0.050286	-45.4771	0	0
CYP1B1-A	378.3	-2.958560052	0.213128	-13.8816	8.19E-44	1.46E-42
AC012354.	251.4074	-2.447070583	0.242671	-10.0839	6.51E-24	7.03E-23
BCYRN1	1974.943	2.364753595	0.431177	5.484413	4.15E-08	2.25E-07
KCNK12	875.739	-4.431421454	0.189674	-23.3634	1.01E-120	4.90E-119
LHCGR	49.03733	-2.910082814	0.428683	-6.78842	1.13E-11	7.62E-11
NRXN1	47.46208	-3.051802337	0.432605	-7.05447	1.73E-12	1.21E-11
EML6	221.7648	-3.411215983	0.285193	-11.9611	5.68E-33	7.86E-32
MIR4432H	24.37161	-2.004556348	0.449514	-4.45939	8.22E-06	3.62E-05
REL	45.89032	-2.196965837	0.423563	-5.18687	2.14E-07	1.09E-06
TGFA	149.4614	2.48235925	0.269784	9.201284	3.54E-20	3.39E-19
ACTG2	312.0732	2.237846549	0.192081	11.65053	2.28E-31	3.03E-30
AC007099.	303.4384	-5.26980046	0.347195	-15.1782	4.93E-52	1.02E-50
CTNNA2	53.67763	-2.020432523	0.403318	-5.00953	5.46E-07	2.70E-06
DNAH6	57.95498	-2.334188596	0.399583	-5.84156	5.17E-09	2.99E-08
TRABD2A	640.931	-2.542137564	0.16752	-15.1752	5.17E-52	1.07E-50
SH2D6	124.0153	-3.483740687	0.356795	-9.76399	1.61E-22	1.67E-21
REEP1	387.628	-2.642308918	0.209871	-12.5902	2.39E-36	3.59E-35
FOXI3	66.43399	-2.7409489	0.395173	-6.93608	4.03E-12	2.77E-11
AC027612.	35.34852	-2.795709029	0.444163	-6.29433	3.09E-10	1.92E-09
GGT8P	63.8948	-3.097962854	0.411606	-7.52652	5.21E-14	3.92E-13
KCNIP3	845.6401	-3.318570748	0.161906	-20.4969	2.30E-93	8.39E-92
LONRF2	579.1499	-3.322172524	0.188009	-17.6703	7.11E-70	1.97E-68
LINC01102	22.58615	-2.205085717	0.451176	-4.88742	1.02E-06	4.94E-06
PANTR1	19.31712	-2.004790076	0.451173	-4.44351	8.85E-06	3.89E-05
FHL2	8826.594	2.331102104	0.044503	52.38117	0	0
ST6GAL2	791.026	2.468893602	0.130527	18.91484	8.61E-80	2.71E-78
SULT1C2	393.83	-3.314476643	0.229775	-14.4249	3.61E-47	6.84E-46

BCL2L11	1241.749	-2.068826329	0.111596	-18.5386	1.01E-76	3.00E-75
MERTK	299.3095	-2.384208388	0.238619	-9.99169	1.66E-23	1.77E-22
IL1A	114.7363	-2.268433033	0.321788	-7.04946	1.80E-12	1.25E-11
IL1B	1173.642	-4.441337883	0.167168	-26.5681	1.59E-155	9.97E-154
AC016745.	218.172	-2.057334291	0.248002	-8.29563	1.08E-16	9.15E-16
CFAP221	121.0393	-2.451894072	0.32135	-7.62998	2.35E-14	1.79E-13
LIMS2	2872.521	2.831567714	0.072367	39.12781	0	0
KYNU	77.45531	-2.989723558	0.387963	-7.7062	1.30E-14	1.00E-13
TEX41	48.00475	-2.58166341	0.425744	-6.06389	1.33E-09	7.97E-09
KIF5C	235.1302	-2.29295183	0.242431	-9.45816	3.13E-21	3.12E-20
DAPL1	330.7969	-4.048599039	0.273958	-14.7782	2.03E-49	4.01E-48
KCNH7	121.6613	-2.773752354	0.331614	-8.3644	6.04E-17	5.17E-16
AC019197.	22.30422	-2.184182494	0.451258	-4.84021	1.30E-06	6.23E-06
SLC38A11	119.7284	-2.687382138	0.339093	-7.9252	2.28E-15	1.82E-14
ABCB11	20.7085	-2.072060123	0.451342	-4.59089	4.41E-06	2.01E-05
LRP2	68.17009	-2.676904877	0.393735	-6.79875	1.06E-11	7.11E-11
MYO3B	80.81707	-2.660390084	0.373197	-7.12864	1.01E-12	7.16E-12
AC007405.	86.62353	-2.079093885	0.347223	-5.98777	2.13E-09	1.26E-08
TTN	269.4996	-2.619717381	0.242436	-10.8058	3.23E-27	3.85E-26
CCDC141	58.31778	-2.083827425	0.389813	-5.34571	9.01E-08	4.75E-07
KRT18P29	648.5783	3.42380654	0.150761	22.7102	3.55E-114	1.64E-112
FRZB	264.4545	-3.745978986	0.280166	-13.3706	8.99E-41	1.49E-39
KRT8P10	129.035	2.327240101	0.281694	8.261601	1.44E-16	1.21E-15
GULP1	1580.507	-2.06001296	0.103326	-19.9371	1.94E-88	6.70E-87
COL3A1	475.2817	2.111183244	0.157205	13.4295	4.06E-41	6.80E-40
STAT4	89.13728	-2.035276151	0.34363	-5.92287	3.16E-09	1.85E-08
TMEFF2	1111.235	-2.005891691	0.116637	-17.1977	2.76E-66	7.25E-65
DNAH7	79.4145	-2.165721058	0.360303	-6.01084	1.85E-09	1.10E-08
PLCL1	122.411	-3.316644742	0.353519	-9.38181	6.49E-21	6.38E-20
LINC01877	26.57916	-2.394380147	0.449975	-5.32114	1.03E-07	5.41E-07
GPR1	318.6253	2.743755069	0.19466	14.09512	4.07E-45	7.44E-44
UNC80	210.5364	-2.248434832	0.259387	-8.66827	4.39E-18	3.91E-17
ERBB4	127.0263	-2.127604767	0.306149	-6.94958	3.66E-12	2.52E-11
MARCHF4	935.1746	2.117996314	0.112731	18.78809	9.45E-79	2.93E-77
IGFBP5	15073.5	-3.862560222	0.048778	-79.1864	0	0
PLCD4	219.5214	-3.749144283	0.300961	-12.4573	1.28E-35	1.88E-34
CYP27A1	3533.342	-2.64670738	0.074163	-35.6877	6.13E-279	7.43E-277
PTPRN	2494.412	-4.693757632	0.124731	-37.631	0	0
KCNE4	235.8322	-3.327769964	0.275749	-12.0681	1.56E-33	2.19E-32
SCG2	5562.176	-6.09176624	0.12263	-49.6758	0	0
AC016717.	64.60321	2.650535224	0.358722	7.388834	1.48E-13	1.09E-12
COL4A4	687.568	2.614536794	0.140183	18.65094	1.24E-77	3.77E-76
SPHKAP	36.31401	-2.812068146	0.44402	-6.33321	2.40E-10	1.51E-09
NPPC	86.50848	3.322577295	0.340272	9.764467	1.60E-22	1.66E-21
ECEL1P2	46.27766	2.067713232	0.39225	5.271417	1.35E-07	7.04E-07
MLPH	4899.96	-2.190184817	0.061156	-35.813	6.94E-281	8.54E-279
RAB17	1516.121	3.719974229	0.098964	37.58916	0	0

KLHL30	141.026	2.853964905	0.280113	10.18863	2.23E-24	2.45E-23
CROCC2	31.37597	-2.635756457	0.446854	-5.89847	3.67E-09	2.14E-08
BOK	6094.766	2.047267152	0.049298	41.52802	0	0
OXTR	4190.514	6.739674012	0.09722	69.32369	0	0
ATP2B2	58.06298	-3.461596342	0.429299	-8.06336	7.42E-16	6.08E-15
SLC6A1	27.19427	-2.438145031	0.449484	-5.42432	5.82E-08	3.12E-07
KRT18P17	437.5704	3.12384054	0.17295	18.06207	6.34E-73	1.82E-71
GRIP2	86.26285	-2.254697364	0.358466	-6.28985	3.18E-10	1.98E-09
GALNT15	545.4344	-2.694335281	0.17864	-15.0825	2.11E-51	4.33E-50
ZNF385D	251.3505	-2.123056788	0.235492	-9.0154	1.96E-19	1.82E-18
THRB	375.8378	-2.001600139	0.191444	-10.4553	1.39E-25	1.57E-24
ITGA9	24.3735	-2.162219759	0.449847	-4.80657	1.54E-06	7.32E-06
SCN10A	22.48965	-2.19892242	0.451199	-4.87351	1.10E-06	5.29E-06
SCN11A	45.8308	-2.049783539	0.414364	-4.94681	7.54E-07	3.68E-06
CSRNP1	1162.402	2.041297193	0.10323	19.77431	4.96E-87	1.68E-85
MOBP	19.86754	-2.045356359	0.451302	-4.53212	5.84E-06	2.62E-05
ENTPD3	523.9462	-3.872857095	0.2144	-18.0637	6.16E-73	1.77E-71
SLC6A20	19.91983	-2.050620051	0.451316	-4.54365	5.53E-06	2.49E-05
CCR1	48.20446	-2.890037261	0.429196	-6.73361	1.65E-11	1.10E-10
CELSR3	1053.527	-2.304608915	0.133182	-17.3042	4.37E-67	1.17E-65
KLHDC8B	14807.44	-2.714702078	0.041226	-65.8487	0	0
UBA7	125.9479	2.161869029	0.292262	7.397018	1.39E-13	1.02E-12
CAMKV	230.0872	-4.887615286	0.355716	-13.7402	5.83E-43	1.02E-41
TNNC1	815.5644	3.078437467	0.143539	21.44668	4.90E-102	1.97E-100
ITIH3	70.6646	2.155977335	0.343894	6.269303	3.63E-10	2.25E-09
ABHD6	1627.889	-2.21938561	0.1012	-21.9306	1.33E-106	5.68E-105
FAM107A	186.8319	-4.790663606	0.374789	-12.7823	2.06E-37	3.17E-36
SYNPR	45.77765	-2.195949783	0.423463	-5.18569	2.15E-07	1.10E-06
SAMMSON	29.49402	-2.557680828	0.447969	-5.7095	1.13E-08	6.39E-08
GPR27	57.70019	2.033899213	0.365636	5.56263	2.66E-08	1.46E-07
CNTN3	949.4392	-2.173881839	0.129096	-16.8393	1.26E-63	3.16E-62
ROBO2	72.5833	-3.504611735	0.412905	-8.4877	2.11E-17	1.83E-16
VGLL3	1024.946	2.908839174	0.114996	25.29513	3.61E-141	2.09E-139
LINC00635	19.84759	-2.055533922	0.451333	-4.55436	5.25E-06	2.37E-05
NECTIN3-A	162.9466	2.846335417	0.2605	10.92642	8.62E-28	1.04E-26
TAGLN3	99.94862	-2.223088791	0.338201	-6.57328	4.92E-11	3.21E-10
CD200	523.3732	3.067646595	0.153548	19.97839	8.49E-89	2.95E-87
SIDT1	27.66058	-2.470211727	0.449086	-5.50053	3.79E-08	2.06E-07
LSAMP	2400.112	-5.718421408	0.163388	-34.9989	2.33E-268	2.63E-266
AC092691.	41.1014	-2.06557382	0.422094	-4.89364	9.90E-07	4.79E-06
CD80	20.31012	-2.083088074	0.451366	-4.61508	3.93E-06	1.80E-05
PLA1A	41.50059	-3.009156809	0.439944	-6.83986	7.93E-12	5.37E-11
HGD	37.68545	-2.601476883	0.438049	-5.93878	2.87E-09	1.69E-08
CASR	20.90906	-2.115585808	0.451367	-4.68707	2.77E-06	1.29E-05
SEMA5B	57.20608	-2.524681506	0.406749	-6.20698	5.40E-10	3.31E-09
COL6A5	20.53924	-2.098107693	0.45137	-4.64831	3.35E-06	1.54E-05
ACP3	102.5706	-2.704352025	0.353341	-7.65366	1.95E-14	1.50E-13

TMEM108	942.8635	-2.260891855	0.130026	-17.388	1.02E-67	2.74E-66
TF	104.4932	-2.166545034	0.33482	-6.47077	9.75E-11	6.25E-10
AMOTL2	17955.43	2.427786945	0.033824	71.77644	0	0
AC092969.	26.50745	-2.095197438	0.448485	-4.67172	2.99E-06	1.38E-05
ESYT3	1957.061	-2.836208363	0.100165	-28.3154	2.24E-176	1.60E-174
SLC9A9	517.3375	-4.356965393	0.235722	-18.4835	2.81E-76	8.32E-75
TM4SF18	971.9284	-2.514446968	0.132458	-18.9829	2.36E-80	7.48E-79
IGSF10	64.72991	-2.546164233	0.391602	-6.50192	7.93E-11	5.10E-10
MBNL1-AS	246.7132	2.599799484	0.216625	12.00137	3.49E-33	4.87E-32
P2RY1	626.12	-4.554811012	0.226141	-20.1415	3.20E-90	1.13E-88
ARHGEF26	2226.548	2.525256587	0.076391	33.05681	1.24E-239	1.22E-237
PTX3	1658.727	-2.195507649	0.10058	-21.8286	1.24E-105	5.22E-104
SLC66A1L	235.026	-2.044547883	0.242706	-8.42397	3.64E-17	3.13E-16
RARRES1	1822.322	2.852366186	0.085244	33.46122	1.77E-245	1.80E-243
ARL14	69.27994	2.991865171	0.357958	8.358145	6.37E-17	5.45E-16
LINC01192	19.04758	-2.002270065	0.451181	-4.43785	9.09E-06	3.99E-05
SLITRK3	41.8429	-2.740110714	0.434714	-6.30325	2.91E-10	1.82E-09
LINC01322	27.18241	-2.158347668	0.447264	-4.82567	1.40E-06	6.68E-06
SKIL	2563.451	2.379660393	0.07132	33.36598	4.27E-244	4.35E-242
CLDN11	5087.813	3.806539023	0.058581	64.97954	0	0
PEX5L	80.08173	-2.875083484	0.38186	-7.52916	5.11E-14	3.85E-13
LINC01206	44.96314	-2.837257054	0.432209	-6.56455	5.22E-11	3.40E-10
MCF2L2	95.90672	-2.723242948	0.354677	-7.67809	1.61E-14	1.24E-13
LIPH	321.6339	2.787966045	0.191592	14.5516	5.71E-48	1.10E-46
ETV5	1882.403	3.615170311	0.088259	40.96079	0	0
AC068631.	22.35703	-2.182101546	0.451262	-4.83555	1.33E-06	6.37E-06
MASP1	93.07389	-2.500711914	0.356381	-7.01696	2.27E-12	1.58E-11
SST	226.8381	-3.45303951	0.288625	-11.9638	5.50E-33	7.61E-32
P3H2	9216.343	2.738826035	0.045725	59.89739	0	0
CLDN1	3221.976	2.332665917	0.065996	35.34549	1.18E-273	1.37E-271
ATP13A4	37.88413	-2.136692758	0.42875	-4.98354	6.24E-07	3.08E-06
CPN2	48.93295	-2.714323702	0.424146	-6.39951	1.56E-10	9.86E-10
LRRC15	2897.117	-5.159514919	0.24609	-20.966	1.34E-97	5.14E-96
FAM43A	598.0811	2.243152369	0.150796	14.87537	4.76E-50	9.52E-49
LINC01968	28.47729	-2.223896151	0.446092	-4.98529	6.19E-07	3.05E-06
MUC4	51.98884	-2.785746012	0.419294	-6.64389	3.06E-11	2.01E-10
TFRC	6833.187	2.568764363	0.05266	48.78059	0	0
DOK7	944.7696	4.905916345	0.139302	35.21784	1.07E-271	1.23E-269
C4orf50	32.68788	-2.338228642	0.442813	-5.28039	1.29E-07	6.71E-07
CLNK	24.35596	-2.013681982	0.449278	-4.48204	7.39E-06	3.28E-05
HS3ST1	532.2008	-2.675980362	0.177453	-15.0799	2.19E-51	4.50E-50
LINC00504	42.32969	-2.76118511	0.434002	-6.36215	1.99E-10	1.25E-09
FGFBP1	88.36203	2.886515325	0.326491	8.841015	9.49E-19	8.63E-18
PPARGC1A	767.1709	-3.751701761	0.176399	-21.2683	2.23E-100	8.85E-99
SOD3	1169.739	-3.862640386	0.150677	-25.6353	6.17E-145	3.65E-143
NWD2	18.97912	-2.00203564	0.451184	-4.4373	9.11E-06	4.00E-05
ATP8A1	1875.453	-3.161432889	0.105805	-29.8799	3.59E-196	2.83E-194

LNX1	1028.635	-2.060353316	0.146595	-14.0547	7.21E-45	1.31E-43
KIT	207.6837	-3.065770903	0.284485	-10.7766	4.44E-27	5.26E-26
EPHA5	220.8397	-2.677596495	0.259401	-10.3222	5.59E-25	6.24E-24
MT2P1	317.1828	2.026897215	0.194083	10.44346	1.57E-25	1.78E-24
SULT1B1	149.5996	3.105661595	0.269052	11.54298	8.01E-31	1.05E-29
SULT1E1	57.12371	3.422251648	0.395393	8.655327	4.92E-18	4.37E-17
RASSF6	685.5833	-2.353004698	0.180392	-13.0439	6.89E-39	1.10E-37
CXCL8	146.0751	3.153771653	0.272244	11.58436	4.95E-31	6.52E-30
PARM1	1873.746	-3.159959696	0.108554	-29.1096	2.71E-186	2.03E-184
ANXA3	7197.292	3.121791023	0.054864	56.90095	0	0
LINC00989	20.47456	-2.059706597	0.451315	-4.56379	5.02E-06	2.27E-05
CCSER1	135.4459	-3.681262335	0.357532	-10.2963	7.32E-25	8.15E-24
BMPR1B	304.3237	-2.087307511	0.252716	-8.25951	1.46E-16	1.23E-15
UNC5C	50.91928	-2.955822692	0.427606	-6.91249	4.76E-12	3.26E-11
STPG2-AS	23.91663	-2.272105538	0.450877	-5.0393	4.67E-07	2.33E-06
ETNPPL	111.4714	-2.080306286	0.319252	-6.5162	7.21E-11	4.65E-10
EGF	561.3808	2.159563636	0.147964	14.59516	3.01E-48	5.86E-47
ARSJ	1769.131	2.076876522	0.084136	24.68479	1.56E-134	8.62E-133
UGT8	295.6704	-2.425531888	0.225388	-10.7616	5.23E-27	6.18E-26
METTL14-	43.28144	-2.101812475	0.423145	-4.96712	6.80E-07	3.33E-06
SYNPO2	634.7852	-3.158698103	0.176531	-17.8931	1.33E-71	3.76E-70
NDNF	187.2086	-3.066661952	0.29058	-10.5536	4.89E-26	5.65E-25
TRPC3	18.99529	-2.000128929	0.451173	-4.43317	9.29E-06	4.07E-05
IL21-AS1	185.2182	3.15971454	0.248515	12.7144	4.92E-37	7.51E-36
FGF2	11837.35	2.882032081	0.04151	69.42942	0	0
SPRY1	542.5007	2.686816036	0.15098	17.79589	7.61E-71	2.13E-69
LINC01091	31.93176	-2.111953496	0.441468	-4.78394	1.72E-06	8.15E-06
SLC7A11	609.5948	-2.080905011	0.155848	-13.3522	1.15E-40	1.90E-39
LINC00499	33.16144	-2.410009975	0.442806	-5.44258	5.25E-08	2.83E-07
MGARP	214.6735	2.330288474	0.238038	9.789554	1.25E-22	1.30E-21
FAM160A1	1148.953	-2.647182495	0.123163	-21.4934	1.80E-102	7.23E-101
DCHS2	40.22136	-2.531352729	0.432695	-5.85021	4.91E-09	2.84E-08
FGG	27.39156	-2.406670562	0.44996	-5.34863	8.86E-08	4.68E-07
GUCY1A1	92.30555	-2.929014604	0.371523	-7.88381	3.18E-15	2.53E-14
GUCY1B1	424.6828	-2.595640302	0.193872	-13.3884	7.07E-41	1.17E-39
CPE	8237.543	-2.138201601	0.048396	-44.1814	0	0
LINC02268	27.11049	-2.090743888	0.447281	-4.67434	2.95E-06	1.37E-05
GPM6A	271.9337	-2.121456305	0.242269	-8.7566	2.01E-18	1.81E-17
CASP3	5441.866	2.429570616	0.051934	46.78228	0	0
ANKRD37	242.459	2.226670434	0.223428	9.965953	2.15E-23	2.29E-22
ZDHHC11	141.9778	-2.358969618	0.298823	-7.89421	2.92E-15	2.33E-14
ADCY2	36.73612	-2.836347421	0.44349	-6.39551	1.60E-10	1.01E-09
SEMA5A	1609.898	-2.151135578	0.101395	-21.2153	6.90E-100	2.71E-98
AC138951.	27.2347	-2.16183691	0.447194	-4.83423	1.34E-06	6.41E-06
PRLR	58.52551	-3.475340173	0.428888	-8.10314	5.36E-16	4.42E-15
DAB2	7471.762	2.149023533	0.047149	45.57956	0	0
C7	32.12748	-2.655697205	0.446656	-5.94573	2.75E-09	1.62E-08

MROH2B	25.51771	-2.074764781	0.448517	-4.62583	3.73E-06	1.71E-05
C6	37.74477	-2.869181577	0.442942	-6.47755	9.32E-11	5.98E-10
SELENOP	402.8088	-3.243999056	0.228571	-14.1925	1.02E-45	1.89E-44
NIM1K	162.2643	-2.353244093	0.284466	-8.27251	1.31E-16	1.11E-15
LINC01948	26.67514	-2.422257958	0.449626	-5.38727	7.15E-08	3.81E-07
PDE4D	399.912	-2.684390016	0.201481	-13.3233	1.69E-40	2.79E-39
PART1	21.38346	-2.136408987	0.451351	-4.73337	2.21E-06	1.04E-05
C5orf64	53.78099	-3.359541024	0.431799	-7.78033	7.23E-15	5.65E-14
RGS7BP	52.89861	2.111825342	0.392707	5.377607	7.55E-08	4.01E-07
ENC1	21177.57	3.470553392	0.033725	102.907	0	0
SV2C	28.19105	-2.493955592	0.448825	-5.55663	2.75E-08	1.51E-07
ZBED3-AS	232.8102	-2.406277948	0.25339	-9.49635	2.17E-21	2.17E-20
ACTBP2	426.4526	2.143480022	0.174347	12.29433	9.72E-35	1.40E-33
CKMT2	118.7702	-2.312633093	0.322391	-7.17338	7.32E-13	5.20E-12
LINC00461	23.7921	-2.276553746	0.450822	-5.04979	4.42E-07	2.21E-06
TSLP	411.831	3.208012686	0.173735	18.46499	3.95E-76	1.17E-74
ARL14EPL	42.1742	-2.28545437	0.424266	-5.38685	7.17E-08	3.82E-07
SEMA6A	606.1447	-2.184539503	0.157464	-13.8732	9.20E-44	1.63E-42
ACSL6	21.97861	-2.175016942	0.451272	-4.81975	1.44E-06	6.88E-06
LRRTM2	40.24288	-2.690127579	0.435861	-6.17198	6.74E-10	4.11E-09
HBEGF	1294.923	2.159097541	0.099149	21.77632	3.89E-105	1.62E-103
SPRY4	533.0563	2.402162641	0.15213	15.79019	3.63E-56	8.12E-55
FGF1	1364.197	5.276404158	0.12793	41.24451	0	0
C5orf46	1026.087	2.803513568	0.112736	24.86789	1.66E-136	9.24E-135
AC011352.	79.17498	3.174448844	0.358816	8.847001	8.99E-19	8.18E-18
AC011352.	258.447	3.464191671	0.217106	15.95621	2.58E-57	5.90E-56
ARHGEF37	2716.446	-2.180397118	0.078807	-27.6674	1.72E-168	1.17E-166
CD74	4203.503	-2.565570875	0.06857	-37.4153	2.21E-306	3.04E-304
NDST1	22562.38	2.425762377	0.033787	71.79588	0	0
TNIP1	11824.95	2.155472076	0.040508	53.2112	0	0
FAT2	46.56988	-2.762215642	0.428434	-6.44723	1.14E-10	7.26E-10
SGCD	147.5047	-3.356938032	0.329977	-10.1732	2.61E-24	2.86E-23
ITK	36.42774	-2.0593009	0.435993	-4.72324	2.32E-06	1.09E-05
EBF1	66.78186	-2.295238005	0.390583	-5.87644	4.19E-09	2.44E-08
ATP10B	373.9516	-2.308420675	0.204332	-11.2974	1.35E-29	1.71E-28
GABRG2	27.24225	-2.454529359	0.44925	-5.46362	4.67E-08	2.52E-07
TENM2	551.2221	2.000333332	0.145792	13.7205	7.65E-43	1.33E-41
FGF18	1044.184	4.1108307	0.122786	33.47976	9.50E-246	9.76E-244
STC2	13619.08	2.347615455	0.038779	60.5377	0	0
LINC01411	39.14939	-2.600058375	0.436666	-5.95435	2.61E-09	1.54E-08
AC145098.	712.1869	2.485623545	0.139876	17.77021	1.20E-70	3.36E-69
FAM153CP	29.4137	-2.223136122	0.446748	-4.97627	6.48E-07	3.19E-06
LINC01622	23.98939	-2.28805282	0.450739	-5.07622	3.85E-07	1.93E-06
FOXQ1	928.3368	-3.40630125	0.151973	-22.4138	2.89E-111	1.30E-109
PXDC1	3054.22	2.228553928	0.072125	30.89837	1.26E-209	1.08E-207
RNA5SP20	126.9686	-4.266415653	0.390695	-10.9201	9.24E-28	1.12E-26
PPP1R3G	72.92213	2.699491873	0.357655	7.547748	4.43E-14	3.34E-13

LY86-AS1	28.5048	-2.51274807	0.448564	-5.60176	2.12E-08	1.18E-07
NEDD9	1436.396	3.609504335	0.099942	36.11614	1.27E-285	1.58E-283
EDN1	1024.419	4.951257832	0.14618	33.87098	1.78E-251	1.88E-249
KRT18P38	30.86026	2.123909418	0.428339	4.958482	7.10E-07	3.48E-06
RIPOR2	116.9332	-2.14472738	0.325803	-6.58291	4.61E-11	3.01E-10
IER3	4510.649	3.4122237	0.06587	51.80256	0	0
SLC44A4	35.79269	-2.047075224	0.435752	-4.6978	2.63E-06	1.22E-05
HLA-DRB1	25.73064	-2.36371034	0.450209	-5.25026	1.52E-07	7.85E-07
HLA-DMB	36.74368	-2.842715298	0.443342	-6.41201	1.44E-10	9.10E-10
HLA-DOA	23.73224	-2.254339687	0.450978	-4.99878	5.77E-07	2.85E-06
AL138889.	23.32148	-2.234386789	0.451083	-4.95338	7.29E-07	3.56E-06
KCTD20	6051.077	2.212092893	0.05071	43.62277	0	0
CPNE5	232.8809	-3.143197699	0.269899	-11.6458	2.41E-31	3.20E-30
DNAH8	26.99697	-2.442845104	0.449379	-5.43605	5.45E-08	2.93E-07
DLK2	159.2316	2.030917587	0.250451	8.109028	5.10E-16	4.21E-15
TMEM151B	79.70387	-2.108573464	0.360368	-5.85116	4.88E-09	2.82E-08
ADGRF1	26.51448	-2.419434151	0.449625	-5.381	7.41E-08	3.94E-07
C6orf141	2421.773	-2.594003605	0.343942	-7.54198	4.63E-14	3.49E-13
PKHD1	47.70495	-3.196298272	0.435768	-7.33486	2.22E-13	1.62E-12
AL109918.	4229.334	-3.454669193	0.07703	-44.8483	0	0
GSTA1	25.03469	-2.34572965	0.45031	-5.20914	1.90E-07	9.72E-07
ELOVL5	21015.93	-2.316864246	0.036862	-62.8522	0	0
AL034374.	64.90799	-2.255251172	0.391959	-5.7538	8.73E-09	4.96E-08
ADGRB3	33.26654	-2.425481766	0.4422	-5.48503	4.13E-08	2.24E-07
COL19A1	20.64382	-2.104264408	0.45137	-4.66195	3.13E-06	1.45E-05
COL9A1	34.11486	-2.39827829	0.441586	-5.43106	5.60E-08	3.01E-07
OGFRL1	4135.216	2.597846675	0.058821	44.16512	0	0
LCAL1	20.3306	-2.033213942	0.451236	-4.50588	6.61E-06	2.95E-05
RRAGD	809.1538	-2.319027638	0.139357	-16.641	3.52E-62	8.65E-61
AL132996.	19.95217	-2.062624002	0.451345	-4.56995	4.88E-06	2.21E-05
GRIK2	119.39	-2.475355485	0.3275	-7.55834	4.08E-14	3.09E-13
LINC02532	38.64318	-2.556770713	0.438172	-5.83509	5.38E-09	3.10E-08
SLC16A10	133.089	-2.014538058	0.300391	-6.70639	1.99E-11	1.32E-10
FRK	115.4463	-2.304829644	0.320338	-7.19499	6.25E-13	4.45E-12
VGLL2	158.6109	2.405858366	0.258282	9.314867	1.22E-20	1.19E-19
MAN1A1	6614.24	-2.963419416	0.058875	-50.3344	0	0
AL121938.	209.1471	-3.299190847	0.287935	-11.4581	2.14E-30	2.78E-29
LAMA2	258.479	-2.374161176	0.238185	-9.96772	2.11E-23	2.25E-22
CCN2	34584.37	7.083224064	0.043764	161.8498	0	0
TARID	72.0542	-3.249040771	0.404488	-8.03247	9.55E-16	7.80E-15
AL078590.	41.13699	-2.255079912	0.424914	-5.30714	1.11E-07	5.82E-07
AL138828.	54.3801	-2.144059461	0.410349	-5.22497	1.74E-07	8.95E-07
LINC01625	26.8444	-2.425214828	0.449603	-5.39412	6.89E-08	3.67E-07
STX11	463.1438	4.156948752	0.182446	22.78458	6.52E-115	3.06E-113
IYD	26.23308	-2.404030675	0.449785	-5.34485	9.05E-08	4.77E-07
OPRM1	25.94737	-2.388437625	0.449949	-5.30824	1.11E-07	5.78E-07
AL360169.	51.47263	-3.299733892	0.433294	-7.61546	2.63E-14	2.00E-13

EZR	112038.7	2.683455385	0.025792	104.0432	0	0
C6orf99	1401.441	4.460123984	0.112867	39.5168	0	0
AL356417.	35.25254	-2.762764403	0.444982	-6.20871	5.34E-10	3.28E-09
SOD2	15444.84	-2.17876916	0.039678	-54.911	0	0
MAS1	23.76784	-2.262862206	0.450932	-5.01819	5.22E-07	2.59E-06
SLC22A3	1815.153	2.897349476	0.085754	33.7869	3.07E-250	3.21E-248
LPA	25.92742	-2.390674122	0.449909	-5.31368	1.07E-07	5.62E-07
PACRG	174.5754	-2.635100364	0.288247	-9.14182	6.14E-20	5.84E-19
PDE10A	402.4719	-3.809372594	0.23709	-16.0672	4.33E-58	1.01E-56
ELFN1	308.7758	2.643954593	0.192252	13.75258	4.91E-43	8.59E-42
LFNG	154.9696	2.512098527	0.284947	8.816013	1.19E-18	1.07E-17
AC005532.	40.12749	-2.209373948	0.430721	-5.12948	2.91E-07	1.47E-06
CHN2	27.8897	-2.484220224	0.448908	-5.53391	3.13E-08	1.71E-07
AQP1	364.996	-2.135680134	0.21001	-10.1694	2.71E-24	2.97E-23
BMPER	456.4385	-2.161348382	0.182024	-11.874	1.62E-32	2.21E-31
AOAH	36.21374	-2.547274468	0.439348	-5.79785	6.72E-09	3.85E-08
AMPH	513.2798	-2.802890075	0.194969	-14.3761	7.31E-47	1.38E-45
INHBA	4073.393	2.369681365	0.061966	38.2415	0	0
IGFBP3	14841.72	3.425299682	0.037404	91.57697	0	0
C7orf69	111.8731	2.453449156	0.294633	8.32714	8.28E-17	7.05E-16
CALN1	22.63413	-2.217760653	0.451115	-4.91618	8.82E-07	4.29E-06
ELN	730.5667	-4.102083528	0.195107	-21.0247	3.89E-98	1.50E-96
UPK3B	7825.307	2.096828233	0.051305	40.86967	0	0
CD36	155.4073	-2.049208899	0.280714	-7.29999	2.88E-13	2.08E-12
ABCB1	1882.2	2.283725169	0.088012	25.94775	1.93E-148	1.17E-146
ADAM22	365.2786	-2.169737095	0.204562	-10.6067	2.77E-26	3.22E-25
PEG10	3411.227	-2.561128181	0.075829	-33.7752	4.57E-250	4.76E-248
PDK4	3572.452	-5.230667993	0.119303	-43.8436	0	0
DLX6-AS1	30.12152	-2.273890497	0.445594	-5.10306	3.34E-07	1.68E-06
NPTX2	407.6622	-2.004341171	0.185387	-10.8117	3.03E-27	3.61E-26
MCM7	6507.96	2.177452936	0.051347	42.40656	0	0
MUC3A	46.12049	-2.671333684	0.428902	-6.22831	4.71E-10	2.90E-09
MUC12	64.45767	-2.266152798	0.39499	-5.73725	9.62E-09	5.46E-08
MUC17	36.82453	-2.843214841	0.443328	-6.41335	1.42E-10	9.03E-10
SERPINE1	70493.94	3.522894769	0.026663	132.1259	0	0
CDHR3	130.189	-2.394135854	0.32023	-7.4763	7.64E-14	5.70E-13
DOCK4	1686.622	-2.791695578	0.109753	-25.4361	1.01E-142	5.86E-141
TES	9360.761	2.325313745	0.044293	52.49865	0	0
PTPRZ1	85.42125	-2.133162073	0.351364	-6.07109	1.27E-09	7.63E-09
AC006148.	31.22205	-2.212872791	0.442654	-4.99911	5.76E-07	2.85E-06
KCP	796.1036	-3.103603632	0.15686	-19.7859	3.94E-87	1.34E-85
STRIP2	13319.47	-3.894010309	0.136966	-28.4304	8.51E-178	6.13E-176
RNU1-72P	19.81955	-2.053495588	0.45133	-4.54988	5.37E-06	2.42E-05
CPA4	45434.83	2.136980538	0.030212	70.73372	0	0
MEST	11102.7	2.951092408	0.043927	67.18105	0	0
CALD1	52520.04	2.26043928	0.028232	80.06575	0	0
PARP12	531.2906	2.133809608	0.148891	14.33135	1.39E-46	2.62E-45

WDR86	5605.532	-2.944365411	0.063914	-46.068	0	0
WDR86-AS	133.1091	-2.220008008	0.30334	-7.31854	2.51E-13	1.82E-12
DPP6	41.0049	-2.062236773	0.422205	-4.88444	1.04E-06	5.02E-06
AC006062.	805.9125	3.262764962	0.448282	7.278373	3.38E-13	2.44E-12
LINC01203	397.7033	4.140240325	0.207008	20.00036	5.47E-89	1.91E-87
PCYT1B	145.4159	-2.243473197	0.299933	-7.47992	7.44E-14	5.55E-13
DMD	1248.309	2.503753152	0.100215	24.98388	9.15E-138	5.15E-136
TSPAN7	294.5794	-2.480388176	0.22587	-10.9815	4.69E-28	5.71E-27
NYX	137.9266	-3.995757146	0.368625	-10.8396	2.23E-27	2.67E-26
MAOA	645.1471	-2.828233171	0.181726	-15.5632	1.29E-54	2.81E-53
NDP	159.2451	-3.052873833	0.309393	-9.8673	5.77E-23	6.07E-22
NHSL2	107.2809	-2.245619047	0.33196	-6.76473	1.34E-11	8.95E-11
RTL5	1227.445	-2.089480596	0.112369	-18.5948	3.54E-77	1.07E-75
TSIX	41.85529	-2.744472461	0.434456	-6.31703	2.67E-10	1.67E-09
KRT18P11	189.793	3.03685733	0.258182	11.76246	6.09E-32	8.23E-31
TSC22D3	2567.135	-2.003842449	0.084087	-23.8305	1.61E-125	8.20E-124
COL4A6	1857.314	-2.487116151	0.096829	-25.6856	1.69E-145	1.01E-143
TMEM164	10665.87	-2.468382373	0.051271	-48.1437	0	0
CAPN6	747.6639	2.211644968	0.13584	16.28122	1.34E-59	3.19E-58
DCX	28.43719	-2.142821533	0.446615	-4.79792	1.60E-06	7.63E-06
XACT	215.2296	-4.990508177	0.370549	-13.4679	2.42E-41	4.06E-40
IGSF1	275.0212	-3.233963922	0.255986	-12.6333	1.38E-36	2.09E-35
SMIM10L2	237.5332	-3.349746035	0.275241	-12.1702	4.48E-34	6.37E-33
ARHGEF6	1119.372	-2.995158456	0.133035	-22.5141	3.02E-112	1.37E-110
GABRA3	502.0008	-2.760172551	0.190579	-14.4831	1.55E-47	2.97E-46
MPP1	2570.79	-2.334605427	0.08208	-28.4429	5.97E-178	4.31E-176
CSMD1	54.20932	-2.650439658	0.411536	-6.44035	1.19E-10	7.59E-10
PRAG1	3839.683	3.032054109	0.079851	37.97137	0	0
AC107918.	31.24442	-2.089036548	0.441774	-4.72874	2.26E-06	1.06E-05
SLC7A2	966.3476	-4.527195534	0.186845	-24.2297	1.08E-129	5.76E-128
SH2D4A	2964.376	2.273168376	0.076184	29.83786	1.26E-195	9.87E-194
GFRA2	66.39066	-2.185795988	0.380628	-5.74261	9.32E-09	5.29E-08
PHYHIP	1305.626	-2.432259191	0.113008	-21.5228	9.52E-103	3.84E-101
NKX3-1	163.1392	3.299709184	0.282432	11.68318	1.55E-31	2.08E-30
STC1	2743.5	2.252716758	0.070124	32.12498	1.98E-226	1.86E-224
NEFM	333.5533	-3.311659488	0.238161	-13.9051	5.89E-44	1.05E-42
NEFL	94.99795	-2.741590451	0.365455	-7.50185	6.29E-14	4.71E-13
CLU	31116.15	-2.129676035	0.033244	-64.0613	0	0
SCARA5	84.847	-3.9797743	0.415467	-9.57904	9.80E-22	9.91E-21
PURG	132.1122	-2.077886792	0.314851	-6.59958	4.12E-11	2.70E-10
UNC5D	1557.338	-2.006194476	0.099321	-20.1991	9.98E-91	3.55E-89
AC124290.	22.73493	-2.212225463	0.45115	-4.90352	9.41E-07	4.57E-06
TCIM	120.1634	3.332483513	0.296664	11.23319	2.80E-29	3.52E-28
LINC00293	34.37546	-2.759080367	0.444813	-6.20278	5.55E-10	3.40E-09
EFCAB1	83.33333	-3.233827351	0.390698	-8.27706	1.26E-16	1.07E-15
LINC00968	426.9654	2.994942156	0.184585	16.22531	3.34E-59	7.90E-58
KRT8P3	392.868	2.864765514	0.174725	16.39584	2.05E-60	4.93E-59

NKAIN3	147.2204	-2.210108447	0.293559	-7.52867	5.13E-14	3.86E-13
MSC	476.8012	2.639980481	0.159953	16.50472	3.39E-61	8.25E-60
LINC01111	73.02459	2.525666206	0.350295	7.210118	5.59E-13	3.99E-12
CA3-AS1	45.75457	-2.16654165	0.417671	-5.1872	2.13E-07	1.09E-06
CA2	115.2144	-2.665708748	0.334085	-7.97913	1.47E-15	1.19E-14
CNGB3	175.5334	-4.520996621	0.366872	-12.3231	6.80E-35	9.84E-34
AC090572.	45.84518	-2.513755672	0.427846	-5.87538	4.22E-09	2.45E-08
LINC00534	23.14329	-2.093843519	0.450465	-4.64819	3.35E-06	1.54E-05
GEM	6947.522	-3.305898998	0.06109	-54.1156	0	0
GDF6	3790.504	4.645438482	0.073197	63.46465	0	0
AP003548.	23.60289	-2.240031843	0.451075	-4.96598	6.84E-07	3.35E-06
SDC2	22038.69	-2.16759248	0.035049	-61.8445	0	0
NCALD	1008.476	-5.017890688	0.20481	-24.5002	1.47E-132	7.97E-131
BAALC	328.4404	-4.666672439	0.303072	-15.3979	1.69E-53	3.58E-52
AC012213.	24.42337	-2.308144567	0.45062	-5.12215	3.02E-07	1.52E-06
MAL2	3815.927	2.211021586	0.06051	36.53962	2.61E-292	3.38E-290
DEPTOR	87.74029	-2.477814865	0.361279	-6.85846	6.96E-12	4.73E-11
HAS2	63.56761	-3.580637311	0.426318	-8.39898	4.50E-17	3.87E-16
LINC01151	30.81264	-2.602895757	0.447429	-5.81745	5.98E-09	3.43E-08
AC016405.	68.33749	-2.13675448	0.376267	-5.67883	1.36E-08	7.62E-08
ANXA13	66.76881	-3.044857059	0.40589	-7.50168	6.30E-14	4.72E-13
FER1L6	55.77237	-2.903418021	0.418023	-6.94559	3.77E-12	2.59E-11
LINC00964	34.29135	-2.743294098	0.445177	-6.16225	7.17E-10	4.37E-09
KNOP1P5	23.05455	2.089651668	0.445887	4.686502	2.78E-06	1.29E-05
CASC19	61.07984	-2.176758418	0.391062	-5.56627	2.60E-08	1.43E-07
CCDC26	101.7777	-3.122715786	0.363686	-8.58629	8.98E-18	7.93E-17
GSDMC	44.54651	-2.47262032	0.429005	-5.76361	8.23E-09	4.70E-08
ADCY8	114.8879	-3.26385619	0.357247	-9.13613	6.47E-20	6.15E-19
LINC02055	58.16862	-2.9548866	0.416784	-7.08973	1.34E-12	9.45E-12
FAM135B	34.36738	-2.757912372	0.444844	-6.19972	5.66E-10	3.46E-09
COL22A1	666.6538	-3.327562716	0.174925	-19.0228	1.10E-80	3.50E-79
EPPK1	855.6732	4.326830492	0.137189	31.53928	2.52E-218	2.26E-216
SCX	1455.383	2.634218635	0.098306	26.79598	3.60E-158	2.30E-156
AL449043.	39.48846	-2.196545183	0.426805	-5.14648	2.65E-07	1.35E-06
GLIS3	2979.429	-2.211024191	0.078699	-28.0947	1.14E-173	8.01E-172
PDCD1LG2	196.9137	3.039031033	0.241342	12.5922	2.33E-36	3.51E-35
PTPRD	1542.163	-3.380088175	0.127177	-26.5778	1.22E-155	7.70E-154
FREM1	33.32691	-2.42352576	0.442314	-5.4792	4.27E-08	2.32E-07
ADAMTSL1	1367.146	-2.129074189	0.108972	-19.5378	5.24E-85	1.74E-83
PLIN2	33191.51	-2.904457276	0.035858	-80.9983	0	0
ALDH1B1	11745.54	2.384214953	0.041216	57.8463	0	0
PIP5K1B	663.7536	-2.859309497	0.16371	-17.4656	2.62E-68	7.09E-67
TRPM3	2205.082	-3.010334726	0.096194	-31.2945	5.55E-215	4.81E-213
LINC01504	108.5493	-2.67725846	0.354163	-7.55939	4.05E-14	3.06E-13
ALDH1A1	1479.681	-2.012040384	0.108261	-18.5851	4.24E-77	1.27E-75
RORB	120.2035	-2.906607206	0.339063	-8.57247	1.01E-17	8.92E-17
PCA3	27.22316	-2.09605513	0.447191	-4.68716	2.77E-06	1.29E-05

RASEF	1275.749	-2.91394121	0.121686	-23.9463	1.01E-126	5.17E-125
AL499602.	175.5288	-3.171385724	0.30658	-10.3444	4.44E-25	4.97E-24
AL159996.	326.2224	-5.755114057	0.369798	-15.5628	1.30E-54	2.82E-53
GAS1	7257.398	-4.644179438	0.07612	-61.0112	0	0
DIRAS2	33.42374	-2.368518804	0.442185	-5.3564	8.49E-08	4.49E-07
ROR2	935.271	-2.218491495	0.132269	-16.7725	3.88E-63	9.63E-62
FGD3	309.3948	3.930532512	0.208816	18.82295	4.90E-79	1.52E-77
ZNF367	105.5061	2.23764111	0.308869	7.244622	4.34E-13	3.11E-12
GABBR2	27.63486	-2.293987392	0.447755	-5.1233	3.00E-07	1.52E-06
MUSK	21.78183	-2.168507771	0.451281	-4.80522	1.55E-06	7.37E-06
DELEC1	608.2956	2.033479666	0.148064	13.73383	6.37E-43	1.11E-41
AL160272.	25.39748	-2.061767082	0.448722	-4.59475	4.33E-06	1.97E-05
PTGS1	285.3429	-2.854474668	0.245418	-11.6311	2.87E-31	3.80E-30
ANGPTL2	3526.379	-2.397934032	0.073061	-32.8212	2.94E-236	2.89E-234
PTGES	24833.18	-2.779062085	0.037333	-74.4408	0	0
HMCN2	49.92766	-2.467758292	0.417492	-5.91091	3.40E-09	1.99E-08
AIF1L	611.3924	2.294284021	0.144342	15.89481	6.88E-57	1.56E-55
PTGDS	3398.833	-3.016334061	0.078457	-38.4459	0	0
LCNL1	5295.574	-2.391210658	0.061975	-38.5838	0	0
RASSF7	5230.648	2.436488982	0.052716	46.21959	0	0
MIR210HG	1792.135	3.356849405	0.090972	36.89995	4.63E-298	6.08E-296
MIR210	113.1526	3.498181034	0.310048	11.28272	1.60E-29	2.02E-28
IRF7	659.2498	2.692319441	0.156229	17.23315	1.50E-66	3.95E-65
DRD4	631.3554	2.186084435	0.139489	15.67204	2.35E-55	5.17E-54
CEND1	3813.394	-2.25417104	0.07535	-29.9158	1.23E-196	9.71E-195
MUC2	19.75109	-2.049792983	0.451322	-4.54175	5.58E-06	2.51E-05
MUC5AC	26.36948	-2.402189492	0.449851	-5.33997	9.30E-08	4.90E-07
PHLDA2	2266.577	2.805365439	0.079134	35.45102	2.80E-275	3.31E-273
STIM1	6504.262	-2.09920531	0.053443	-39.2796	0	0
OR51E2	318.9094	-3.122238352	0.242925	-12.8527	8.31E-38	1.29E-36
DCHS1	237.6846	-2.202810196	0.241441	-9.12359	7.27E-20	6.89E-19
OLFML1	120.8341	2.604256771	0.310415	8.389604	4.88E-17	4.19E-16
ABCC8	26.52635	-2.414279415	0.449711	-5.36851	7.94E-08	4.22E-07
LDHA	41317.67	2.233355069	0.029407	75.94692	0	0
IGSF22	134.6539	-2.030042801	0.300575	-6.75387	1.44E-11	9.63E-11
AC131571.	204.8794	3.30490972	0.238493	13.85748	1.15E-43	2.03E-42
WT1	19.60231	-2.040281565	0.451302	-4.52088	6.16E-06	2.75E-05
SLC1A2	61.2056	-3.010830441	0.412363	-7.30141	2.85E-13	2.06E-12
C11orf96	245.3748	-3.20332762	0.266517	-12.0192	2.82E-33	3.93E-32
CHRM4	386.0921	-4.651651173	0.284347	-16.3591	3.75E-60	9.01E-59
OR5AS1	19.06753	-2.007263719	0.451202	-4.4487	8.64E-06	3.80E-05
RTN4RL2	68.45909	-2.200589217	0.377533	-5.82886	5.58E-09	3.21E-08
TCN1	143.7691	-2.543112723	0.302754	-8.39994	4.47E-17	3.84E-16
MS4A6A	19.10795	-2.000651825	0.451167	-4.43439	9.23E-06	4.05E-05
RPLP0P2	874.8538	-2.380276837	0.140217	-16.9757	1.24E-64	3.18E-63
RAB3IL1	316.4585	-2.023670978	0.21217	-9.53797	1.46E-21	1.47E-20
BEST1	195.3302	-2.140646788	0.28843	-7.42172	1.16E-13	8.55E-13

NRXN2	71.53045	-3.254435653	0.406697	-8.00211	1.22E-15	9.93E-15
FOSL1	1504.58	2.633290678	0.093139	28.27264	7.50E-176	5.33E-174
PC	2065.894	-2.281849511	0.096794	-23.5743	7.07E-123	3.52E-121
CCND1	49873.97	4.5281657	0.032098	141.0731	0	0
ATG16L2	2264.176	-2.292188677	0.089658	-25.5658	3.66E-144	2.15E-142
P4HA3	923.7718	-2.49782494	0.145715	-17.1418	7.24E-66	1.89E-64
LRRC32	1540.362	3.016553952	0.093614	32.22334	8.31E-228	7.89E-226
MYO7A	8488.837	-4.670420341	0.071731	-65.1102	0	0
GDPD4	36.92911	-2.84450306	0.443326	-6.41628	1.40E-10	8.86E-10
AP001284.	83.42056	2.753922464	0.360408	7.641115	2.15E-14	1.65E-13
CCDC81	176.1011	2.642989762	0.256979	10.28483	8.25E-25	9.18E-24
AP001528.	157.764	-2.121464415	0.285699	-7.42554	1.12E-13	8.31E-13
RAB38	1453.094	-2.027757822	0.106116	-19.1089	2.13E-81	6.81E-80
TYR	1437.462	-5.75121852	0.207418	-27.7276	3.24E-169	2.23E-167
LINC02552	25.15975	-2.322993209	0.450566	-5.15572	2.53E-07	1.28E-06
GRIA4	24.07592	-2.145543094	0.450017	-4.76769	1.86E-06	8.80E-06
GUCY1A2	983.7942	-2.557954237	0.136759	-18.7041	4.59E-78	1.40E-76
LINC02732	186.6715	-5.028123411	0.38745	-12.9775	1.64E-38	2.59E-37
IL18	7601.9	3.194037596	0.046887	68.12216	0	0
AP000880.	43.03186	-2.119413833	0.420881	-5.03566	4.76E-07	2.37E-06
ZBTB16	19.56997	-2.024009813	0.451239	-4.48545	7.28E-06	3.23E-05
SIK3	3059.766	-2.093229863	0.074612	-28.0548	3.49E-173	2.44E-171
TAGLN	76777.98	2.334080728	0.028665	81.42708	0	0
TMPRSS4	27.20236	-2.146137641	0.447632	-4.79442	1.63E-06	7.76E-06
SCN2B	69.04509	-2.469192224	0.383602	-6.43686	1.22E-10	7.76E-10
PHLDB1	16865.94	2.013351305	0.035093	57.37204	0	0
MFRP	287.0339	-3.709241135	0.269605	-13.7581	4.56E-43	7.97E-42
GRIK4	137.3928	-3.208670583	0.337499	-9.50719	1.96E-21	1.96E-20
AP001993.	28.18297	-2.490568765	0.448884	-5.54836	2.88E-08	1.58E-07
ADAMTS15	7737.692	2.531664768	0.04577	55.31259	0	0
GLB1L3	20.36241	-2.085452234	0.451367	-4.62031	3.83E-06	1.75E-05
AKR1C1	106.9122	-2.360230009	0.331029	-7.12999	1.00E-12	7.10E-12
AKR1C2	54.40954	-2.866169151	0.41926	-6.83626	8.13E-12	5.50E-11
AL392086.	31.57706	-2.330403586	0.444644	-5.24105	1.60E-07	8.24E-07
AL136452.	78.68946	2.520411292	0.33632	7.494081	6.68E-14	4.99E-13
SFTA1P	972.9318	2.311828974	0.114517	20.1877	1.26E-90	4.46E-89
PROSER2	555.5868	2.029806958	0.148656	13.65441	1.90E-42	3.28E-41
CAMK1D	916.0862	2.461771846	0.120852	20.37006	3.08E-92	1.11E-90
TMEM236	23.12092	-2.236415958	0.45104	-4.95835	7.11E-07	3.48E-06
SLC39A12	36.44285	-2.555382527	0.439179	-5.81854	5.94E-09	3.41E-08
LINC00836	20.10905	-2.071801063	0.451356	-4.59017	4.43E-06	2.02E-05
CXCL12	1501.19	-3.990593684	0.135598	-29.4296	2.30E-190	1.75E-188
ANXA8L1	297.5142	4.14838749	0.216888	19.1269	1.51E-81	4.83E-80
LINC00842	118.6301	2.927338649	0.297206	9.849537	6.89E-23	7.23E-22
GPRIN2	138.4634	2.958568477	0.278335	10.62951	2.17E-26	2.53E-25
ANXA8	720.5517	3.79259456	0.361288	10.49743	8.88E-26	1.02E-24
FO681492.	226.5123	2.202893063	0.243054	9.063387	1.26E-19	1.19E-18

AC245041.	395.4535	6.046242174	0.252337	23.96099	7.10E-127	3.64E-125
FRMPD2	22.32039	-2.194113165	0.451215	-4.86268	1.16E-06	5.58E-06
WDFY4	52.47188	-2.156750403	0.402408	-5.35961	8.34E-08	4.42E-07
VSTM4	567.8424	-2.380091294	0.168623	-14.1149	3.08E-45	5.64E-44
OGDHL	525.3998	-3.271125733	0.193374	-16.916	3.43E-64	8.71E-63
DKK1	466.6425	2.025508121	0.161311	12.55653	3.66E-36	5.48E-35
ANK3	404.3136	-2.337713271	0.191922	-12.1805	3.95E-34	5.62E-33
TMEM26	114.3227	-3.730549388	0.377201	-9.89007	4.60E-23	4.85E-22
ADAMTS14	5361.864	-2.188728515	0.064658	-33.8507	3.54E-251	3.72E-249
VSIR	3191.736	2.761103855	0.069437	39.76391	0	0
SPOCK2	2626.264	-5.926992872	0.16646	-35.6062	1.12E-277	1.34E-275
SYNPO2L	99.25641	2.059167583	0.306558	6.717052	1.85E-11	1.23E-10
LINC00595	68.32593	-2.44485772	0.385252	-6.34613	2.21E-10	1.39E-09
RGR	38.59759	-2.913595475	0.44189	-6.59348	4.30E-11	2.81E-10
ANKRD22	116.4022	-3.922334254	0.383734	-10.2215	1.59E-24	1.75E-23
ACTA2-AS1	108.7944	2.218880508	0.314853	7.047357	1.82E-12	1.27E-11
ACTA2	15896.64	3.49157916	0.038087	91.67322	0	0
ANKRD1	4325.425	6.998675416	0.10164	68.85732	0	0
PLCE1-AS1	196.3951	3.86550822	0.253563	15.24474	1.78E-52	3.73E-51
FRAT1	124.9005	2.553383695	0.292843	8.719292	2.80E-18	2.51E-17
ANKRD2	199.2542	2.892010415	0.247499	11.68492	1.52E-31	2.04E-30
SFRP5	517.2654	-3.088334781	0.189497	-16.2975	1.03E-59	2.45E-58
ABCC2	36.26709	-2.063725744	0.435365	-4.74022	2.13E-06	1.00E-05
COL17A1	55.27457	-2.673682709	0.411119	-6.50342	7.85E-11	5.05E-10
SORCS1	179.422	-3.757610699	0.326761	-11.4996	1.33E-30	1.73E-29
NRAP	27.98241	-2.472358734	0.44912	-5.5049	3.69E-08	2.01E-07
PNLIPRP3	23.76406	-2.279667903	0.450788	-5.05707	4.26E-07	2.12E-06
FGFR2	1269.943	-4.230514469	0.155999	-27.1189	5.90E-162	3.86E-160
DMBT1	22.57187	-2.0438295	0.450903	-4.53275	5.82E-06	2.61E-05
IQSEC3	332.1632	-4.73125755	0.307582	-15.3821	2.16E-53	4.56E-52
AC026369.	100.2932	-3.924888874	0.399095	-9.83447	8.00E-23	8.38E-22
AC125807.	411.7135	2.441495047	0.174019	14.03006	1.02E-44	1.84E-43
AC005906.	19.64651	-2.04203076	0.451305	-4.52473	6.05E-06	2.71E-05
C3AR1	65.10112	-2.250934298	0.383029	-5.87667	4.19E-09	2.43E-08
A2ML1	22.06565	-2.034157592	0.450881	-4.51152	6.44E-06	2.87E-05
RPL13AP2	370.3888	3.08724705	0.183363	16.83678	1.31E-63	3.29E-62
GPRC5A	30694.87	2.792343401	0.03407	81.95795	0	0
GRIN2B	83.76837	-2.904507254	0.380743	-7.62851	2.37E-14	1.81E-13
RERG	53.56805	-3.347519747	0.432192	-7.74545	9.52E-15	7.40E-14
PDE3A	201.5406	-4.910039456	0.372319	-13.1877	1.03E-39	1.67E-38
KCNJ8	290.4366	-3.549369389	0.261142	-13.5917	4.48E-42	7.66E-41
BCAT1	14271.66	2.188483167	0.037312	58.65363	0	0
OVCH1	51.39495	-2.367677774	0.417018	-5.67764	1.37E-08	7.67E-08
SLC2A13	297.1086	-3.030016517	0.242399	-12.5001	7.46E-36	1.11E-34
RAPGEF3	837.3068	3.516877311	0.127739	27.53183	7.30E-167	4.92E-165
VDR	276.5552	2.044409705	0.20236	10.10281	5.37E-24	5.82E-23
FAIM2	320.5869	-4.132265083	0.276133	-14.9648	1.25E-50	2.52E-49

LINC02395	43.66394	-3.079011228	0.438402	-7.02325	2.17E-12	1.51E-11
AQP2	54.3039	-3.365572193	0.431753	-7.79513	6.43E-15	5.03E-14
AC025154.	52.27971	-2.548307762	0.414119	-6.15357	7.58E-10	4.61E-09
AQP5	437.4586	-3.593060338	0.220858	-16.2686	1.65E-59	3.91E-58
GPD1	85.18079	-2.201840827	0.352545	-6.24555	4.22E-10	2.61E-09
METTL7A	646.6803	-2.815787955	0.164681	-17.0985	1.52E-65	3.97E-64
KRT7	317444.1	2.000363404	0.028969	69.05268	0	0
KRT8	322105.3	3.013701515	0.026045	115.712	0	0
KRT18	141819.7	3.522674737	0.02816	125.0936	0	0
GDF11	5563.383	-2.066513929	0.058596	-35.2671	1.88E-272	2.17E-270
PMEL	7654.382	-2.958592552	0.05655	-52.3186	0	0
ERBB3	193.1867	-2.291097965	0.263445	-8.6967	3.42E-18	3.05E-17
MYO1A	27.86166	-2.477837422	0.449005	-5.51851	3.42E-08	1.87E-07
GLI1	223.8639	-3.782516155	0.300502	-12.5873	2.48E-36	3.72E-35
CYP27B1	235.7457	-3.021461496	0.276519	-10.9268	8.58E-28	1.04E-26
AC083805.	27.29076	-2.443520468	0.449423	-5.43701	5.42E-08	2.92E-07
MSRB3	3806.946	2.076868032	0.068554	30.29549	1.31E-201	1.08E-199
HMGA2	4354.709	2.451829425	0.062432	39.27208	0	0
LINC02384	26.33011	-2.120796821	0.447778	-4.73627	2.18E-06	1.02E-05
TSPAN8	41.52212	-2.48462669	0.431104	-5.7634	8.24E-09	4.70E-08
OTOGL	20.48317	-2.082600082	0.451363	-4.61403	3.95E-06	1.80E-05
NTS	64.92072	-2.136964362	0.382922	-5.58068	2.40E-08	1.32E-07
MGAT4C	83.65295	-3.043287764	0.387035	-7.86307	3.75E-15	2.97E-14
DUSP6	250.2843	2.270370549	0.207791	10.9262	8.64E-28	1.05E-26
LINC02412	24.19479	-2.004633295	0.449422	-4.46047	8.18E-06	3.61E-05
ELK3	4063.843	2.12556321	0.062107	34.22436	1.05E-256	1.15E-254
DRAM1	7466.175	2.611442504	0.049272	53.00016	0	0
HELLPAR	322.7219	-3.761904039	0.259646	-14.4886	1.43E-47	2.74E-46
AC089983.	61.53634	2.85611999	0.377894	7.557983	4.09E-14	3.09E-13
CHST11	4065.522	-2.258223648	0.066725	-33.8437	4.49E-251	4.70E-249
NUAK1	2188.562	2.058433457	0.076428	26.93297	9.03E-160	5.81E-158
RPH3A	25.08321	-2.026504649	0.449401	-4.50935	6.50E-06	2.90E-05
RASAL1	301.0916	-2.610046499	0.228967	-11.3992	4.22E-30	5.44E-29
LINC01234	34.0256	-2.744725217	0.445074	-6.1669	6.96E-10	4.24E-09
NOS1	43.22998	-2.774734886	0.434087	-6.39212	1.64E-10	1.03E-09
BICDL1	1951.657	2.138133634	0.081648	26.18725	3.71E-151	2.28E-149
HPD	10166.91	-6.628826417	0.107601	-61.6054	0	0
AC079360.	24.16622	-2.300983039	0.450646	-5.10597	3.29E-07	1.66E-06
PITPNM2	6182.633	-3.035163034	0.063153	-48.0606	0	0
RFLNA	1171.4	3.168761359	0.11148	28.42457	1.01E-177	7.23E-176
SHISA2	239.4793	-3.033277628	0.271493	-11.1726	5.56E-29	6.93E-28
FLT1	24.14575	-2.273638944	0.450894	-5.04251	4.59E-07	2.29E-06
MEDAG	622.5595	3.25499579	0.156982	20.73486	1.68E-95	6.31E-94
DCLK1	1483.321	-4.052401648	0.140115	-28.922	6.31E-184	4.69E-182
LINC00547	62.6441	2.67378575	0.368874	7.248514	4.21E-13	3.03E-12
AL354809.	36.35718	-2.390079518	0.436447	-5.47622	4.35E-08	2.35E-07
LINC00598	81.89331	-3.246806953	0.394203	-8.23639	1.77E-16	1.49E-15

RGCC	182.4103	-3.86729119	0.33006	-11.7169	1.04E-31	1.40E-30
NRAD1	69.61463	2.495455722	0.360094	6.930008	4.21E-12	2.89E-11
AL589745.	25.28103	-2.034702497	0.449252	-4.52909	5.92E-06	2.66E-05
CPB2-AS1	82.15338	-2.176467344	0.361899	-6.01402	1.81E-09	1.08E-08
LCP1	500.7011	-2.613364144	0.19034	-13.73	6.72E-43	1.17E-41
CYSLTR2	39.07251	-2.900803117	0.442427	-6.55656	5.51E-11	3.58E-10
FAM124A	2216.231	-2.203428267	0.088566	-24.8789	1.26E-136	7.04E-135
SERPINE3	16021.49	-2.648439791	0.040171	-65.9292	0	0
PCDH17	428.1268	-3.261312994	0.2111	-15.4492	7.64E-54	1.63E-52
DACH1	1255.884	-2.353670715	0.115781	-20.3286	7.18E-92	2.58E-90
LMO7	1192.825	2.2732864	0.120896	18.80359	7.06E-79	2.19E-77
AL158064.	25.25194	-2.355711366	0.450226	-5.23228	1.67E-07	8.62E-07
SLITRK6	27.26221	-2.450518933	0.449326	-5.45377	4.93E-08	2.66E-07
SLITRK5	555.6469	-2.378778981	0.166723	-14.2678	3.47E-46	6.49E-45
GPC5	86.60548	-3.532299669	0.39718	-8.89345	5.92E-19	5.43E-18
DCT	637.0398	-6.615484513	0.351254	-18.8339	3.98E-79	1.24E-77
CCDC168	33.32586	-2.71406094	0.445629	-6.09041	1.13E-09	6.79E-09
LINC00343	19.88371	-2.0544025	0.451328	-4.5519	5.32E-06	2.40E-05
AL161431.	378.0779	2.680340567	0.186652	14.3601	9.21E-47	1.74E-45
5_8S_rRNA	4093.475	-6.984247148	0.364355	-19.1688	6.74E-82	2.17E-80
NDRG2	298.0191	-2.058139413	0.231164	-8.90337	5.42E-19	4.97E-18
AC244502.	19.0837	-2.0079881	0.451206	-4.45027	8.58E-06	3.77E-05
TRAC	151.1267	2.074037159	0.256812	8.076082	6.69E-16	5.49E-15
SLC7A8	25279.11	-2.380524669	0.041618	-57.1997	0	0
MYH7	19.64651	-2.04203076	0.451305	-4.52473	6.05E-06	2.71E-05
CARMIL3	113.5309	-2.438271473	0.326727	-7.46271	8.48E-14	6.31E-13
TGM1	394.75	2.896407299	0.176066	16.45069	8.29E-61	2.01E-59
AKAP6	410.7147	-2.394209494	0.19888	-12.0385	2.23E-33	3.13E-32
LRFN5	227.4738	-2.926363727	0.263879	-11.0898	1.41E-28	1.74E-27
LINC02302	212.1254	-2.854575252	0.280018	-10.1943	2.10E-24	2.31E-23
TRIM9	168.1758	-3.069601136	0.302656	-10.1422	3.59E-24	3.91E-23
GNG2	297.6089	-2.100903149	0.215547	-9.74683	1.90E-22	1.97E-21
LINC00520	85.45199	-2.633146388	0.367101	-7.17281	7.35E-13	5.22E-12
OTX2	7889.034	-2.349676355	0.051012	-46.0613	0	0
SYNE2	939.3579	-2.062084988	0.134683	-15.3107	6.49E-53	1.37E-51
ESR2	20.43466	-2.091195583	0.451369	-4.633	3.60E-06	1.65E-05
PLEKHH1	1528.871	-2.567913189	0.110328	-23.2753	7.89E-120	3.82E-118
ARG2	98.63359	-2.101357458	0.336913	-6.2371	4.46E-10	2.75E-09
PGF	375.4377	-2.027747018	0.196082	-10.3413	4.58E-25	5.13E-24
TMEM63C	1065.281	-2.31302775	0.122753	-18.8429	3.36E-79	1.05E-77
TSHR	36.4984	-2.833520471	0.443519	-6.38873	1.67E-10	1.06E-09
SLC24A4	29.32907	-2.548890589	0.448102	-5.68819	1.28E-08	7.22E-08
CYP46A1	255.098	-2.116072324	0.23639	-8.95161	3.50E-19	3.24E-18
TMEM179	32.10659	-2.245218787	0.440781	-5.09372	3.51E-07	1.76E-06
PWRN1	44.84704	-2.243675633	0.41872	-5.35841	8.40E-08	4.45E-07
ATP10A	409.0376	3.160928584	0.174114	18.15434	1.19E-73	3.46E-72
GABRA5	1041.426	3.251607	0.120292	27.03095	6.40E-161	4.15E-159

GABRG3	63.69701	-2.376924269	0.388622	-6.1163	9.58E-10	5.80E-09
AC009562.	40.83563	-2.239820237	0.425589	-5.26287	1.42E-07	7.35E-07
TRPM1	2281.488	-4.121231405	0.262211	-15.7173	1.15E-55	2.55E-54
CHRNA7	75.19013	-2.471178502	0.373735	-6.61212	3.79E-11	2.48E-10
ACTC1	375.4422	3.915812243	0.195904	19.98843	6.94E-89	2.42E-87
LINC02694	25.70587	-2.35134666	0.450371	-5.22091	1.78E-07	9.14E-07
THBS1	4787.404	3.036645498	0.057461	52.84736	0	0
PLA2G4D	25.4336	-2.069459622	0.448565	-4.61351	3.96E-06	1.81E-05
PATL2	42.17662	-2.19338143	0.422289	-5.19403	2.06E-07	1.05E-06
SEMA6D	672.0561	-3.568042095	0.182217	-19.5813	2.23E-85	7.44E-84
SLC24A5	172.5574	-2.99427196	0.29963	-9.99323	1.63E-23	1.75E-22
SLC12A1	25.78725	-2.093447376	0.448186	-4.67094	3.00E-06	1.39E-05
CYP19A1	20.92523	-2.120571171	0.451362	-4.69816	2.63E-06	1.22E-05
AC023906.	69.04509	-2.469192224	0.383602	-6.43686	1.22E-10	7.76E-10
AC103740.	38.36826	2.229868475	0.408812	5.454511	4.91E-08	2.65E-07
TPM1	127933.8	2.732357856	0.024733	110.4753	0	0
TPM1-AS	74.37395	2.962700365	0.360108	8.227257	1.92E-16	1.61E-15
IGDCC4	297.1148	-3.834922191	0.271236	-14.1387	2.19E-45	4.04E-44
PAQR5	405.0622	-2.575326698	0.198585	-12.9684	1.85E-38	2.91E-37
AC027237.	292.4895	3.337260008	0.210869	15.8262	2.05E-56	4.61E-55
DRAIC	125.2898	-3.2777543	0.347635	-9.42873	4.15E-21	4.11E-20
UACA	4381.824	2.003852083	0.058584	34.20455	2.07E-256	2.25E-254
CCDC33	166.5451	-2.792193902	0.296688	-9.41121	4.90E-21	4.84E-20
SEMA7A	2327.943	3.015785544	0.077728	38.79926	0	0
CHRNB4	185.9842	-2.622126017	0.285921	-9.1708	4.70E-20	4.48E-19
RASGRF1	243.8932	2.646482726	0.212846	12.43376	1.71E-35	2.52E-34
IL16	30.82641	-2.054057787	0.440219	-4.66599	3.07E-06	1.42E-05
AP3B2	29.04766	-2.537682509	0.448239	-5.66145	1.50E-08	8.40E-08
HOMER2	3361.974	-2.768201418	0.077631	-35.6587	1.73E-278	2.08E-276
NMB	2883.689	-2.794872062	0.081966	-34.0981	7.88E-255	8.43E-253
AGBL1	40.49607	-2.088913987	0.424453	-4.92142	8.59E-07	4.18E-06
NTRK3	102.7441	-2.097447482	0.328861	-6.37792	1.80E-10	1.13E-09
PDIA2	393.399	4.205651798	0.193179	21.77079	4.39E-105	1.82E-103
CEROX1	2689.887	-3.373842407	0.094134	-35.841	2.54E-281	3.14E-279
SOX8	4014.858	-3.452176517	0.078942	-43.7305	0	0
AC004233.	15.14603	2.057921222	0.450927	4.563761	5.02E-06	2.27E-05
AC004233.	106.549	2.586800715	0.306549	8.438455	3.22E-17	2.77E-16
CLDN9	47.75236	2.676495791	0.394952	6.776755	1.23E-11	8.25E-11
CLDN6	105.4106	2.369301838	0.304629	7.777655	7.39E-15	5.77E-14
TNFRSF12	11563.58	4.08441051	0.042191	96.80705	0	0
UBALD1	3789.687	2.343869742	0.068114	34.41121	1.71E-259	1.88E-257
RBFOX1	41.24774	-2.988293925	0.440502	-6.78384	1.17E-11	7.87E-11
CIITA	300.3099	-2.653361715	0.230162	-11.5283	9.50E-31	1.24E-29
AC099489.	84.20416	-2.09490145	0.355452	-5.89363	3.78E-09	2.20E-08
MIR193BH	133.7011	2.054190042	0.274883	7.472974	7.84E-14	5.84E-13
MYH11	175.376	-3.578144914	0.318857	-11.2218	3.19E-29	4.00E-28
ABCC6P1	49.86802	-2.170987368	0.409848	-5.29706	1.18E-07	6.14E-07

CLEC19A	286.3994	-2.636830785	0.232178	-11.3569	6.85E-30	8.76E-29
ACSM2A	20.01255	-2.06575195	0.451349	-4.57684	4.72E-06	2.14E-05
ACSM2B	21.9953	-2.132740631	0.451365	-4.72509	2.30E-06	1.08E-05
DNAH3	74.05899	-3.178046745	0.403895	-7.86849	3.59E-15	2.85E-14
OTOA	19.16403	-2.013184035	0.451223	-4.46162	8.13E-06	3.59E-05
AC008938.	20.15325	-2.07486941	0.451359	-4.59694	4.29E-06	1.95E-05
SLC5A11	38.70976	-2.260707943	0.430159	-5.25552	1.48E-07	7.64E-07
AC106782.	122.8697	-2.48092338	0.360189	-6.88784	5.66E-12	3.87E-11
ABCC12	28.77434	-2.524195108	0.448416	-5.62914	1.81E-08	1.01E-07
AC007614.	39.53022	-2.940608559	0.441417	-6.66175	2.71E-11	1.79E-10
NKD1	43.4718	-2.745355658	0.433032	-6.33984	2.30E-10	1.44E-09
AC087564.	21.47995	-2.141257841	0.451345	-4.74417	2.09E-06	9.84E-06
TOX3	50.14699	-2.751654855	0.422926	-6.50624	7.71E-11	4.96E-10
MT2A	10908.07	2.558798009	0.04316	59.28694	0	0
MT1F	138.588	-2.62821911	0.311713	-8.43155	3.41E-17	2.94E-16
ADGRG5	200.464	-3.892091901	0.323928	-12.0153	2.95E-33	4.12E-32
KIFC3	5914.482	2.139181287	0.056546	37.83064	0	0
CNGB1	19.52198	-2.035737381	0.451292	-4.51091	6.46E-06	2.88E-05
CDH8	465.6937	2.119362042	0.157163	13.48515	1.91E-41	3.22E-40
PLEKHG4	640.3152	-2.022436014	0.15519	-13.032	8.05E-39	1.28E-37
KCTD19	46.46804	-2.626711604	0.424213	-6.19196	5.94E-10	3.63E-09
SMPD3	2450.599	-2.048367709	0.082115	-24.9453	2.40E-137	1.35E-135
MARVELD3	131.7381	-2.02720263	0.299325	-6.77259	1.26E-11	8.48E-11
LINC01568	26.49831	-2.418150567	0.449644	-5.37792	7.54E-08	4.01E-07
CHST6	123.592	-2.640894385	0.335155	-7.87963	3.28E-15	2.61E-14
PKD1L2	857.7194	-3.189040099	0.152111	-20.9653	1.36E-97	5.21E-96
AC009063.	25.11932	-2.34508974	0.450326	-5.20754	1.91E-07	9.80E-07
FOXC2	2042.804	2.623892703	0.095804	27.38807	3.80E-165	2.54E-163
SLC22A31	90.23183	-2.174492937	0.346872	-6.26887	3.64E-10	2.25E-09
SERPINF1	13398.04	-2.312676579	0.043188	-53.5491	0	0
ALOX12P2	20.91714	-2.118586346	0.451364	-4.69374	2.68E-06	1.25E-05
SLC2A4	105.7791	-3.369794819	0.374369	-9.00127	2.23E-19	2.07E-18
DNAH2	99.08896	-2.390431079	0.352705	-6.77742	1.22E-11	8.22E-11
DNAH9	59.71865	-3.342049406	0.424226	-7.87799	3.33E-15	2.64E-14
AC005224.	205.133	2.242470011	0.234582	9.559414	1.18E-21	1.20E-20
RASD1	165.4343	-3.432186324	0.327554	-10.4782	1.09E-25	1.24E-24
CCDC144N	144.5978	2.014188628	0.265416	7.588807	3.23E-14	2.45E-13
AC007923.	31.85899	-2.373819939	0.443203	-5.35606	8.51E-08	4.50E-07
RHBDL3	131.0436	-3.74002513	0.360227	-10.3824	2.98E-25	3.35E-24
CCL2	4820.907	3.119563812	0.065331	47.74991	0	0
PLXDC1	261.0085	-3.904437729	0.290816	-13.4258	4.27E-41	7.14E-40
PPP1R1B	37.54852	-2.850927253	0.443351	-6.4304	1.27E-10	8.09E-10
CCR7	57.70254	-2.575857916	0.407502	-6.32109	2.60E-10	1.63E-09
KRTAP2-3	186.7219	4.977228342	0.293276	16.97113	1.34E-64	3.43E-63
KRT32	129.2509	-2.09171518	0.325096	-6.43415	1.24E-10	7.89E-10
KRT36	25.07133	-2.309975423	0.450679	-5.12554	2.97E-07	1.50E-06
CAVIN1	48833.92	2.815032031	0.031689	88.83238	0	0

CNTNAP1	1047.492	-2.238898895	0.13117	-17.0687	2.54E-65	6.58E-64
RUNDC3A	515.925	-2.581532288	0.179484	-14.3831	6.61E-47	1.25E-45
AC005180.	305.93	2.06146643	0.20544	10.03439	1.08E-23	1.16E-22
AC005180.	151.6958	2.025606269	0.258441	7.837793	4.59E-15	3.61E-14
GFAP	45.76453	-2.608265632	0.424613	-6.14269	8.11E-10	4.93E-09
TTLL6	20.80448	-2.106486187	0.45137	-4.66687	3.06E-06	1.41E-05
B4GALNT2	22.83952	-2.213815493	0.45115	-4.90705	9.25E-07	4.49E-06
COL1A1	108468	3.593737775	0.028418	126.4587	0	0
HLF	42.38145	-3.03726913	0.439323	-6.91353	4.73E-12	3.24E-11
ANKFN1	22.36512	-2.176151243	0.451284	-4.82213	1.42E-06	6.80E-06
SRSF1	1636.689	2.035013108	0.089628	22.70503	4.00E-114	1.85E-112
SEPTIN4	2843.568	-2.377251864	0.082725	-28.7367	1.33E-181	9.73E-180
SCN4A	21.00986	-2.120034769	0.451363	-4.69696	2.64E-06	1.23E-05
SMURF2	4368.618	2.536982372	0.058706	43.21503	0	0
SLC16A6	119.498	-2.461983086	0.324003	-7.59865	2.99E-14	2.27E-13
MAP2K6	245.2764	-2.226327315	0.24226	-9.18982	3.93E-20	3.77E-19
SOX9-AS1	67.10036	-2.254026336	0.383139	-5.88305	4.03E-09	2.34E-08
RAB37	736.7694	-2.073722161	0.145917	-14.2117	7.76E-46	1.44E-44
UBALD2	1120.284	2.820030288	0.129939	21.70277	1.93E-104	7.96E-103
CYGB	510.5426	-2.381634732	0.180281	-13.2107	7.61E-40	1.23E-38
MGAT5B	1657.642	-2.145762308	0.098674	-21.746	7.55E-105	3.13E-103
AC144831.	187.6726	2.266693436	0.235171	9.638479	5.50E-22	5.60E-21
COLEC12	4537.208	-2.294884806	0.063524	-36.126	8.86E-286	1.11E-283
LINC00470	26.66139	-2.275150745	0.448556	-5.07216	3.93E-07	1.97E-06
EMILIN2	317.0861	-2.38685238	0.220947	-10.8028	3.34E-27	3.97E-26
DLGAP1	89.29858	-3.752583381	0.404594	-9.27494	1.78E-20	1.72E-19
MIR3976H	43.14156	-3.054746036	0.439014	-6.9582	3.45E-12	2.38E-11
TMEM200C	106.6153	-2.414581376	0.336639	-7.17262	7.36E-13	5.22E-12
ARHGAP28	385.391	-4.224134983	0.261102	-16.1781	7.20E-59	1.70E-57
KRT18P8	76.36647	2.69988908	0.340374	7.93212	2.15E-15	1.73E-14
APCDD1	328.6501	-3.364685372	0.42421	-7.93165	2.16E-15	1.73E-14
IMPA2	3524.453	-2.111745844	0.069081	-30.5692	3.15E-205	2.64E-203
TUBB6	24509.76	2.447663937	0.031518	77.65995	0	0
LINC01443	74.51806	-3.542598969	0.411637	-8.60612	7.56E-18	6.68E-17
LINC01915	19.05136	-2.002041475	0.451175	-4.4374	9.11E-06	3.99E-05
AC018697.	21.52846	-2.155661713	0.45131	-4.77645	1.78E-06	8.45E-06
TTR	36.07198	-2.802884089	0.444239	-6.30941	2.80E-10	1.75E-09
GAREM1	354.5913	-3.024001044	0.221472	-13.6541	1.91E-42	3.29E-41
KLHL14	51.20522	-2.131166805	0.407276	-5.23273	1.67E-07	8.60E-07
AC068506.	67.69674	-2.624565659	0.391954	-6.69611	2.14E-11	1.42E-10
LINC00907	35.19355	-2.233245273	0.435644	-5.12631	2.95E-07	1.49E-06
SLC14A1	21.10635	-2.126274868	0.451358	-4.71084	2.47E-06	1.15E-05
LOXHD1	32.13934	-2.632394407	0.447189	-5.88654	3.94E-09	2.30E-08
ST8SIA5	32.16981	-2.105066711	0.439086	-4.7942	1.63E-06	7.77E-06
ZBTB7C	96.935	-2.873087047	0.359938	-7.98217	1.44E-15	1.16E-14
SMAD7	2171.886	3.105007252	0.081163	38.25634	0	0
MRO	47.71031	-2.68145497	0.425109	-6.30769	2.83E-10	1.77E-09

LINC01630	43.52755	-2.786025404	0.433743	-6.42321	1.33E-10	8.47E-10
DCC	63.20265	-2.468716822	0.398279	-6.19846	5.70E-10	3.49E-09
RAB27B	1247.912	-3.178352878	0.129392	-24.5637	3.09E-133	1.69E-131
CCDC68	416.2921	-2.286541528	0.203068	-11.26	2.07E-29	2.61E-28
ATP8B1	1221.424	2.062180339	0.10244	20.1307	3.97E-90	1.40E-88
ALPK2	514.3479	3.299739233	0.188531	17.50238	1.37E-68	3.74E-67
PMAIP1	2011.783	-2.203360304	0.090525	-24.3398	7.43E-131	3.99E-129
CDH7	56.01641	-3.022618501	0.427699	-7.06716	1.58E-12	1.11E-11
NETO1	50.42303	-3.267003145	0.434167	-7.52476	5.28E-14	3.97E-13
CHGB	214.3288	-2.166076225	0.253013	-8.56113	1.12E-17	9.82E-17
PLCB4	3802.031	2.539515239	0.069316	36.63689	7.40E-294	9.64E-292
SNAP25	1003.057	-2.210215049	0.12444	-17.7614	1.41E-70	3.92E-69
JAG1	5804.098	-3.226083941	0.063592	-50.7307	0	0
SPTLC3	539.2291	-2.112225178	0.192181	-10.9908	4.23E-28	5.16E-27
AL118508.	23.83682	-2.25452261	0.450988	-4.99908	5.76E-07	2.85E-06
LINC01721	21.38346	-2.136408987	0.451351	-4.73337	2.21E-06	1.04E-05
FOXS1	83.69221	2.78726037	0.351091	7.93886	2.04E-15	1.64E-14
MMP24	6929.722	2.350791194	0.048566	48.40366	0	0
TGM2	44766.34	5.177975514	0.031297	165.4473	0	0
MAFB	154.3681	-2.854436836	0.305358	-9.34783	8.95E-21	8.76E-20
PTPRT	29.8358	-2.572741943	0.447762	-5.74579	9.15E-09	5.20E-08
TOX2	5210.434	-2.012820846	0.06524	-30.8526	5.16E-209	4.42E-207
JPH2	1660.271	2.835836726	0.090078	31.48212	1.53E-217	1.36E-215
CCN5	103.0171	-2.701152459	0.346092	-7.80472	5.96E-15	4.67E-14
KCNS1	293.3338	-2.113683627	0.217031	-9.73907	2.05E-22	2.12E-21
LINC00494	59.98694	-2.994298266	0.415549	-7.20564	5.78E-13	4.12E-12
PREX1	1800.478	-2.020913172	0.093103	-21.7063	1.79E-104	7.39E-103
KCNB1	42.82647	-2.124715574	0.419971	-5.0592	4.21E-07	2.10E-06
PELATON	88.93417	-2.606805771	0.359647	-7.24823	4.22E-13	3.03E-12
LINC01524	43.60202	-2.419828992	0.431156	-5.61242	2.00E-08	1.11E-07
TSHZ2	719.556	-3.258608499	0.167782	-19.4217	5.06E-84	1.66E-82
BMP7	34.88464	-2.010743642	0.433035	-4.64338	3.43E-06	1.58E-05
PMEPA1	3841.531	2.109309718	0.059468	35.46946	1.45E-275	1.73E-273
FLJ16779	263.4446	2.168901903	0.435543	4.979769	6.37E-07	3.13E-06
FSTL3	31899.36	2.818140578	0.03613	77.99913	0	0
LINC01836	129.8289	2.518451872	0.291252	8.646986	5.29E-18	4.70E-17
GADD45B	4641.691	3.720073464	0.061051	60.93358	0	0
TJP3	127.9759	-2.21140229	0.306725	-7.20972	5.61E-13	4.00E-12
MUC16	177.8606	-4.969350792	0.388835	-12.7801	2.12E-37	3.26E-36
C3P1	29.84621	-2.394994963	0.446214	-5.36736	7.99E-08	4.24E-07
ICAM5	388.7659	-2.474064514	0.200613	-12.3325	6.05E-35	8.76E-34
AP1M2	1158.868	4.18874086	0.117218	35.73454	1.15E-279	1.40E-277
RGL3	54.28967	-2.209415504	0.399951	-5.52422	3.31E-08	1.81E-07
CNN1	6065.352	2.671494171	0.050126	53.29561	0	0
CACNA1A	127.5011	-3.004539456	0.334413	-8.98451	2.60E-19	2.41E-18
ADGRE2	23.1813	-2.233135089	0.451067	-4.95079	7.39E-07	3.61E-06
OR7C1	20.92092	-2.0928528	0.451364	-4.63673	3.54E-06	1.62E-05

KLF2	203.9113	2.147683918	0.230817	9.304713	1.34E-20	1.31E-19
UNC13A	52.56509	-2.592302539	0.414256	-6.25774	3.91E-10	2.41E-09
RAB3A	877.546	-2.744146529	0.14245	-19.2639	1.08E-82	3.50E-81
LRRC25	60.62524	-3.259791691	0.41995	-7.76233	8.34E-15	6.50E-14
AC003973.	19.30095	-2.014337309	0.451221	-4.46419	8.04E-06	3.55E-05
AC139769.	196.6301	-3.152282204	0.29211	-10.7914	3.78E-27	4.49E-26
LINC01837	21.86594	-2.160278438	0.451315	-4.78663	1.70E-06	8.05E-06
RGS9BP	149.1458	-3.299779149	0.335765	-9.82765	8.56E-23	8.96E-22
GARRE1	1553.537	2.004026293	0.101117	19.81893	2.04E-87	6.95E-86
WTIP	3211.687	3.464864782	0.07062	49.06361	0	0
AD000090.	29.86384	-2.57191046	0.447793	-5.74353	9.27E-09	5.27E-08
KIRREL2	176.3417	-2.871668497	0.291995	-9.83464	7.99E-23	8.37E-22
WDR87	32.18407	-2.672224614	0.446269	-5.98792	2.13E-09	1.26E-08
ACP7	25.81183	-2.023121156	0.448294	-4.51293	6.39E-06	2.86E-05
FCGBP	135.9631	-2.392980651	0.317365	-7.54016	4.69E-14	3.54E-13
PRX	582.9647	-2.074328052	0.161493	-12.8447	9.21E-38	1.43E-36
CYP2B7P	19.13977	-2.003174562	0.451172	-4.43994	9.00E-06	3.95E-05
CYP2B6	19.42118	-2.013322525	0.4512	-4.46215	8.11E-06	3.58E-05
PCAT19	22.76297	-2.222840928	0.451093	-4.92768	8.32E-07	4.05E-06
CNFN	104.8542	-2.185473158	0.348679	-6.26787	3.66E-10	2.27E-09
LIPE	2720.184	-2.916003203	0.087625	-33.2781	8.00E-243	8.06E-241
LYPD3	145.9909	-2.081198723	0.299108	-6.95801	3.45E-12	2.38E-11
GIPR	1425.494	-2.368830543	0.107583	-22.0186	1.91E-107	8.25E-106
IGFL2	160.7569	-2.669695788	0.294899	-9.05291	1.39E-19	1.30E-18
HIF3A	315.1559	-2.697087081	0.225842	-11.9424	7.12E-33	9.82E-32
DACT3	746.0365	-2.652253481	0.151695	-17.4841	1.89E-68	5.14E-67
CCDC114	153.0248	-2.002259251	0.286773	-6.98204	2.91E-12	2.01E-11
SLC17A7	33.69191	-2.39001993	0.443935	-5.38372	7.30E-08	3.88E-07
CD33	21.63683	-2.155681422	0.451317	-4.77642	1.78E-06	8.45E-06
VSIG10L	624.3142	-2.015463534	0.155704	-12.9442	2.53E-38	3.98E-37
HAS1	2004.901	-2.505606065	0.098314	-25.4857	2.84E-143	1.66E-141
CACNG7	81.38761	-2.118926175	0.359752	-5.88996	3.86E-09	2.25E-08
COX6B2	258.9625	-2.72251979	0.253922	-10.7219	8.04E-27	9.48E-26
ZSCAN1	215.119	-2.20261826	0.249681	-8.82172	1.13E-18	1.02E-17
LINC00279	23.69938	-2.244144373	0.451059	-4.97528	6.52E-07	3.20E-06
RTN4R	349.0507	2.988701133	0.189101	15.80483	2.88E-56	6.45E-55
ZDHHC8P1	1798.511	2.512094359	0.094368	26.62025	3.96E-156	2.50E-154
AP000344.	380.9536	2.260431709	0.184719	12.23712	1.97E-34	2.82E-33
GGT1	47.17964	-2.642810681	0.423966	-6.23355	4.56E-10	2.81E-09
SGSM1	19.1802	-2.014272971	0.451228	-4.46398	8.05E-06	3.55E-05
MYO18B	34.5626	-2.0897475	0.439799	-4.7516	2.02E-06	9.50E-06
LINC01638	500.8154	2.49579759	0.166151	15.02125	5.33E-51	1.08E-49
KREMEN1	2574.451	-2.500527941	0.083659	-29.8896	2.68E-196	2.12E-194
CABP7	252.4431	-2.148265988	0.237983	-9.02699	1.76E-19	1.64E-18
HORMAD2-	44.82487	-2.190736895	0.41761	-5.2459	1.56E-07	8.03E-07
HORMAD2	57.74091	-2.044386526	0.398321	-5.13251	2.86E-07	1.45E-06
LIF	17308.99	2.220564372	0.037803	58.73983	0	0

AC004264.	1206.933	3.025631192	0.110572	27.36352	7.46E-165	4.97E-163
AC005005.	33.40379	-2.160555451	0.437681	-4.93636	7.96E-07	3.88E-06
SLC5A1	23.39855	-2.228768132	0.451112	-4.94061	7.79E-07	3.80E-06
AL021877.	24.72095	-2.325179683	0.45049	-5.16144	2.45E-07	1.25E-06
Z82217.1	21.70529	-2.165528351	0.451289	-4.79854	1.60E-06	7.61E-06
Z95114.1	32.75497	-2.69445541	0.445922	-6.04244	1.52E-09	9.08E-09
MAFF	579.5647	2.107305612	0.140822	14.9643	1.26E-50	2.54E-49
NPTXR	6409.76	-2.260419299	0.060898	-37.1179	1.44E-301	1.94E-299
PDGFB	432.6258	4.061446562	0.18638	21.79126	2.81E-105	1.17E-103
FAM83F	29.90565	-2.014284933	0.441114	-4.56636	4.96E-06	2.25E-05
SCUBE1	309.2671	-3.264769388	0.24588	-13.2779	3.11E-40	5.07E-39
AL031595.	26.42985	-2.410051774	0.449751	-5.35864	8.39E-08	4.44E-07
AL117329.	75.61975	-3.535591017	0.41033	-8.61646	6.91E-18	6.12E-17
TAFA5	397.4501	2.547938931	0.171009	14.89941	3.32E-50	6.66E-49
AC207130.	86.46744	2.227158476	0.324657	6.860027	6.88E-12	4.68E-11
MOV10L1	452.5341	2.517797539	0.162166	15.52606	2.31E-54	4.96E-53
KCNE1B	35.93679	2.258999147	0.442449	5.105673	3.30E-07	1.66E-06
FP671120.	383.4046	-5.959925392	0.365298	-16.3152	7.69E-60	1.84E-58
RNA5-8SN	64.00967	-3.575889746	0.426723	-8.37989	5.30E-17	4.54E-16
FP236383.	696.6159	-6.433012493	0.33198	-19.3777	1.19E-83	3.89E-82
RNA5-8SN	320.2381	-4.857454047	0.319471	-15.2047	3.29E-52	6.86E-51
FP236383.	9577.589	-7.80266137	0.157645	-49.4952	0	0
RNA5-8SN	150.2469	-4.665470868	0.399098	-11.69	1.43E-31	1.92E-30
AJ009632.	37.28487	-2.0927369	0.434798	-4.81312	1.49E-06	7.10E-06
MIR99AHG	86.02711	-2.319259086	0.358835	-6.46331	1.02E-10	6.56E-10
LINC00320	23.6999	-2.271255629	0.450847	-5.03776	4.71E-07	2.34E-06
CYYR1	134.9776	-2.830062701	0.3232	-8.75638	2.02E-18	1.81E-17
MAP3K7CL	556.8301	3.666916511	0.1788	20.50845	1.81E-93	6.62E-92
AP000331.	63.28212	-2.758409726	0.407757	-6.76484	1.33E-11	8.94E-11
AP000695.	30.43803	2.289492675	0.429525	5.330294	9.81E-08	5.15E-07
KCNJ6	27.1215	-2.445640147	0.449369	-5.44239	5.26E-08	2.83E-07
KCNJ15	27.75813	-2.131136832	0.448232	-4.75453	1.99E-06	9.37E-06
ETS2	2610.158	2.237883365	0.07088	31.57269	8.76E-219	7.88E-217
AP001042.	30.73609	-2.583467123	0.447817	-5.76903	7.97E-09	4.55E-08
B3GALT5	702.5987	-2.58839442	0.161474	-16.0298	7.92E-58	1.83E-56
B3GALT5-A	1127.824	-2.201597077	0.120465	-18.2758	1.29E-74	3.78E-73
IGSF5	172.5706	-3.151297592	0.307599	-10.2448	1.25E-24	1.38E-23
PCP4	5170.409	-5.838832081	0.11801	-49.4775	0	0
PLAC4	20.97374	-2.111112031	0.451369	-4.67713	2.91E-06	1.35E-05
TRPM2	167.1734	2.507242227	0.264873	9.465836	2.91E-21	2.90E-20
PCBP3	718.877	-2.431431771	0.150529	-16.1526	1.09E-58	2.55E-57
RNA5-8SN	99.83415	-4.17833686	0.410524	-10.178	2.48E-24	2.72E-23
