# Supplementary material for: Global Transcriptional and Epigenetic Reconfiguration during Chemical Reprogramming of Human Retinal Pigment Epithelial Cells into Photoreceptor-like Cells
Source: Cells. 2022 Oct 6;11(19):3146. doi: 10.3390/cells11193146 (PMC9564162; doi:10.3390/cells11193146)
Supplement: Supplementary file 1 [file cells-11-03146-s001.zip › Supplementary Table S2.pdf]

Supplementary Materials

Supplementary Table S2:

Differently expressed genes (DEGs) in D10 CiPCs compared with RPE control

Gene	Base mean	Log2 fold change	lfcSE	Stat	p value	p adjust
MTND1P23	4529.22838	7.723677183	0.989378	7.806599	5.88E-15	2.57E-13
MTND2P28	1377.03474	-2.522173006	0.374233	-6.73957	1.59E-11	4.61E-10
MTCO1P12	1072.23262	2.117056064	0.478171	4.427401	9.54E-06	9.14E-05
LINC02593	138.372043	-2.127980552	0.503836	-4.22356	2.40E-05	0.000206
PLEKHN1	79.6259526	-2.22826711	0.692509	-3.21767	0.001292	0.005994
ISG15	534.365446	-2.259967517	0.369796	-6.11138	9.88E-10	2.18E-08
AGRN	36557.4292	-3.487657407	0.300859	-11.5923	4.51E-31	6.76E-29
MXRA8	5870.38654	-2.281345379	0.319291	-7.14503	9.00E-13	3.08E-11
FNDC10	497.793698	-2.834038324	0.392067	-7.22845	4.89E-13	1.74E-11
AL031282.1	204.439298	-2.78635777	0.465817	-5.98166	2.21E-09	4.62E-08
CFAP74	48.6540583	4.580875296	0.912528	5.019982	5.17E-07	6.87E-06
GABRD	41.524992	6.525557934	1.088948	5.992534	2.07E-09	4.34E-08
SKI	2751.59897	-2.018093387	0.334397	-6.03503	1.59E-09	3.41E-08
HES5	10.7539184	4.178531017	1.264949	3.303319	0.000955	0.004683
AL139246.5	423.879453	4.241683265	0.42587	9.960031	2.28E-23	2.18E-21
TNFRSF14-AS1	48.8315061	3.436043776	0.746793	4.601068	4.20E-06	4.42E-05
TNFRSF14	3365.36797	2.900930089	0.396461	7.317056	2.53E-13	9.34E-12
AL139246.2	10.8564181	4.301121925	1.254435	3.428732	0.000606	0.003213
AL139246.3	20.9338129	5.411777726	1.174482	4.607801	4.07E-06	4.29E-05
PRDM16-DT	13343.3696	4.849560752	0.329276	14.72795	4.26E-49	1.95E-46
PRDM16	2250.12503	2.002905034	0.322865	6.203531	5.52E-10	1.27E-08
MEGF6	6204.24084	-4.233976697	0.325551	-13.0056	1.14E-38	2.81E-36
TPRG1L	2257.82978	-2.661406361	0.324787	-8.19431	2.52E-16	1.31E-14
TP73	19.2590566	3.989330917	1.094537	3.644766	0.000268	0.001632
CCDC27	20.2924556	5.289412508	1.18594	4.4601	8.19E-06	7.99E-05
LINC01346	15.0707139	4.794131131	1.222066	3.922974	8.75E-05	0.000633
AL805961.1	14.2829148	4.592011422	1.239841	3.703711	0.000212	0.001343
LINC01777	11.3747884	4.154235208	1.269217	3.273069	0.001064	0.00509
AL355602.1	15.808731	4.534756087	1.250212	3.627189	0.000287	0.001728
LINC01646	11.193212	4.276943502	1.257921	3.400009	0.000674	0.003502
AJAP1	454.632393	2.609881623	0.420247	6.210344	5.29E-10	1.22E-08
Z98886.1	21.8299834	5.452601671	1.171696	4.653596	3.26E-06	3.52E-05
TNFRSF25	735.031162	-2.679709243	0.38871	-6.89385	5.43E-12	1.67E-10
PLEKHG5	1539.41106	-3.107513504	0.336004	-9.24845	2.28E-20	1.73E-18
TAS1R1	14.5025617	4.638184454	1.236066	3.752377	0.000175	0.001146
DNAJC11	1149.57215	-2.10701448	0.333758	-6.313	2.74E-10	6.62E-09
LINC01672	10.405413	3.972768242	1.280392	3.102774	0.001917	0.00826

PER3	738.06587	2.918174168	0.361413	8.074353	6.78E-16	3.40E-14
Y_RNA	539.68757	2.463526501	0.529354	4.653831	3.26E-06	3.52E-05
ERRFI1	700.235808	-3.023056859	0.376863	-8.02163	1.04E-15	5.10E-14
ENO1	45487.1368	-2.825261005	0.397951	-7.09951	1.25E-12	4.21E-11
SLC2A7	10.6250598	4.049542664	1.275176	3.175673	0.001495	0.006735
PIK3CD-AS2	253.628792	2.266139785	0.454381	4.987312	6.12E-07	7.99E-06
CTNNBIP1	606.710991	-2.349203821	0.362302	-6.4841	8.93E-11	2.33E-09
ANGPTL7	2846.9683	-6.061056872	0.340542	-17.7983	7.29E-71	7.26E-68
DISP3	25.034423	3.941590094	1.048008	3.761031	0.000169	0.001112
FBXO2	2083.83708	-2.771508976	0.353266	-7.8454	4.32E-15	1.91E-13
C1orf167	13.7147626	4.229003715	1.270122	3.329606	0.00087	0.004326
PLOD1	7633.12747	-2.863373185	0.318521	-8.98958	2.48E-19	1.73E-17
AADACL3	11.7877232	4.373743725	1.25132	3.495305	0.000474	0.002615
C1orf158	10.0041897	4.063829218	1.271846	3.195222	0.001397	0.006372
PRAMEF1	11.5036471	4.283732053	1.258499	3.403843	0.000664	0.003466
HNRNPCL1	9.47410791	3.993729441	1.275601	3.130862	0.001743	0.007649
TMEM51-AS1	25.1276214	5.499392116	1.177316	4.671125	3.00E-06	3.27E-05
TMEM51	593.563013	2.403318557	0.395441	6.077562	1.22E-09	2.65E-08
FHAD1	83.7525659	4.150676564	0.67533	6.146144	7.94E-10	1.79E-08
PLEKHM2	4110.36874	-2.833959881	0.310076	-9.13956	6.27E-20	4.67E-18
FBLIM1	7605.82275	-6.181769175	0.343582	-17.9921	2.25E-72	2.54E-69
AL450998.1	42.1664215	3.796598865	0.874836	4.339783	1.43E-05	0.000131
HSPB7	350.228467	-3.791777792	0.506263	-7.48973	6.90E-14	2.72E-12
CLCNKA	51.1108704	3.61384573	0.861302	4.195794	2.72E-05	0.000229
EPHA2	5750.20846	-4.989576598	0.316294	-15.7751	4.61E-56	3.07E-53
SZRD1	3634.82812	-2.390316145	0.31936	-7.48472	7.17E-14	2.82E-12
SPATA21	17.1002531	4.969940569	1.210807	4.10465	4.05E-05	0.000325
AL137798.1	10.9354948	3.987525477	1.280888	3.113095	0.001851	0.008019
ESPNP	19.2586508	5.181979812	1.194801	4.337106	1.44E-05	0.000132
ATP13A2	2675.6536	-2.372310535	0.321436	-7.38036	1.58E-13	5.97E-12
LINC02783	84.0684704	-2.753425833	0.755068	-3.64659	0.000266	0.001622
PADI4	11.7877232	4.373743725	1.25132	3.495305	0.000474	0.002615
RCC2	2324.95207	-2.164003227	0.320375	-6.7546	1.43E-11	4.20E-10
IGSF21	112.992221	7.929164856	1.004747	7.891703	2.98E-15	1.36E-13
PAX7	17.9849589	3.967854596	1.139796	3.481196	0.000499	0.002729
ALDH4A1	1387.19186	-2.432928765	0.346475	-7.02195	2.19E-12	7.08E-11
IFFO2	1077.79648	-4.123382949	0.485104	-8.5	1.90E-17	1.09E-15
EMC1	2939.45018	-2.245626863	0.312618	-7.1833	6.80E-13	2.37E-11
CAPZB	9554.6675	-2.046808719	0.356548	-5.74063	9.43E-09	1.76E-07
NBL1	1279.62349	-4.246922031	0.363713	-11.6766	1.68E-31	2.63E-29
HTR6	16.2103601	3.560730241	1.159972	3.069668	0.002143	0.009027
PLA2G2A	9.73182516	4.116361029	1.266495	3.250199	0.001153	0.005447
PLA2G5	37.520383	2.694132452	0.801503	3.361349	0.000776	0.003943
PLA2G2F	24.9521636	4.45896391	1.096934	4.064934	4.80E-05	0.000376

PLA2G2C	36.3413208	6.338944312	1.101261	5.756079	8.61E-09	1.62E-07
UBXN10	219.827188	4.978126454	0.502388	9.908929	3.81E-23	3.59E-21
VWA5B1	43.8814677	5.158549218	0.940588	5.48439	4.15E-08	6.91E-07
LINC01141	19.3117744	3.93202494	1.110305	3.54139	0.000398	0.00227
PINK1	683.372384	-2.152061029	0.358903	-5.99622	2.02E-09	4.26E-08
DDOST	7375.08238	-2.152146438	0.332089	-6.48064	9.13E-11	2.38E-09
SH2D5	347.711333	-2.159746608	0.399707	-5.40333	6.54E-08	1.04E-06
ECE1	3634.53563	-3.451555007	0.313057	-11.0253	2.88E-28	3.80E-26
ALPL	90.6456026	4.812166674	0.689527	6.978941	2.97E-12	9.46E-11
HSPG2	4825.91442	-3.907242977	0.417716	-9.35383	8.45E-21	6.65E-19
EPHA8	16.1149017	4.137559985	1.193229	3.467533	0.000525	0.002852
EPHB2	3354.75235	-3.417421664	0.313023	-10.9175	9.51E-28	1.21E-25
LUZP1	1327.6788	-2.208008318	0.340209	-6.49015	8.58E-11	2.25E-09
E2F2	83.6472674	2.079922627	0.62303	3.338398	0.000843	0.004213
ID3	2618.77231	-2.83032545	0.321028	-8.81645	1.18E-18	7.67E-17
CNR2	17.6566938	4.903090935	1.22011	4.018563	5.86E-05	0.000447
NIPAL3	2454.64143	-2.645574476	0.318833	-8.29768	1.06E-16	5.72E-15
NCMAP	10.4698423	4.061968818	1.273658	3.189215	0.001427	0.006475
RUNX3	21.7011247	5.413751	1.176036	4.603389	4.16E-06	4.38E-05
AL445471.1	9.0728847	3.978599865	1.275392	3.119511	0.001812	0.007878
MAN1C1	1088.39165	3.617697548	0.342695	10.55662	4.73E-26	5.28E-24
SELENON	2379.22642	-2.106281162	0.315518	-6.67564	2.46E-11	7.00E-10
SLC30A2	36.3793912	6.344790338	1.100655	5.764556	8.19E-09	1.55E-07
TRIM63	149.155673	6.358611688	0.716202	8.878244	6.79E-19	4.53E-17
ZPLD2P	46.7455409	3.885685675	0.797843	4.870238	1.11E-06	1.36E-05
SH3BGRL3	4395.67862	-4.021106103	0.433557	-9.27469	1.78E-20	1.38E-18
TENT5B	142.167934	-2.502016654	0.505177	-4.95276	7.32E-07	9.36E-06
FGR	12.938675	4.416877878	1.251663	3.528807	0.000417	0.002357
SESN2	1185.77651	-2.094977722	0.351306	-5.9634	2.47E-09	5.15E-08
Metazoa_SRP	238.177426	2.547074237	0.546636	4.659545	3.17E-06	3.44E-05
TMEM200B	219.532523	-2.257112136	0.495089	-4.559	5.14E-06	5.28E-05
FABP3	1235.21407	2.998421962	0.471499	6.359343	2.03E-10	5.01E-09
SERINC2	2651.00164	-2.855607791	0.416904	-6.84955	7.41E-12	2.23E-10
LINC01226	27.5032558	4.085983888	1.033123	3.954982	7.65E-05	0.000563
TINAGL1	714.297541	-3.88472003	0.411799	-9.43353	3.97E-21	3.22E-19
SPOCD1	2751.9751	-3.66911065	0.321139	-11.4253	3.13E-30	4.49E-28
CCDC28B	785.8032	2.398864307	0.461273	5.200532	1.99E-07	2.88E-06
BSDC1	1662.51552	-2.022815744	0.329866	-6.13224	8.66E-10	1.93E-08
ZBTB8B	32.8065565	3.394977536	0.911223	3.725739	0.000195	0.001254
SYNC	501.633714	-5.207562543	0.438427	-11.8778	1.54E-32	2.55E-30
YARS1	2225.19318	-2.061434299	0.330646	-6.23456	4.53E-10	1.06E-08
CSMD2-AS1	10.4434834	4.166374392	1.26483	3.29402	0.000988	0.004787
DLGAP3	74.1696809	4.143868764	0.714599	5.798873	6.68E-09	1.28E-07
NCDN	1692.35825	-2.071713076	0.324139	-6.39143	1.64E-10	4.11E-09

COL8A2	4703.15106	5.425141509	0.321271	16.88648	5.66E-64	4.46E-61
MAP7D1	9962.87757	-2.258911789	0.36681	-6.15827	7.35E-10	1.66E-08
SH3D21	578.959272	-2.977940026	0.39063	-7.62343	2.47E-14	1.02E-12
CSF3R	34.3440121	5.93062709	1.150437	5.155109	2.53E-07	3.61E-06
GRIK3	36.0545874	5.092621883	1.026134	4.96292	6.94E-07	8.93E-06
RSPO1	12.1625875	4.422982886	1.248134	3.543676	0.000395	0.002253
EPHA10	30.9370884	3.010637829	0.87009	3.460146	0.00054	0.002922
FHL3	974.671591	-3.378059994	0.368508	-9.16685	4.87E-20	3.68E-18
POU3F1	32.0883264	2.868851335	0.925521	3.099716	0.001937	0.008334
LINC01685	14.2363156	3.611303113	1.172747	3.079355	0.002074	0.008797
MACF1	5157.28524	-2.287712699	0.36662	-6.24002	4.38E-10	1.02E-08
HEYL	15.8204426	4.86709657	1.217154	3.998753	6.37E-05	0.00048
HPCAL4	19.4138683	5.181459929	1.195445	4.334337	1.46E-05	0.000133
BMP8B	65.6748535	3.554737415	0.678068	5.242449	1.58E-07	2.34E-06
CAP1	9756.47089	-3.004756803	0.313159	-9.59498	8.39E-22	7.22E-20
KCNQ4	307.304678	3.621950523	0.48981	7.39461	1.42E-13	5.41E-12
AC093151.7	9.72011361	3.945326647	1.280246	3.081694	0.002058	0.008746
EDN2	1253.94497	-4.787586004	0.445373	-10.7496	5.95E-27	7.08E-25
AL158216.1	8.94402608	3.914238868	1.279942	3.058139	0.002227	0.009318
CLDN19	564.924895	7.493646433	0.533026	14.05868	6.81E-45	2.33E-42
PTPRF	11411.7626	-2.027359391	0.325415	-6.23007	4.66E-10	1.09E-08
AL139220.2	54.1946942	2.64527925	0.733681	3.605489	0.000312	0.001856
PLK3	655.055115	-2.865886653	0.389606	-7.35586	1.90E-13	7.06E-12
BTBD19	1141.27413	-2.126799415	0.361863	-5.87737	4.17E-09	8.30E-08
ZSWIM5	125.918263	5.092833044	0.66	7.716419	1.20E-14	5.07E-13
PRDX1	5288.91976	-2.527843496	0.417098	-6.06054	1.36E-09	2.93E-08
FAAH	907.493047	3.647855773	0.410195	8.892988	5.95E-19	4.03E-17
FAAHP1	40.4190102	3.09222289	0.800011	3.865224	0.000111	0.000776
DMBX1	344.706754	4.84179818	0.494507	9.791155	1.23E-22	1.13E-20
CYP4Z1	14.4762028	4.720153283	1.227539	3.845216	0.00012	0.000832
CYP4A22	14.7222085	4.681425272	1.232515	3.798272	0.000146	0.000979
TAL1	16.8161769	4.89730968	1.217703	4.021761	5.78E-05	0.000442
AL135960.1	12.2533757	4.381831723	1.252369	3.498834	0.000467	0.002588
AL356289.2	606.52291	-2.054010885	0.548293	-3.7462	0.00018	0.001169
SPATA6	294.899122	2.381234544	0.417281	5.706551	1.15E-08	2.12E-07
AGBL4	27.3861087	4.122004567	1.016179	4.056378	4.98E-05	0.000388
ELAVL4	22.723218	5.376773655	1.184153	4.540609	5.61E-06	5.70E-05
LINC02808	15.4719371	4.755451117	1.227717	3.87341	0.000107	0.000753
CDKN2C	271.985848	3.301182933	0.469317	7.034011	2.01E-12	6.55E-11
GPX7	325.700509	2.68765837	0.457184	5.878725	4.13E-09	8.26E-08
SLC1A7	36.1894143	3.247429045	0.877077	3.702557	0.000213	0.001349
CPT2	875.28268	2.210162418	0.353318	6.255454	3.96E-10	9.33E-09
LRP8	789.272944	2.294943084	0.396948	5.781474	7.40E-09	1.41E-07
SLC25A3P1	12.7570987	4.511683948	1.241888	3.632924	0.00028	0.001694

FAM151A	36.0453913	2.791246758	0.811227	3.440771	0.00058	0.003093
MROH7	32.0423553	5.064374839	0.994381	5.092991	3.52E-07	4.87E-06
TTC22	21.9593675	3.612874564	1.115852	3.237772	0.001205	0.005634
BSND	29.799041	4.764043763	1.06309	4.481319	7.42E-06	7.30E-05
AL603840.1	29.8253998	4.711545775	1.07853	4.368487	1.25E-05	0.000116
LINC01755	10.7539184	4.178531017	1.264949	3.303319	0.000955	0.004683
AC119674.1	29.8837106	5.836174125	1.150014	5.074874	3.88E-07	5.31E-06
PLPP3	535.952004	2.206164856	0.368627	5.984816	2.17E-09	4.55E-08
FYB2	490.71429	6.323151336	0.457767	13.81304	2.13E-43	6.74E-41
C8B	17.8763406	4.945272106	1.216375	4.065582	4.79E-05	0.000376
DAB1	80.7064324	2.86999234	0.611375	4.694323	2.67E-06	2.96E-05
AL136985.3	13.9344094	4.350352124	1.260838	3.450364	0.00056	0.003011
JUN	5016.75103	-2.089349237	0.313431	-6.66606	2.63E-11	7.46E-10
LINC01135	18.8973522	3.822453977	1.086137	3.519311	0.000433	0.002431
C1orf87	14.5203918	3.671077699	1.162521	3.15786	0.001589	0.007106
LINC01748	46.6805786	4.081084168	0.92518	4.411123	1.03E-05	9.77E-05
L1TD1	103.122373	4.606570669	0.620758	7.420879	1.16E-13	4.49E-12
KANK4	74.6144514	2.225076864	0.617677	3.602333	0.000315	0.001877
LINC00466	31.1254507	5.786231795	1.159902	4.988554	6.08E-07	7.94E-06
FOXD3-AS1	12.8098164	4.225515716	1.267898	3.332692	0.00086	0.004288
ALG6	495.8475	2.000624563	0.417423	4.792797	1.64E-06	1.92E-05
LINC01359	13.7791919	4.396401629	1.256379	3.499265	0.000467	0.002588
AK4	807.08037	-2.742924308	0.349242	-7.85393	4.03E-15	1.80E-13
PDE4B	217.623321	4.867189787	0.552094	8.81587	1.19E-18	7.70E-17
SGIP1	700.668752	2.33661593	0.366917	6.368245	1.91E-10	4.74E-09
C1orf141	16.9713944	4.89148208	1.218905	4.013014	5.99E-05	0.000456
RPE65	441.468521	7.341201183	0.545996	13.44552	3.27E-41	9.47E-39
LRRC7	334.961595	4.083907681	0.419666	9.731337	2.22E-22	2.00E-20
ZRANB2-AS2	61.8922293	4.153009071	0.854133	4.862252	1.16E-06	1.40E-05
LINC01360	9.78454293	4.008050513	1.275572	3.14216	0.001677	0.007433
LRRIQ3	57.1515356	2.643300245	0.750087	3.523989	0.000425	0.002397
LRRC53	12.9650339	4.145609847	1.274605	3.252466	0.001144	0.005427
ERICH3	24.7000393	5.596356402	1.163914	4.808221	1.52E-06	1.80E-05
LHX8	13.0294632	4.313295341	1.261231	3.41991	0.000626	0.003303
ST6GALNAC3	420.299523	2.046390635	0.425599	4.808264	1.52E-06	1.80E-05
TPI1P1	245.816516	-2.374482182	0.49763	-4.77158	1.83E-06	2.10E-05
ST6GALNAC5	550.299202	-2.930919035	0.37105	-7.89898	2.81E-15	1.29E-13
AK5	826.414008	3.30559114	0.364302	9.073761	1.15E-19	8.31E-18
NEXN	2665.70543	-3.357536391	0.336896	-9.96608	2.15E-23	2.05E-21
AL157944.1	18.0959874	4.984482375	1.2129	4.109557	3.96E-05	0.000319
LINC01362	12.7834575	4.424631592	1.250368	3.538662	0.000402	0.002286
LINC01725	34.1487197	3.97693543	0.943198	4.216437	2.48E-05	0.000212
TTLL7-IT1	10.405413	3.972768242	1.280392	3.102774	0.001917	0.00826
SAMD13	53.0249968	3.614180828	0.78572	4.599835	4.23E-06	4.44E-05

MCOLN3	94.9126834	2.222349679	0.576959	3.851833	0.000117	0.000816
CCN1	11189.9497	-6.514010079	0.312617	-20.8371	2.00E-96	5.64E-93
LINC02801	20.5069152	3.541160797	1.056938	3.350396	0.000807	0.004068
PKN2-AS1	62.5762306	5.974435469	0.942239	6.34068	2.29E-10	5.60E-09
GBP1	1578.43204	-4.123442149	0.334155	-12.3399	5.52E-35	1.06E-32
GBP4	31.9249613	6.014818536	1.132671	5.310295	1.09E-07	1.67E-06
GBP5	11.5036471	4.283732053	1.258499	3.403843	0.000664	0.003466
GBP6	14.8129967	4.619407683	1.239067	3.728133	0.000193	0.001244
LRRC8C-DT	64.0399826	3.168302613	0.677666	4.675317	2.94E-06	3.21E-05
AL627316.1	12.2533757	4.381831723	1.252369	3.498834	0.000467	0.002588
AC092805.1	11.4128589	4.32259367	1.254608	3.445374	0.00057	0.003052
LINC02609	257.071961	4.238405483	0.477098	8.883722	6.47E-19	4.33E-17
HFM1	51.4114786	4.581620177	0.84752	5.405914	6.45E-08	1.03E-06
TGFBR3	331.611937	4.561654538	0.433057	10.53361	6.05E-26	6.65E-24
BRDT	12.62824	4.428525514	1.249417	3.544475	0.000393	0.00225
AL449283.1	13.8614513	3.570192994	1.176627	3.034261	0.002411	0.009958
BCAR3	3518.51867	-4.385005996	0.319842	-13.7099	8.86E-43	2.73E-40
ARHGAP29	958.990175	-2.05555154	0.356378	-5.76789	8.03E-09	1.52E-07
F3	5597.79284	-3.054881321	0.334184	-9.14132	6.17E-20	4.61E-18
TLCD4	555.511789	3.054516569	0.39257	7.780822	7.21E-15	3.11E-13
AC092802.1	21.7579566	3.006861053	0.99112	3.033803	0.002415	0.009972
LINC02607	31.6145263	6.014450742	1.131771	5.314195	1.07E-07	1.64E-06
LINC02790	12.3441639	4.315094906	1.258772	3.428019	0.000608	0.003216
EEF1A1P11	183.845119	2.76937337	0.489182	5.661229	1.50E-08	2.71E-07
PLPPR5	78.5051401	6.315872323	0.87658	7.205133	5.80E-13	2.04E-11
AL445433.2	16.3241655	4.95813772	1.209077	4.100762	4.12E-05	0.000329
PLPPR4	81.2107971	7.528386627	1.021439	7.370371	1.70E-13	6.37E-12
LRRC39	128.267707	3.236540422	0.561984	5.759134	8.45E-09	1.59E-07
CDC14A	140.907529	2.036837124	0.494832	4.116224	3.85E-05	0.000311
VCAM1	17.4751174	5.001808518	1.208684	4.138227	3.50E-05	0.000285
LINC01709	11.8140821	4.278206312	1.260145	3.395012	0.000686	0.003556
RPSAP19	1049.53475	2.927397388	0.361317	8.102015	5.41E-16	2.73E-14
OLFM3	11.7496528	4.203681987	1.266356	3.31951	0.000902	0.004457
LINC01677	9.09924358	3.9280641	1.279421	3.07019	0.002139	0.009014
AL355306.2	19.0272925	4.863730184	1.228502	3.959072	7.52E-05	0.000554
LINC01661	11.2840002	4.232661173	1.262212	3.353368	0.000798	0.004039
VAV3	189.007815	3.554850667	0.480178	7.403195	1.33E-13	5.09E-12
LINC02785	19.6749272	3.801080358	1.148054	3.310891	0.00093	0.004582
MYBPHL	69.0055691	2.177485237	0.645096	3.375442	0.000737	0.003778
SYPL2	21.9207716	5.436091077	1.174084	4.63007	3.66E-06	3.91E-05
GPR61	28.5120388	2.92218453	0.905285	3.227917	0.001247	0.005815
GSTM1	117.09688	7.264276059	0.925529	7.848785	4.20E-15	1.87E-13
SLC16A4	674.693666	-2.864405418	0.361217	-7.92987	2.19E-15	1.03E-13
KCNA2	42.2132272	6.442858604	1.102429	5.84424	5.09E-09	9.97E-08

KCNA3	11.9429407	4.379058258	1.251429	3.499245	0.000467	0.002588
PIFO	431.09537	3.025004015	0.435492	6.946169	3.75E-12	1.18E-10
KCND3	298.380178	2.219282644	0.467955	4.742517	2.11E-06	2.39E-05
LINC02884	24.4718637	4.574333845	1.0569	4.328066	1.50E-05	0.000137
CTTNBP2NL	1138.93211	-2.208176444	0.334261	-6.60615	3.94E-11	1.10E-09
WNT2B	2733.87946	-2.546151781	0.320306	-7.94913	1.88E-15	8.93E-14
CAPZA1	4217.05049	-2.141273011	0.349092	-6.13384	8.58E-10	1.91E-08
MOV10	2268.26445	-2.256886471	0.334592	-6.74519	1.53E-11	4.46E-10
RHOC	9172.41948	-3.798275466	0.388673	-9.77241	1.48E-22	1.34E-20
HIPK1-AS1	14.5848211	3.694116774	1.157746	3.190782	0.001419	0.006461
SYT6	21.8299834	5.452601671	1.171696	4.653596	3.26E-06	3.52E-05
DENND2C	95.5530542	2.568820636	0.57812	4.443402	8.85E-06	8.56E-05
AMPD1	14.1657678	4.710734189	1.227281	3.83835	0.000124	0.000852
SYCP1	23.2908532	3.412464779	1.026957	3.322889	0.000891	0.004417
NGF-AS1	10.2501954	3.994010754	1.278276	3.124529	0.001781	0.007777
NGF	156.14584	-3.526192169	0.52475	-6.71975	1.82E-11	5.26E-10
CASQ2	38.0487134	6.386332321	1.099291	5.809499	6.27E-09	1.21E-07
U3	41.6049694	3.084760052	0.888076	3.473531	0.000514	0.0028
NEFHP1	9.56489611	3.948402431	1.27948	3.085944	0.002029	0.008665
VTCN1	10.3146248	4.067189627	1.272691	3.195739	0.001395	0.006369
TENT5C	136.350345	4.074612433	0.569748	7.151607	8.58E-13	2.94E-11
SPAG17	75.822933	2.752886861	0.630624	4.36534	1.27E-05	0.000118
TBX15	16.3241655	4.95813772	1.209077	4.100762	4.12E-05	0.000329
LINC01780	9.38331971	4.017150993	1.273395	3.154678	0.001607	0.00717
HMGCS2	72.0178295	7.334836772	1.035802	7.08131	1.43E-12	4.74E-11
NOTCH2	5370.17277	-2.252103853	0.335134	-6.72001	1.82E-11	5.25E-10
EMBP1	42.463347	4.07197957	0.857447	4.748958	2.04E-06	2.32E-05
AC239859.5	56.9301276	-2.318025827	0.708415	-3.27213	0.001067	0.005106
AC239859.1	28.2558005	4.587895824	1.040371	4.409866	1.03E-05	9.82E-05
NBPF17P	23.3661825	4.754340242	1.138195	4.177087	2.95E-05	0.000246
AC239800.2	18.585063	5.234366707	1.186012	4.413417	1.02E-05	9.68E-05
AC239800.1	11.8785114	4.334365593	1.255325	3.452784	0.000555	0.002989
H2BP2	19.3757979	5.06034134	1.20905	4.185387	2.85E-05	0.000238
CD160	10.1594073	4.067721584	1.272091	3.197666	0.001385	0.006342
ANKRD34A	113.631327	-3.329352466	0.61963	-5.37313	7.74E-08	1.22E-06
BCL9	733.518562	-2.121790828	0.35695	-5.94422	2.78E-09	5.74E-08
LINC02805	11.1141354	4.369419703	1.249271	3.497575	0.00047	0.002598
SF3B4	1371.54835	-2.432778151	0.372241	-6.5355	6.34E-11	1.70E-09
PLEKHO1	1586.08646	-2.102065321	0.342843	-6.13127	8.72E-10	1.94E-08
CA14	776.585668	4.987992562	1.018815	4.895877	9.79E-07	1.21E-05
ECM1	241.123948	-2.635243892	0.466584	-5.64795	1.62E-08	2.91E-07
ADAMTSL4	395.400125	2.955162389	0.397803	7.428714	1.10E-13	4.24E-12
HORMAD1	9.53853722	4.032853085	1.27268	3.168788	0.001531	0.006875
CDC42SE1	1712.17297	-2.869886232	0.328458	-8.73745	2.38E-18	1.51E-16

MLLT11	1009.91363	-2.155541788	0.403036	-5.34826	8.88E-08	1.38E-06
PIP5K1A	1905.73483	-2.437425128	0.320514	-7.60473	2.86E-14	1.17E-12
TUFT1	2221.76937	-4.275158431	0.327245	-13.0641	5.28E-39	1.33E-36
CELF3	21.8130846	3.573769493	1.071625	3.334907	0.000853	0.004258
LINGO4	13.1056041	4.607910412	1.233642	3.735209	0.000188	0.001214
RORC	262.818211	8.138887106	0.768897	10.58514	3.49E-26	3.96E-24
C2CD4D	81.4669327	3.545099251	0.626761	5.656218	1.55E-08	2.78E-07
C2CD4D-AS1	16.5997129	3.967390519	1.118987	3.545519	0.000392	0.002245
THEM5	20.3929509	3.251250464	0.995605	3.265603	0.001092	0.005212
S100A11	4550.7871	-3.236000168	0.438824	-7.37426	1.65E-13	6.22E-12
RPTN	14.7222085	4.681425272	1.232515	3.798272	0.000146	0.000979
FLG2	17.791671	3.854233215	1.16584	3.305971	0.000946	0.004655
IVL	156.563275	-4.50270013	0.66758	-6.74481	1.53E-11	4.46E-10
S100A7A	14.1540562	4.436031606	1.254048	3.537369	0.000404	0.002294
S100A6	12434.9877	-3.16604882	0.401851	-7.87866	3.31E-15	1.50E-13
S100A3	36.6496829	-2.487902974	0.803015	-3.0982	0.001947	0.008371
S100A2	231.333447	-2.593742503	0.450119	-5.76235	8.30E-09	1.57E-07
S100A16	3081.77321	-2.863972253	0.38853	-7.37131	1.69E-13	6.34E-12
NPR1	16.3505244	4.904046298	1.215189	4.035623	5.45E-05	0.00042
SLC39A1	57.7715758	-2.468583219	0.693513	-3.55953	0.000372	0.002146
IL6R-AS1	34.3735537	5.057922249	1.020538	4.956135	7.19E-07	9.23E-06
IL6R	612.455074	4.68347257	0.392752	11.92477	8.79E-33	1.52E-30
SHE	18.0230293	4.034090188	1.122501	3.593842	0.000326	0.001931
TDRD10	8.94402608	3.914238868	1.279942	3.058139	0.002227	0.009318
CHRNB2	15.5805554	3.759291709	1.157359	3.248163	0.001162	0.005473
KCNN3	56.9798887	3.208193498	0.74013	4.334632	1.46E-05	0.000133
PMVK	3216.90645	2.214941682	0.319893	6.924011	4.39E-12	1.37E-10
SHC1	8008.06979	-3.876706089	0.338899	-11.4391	2.67E-30	3.86E-28
EFNA4	268.023469	-2.202323738	0.490719	-4.48796	7.19E-06	7.11E-05
EFNA1	139.961264	-2.35149438	0.521085	-4.51269	6.40E-06	6.40E-05
TRIM46	1466.52097	-3.153452828	0.333304	-9.4612	3.04E-21	2.49E-19
MUC1	281.405029	-2.870034181	0.429581	-6.68101	2.37E-11	6.78E-10
THBS3-AS1	59.7546232	-2.335810327	0.679446	-3.43782	0.000586	0.00312
THBS3	1216.79368	-3.684439558	0.344048	-10.7091	9.23E-27	1.08E-24
MTX1P1	189.90644	-2.228348749	0.48137	-4.62918	3.67E-06	3.92E-05
PKLR	14.1921267	4.645323689	1.234155	3.76397	0.000167	0.001102
AL353807.5	44.6252123	-4.013239026	0.901346	-4.45249	8.49E-06	8.24E-05
ARHGEF2	1488.82597	-2.09861384	0.326992	-6.41794	1.38E-10	3.51E-09
LMNA	18647.9583	-2.237831792	0.360042	-6.21548	5.12E-10	1.19E-08
RHBG	17.8119113	4.887945199	1.222233	3.999192	6.36E-05	0.00048
AL139130.1	14.7222085	4.681425272	1.232515	3.798272	0.000146	0.000979
MIR9-1HG	62.3803101	3.394474067	0.721319	4.705926	2.53E-06	2.81E-05
BCAN	146.289477	4.350496378	0.540686	8.046251	8.54E-16	4.23E-14
AL365181.3	119.789471	-2.044892629	0.593611	-3.44483	0.000571	0.003057

NTRK1	27.5700953	5.706603032	1.159427	4.921917	8.57E-07	1.08E-05
PEAR1	750.271517	-4.570241707	0.386746	-11.8172	3.18E-32	5.09E-30
AL357143.1	21.2237607	5.281155174	1.19031	4.436789	9.13E-06	8.80E-05
FCRL5	21.3906897	5.408849229	1.17549	4.601355	4.20E-06	4.41E-05
FCRL4	10.5342716	4.124847874	1.268711	3.25121	0.001149	0.005437
FCRL3	21.8182718	5.334374844	1.186092	4.497439	6.88E-06	6.83E-05
FCRL2	11.3103591	4.03853363	1.278167	3.159629	0.00158	0.007069
CD5L	12.62824	4.428525514	1.249417	3.544475	0.000393	0.00225
OR10K2	10.6250598	4.049542664	1.275176	3.175673	0.001495	0.006735
OR6Y1	12.2533757	4.381831723	1.252369	3.498834	0.000467	0.002588
OR6P1	9.69375473	4.046019533	1.27217	3.180407	0.001471	0.006648
OR10Z1	13.5976155	4.561543505	1.240264	3.677881	0.000235	0.001464
SPTA1	35.7883414	4.505336852	0.986047	4.569089	4.90E-06	5.05E-05
PYHIN1	13.3135394	4.460011267	1.248997	3.570873	0.000356	0.002065
AIM2	11.6588646	4.283776559	1.259077	3.402316	0.000668	0.003478
FCRL6	11.1287827	4.232433809	1.261651	3.354678	0.000795	0.004025
TAGLN2	14029.2987	-2.963203313	0.41002	-7.22698	4.94E-13	1.75E-11
IGSF9	936.065872	2.350574692	0.385118	6.103525	1.04E-09	2.28E-08
ATP1A2	25.7239867	4.184405124	1.076498	3.887054	0.000101	0.000721
ATP1A4	21.1216667	4.052333647	1.102762	3.674714	0.000238	0.001479
PEA15	9632.1948	-2.259758435	0.351304	-6.43249	1.26E-10	3.21E-09
NCSTN	3368.50409	-2.861627912	0.317198	-9.02157	1.85E-19	1.31E-17
VANGL2	553.636175	-2.025365437	0.38808	-5.21894	1.80E-07	2.63E-06
CD84	23.0717234	5.461781762	1.175049	4.64813	3.35E-06	3.61E-05
SLAMF1	14.4762028	4.720153283	1.227539	3.845216	0.00012	0.000832
LY9	18.3800636	5.060486706	1.20549	4.197869	2.69E-05	0.000227
AL354714.1	9.87533112	3.936139891	1.281456	3.071616	0.002129	0.008981
NR1I3	24.7041534	3.230160714	0.952528	3.391145	0.000696	0.003598
FCGR2A	18.9072096	5.288980435	1.180853	4.47895	7.50E-06	7.37E-05
OLFML2B	87.3456495	-2.578118567	0.711692	-3.62252	0.000292	0.001755
SPATA46	10.7802773	4.027023489	1.277437	3.152423	0.001619	0.007214
CCDC190	95.7969051	-2.784007533	0.695076	-4.00533	6.19E-05	0.00047
RGS4	2026.85945	-3.567641814	0.324252	-11.0027	3.71E-28	4.85E-26
RGS5	4156.90416	-4.797994113	0.315268	-15.2188	2.65E-52	1.50E-49
LMX1A	18.3071054	4.078589226	1.114505	3.659553	0.000253	0.001553
RXRG	18.9749805	3.950680322	1.100749	3.589082	0.000332	0.001956
LRRC52-AS1	22.7495769	5.285376203	1.194895	4.423298	9.72E-06	9.29E-05
AL356441.1	10.2501954	3.994010754	1.278276	3.124529	0.001781	0.007777
FAM78B	679.743555	4.059137523	0.400648	10.13142	4.01E-24	4.03E-22
AL596087.2	62.321139	7.015965583	1.065874	6.582362	4.63E-11	1.27E-09
RPL4P2	14.1953094	3.704280056	1.149899	3.221397	0.001276	0.005925
ILDR2	62.7438466	2.377474275	0.72056	3.29948	0.000969	0.004726
CD247	11.0379945	4.269395787	1.258002	3.393791	0.000689	0.003567
AL359962.3	11.193212	4.276943502	1.257921	3.400009	0.000674	0.003502

MPZL1	2370.55334	-2.183430327	0.333345	-6.55005	5.75E-11	1.55E-09
ADCY10	26.7559374	5.642131227	1.164961	4.843195	1.28E-06	1.53E-05
F5	32.3124685	3.928265274	0.932616	4.212095	2.53E-05	0.000215
SELE	15.187861	4.656333116	1.236755	3.764961	0.000167	0.001099
PRRX1	299.479168	2.659810371	0.418491	6.35572	2.07E-10	5.12E-09
BX284613.2	20.9311557	4.280801356	1.095761	3.906694	9.36E-05	0.00067
MROH9	15.2522903	4.713246257	1.231253	3.828008	0.000129	0.000883
FMO2	19.4402272	5.106787881	1.204105	4.241147	2.22E-05	0.000192
MYOC	144.088292	-2.324789828	0.504615	-4.60705	4.08E-06	4.31E-05
PFN1P1	732.495385	-2.030618215	0.417865	-4.85951	1.18E-06	1.42E-05
DNM3	149.931768	2.558435997	0.510185	5.014719	5.31E-07	7.05E-06
TNFSF18	114.304841	-4.009687359	0.707658	-5.66614	1.46E-08	2.64E-07
SLC9C2	21.1593314	5.244799949	1.19433	4.391414	1.13E-05	0.000106
ANKRD45	34.2935873	2.9167416	0.849118	3.435023	0.000593	0.003148
RABGAP1L-DT	27.3922272	3.671915538	1.019373	3.602131	0.000316	0.001878
TNN	11.8785114	4.334365593	1.255325	3.452784	0.000555	0.002989
TNR	37.2642559	5.061623372	0.991677	5.104105	3.32E-07	4.62E-06
PAPPA2	33.8335033	3.126031972	0.873278	3.579654	0.000344	0.002012
LINC01645	11.3747884	4.154235208	1.269217	3.273069	0.001064	0.00509
CLEC20A	15.0062846	4.760747063	1.225366	3.885164	0.000102	0.000725
TEX35	13.0031044	4.473281904	1.246584	3.588433	0.000333	0.001957
ANGPTL1	25.7868088	4.7044839	1.035707	4.542292	5.56E-06	5.65E-05
AXDND1	23.6577058	4.514740352	1.0642	4.24238	2.21E-05	0.000192
HNRNPA1P54	31.7316734	5.962215082	1.139105	5.234123	1.66E-07	2.44E-06
AL160286.3	12.2914461	4.487496012	1.242404	3.611945	0.000304	0.001818
FAM163A	24.0000927	5.663202137	1.152915	4.912074	9.01E-07	1.13E-05
QSOX1	27826.1839	-2.491427002	0.32723	-7.61368	2.66E-14	1.10E-12
KIAA1614	188.917161	3.531892759	0.521897	6.767409	1.31E-11	3.86E-10
CACNA1E	68.7118531	5.993726383	0.923013	6.493655	8.38E-11	2.20E-09
ZNF648	11.8257936	4.449235975	1.24429	3.575724	0.000349	0.002038
AL355482.1	9.51217834	4.071977747	1.269403	3.20779	0.001338	0.006166
LINC01344	23.549613	3.655210858	1.126857	3.243723	0.00118	0.005539
RGSL1	28.0596965	4.070952507	1.048247	3.883582	0.000103	0.000729
RGS16	46.7677967	2.374521365	0.739278	3.211945	0.001318	0.006089
LAMC1	4809.78299	-2.562684218	0.344134	-7.44677	9.57E-14	3.72E-12
LAMC2	2462.83487	-6.128060092	0.515765	-11.8815	1.48E-32	2.45E-30
AL713852.1	18.9862862	5.240148159	1.186859	4.41514	1.01E-05	9.61E-05
PLA2G4A	101.858302	2.718652045	0.564483	4.816179	1.46E-06	1.74E-05
LINC01036	31.265098	4.399975551	1.020018	4.313627	1.61E-05	0.000145
BRINP3	17.7506647	4.061048864	1.110892	3.655664	0.000257	0.001573
AL713866.1	11.3103591	4.03853363	1.278167	3.159629	0.00158	0.007069
RGS18	12.4993814	4.297396672	1.260864	3.408295	0.000654	0.003427
AL390957.1	40.764642	4.000248612	0.985718	4.058209	4.95E-05	0.000386
RGS21	9.72011361	3.945326647	1.280246	3.081694	0.002058	0.008746

AL353709.1	20.977755	5.31631952	1.185244	4.48542	7.28E-06	7.19E-05
CFHR1	249.225796	3.637253986	0.509948	7.132593	9.85E-13	3.36E-11
ASPM	902.454898	2.612924878	0.347246	7.524707	5.28E-14	2.11E-12
CRB1	41.2004523	2.649461243	0.840978	3.150454	0.00163	0.007258
LHX9	29.1932241	4.06923876	1.009726	4.030044	5.58E-05	0.000428
ATP6V1G3	21.6044649	5.35814196	1.183948	4.525657	6.02E-06	6.06E-05
NR5A2	28.0192549	3.469224459	1.020316	3.400147	0.000673	0.003502
MROH3P	12.7834575	4.424631592	1.250368	3.538662	0.000402	0.002286
KIF21B	1849.74811	4.876163887	0.340844	14.30616	2.00E-46	7.80E-44
AL358473.1	81.9019363	7.495104973	1.02821	7.289469	3.11E-13	1.13E-11
CACNA1S	21.1066136	5.36488684	1.179834	4.547154	5.44E-06	5.55E-05
ASCL5	10.0949779	4.005107431	1.276891	3.136609	0.001709	0.007522
PKP1	877.863463	3.19804961	0.344434	9.284941	1.62E-20	1.25E-18
TNNI1	29.3653403	5.914214209	1.138134	5.196411	2.03E-07	2.94E-06
CSRP1	3853.01878	-3.221437899	0.321859	-10.0088	1.39E-23	1.35E-21
RNPEP	2971.28032	-2.22552724	0.32995	-6.74504	1.53E-11	4.46E-10
GPR37L1	54.1561618	2.840202235	0.701102	4.051057	5.10E-05	0.000396
PTPN7	29.3954075	3.79467295	1.000511	3.792736	0.000149	0.000997
AC099336.2	76.4427373	2.963643584	0.681261	4.350234	1.36E-05	0.000125
SYT2	23.7367824	4.419805354	1.093292	4.042659	5.28E-05	0.000409
PCAT6	199.908687	-3.64526258	0.491074	-7.42304	1.14E-13	4.42E-12
PPFIA4	496.617682	-2.023696703	0.401515	-5.04015	4.65E-07	6.26E-06
MYOG	12.1245171	4.251364794	1.263572	3.36456	0.000767	0.003905
MYBPH	11.0643534	4.176955156	1.266227	3.298742	0.000971	0.004726
CHIT1	38.0399376	6.180569756	1.127615	5.481098	4.23E-08	7.03E-07
ATP2B4	5435.89901	-2.248339734	0.319219	-7.04325	1.88E-12	6.14E-11
AL592114.1	14.9760671	3.351734114	1.101938	3.041672	0.002353	0.00976
LRRN2	1379.8418	2.630895879	0.361559	7.276534	3.43E-13	1.24E-11
NFASC	1738.88172	-2.087124462	0.39685	-5.25922	1.45E-07	2.15E-06
CNTN2	285.669914	9.215914185	0.937582	9.829451	8.41E-23	7.81E-21
KLHDC8A	166.384597	2.220394413	0.5018	4.424862	9.65E-06	9.23E-05
MFSD4A	151.010345	3.442746573	0.559681	6.151265	7.69E-10	1.73E-08
SLC45A3	214.056497	-3.123748702	0.520116	-6.00587	1.90E-09	4.03E-08
SLC26A9	15.5627253	4.692214931	1.234504	3.800889	0.000144	0.00097
RAB7B	190.570794	-2.938393633	0.619319	-4.74455	2.09E-06	2.37E-05
CTSE	9.84897224	4.056445929	1.271884	3.189321	0.001426	0.006475
AL591846.1	45.8740696	-3.156984885	0.777297	-4.06149	4.88E-05	0.000381
IL10	13.6239744	4.426435034	1.253183	3.532154	0.000412	0.002334
IL19	19.349439	5.149628194	1.198899	4.295298	1.74E-05	0.000155
CR2	19.4025626	3.911717219	1.116844	3.502473	0.000461	0.002568
CR1	18.0051992	5.030607371	1.207457	4.166283	3.10E-05	0.000256
MIR29B2CHG	73.5280766	4.209934364	0.791694	5.317627	1.05E-07	1.61E-06
AL356275.1	11.4772882	4.356824519	1.251683	3.480772	0.0005	0.002731
LINC02767	20.6132736	4.494165365	1.167019	3.850978	0.000118	0.000818

SYT14	398.45153	2.395150388	0.387117	6.187146	6.13E-10	1.40E-08
SERTAD4-AS1	105.824331	-2.099208374	0.55841	-3.75926	0.00017	0.001119
AC092017.1	18.2839293	3.503561799	1.086119	3.225762	0.001256	0.005851
RPL21P28	199.690908	3.174739749	0.480922	6.601361	4.07E-11	1.13E-09
PROX1-AS1	28.0244421	4.464237739	1.071819	4.165104	3.11E-05	0.000257
PROX1	31.8436332	4.150534057	1.017712	4.078298	4.54E-05	0.000358
SMYD2	461.88546	-2.825279352	0.385952	-7.32028	2.47E-13	9.13E-12
USH2A	67.3327342	4.090138872	0.77524	5.275966	1.32E-07	1.98E-06
ESRRG	101.138941	3.041844846	0.574831	5.291721	1.21E-07	1.84E-06
TGFB2	2711.19433	-4.651523954	0.335473	-13.8656	1.02E-43	3.34E-41
SLC30A10	50.4579764	3.014804751	0.721449	4.17882	2.93E-05	0.000244
MTARC2	244.108597	2.599651384	0.443006	5.868202	4.41E-09	8.73E-08
AL392172.1	10.7539184	4.178531017	1.264949	3.303319	0.000955	0.004683
AL929091.1	17.6566938	4.903090935	1.22011	4.018563	5.86E-05	0.000447
TLR5	50.924185	3.444789449	0.76879	4.480793	7.44E-06	7.32E-05
CAPN8	24.8435453	5.428252261	1.184878	4.581275	4.62E-06	4.80E-05
CAPN2	21246.4525	-2.765041432	0.301691	-9.16515	4.95E-20	3.73E-18
ACTBP11	69.5051719	-3.755596655	0.739409	-5.07919	3.79E-07	5.21E-06
ENAH	2943.79688	-2.494396134	0.323614	-7.70793	1.28E-14	5.40E-13
STUM	35.8522452	6.277677409	1.108287	5.664307	1.48E-08	2.66E-07
AL731702.1	36.0426144	2.731261972	0.819734	3.33189	0.000863	0.004298
WNT9A	750.269729	-2.384515634	0.350521	-6.80278	1.03E-11	3.05E-10
LINC02809	11.8140821	4.278206312	1.260145	3.395012	0.000686	0.003556
TRIM17	416.13826	-3.710302424	0.413874	-8.96481	3.11E-19	2.16E-17
LINC02814	9.87533112	3.936139891	1.281456	3.071616	0.002129	0.008981
ACTA1	45.3376368	3.117028257	0.897698	3.472245	0.000516	0.002811
LINC01682	11.2840002	4.232661173	1.262212	3.353368	0.000798	0.004039
LINC01736	9.48581945	4.084682979	1.268368	3.220423	0.00128	0.005941
GALNT2	8398.27682	-3.098885863	0.308708	-10.0383	1.03E-23	1.01E-21
PGBD5	71.2675568	2.043365095	0.639599	3.194761	0.001399	0.006382
AGT	23.9976824	3.964697154	1.0197	3.888102	0.000101	0.000719
CAPN9	15.2903607	4.82792795	1.219301	3.959585	7.51E-05	0.000553
FAM89A	201.981974	4.046997701	0.488332	8.287394	1.16E-16	6.21E-15
TRIM67	88.1634912	4.05714192	0.664295	6.107437	1.01E-09	2.23E-08
AL136171.2	33.7992829	6.090486684	1.128057	5.399093	6.70E-08	1.07E-06
AL663058.2	11.1287827	4.232433809	1.261651	3.354678	0.000795	0.004025
GNG4	23.560799	5.629107457	1.155719	4.870655	1.11E-06	1.35E-05
ACTN2	51.1218571	3.395568755	0.790153	4.297357	1.73E-05	0.000154
RYR2	101.022984	7.667693502	1.028465	7.455476	8.95E-14	3.50E-12
LINC01139	18.7168575	4.92681021	1.221153	4.034555	5.47E-05	0.000421
AL583825.1	13.3779687	4.51654038	1.243835	3.63114	0.000282	0.001704
AC093426.2	13.2871805	4.560426404	1.239161	3.680253	0.000233	0.001453
AC093426.1	10.5606305	3.934530818	1.283673	3.065058	0.002176	0.009142
CHRM3-AS2	23.5524291	4.141911744	1.108614	3.736118	0.000187	0.001211

RGS7	32.4818078	4.845997313	1.010119	4.797454	1.61E-06	1.88E-05
AL359764.1	14.1013385	4.679991737	1.230232	3.804153	0.000142	0.000961
AL359764.2	34.8145182	4.682454979	0.991578	4.722228	2.33E-06	2.61E-05
KMO	88.4963021	-2.132785292	0.683686	-3.11954	0.001811	0.007878
OPN3	181.292668	-3.329812862	0.485721	-6.8554	7.11E-12	2.15E-10
PLD5	1004.95108	3.26019183	0.357982	9.107138	8.46E-20	6.19E-18
AL590483.3	15.4075078	4.704549513	1.232708	3.816434	0.000135	0.000921
AL390728.4	197.444154	-2.211318774	0.461452	-4.79208	1.65E-06	1.93E-05
NLRP3	17.9938935	3.762090308	1.135702	3.312567	0.000924	0.004556
OR2B11	13.0675337	4.51829105	1.242467	3.636549	0.000276	0.001677
GCSAML	20.784467	5.219624126	1.195947	4.364428	1.27E-05	0.000118
OR14L1P	9.09924358	3.9280641	1.279421	3.07019	0.002139	0.009014
OR1C1	12.0600878	4.135111753	1.272919	3.248527	0.00116	0.005468
OR9H1P	13.9988387	4.466753428	1.25076	3.571231	0.000355	0.002065
TRIM58	413.310962	-2.054095775	0.390357	-5.26209	1.42E-07	2.13E-06
OR2L3	10.405413	3.972768242	1.280392	3.102774	0.001917	0.00826
OR2M3	23.5110171	5.508821902	1.170766	4.705316	2.53E-06	2.81E-05
OR2T12	13.2491101	4.384912975	1.255665	3.492104	0.000479	0.002637
OR2T6	13.4043276	4.355513885	1.258772	3.460129	0.00054	0.002922
OR2T1	11.3747884	4.154235208	1.269217	3.273069	0.001064	0.00509
OR2G6	10.1594073	4.067721584	1.272091	3.197666	0.001385	0.006342
SH3BP5L	2000.74692	-2.169728653	0.388613	-5.58327	2.36E-08	4.09E-07
AC079779.3	14.5467506	3.62241693	1.175044	3.082793	0.002051	0.008733
AC093326.1	11.5036471	4.283732053	1.258499	3.403843	0.000664	0.003466
SNTG2	17.5926703	3.777317693	1.126072	3.354418	0.000795	0.004028
TPO	40.3154825	6.202598601	1.130345	5.487349	4.08E-08	6.80E-07
PXDN	11497.0782	-2.72829559	0.313119	-8.7133	2.95E-18	1.85E-16
MYT1L	103.055459	7.597737679	1.041128	7.297601	2.93E-13	1.07E-11
AC093390.1	10.5606305	3.934530818	1.283673	3.065058	0.002176	0.009142
AC018685.2	18.2892754	5.094848856	1.201233	4.241351	2.22E-05	0.000192
AC011995.2	34.3477595	3.97663596	1.061395	3.746614	0.000179	0.001168
COLEC11	12.821528	4.544094362	1.238932	3.667753	0.000245	0.001511
ALLC	12.3822343	4.464991456	1.24496	3.586452	0.000335	0.001968
LINC01304	21.4029268	3.648332933	1.093194	3.337316	0.000846	0.004227
LINC01249	12.0981582	4.381361397	1.251818	3.499999	0.000465	0.002588
MIR7515HG	27.3081137	5.054062943	1.105864	4.57024	4.87E-06	5.03E-05
GRASLND	21.2679496	4.248920198	1.109485	3.829632	0.000128	0.000879
AC013460.1	34.4845823	6.102870954	1.128368	5.408584	6.35E-08	1.02E-06
LINC00299	53.1294521	4.29718511	0.953358	4.507421	6.56E-06	6.54E-05
LINC01814	36.7247224	3.901145321	0.888496	4.390727	1.13E-05	0.000106
ID2	667.734131	-3.294636312	0.362722	-9.0831	1.06E-19	7.66E-18
AC082651.4	12.6018811	4.505303377	1.241896	3.627762	0.000286	0.001725
HPCAL1	1603.08091	-2.14271854	0.327825	-6.53616	6.31E-11	1.69E-09
ATP6V1C2	376.279948	4.144111134	0.43227	9.586854	9.08E-22	7.77E-20

KCNF1	800.528391	7.358869674	0.498291	14.76821	2.35E-49	1.09E-46
C2orf50	30.2058572	5.933862742	1.13819	5.21342	1.85E-07	2.71E-06
LINC00570	9.72011361	3.945326647	1.280246	3.081694	0.002058	0.008746
AC099344.3	25.0368332	5.538851586	1.172224	4.72508	2.30E-06	2.58E-05
GREB1	111.41931	2.020456956	0.576635	3.503875	0.000459	0.002557
MIR3681HG	55.0999961	5.046669642	0.874934	5.768056	8.02E-09	1.52E-07
TRIB2	2474.04239	4.257665528	0.336879	12.63857	1.29E-36	2.74E-34
AC064875.1	19.1795742	5.287178864	1.181977	4.473167	7.71E-06	7.56E-05
AC093912.1	13.5068273	4.600284725	1.236028	3.721829	0.000198	0.001268
LINC00276	44.9221941	6.664472917	1.077848	6.183126	6.28E-10	1.44E-08
LRATD1	1423.61965	3.921562163	0.331143	11.8425	2.35E-32	3.83E-30
LINC01804	12.2270168	4.457657865	1.245054	3.580294	0.000343	0.002009
MYCN	137.682324	-2.974060416	0.617918	-4.81303	1.49E-06	1.76E-05
AC010745.2	10.0422602	4.156981159	1.264143	3.288379	0.001008	0.004862
AC008164.1	10.1330484	4.143717258	1.26561	3.274087	0.00106	0.005082
VSNL1	54.4205538	3.094016861	0.714173	4.332305	1.48E-05	0.000134
SMC6	3254.32628	2.580841902	0.340481	7.579981	3.46E-14	1.40E-12
GEN1	469.823571	2.062754823	0.377509	5.464117	4.65E-08	7.67E-07
MSGN1	9.47410791	3.993729441	1.275601	3.130862	0.001743	0.007649
LINC00954	38.0416414	3.418101821	0.872702	3.91669	8.98E-05	0.000647
SDC1	906.503893	-4.12030235	0.37215	-11.0716	1.72E-28	2.30E-26
RPS25P3	26.0442791	5.67848686	1.158036	4.90355	9.41E-07	1.17E-05
GDF7	22.8934886	4.04746508	1.125045	3.597601	0.000321	0.001907
APOB	42.9512443	6.391769058	1.111353	5.751338	8.85E-09	1.66E-07
TDRD15	15.5627253	4.692214931	1.234504	3.800889	0.000144	0.00097
AC096570.1	18.2512049	4.975026463	1.214478	4.096431	4.20E-05	0.000334
LINC01884	9.72011361	3.945326647	1.280246	3.081694	0.002058	0.008746
AC009242.1	33.0487281	2.994554839	0.86631	3.456676	0.000547	0.002956
TP53I3	1149.09117	-2.074266798	0.387747	-5.34953	8.82E-08	1.37E-06
DNAJC27-AS1	89.8767849	2.093718934	0.602928	3.472586	0.000515	0.002809
POMC	131.449671	3.729608484	0.652941	5.712017	1.12E-08	2.06E-07
KIF3C	1710.41364	-2.312410575	0.37614	-6.14774	7.86E-10	1.77E-08
RAB10	2770.79961	-2.276897872	0.327224	-6.95822	3.45E-12	1.09E-10
DRC1	15.72539	4.297263437	1.158395	3.709672	0.000208	0.001318
OTOF	29.5619699	4.610877936	1.047494	4.401817	1.07E-05	0.000102
DPYSL5	681.125135	10.35873299	0.892881	11.60148	4.05E-31	6.13E-29
TRIM54	13.131963	4.556748427	1.238911	3.678026	0.000235	0.001464
UCN	75.843606	2.107532171	0.67504	3.122083	0.001796	0.007832
FNDC4	155.840193	-3.596825245	0.556674	-6.46128	1.04E-10	2.68E-09
FOSL2	4893.18834	-2.886411188	0.361309	-7.98876	1.36E-15	6.60E-14
TOGARAM2	119.502033	4.857781205	0.612968	7.925018	2.28E-15	1.06E-13
PCARE	112.353831	7.897142165	1.007994	7.834511	4.71E-15	2.08E-13
ALK	29.6701823	4.716404178	1.075584	4.38497	1.16E-05	0.000109
LBH	19069.3769	-2.401627318	0.319799	-7.50979	5.92E-14	2.36E-12

CAPN13	24.5975396	5.486096555	1.177227	4.660187	3.16E-06	3.43E-05
SRD5A2	10.8447066	4.116553698	1.270511	3.240076	0.001195	0.005595
LINC01320	32.9470545	5.984929676	1.139665	5.251483	1.51E-07	2.24E-06
AC008170.1	13.9724798	4.602774761	1.237603	3.719104	0.0002	0.00128
AC012593.1	14.9037849	4.512217334	1.249531	3.611129	0.000305	0.001821
CDC42EP3	3527.80489	-3.813754282	0.313761	-12.155	5.40E-34	9.79E-32
LINC02613	20.9664492	4.050631403	1.101208	3.678352	0.000235	0.001463
SLC8A1-AS1	22.6385483	4.292980211	1.114986	3.850255	0.000118	0.000819
LINC01913	16.3768833	4.818032405	1.224528	3.934605	8.33E-05	0.000606
KCNG3	18.0696285	5.064439282	1.203874	4.206784	2.59E-05	0.00022
OXER1	26.8300738	3.694679143	0.935342	3.950086	7.81E-05	0.000573
LINC01819	11.9810111	4.463474905	1.243518	3.589394	0.000331	0.001954
C1GALT1C1L	45.7930421	2.677703964	0.747076	3.584248	0.000338	0.001983
ABCG8	36.6645575	4.012358453	0.918046	4.370542	1.24E-05	0.000116
PPM1B	1465.29893	2.598146698	0.434135	5.984651	2.17E-09	4.55E-08
LINC01121	13.8172623	4.603767001	1.236903	3.722012	0.000198	0.001268
BCYRN1	927.533653	-2.169824443	0.510171	-4.25313	2.11E-05	0.000184
KCNK12	1270.52176	6.358003502	0.374916	16.95847	1.67E-64	1.38E-61
AC092650.1	27.6206253	3.913538918	1.018476	3.842545	0.000122	0.000839
LHCGR	16.714083	3.658680631	1.14325	3.200246	0.001373	0.0063
NRXN1	92.9445055	6.868462966	0.97688	7.031021	2.05E-12	6.67E-11
AC007402.2	13.0675337	4.51829105	1.242467	3.636549	0.000276	0.001677
EML6	156.170457	4.467769645	0.556714	8.02525	1.01E-15	4.97E-14
RTN4	22235.609	-2.478825609	0.312077	-7.94299	1.97E-15	9.32E-14
AC012358.2	17.9764301	3.450135368	1.095753	3.148644	0.00164	0.007298
LINC01122	36.1786647	3.730953192	0.888893	4.197301	2.70E-05	0.000227
AC007179.2	19.039004	5.153283093	1.197322	4.304007	1.68E-05	0.00015
MIR4432HG	23.698712	4.328305882	1.118052	3.871293	0.000108	0.000759
REL	66.1323585	4.769439866	0.830558	5.742455	9.33E-09	1.75E-07
FAM161A	940.686218	4.472291643	0.358143	12.48745	8.74E-36	1.74E-33
AC007098.1	70.4304367	2.212272824	0.634077	3.488965	0.000485	0.002663
AC012368.1	30.0159439	2.935679356	0.89925	3.264588	0.001096	0.005224
LINC00309	13.0031044	4.473281904	1.246584	3.588433	0.000333	0.001957
ACTR2	7245.33088	-2.29119043	0.324668	-7.05703	1.70E-12	5.60E-11
AC007389.1	44.5244799	3.479403822	0.903627	3.850485	0.000118	0.000819
AC007741.1	9.38331971	4.017150993	1.273395	3.154678	0.001607	0.00717
LINC01828	25.0960754	3.810832691	1.042243	3.656378	0.000256	0.00157
LINC02831	11.7613643	4.425453629	1.246312	3.550839	0.000384	0.002208
BMP10	17.293541	5.054323519	1.202013	4.204883	2.61E-05	0.000222
CLEC4F	74.3671049	5.741567215	0.828675	6.92861	4.25E-12	1.33E-10
CD207	43.9890043	4.178906526	0.87593	4.770825	1.83E-06	2.11E-05
LINC01143	40.7840391	6.504410031	1.090678	5.963637	2.47E-09	5.15E-08
AC007040.3	11.5270701	4.425460209	1.245845	3.552177	0.000382	0.002199
VAX2	681.314667	3.135390737	0.405404	7.733996	1.04E-14	4.45E-13

TEX261	1705.69637	-2.290090023	0.335871	-6.81836	9.21E-12	2.75E-10
DYSF	1728.44494	-4.904155162	0.482643	-10.161	2.96E-24	2.99E-22
CYP26B1	880.733302	4.850658808	0.381959	12.69941	5.96E-37	1.28E-34
EMX1	14.5406321	4.751032377	1.224537	3.879859	0.000105	0.000737
RAB11FIP5	2669.14801	-2.567892278	0.315245	-8.14571	3.77E-16	1.94E-14
C2orf78	10.2501954	3.994010754	1.278276	3.124529	0.001781	0.007777
ACTG2	120.921245	-3.037618705	0.635681	-4.77852	1.77E-06	2.04E-05
SLC4A5	334.36462	6.770793659	0.555888	12.18015	3.97E-34	7.26E-32
DCTN1-AS1	11.1287827	4.232433809	1.261651	3.354678	0.000795	0.004025
AC005041.1	14.7602789	4.784725864	1.221827	3.916041	9.00E-05	0.000647
C2orf81	35.9349189	3.169272514	0.829593	3.820275	0.000133	0.000908
HMGA1P8	34.6578537	-3.844035115	0.901142	-4.26574	1.99E-05	0.000175
HK2	362.474735	-2.897709111	0.489794	-5.91618	3.30E-09	6.69E-08
TACR1	16.1369961	3.731453725	1.172713	3.181898	0.001463	0.006621
AC005034.6	11.4392177	4.228541376	1.263141	3.347639	0.000815	0.004096
AC073091.1	13.4043276	4.355513885	1.258772	3.460129	0.00054	0.002922
RN7SKP203	43.3261086	6.402701031	1.11076	5.764253	8.20E-09	1.55E-07
AC068616.2	9.72011361	3.945326647	1.280246	3.081694	0.002058	0.008746
AC068616.3	12.0600878	4.135111753	1.272919	3.248527	0.00116	0.005468
LRRTM4	33.3104543	5.117112793	0.99009	5.168332	2.36E-07	3.38E-06
AC012494.1	12.4349521	4.180902917	1.270392	3.291035	0.000998	0.004829
REG1B	10.2501954	3.994010754	1.278276	3.124529	0.001781	0.007777
REG1CP	10.6250598	4.049542664	1.275176	3.175673	0.001495	0.006735
CTNNA2	39.2721149	3.246514434	0.885077	3.66806	0.000244	0.001511
AC008067.1	10.572342	4.23381264	1.259403	3.361761	0.000774	0.003938
ANKRD11P1	12.8742457	4.341881084	1.258229	3.450788	0.000559	0.003008
LINC01815	9.93976043	4.008663455	1.276073	3.141406	0.001681	0.007444
DNAH6	74.462078	4.584688804	0.740923	6.187806	6.10E-10	1.40E-08
CAPG	1365.95291	-3.014956912	0.387392	-7.7827	7.10E-15	3.08E-13
SFTPB	13.5595451	4.310027042	1.263134	3.412169	0.000644	0.00339
GNLY	12.1889464	4.326075881	1.257234	3.440947	0.00058	0.003092
CD8B	13.9724798	4.602774761	1.237603	3.719104	0.0002	0.00128
AC068279.2	21.554683	5.48617511	1.167293	4.699912	2.60E-06	2.88E-05
SMYD1	14.1394089	4.751532671	1.22287	3.885559	0.000102	0.000724
LSP1P5	182.966427	-2.013190905	0.485625	-4.14557	3.39E-05	0.000277
ANKRD20A8P	13.3252509	4.642504376	1.230987	3.771369	0.000162	0.001076
AC097374.1	31.4739561	5.871404382	1.150165	5.104835	3.31E-07	4.61E-06
AC009237.3	225.612572	-2.665831911	0.461352	-5.7783	7.55E-09	1.44E-07
ANKRD36C	156.82252	2.836353593	0.496529	5.712361	1.11E-08	2.06E-07
ADRA2B	18.1608225	3.869048282	1.110591	3.483775	0.000494	0.002706
FAM178B	27.1161544	5.804295843	1.145295	5.06795	4.02E-07	5.49E-06
GPAT2P2	8.69802037	3.906713832	1.279695	3.052849	0.002267	0.009467
VWA3B	46.2334956	4.643832106	0.92495	5.020629	5.15E-07	6.85E-06
MGAT4A	212.895531	2.771545076	0.451543	6.137943	8.36E-10	1.87E-08

LINC02611	15.691584	4.795258917	1.224373	3.916503	8.98E-05	0.000647
C2orf15	90.7487366	2.031188008	0.589993	3.442731	0.000576	0.003077
LINC01104	9.3188904	3.98167413	1.276003	3.120427	0.001806	0.007857
TBC1D8	134.216358	2.105236287	0.54086	3.892387	9.93E-05	0.000707
CREG2	20.6888974	3.100845808	1.020686	3.038002	0.002382	0.009855
RFX8	13.442398	4.562290166	1.239586	3.680494	0.000233	0.001452
IL1RL1	18.1665353	3.884717528	1.163365	3.339207	0.00084	0.004202
IL18R1	25.7221326	5.54706044	1.173418	4.727266	2.28E-06	2.55E-05
IL18RAP	11.3103591	4.03853363	1.278167	3.159629	0.00158	0.007069
LINC01965	12.4085932	4.379167019	1.253201	3.494385	0.000475	0.002618
LINC01102	26.3166437	5.592845043	1.169683	4.781503	1.74E-06	2.02E-05
AC013402.3	9.62932542	4.002668209	1.275446	3.138251	0.0017	0.0075
AC068057.3	18.6260693	5.004139642	1.212623	4.126708	3.68E-05	0.000298
LINC01114	14.5025617	4.638184454	1.236066	3.752377	0.000175	0.001146
PANTR1	22.2575655	5.388374503	1.181148	4.561979	5.07E-06	5.21E-05
POU3F3	22.9428648	5.404632069	1.181561	4.574146	4.78E-06	4.94E-05
LINC01159	11.2840002	4.232661173	1.262212	3.353368	0.000798	0.004039
FHL2	3202.69612	-4.526300757	0.321852	-14.0633	6.38E-45	2.21E-42
ECRG4	61.8851869	5.902722863	0.917373	6.434373	1.24E-10	3.17E-09
CD8B2	11.3747884	4.154235208	1.269217	3.273069	0.001064	0.00509
EEF1A1P12	53.9532219	3.880936855	0.763829	5.080899	3.76E-07	5.17E-06
ST6GAL2	312.229096	-3.106249319	0.4572	-6.79408	1.09E-11	3.23E-10
AC005040.2	22.7876473	5.408693913	1.180539	4.581547	4.62E-06	4.80E-05
LINC01885	9.56489611	3.948402431	1.27948	3.085944	0.002029	0.008665
SLC5A7	13.6884037	4.501752988	1.246408	3.611783	0.000304	0.001818
SULT1C2	807.255074	5.755857956	0.445929	12.90756	4.08E-38	9.41E-36
SULT1C2P1	12.9240277	4.622259838	1.2315	3.753358	0.000174	0.001143
EDAR	17.0094649	4.999090528	1.207185	4.141114	3.46E-05	0.000282
AC114776.1	13.8172623	4.603767001	1.236903	3.722012	0.000198	0.001268
MERTK	512.192629	4.629384625	0.449032	10.30969	6.37E-25	6.71E-23
NT5DC4	13.7264741	4.638510683	1.233011	3.761939	0.000169	0.001109
IL36RN	9.78454293	4.008050513	1.275572	3.14216	0.001677	0.007433
LINC01961	9.25446109	3.938692024	1.279145	3.07916	0.002076	0.008797
PGM5P4-AS1	56.7563442	2.245543425	0.687796	3.264839	0.001095	0.005222
FAM138B	20.3515389	3.682257025	1.062412	3.465939	0.000528	0.002867
DDX11L2	55.0660739	-2.826966367	0.718935	-3.93216	8.42E-05	0.000612
DPP10	378.789154	2.815942064	0.394404	7.139748	9.35E-13	3.19E-11
LINC01956	17.2525348	5.136611954	1.192617	4.30701	1.65E-05	0.000149
EN1	11.6325057	4.366275091	1.251409	3.489086	0.000485	0.002662
STEAP3	939.545428	-2.44818742	0.341231	-7.17458	7.25E-13	2.51E-11
CFAP221	88.0829748	3.55552321	0.631239	5.632612	1.78E-08	3.17E-07
TFCP2L1	23.2152294	5.22759377	1.202736	4.346419	1.38E-05	0.000127
LINC01823	12.4466636	4.49720804	1.242071	3.620732	0.000294	0.001764
AC011246.1	21.3145489	5.236732057	1.195789	4.37931	1.19E-05	0.000111

AC079154.1	14.4117735	4.684735938	1.230972	3.80572	0.000141	0.000956
CNTNAP5	22.7208077	3.784741083	1.066679	3.548155	0.000388	0.002229
LIMS2	1081.51111	-5.058096995	0.500484	-10.1064	5.17E-24	5.14E-22
AMMECR1L	653.238434	-2.070932735	0.356536	-5.80849	6.30E-09	1.21E-07
LINC01854	14.877426	4.676274107	1.233625	3.790678	0.00015	0.001004
AC079776.5	14.8134025	3.508512642	1.151312	3.047404	0.002308	0.009608
FAR2P1	9.44774903	4.046930126	1.271214	3.183517	0.001455	0.006587
SMPD4	1307.78386	-2.046912201	0.332127	-6.16305	7.14E-10	1.62E-08
AMER3	19.7506622	5.087485967	1.20733	4.213833	2.51E-05	0.000214
FAM168B	2672.97241	-2.198056652	0.314463	-6.98987	2.75E-12	8.79E-11
LINC01120	12.0337289	4.332064733	1.25612	3.448766	0.000563	0.003025
AC093838.1	16.312454	4.760908736	1.230198	3.870034	0.000109	0.000763
LINC01087	10.4434834	4.166374392	1.26483	3.29402	0.000988	0.004787
AC093787.2	22.3717767	5.544562505	1.162174	4.770856	1.83E-06	2.11E-05
AC093787.1	26.0296318	5.779147083	1.144911	5.047683	4.47E-07	6.05E-06
ANKRD30BL	25.5141973	5.658504042	1.158798	4.883082	1.04E-06	1.28E-05
AC097532.3	9.79625447	4.136713819	1.265037	3.270034	0.001075	0.005139
NCKAP5	448.318646	3.645736585	0.398993	9.137356	6.40E-20	4.76E-18
NCKAP5-AS1	13.1846808	4.268440808	1.265487	3.372963	0.000744	0.003804
MAP3K19	29.9744988	5.806670916	1.154065	5.031492	4.87E-07	6.52E-06
LCT	26.7048904	4.929157284	1.099406	4.483473	7.34E-06	7.24E-05
CXCR4	105.510573	2.143814964	0.571757	3.749519	0.000177	0.001157
THSD7B	36.2954059	4.008054647	0.965628	4.150722	3.31E-05	0.000272
HNMT	183.576336	2.472461394	0.471505	5.243763	1.57E-07	2.33E-06
KYNU	55.0300935	4.356942819	0.887331	4.910167	9.10E-07	1.13E-05
ARHGAP15	27.6345247	5.731163827	1.156507	4.955582	7.21E-07	9.25E-06
AC079793.1	18.0051992	5.030607371	1.207457	4.166283	3.10E-05	0.000256
AC092652.1	10.1330484	4.143717258	1.26561	3.274087	0.00106	0.005082
AC009951.4	10.8183477	4.222792645	1.261322	3.34791	0.000814	0.004096
AC009951.6	27.2279554	4.177156002	0.991238	4.214078	2.51E-05	0.000214
TEX41	79.091313	5.553305773	0.851644	6.520687	7.00E-11	1.86E-09
KIF5C-AS1	13.0031044	4.473281904	1.246584	3.588433	0.000333	0.001957
KIF5C	359.793086	4.401798524	0.449154	9.800196	1.12E-22	1.03E-20
MMADHC	2261.93724	-2.303903926	0.386714	-5.95764	2.56E-09	5.32E-08
RND3	4240.20094	-3.086660133	0.318969	-9.67698	3.78E-22	3.36E-20
RBM43	436.327558	2.331345431	0.430516	5.415231	6.12E-08	9.85E-07
TNFAIP6	12.5374518	4.470435694	1.245048	3.590572	0.00033	0.001948
NEB	151.496243	2.620368285	0.621421	4.216733	2.48E-05	0.000212
CACNB4	187.036998	4.292496485	0.521333	8.233688	1.82E-16	9.66E-15
AC061961.1	37.0299617	5.113406004	0.974683	5.246227	1.55E-07	2.30E-06
KCNJ3	280.50709	5.231173051	0.483404	10.82154	2.72E-27	3.34E-25
AC073225.1	37.6715976	4.516501449	0.95048	4.751809	2.02E-06	2.29E-05
AC096589.2	9.64103696	4.11173048	1.266623	3.246216	0.00117	0.005502
AC096589.1	11.7613643	4.425453629	1.246312	3.550839	0.000384	0.002208

ERMN	49.7769329	3.802525976	0.758611	5.012485	5.37E-07	7.12E-06
ACVR1C	53.4682359	3.270496099	0.752544	4.345918	1.39E-05	0.000128
UPP2	16.0241135	4.190202664	1.182594	3.543231	0.000395	0.002255
CCDC148	171.284632	2.790577421	0.48445	5.760302	8.40E-09	1.58E-07
PKP4-AS1	24.9140932	4.339307887	1.128296	3.845895	0.00012	0.000832
DAPL1	949.072408	7.042859777	0.529542	13.29991	2.32E-40	6.33E-38
CD302	87.5732055	3.21375744	0.606975	5.294707	1.19E-07	1.81E-06
AC009299.2	17.6570996	3.798669298	1.121521	3.387069	0.000706	0.003645
SLC4A10	346.51307	5.308317767	1.18641	4.474268	7.67E-06	7.53E-05
AC062022.2	11.0116356	4.317624376	1.253511	3.444425	0.000572	0.00306
DPP4	1501.20934	-2.606009839	0.32711	-7.96677	1.63E-15	7.79E-14
GCG	9.44774903	4.046930126	1.271214	3.183517	0.001455	0.006587
FAP	847.198373	-4.003832791	0.362967	-11.0309	2.71E-28	3.59E-26
IFIH1	232.857634	2.131962825	0.431489	4.940941	7.77E-07	9.88E-06
KCNH7-AS1	12.3822343	4.464991456	1.24496	3.586452	0.000335	0.001968
AC107075.1	42.7885174	3.838870559	0.902827	4.252054	2.12E-05	0.000184
AC019197.1	107.21407	7.914028219	0.999298	7.919586	2.38E-15	1.11E-13
RNA5SP111	14.6548434	4.855730572	1.214044	3.999633	6.34E-05	0.000479
SLC38A11	606.942353	6.72380667	0.519121	12.95228	2.28E-38	5.44E-36
SCN3A	29.3272699	5.864987305	1.144536	5.124338	2.99E-07	4.18E-06
CSRNP3	117.986405	2.856288143	0.58587	4.875291	1.09E-06	1.33E-05
SCN1A-AS1	41.2589048	5.185306086	0.988707	5.244532	1.57E-07	2.32E-06
SCN1A	58.8285187	4.520510742	0.840857	5.376074	7.61E-08	1.20E-06
SCN7A	28.0799368	4.670634626	1.071915	4.35728	1.32E-05	0.000122
XIRP2	40.2688832	5.222577111	1.023594	5.102197	3.36E-07	4.67E-06
AC073050.1	19.7740853	5.338171529	1.178076	4.531262	5.86E-06	5.92E-05
B3GALT1	134.090573	2.946421485	0.526708	5.594028	2.22E-08	3.87E-07
CERS6	365.439251	2.14977298	0.464796	4.625199	3.74E-06	3.98E-05
NOSTRIN	26.4718612	5.585963372	1.171011	4.770207	1.84E-06	2.11E-05
ABCB11	24.158246	5.428568905	1.182707	4.589951	4.43E-06	4.63E-05
LRP2	192.068115	6.197987233	0.640439	9.677716	3.75E-22	3.34E-20
CCDC173	54.2847197	2.732032983	0.849102	3.217557	0.001293	0.005996
MYO3B	57.2522447	3.943473439	0.761529	5.178364	2.24E-07	3.22E-06
MYO3B-AS1	28.1909654	5.685674534	1.16396	4.884766	1.04E-06	1.27E-05
AC007405.3	45.8910941	2.715030408	0.746855	3.635284	0.000278	0.001682
RAPGEF4-AS1	15.5627253	4.692214931	1.234504	3.800889	0.000144	0.00097
RAPGEF4	61.8303325	2.297047192	0.69469	3.30658	0.000944	0.004646
EVX2	11.5300059	4.129467712	1.271744	3.247089	0.001166	0.005489
HOXD13	11.4128589	4.32259367	1.254608	3.445374	0.00057	0.003052
AC092162.2	15.3167196	4.759284449	1.226727	3.879662	0.000105	0.000737
AC073636.1	14.373703	4.505843839	1.248403	3.609286	0.000307	0.001832
AC074286.1	18.7168575	4.92681021	1.221153	4.034555	5.47E-05	0.000421
PDE11A	74.6894139	2.434585218	0.643811	3.781523	0.000156	0.001037
TTN-AS1	56.3210679	3.027787313	0.837325	3.616023	0.000299	0.001793

TTN	331.976434	4.273466691	0.59241	7.213693	5.45E-13	1.92E-11
CCDC141	62.4669451	3.936670826	0.745216	5.282587	1.27E-07	1.92E-06
ZNF385B	26.2491038	3.184304484	1.020827	3.119338	0.001813	0.007881
LINC01934	37.7219443	3.761138848	1.004224	3.745317	0.00018	0.001173
CERKL	18.418134	5.148149712	1.195544	4.306116	1.66E-05	0.000149
PDE1A	50.7162106	3.422172549	0.734775	4.657442	3.20E-06	3.47E-05
FRZB	328.300271	5.646000261	0.49825	11.33165	9.15E-30	1.29E-27
KRT8P10	48.8979778	-3.693509173	0.889799	-4.15095	3.31E-05	0.000271
AC021851.1	10.0422602	4.156981159	1.264143	3.288379	0.001008	0.004862
AC096667.1	53.476981	2.729089366	0.700317	3.896936	9.74E-05	0.000695
FSIP2	70.0573909	4.347519097	0.782225	5.55789	2.73E-08	4.70E-07
GULP1	1019.8024	2.768473688	0.383727	7.214696	5.41E-13	1.91E-11
C2orf88	41.8037783	2.757151138	0.786877	3.503918	0.000458	0.002557
AC108047.1	37.4758127	2.947282933	0.816571	3.609339	0.000307	0.001832
AC006460.1	22.2373251	4.323908078	1.101307	3.92616	8.63E-05	0.000626
GLS	3730.24856	-2.447529399	0.318859	-7.67589	1.64E-14	6.88E-13
STAT4	45.4227844	2.586783176	0.751615	3.441634	0.000578	0.003088
MYO1B	1109.03668	-2.046932671	0.345706	-5.92101	3.20E-09	6.52E-08
NABP1	3706.61248	-4.128352856	0.328421	-12.5703	3.08E-36	6.28E-34
CAVIN2	150.82899	-3.210094018	0.511365	-6.2775	3.44E-10	8.14E-09
CAVIN2-AS1	13.9461209	4.675279624	1.230098	3.800737	0.000144	0.000971
LINC01821	17.4106881	4.965599823	1.212466	4.095453	4.21E-05	0.000335
DNAH7	132.34505	4.60377027	0.614318	7.494117	6.67E-14	2.63E-12
HECW2	68.7460514	2.819339221	0.784989	3.591563	0.000329	0.001942
PGAP1	580.000283	2.907059906	0.367683	7.906438	2.65E-15	1.22E-13
BOLL	16.6026487	3.699605693	1.186477	3.118144	0.00182	0.007912
PLCL1	43.4364698	3.53039986	0.822099	4.294372	1.75E-05	0.000156
LINC01877	28.7210472	5.695808823	1.164276	4.892146	9.97E-07	1.23E-05
AC097717.1	21.3968083	4.290690723	1.099454	3.902564	9.52E-05	0.00068
AOX1	425.844197	-3.5104765	0.545149	-6.43948	1.20E-10	3.07E-09
C2CD6	30.94043	2.732118572	0.897784	3.043179	0.002341	0.009719
AC079354.2	9.47410791	3.993729441	1.275601	3.130862	0.001743	0.007649
ICA1L	126.273943	2.325502992	0.519886	4.473099	7.71E-06	7.56E-05
ICOS	14.2829148	4.592011422	1.239841	3.703711	0.000212	0.001343
GPR1	140.34957	-2.224726247	0.519793	-4.28002	1.87E-05	0.000165
ADAM23	786.934971	-2.071917873	0.357756	-5.79143	6.98E-09	1.34E-07
DYTN	11.8521525	4.408207058	1.248315	3.531327	0.000413	0.002339
LINC01857	20.4710962	5.419958231	1.170802	4.629271	3.67E-06	3.92E-05
PPP1R14BP2	33.0989318	-4.13885789	0.971885	-4.25859	2.06E-05	0.00018
PTH2R	18.4708518	5.013976713	1.211002	4.140355	3.47E-05	0.000283
UNC80	182.672683	3.538081217	0.509599	6.942874	3.84E-12	1.21E-10
AC006994.1	20.5384613	5.262181298	1.190099	4.421634	9.80E-06	9.35E-05
CPS1	193.460166	2.331875039	0.508362	4.587034	4.50E-06	4.69E-05
ERBB4	199.35493	4.38517709	0.495683	8.846743	9.01E-19	5.92E-17

AC108066.2	14.877426	4.676274107	1.233625	3.790678	0.00015	0.001004
AC079610.1	20.91732	3.137700933	1.017002	3.085244	0.002034	0.008681
AC068051.1	13.0938926	4.404207729	1.253385	3.513852	0.000442	0.002474
VWC2L	15.3167196	4.759284449	1.226727	3.879662	0.000105	0.000737
BARD1	218.512569	2.507591594	0.442823	5.662743	1.49E-08	2.69E-07
RPL10P6	127.746579	2.082689895	0.605261	3.440977	0.00058	0.003092
FN1	44282.6054	-5.42388106	0.309549	-17.5219	9.75E-69	8.70E-66
LINC01963	300.46141	-2.262034792	0.422572	-5.35302	8.65E-08	1.35E-06
IGFBP5	8431.17542	4.250322848	0.34656	12.26432	1.41E-34	2.65E-32
DIRC3-AS1	22.2287963	3.795008524	1.052933	3.604226	0.000313	0.001864
TNS1	1582.28365	-2.141818528	0.365159	-5.86544	4.48E-09	8.86E-08
RUFY4	16.5965301	4.858850005	1.221017	3.979348	6.91E-05	0.000515
TMBIM1	2851.00891	-2.407011226	0.318294	-7.56223	3.96E-14	1.61E-12
SLC11A1	40.4145379	3.160297524	0.847668	3.728227	0.000193	0.001244
CYP27A1	5629.01558	4.660593191	0.381012	12.23214	2.09E-34	3.88E-32
AC009974.2	12.4466636	4.49720804	1.242071	3.620732	0.000294	0.001764
AC009974.1	31.7934138	4.976225916	1.023759	4.86074	1.17E-06	1.41E-05
WNT10A	132.871742	8.192905367	0.986214	8.307428	9.78E-17	5.28E-15
LINC00608	12.4993814	4.297396672	1.260864	3.408295	0.000654	0.003427
AC097468.1	10.0949779	4.005107431	1.276891	3.136609	0.001709	0.007522
CFAP65	56.5477867	3.964122045	0.809085	4.899514	9.61E-07	1.19E-05
ATG9A	561.093033	-3.400762492	0.413076	-8.23277	1.83E-16	9.72E-15
SPEG	9691.56983	-2.130410053	0.313143	-6.80332	1.02E-11	3.04E-10
ASIC4	23.6889182	3.709164885	0.98534	3.764348	0.000167	0.001101
INHA	96.299555	-3.105396401	0.589846	-5.26476	1.40E-07	2.10E-06
SLC4A3	2116.43927	-2.900233933	0.330234	-8.78236	1.60E-18	1.03E-16
AC009955.2	35.9225022	-4.100860346	0.91301	-4.49158	7.07E-06	7.00E-05
PAX3	23.4348762	5.273178239	1.198353	4.400356	1.08E-05	0.000102
CCDC140	10.3146248	4.067189627	1.272691	3.195739	0.001395	0.006369
AP1S3	1077.93815	-2.85520255	0.360569	-7.9186	2.40E-15	1.11E-13
SERPINE2	558.48065	-2.003147404	0.397632	-5.03769	4.71E-07	6.33E-06
FAM124B	15.6069143	3.699733032	1.172615	3.155112	0.001604	0.007166
C2orf83	11.3484295	4.282003863	1.258062	3.403651	0.000665	0.003466
SLC19A3	35.6278169	4.04181265	0.885223	4.565869	4.97E-06	5.12E-05
AC073065.1	10.6894891	4.123736642	1.269366	3.248658	0.00116	0.005468
SPHKAP	29.2247702	5.773003239	1.156107	4.993486	5.93E-07	7.77E-06
SLC16A14	948.834748	5.825336111	0.492414	11.83015	2.73E-32	4.42E-30
SP140L	198.829153	-2.877358753	0.489929	-5.87301	4.28E-09	8.50E-08
GCSIR	22.4772123	5.413998552	1.17881	4.592764	4.37E-06	4.58E-05
RPS28P4	370.412556	7.178029722	0.58619	12.24523	1.78E-34	3.32E-32
NMUR1	74.7736742	7.410646874	1.02877	7.203405	5.87E-13	2.06E-11
PTMA	103776.462	2.736379826	0.303298	9.022081	1.85E-19	1.30E-17
ALPG	14.7866378	4.723776122	1.228387	3.845512	0.00012	0.000832
ALPI	12.8859573	4.572223503	1.236371	3.698098	0.000217	0.00137

CHRND	12.1625875	4.422982886	1.248134	3.543676	0.000395	0.002253
CHRNG	13.9724798	4.602774761	1.237603	3.719104	0.0002	0.00128
KCNJ13	73.2759207	4.479850987	0.729489	6.14108	8.20E-10	1.84E-08
SAG	42.7757864	5.357780951	1.00233	5.345325	9.03E-08	1.40E-06
UGT1A7	10.9091359	4.179411771	1.265451	3.302706	0.000958	0.004688
ARL4C	3276.69713	-2.085418994	0.316529	-6.5884	4.45E-11	1.22E-09
SH3BP4	4348.69642	-2.505656794	0.317873	-7.88258	3.21E-15	1.46E-13
ASB18	15.0326435	4.668138074	1.235019	3.779812	0.000157	0.001042
ACKR3	856.250629	-4.840407375	0.372717	-12.9868	1.45E-38	3.54E-36
AC012063.1	49.1745468	2.601812236	0.782284	3.325918	0.000881	0.004375
AC105760.1	10.4434834	4.166374392	1.26483	3.29402	0.000988	0.004787
COL6A3	177.931175	4.025066136	0.556632	7.231109	4.79E-13	1.71E-11
RAB17	612.174397	-4.556889362	0.503967	-9.04204	1.54E-19	1.09E-17
LRRFIP1	3600.17809	-2.273232614	0.320649	-7.08946	1.35E-12	4.49E-11
ERFE	66.8393234	-2.318474409	0.664624	-3.4884	0.000486	0.002668
AC023787.3	12.4085932	4.379167019	1.253201	3.494385	0.000475	0.002618
AC079612.1	11.6852234	4.08769102	1.275511	3.204748	0.001352	0.006218
AC093802.1	15.3811489	4.797622762	1.222922	3.923082	8.74E-05	0.000633
GPC1	6905.33328	-2.694419236	0.314647	-8.56332	1.10E-17	6.47E-16
AGXT	12.6018811	4.505303377	1.241896	3.627762	0.000286	0.001725
CROCC2	25.2301212	5.616573932	1.16317	4.828679	1.37E-06	1.64E-05
UICLM	16.8776705	5.098804711	1.195564	4.264769	2.00E-05	0.000175
BOK	2375.82389	-2.612593077	0.325674	-8.0221	1.04E-15	5.09E-14
D2HGDH	1079.05004	-2.357356652	0.375716	-6.2743	3.51E-10	8.30E-09
RTP5	11.696935	4.398045908	1.248667	3.522193	0.000428	0.002409
AC093642.3	15.0768325	3.663598252	1.173337	3.122375	0.001794	0.007828
AC093642.6	108.277138	7.889610717	1.004966	7.850623	4.14E-15	1.84E-13
LINC01880	573.652831	5.063384778	1.244382	4.068994	4.72E-05	0.000371
LINC01881	250.443326	3.452880105	0.449811	7.676285	1.64E-14	6.87E-13
CHL1-AS2	10.0949779	4.005107431	1.276891	3.136609	0.001709	0.007522
AC066595.2	11.3484295	4.282003863	1.258062	3.403651	0.000665	0.003466
CHL1	35.5711048	6.084793251	1.133804	5.366706	8.02E-08	1.26E-06
LINC01266	67.6399804	2.66878065	0.657624	4.058218	4.94E-05	0.000386
AC087430.1	10.5079127	4.202889647	1.26189	3.330629	0.000866	0.004313
CNTN4	63.6522615	3.013020984	0.706117	4.267025	1.98E-05	0.000174
IL5RA	26.24795	5.016247005	1.105815	4.536242	5.73E-06	5.80E-05
PNPT1P1	10.0041897	4.063829218	1.271846	3.195222	0.001397	0.006372
BHLHE40-AS1	65.4806513	2.864502007	0.665633	4.303425	1.68E-05	0.000151
AC069277.1	21.8888197	4.244258073	1.118858	3.793384	0.000149	0.000995
GRM7-AS3	9.72011361	3.945326647	1.280246	3.081694	0.002058	0.008746
GRM7	31.1315692	4.673895728	1.100832	4.245785	2.18E-05	0.000189
AC077690.1	16.2922136	3.724781024	1.176507	3.165966	0.001546	0.006937
LMCD1-AS1	46.0522223	2.695184489	0.796475	3.383891	0.000715	0.003679
AC023481.1	10.8447066	4.116553698	1.270511	3.240076	0.001195	0.005595

OR7E122P	14.1276973	4.598912982	1.238579	3.713055	0.000205	0.001303
OXTR	1808.57882	-4.429516995	0.438105	-10.1106	4.96E-24	4.94E-22
AC037193.1	9.62932542	4.002668209	1.275446	3.138251	0.0017	0.0075
LHFPL4	17.2174002	4.806991807	1.228583	3.91263	9.13E-05	0.000656
CIDEC	9.69375473	4.046019533	1.27217	3.180407	0.001471	0.006648
ATP2B2	111.38152	7.923992203	1.002765	7.902142	2.74E-15	1.26E-13
SLC6A11	23.0604177	4.205280939	1.086281	3.871263	0.000108	0.000759
SLC6A1	53.1223631	6.763966146	1.082367	6.249236	4.12E-10	9.70E-09
SYN2	68.5710545	3.13785035	0.660694	4.749325	2.04E-06	2.32E-05
TIMP4	45.5582309	2.359054299	0.764448	3.085957	0.002029	0.008665
KRT18P17	173.059332	-4.241549891	0.541214	-7.8371	4.61E-15	2.04E-13
WNT7A	12.4993814	4.297396672	1.260864	3.408295	0.000654	0.003427
FGD5P1	11.0116356	4.317624376	1.253511	3.444425	0.000572	0.00306
XPC	817.728595	-2.114078572	0.365798	-5.77935	7.50E-09	1.43E-07
LINC01267	9.44774903	4.046930126	1.271214	3.183517	0.001455	0.006587
FGD5	18.2046057	3.996491304	1.135416	3.519848	0.000432	0.002427
COL6A4P1	12.00737	4.41656174	1.248133	3.538534	0.000402	0.002286
RN7SL4P	125.502193	4.548716384	0.653755	6.957832	3.46E-12	1.09E-10
RFTN1	928.451711	-2.347320676	0.349633	-6.71368	1.90E-11	5.48E-10
DAZL	18.7549279	5.089643378	1.203574	4.228774	2.35E-05	0.000202
SATB1-AS1	94.6126032	4.90834409	0.662108	7.413208	1.23E-13	4.74E-12
KCNH8	41.1138808	3.329298865	0.925578	3.596993	0.000322	0.001911
AC061958.1	10.0949779	4.005107431	1.276891	3.136609	0.001709	0.007522
SGO1-AS1	16.6877241	3.700771305	1.132239	3.268543	0.001081	0.005164
AC116096.1	10.9735652	4.22904493	1.261362	3.35276	0.0008	0.00404
ZNF385D	308.285338	3.910915323	0.431366	9.066355	1.23E-19	8.85E-18
RPL24P7	25.1429215	3.988916313	0.972454	4.101906	4.10E-05	0.000328
UBE2E1	472.739266	-2.193199461	0.378814	-5.78964	7.05E-09	1.35E-07
LINC00691	9.78454293	4.008050513	1.275572	3.14216	0.001677	0.007433
THRB	224.550028	2.639786999	0.443087	5.957714	2.56E-09	5.32E-08
THRB-AS1	23.7570228	5.479333358	1.175261	4.662227	3.13E-06	3.40E-05
AC099754.1	11.5680764	4.328984445	1.254624	3.450423	0.00056	0.003011
LRRC3B	21.4170486	5.367116452	1.180708	4.545677	5.48E-06	5.57E-05
EOMES	18.1401764	3.968443119	1.141889	3.475331	0.00051	0.002783
LINC01980	27.6349305	4.578080166	1.036427	4.417174	1.00E-05	9.52E-05
ZCWPW2	50.2424106	3.142097168	0.748827	4.196025	2.72E-05	0.000228
RBMS3-AS3	30.142765	3.437363786	0.924559	3.717841	0.000201	0.001284
GADL1	30.6978686	5.891721157	1.145265	5.144416	2.68E-07	3.80E-06
CCR4	10.7539184	4.178531017	1.264949	3.303319	0.000955	0.004683
GLB1	2498.53012	-2.089744602	0.325782	-6.41455	1.41E-10	3.58E-09
LINC01811	40.7503699	2.838723213	0.864618	3.283212	0.001026	0.004939
ARPP21	51.3409393	3.699815971	0.764567	4.8391	1.30E-06	1.56E-05
ITGA9	37.944885	5.626703567	1.049265	5.36252	8.21E-08	1.29E-06
ITGA9-AS1	84.2638314	3.144655591	0.664497	4.732384	2.22E-06	2.50E-05

DLEC1	47.416997	3.310128892	0.818396	4.044654	5.24E-05	0.000406
MYD88	545.752072	-2.610821516	0.369479	-7.06623	1.59E-12	5.26E-11
SCN10A	28.8499058	5.755780303	1.157158	4.974068	6.56E-07	8.50E-06
SCN11A	48.4438716	3.902519333	0.90727	4.301386	1.70E-05	0.000152
MOBP	26.4835728	5.718093014	1.154416	4.953235	7.30E-07	9.34E-06
MYRIP	803.5392	7.305484897	0.458787	15.92347	4.36E-57	3.01E-54
EIF1B-AS1	65.6294007	2.282269704	0.734227	3.1084	0.001881	0.008129
ENTPD3-AS1	121.710288	2.380415173	0.527909	4.509143	6.51E-06	6.50E-05
ULK4	320.233127	2.730601058	0.419483	6.509451	7.54E-11	1.99E-09
CCK	182.283297	3.302180016	0.59927	5.510338	3.58E-08	6.02E-07
VIPR1-AS1	14.4381324	4.581379308	1.241445	3.69036	0.000224	0.001405
HHATL	12.3822343	4.464991456	1.24496	3.586452	0.000335	0.001968
CYP8B1	9.47410791	3.993729441	1.275601	3.130862	0.001743	0.007649
TOPAZ1	18.107699	5.140304058	1.19525	4.300611	1.70E-05	0.000152
CDCP1	1849.14681	-5.515135284	0.416304	-13.2479	4.64E-40	1.25E-37
TMEM158	967.699234	-2.184557835	0.354608	-6.16049	7.25E-10	1.64E-08
LIMD1	1763.10171	2.638801793	0.33296	7.925286	2.28E-15	1.06E-13
AC098476.1	20.8459605	5.448809337	1.168621	4.662599	3.12E-06	3.40E-05
SLC6A20	1332.56856	11.19758385	0.868931	12.88662	5.35E-38	1.22E-35
XCR1	12.4085932	4.379167019	1.253201	3.494385	0.000475	0.002618
CCR3	11.2195709	4.169021091	1.267451	3.289296	0.001004	0.00485
CCR2	9.87533112	3.936139891	1.281456	3.071616	0.002129	0.008981
ALS2CL	392.10944	-2.58719636	0.445195	-5.81138	6.20E-09	1.20E-07
CSPG5	1326.29021	5.016378818	0.358523	13.99178	1.75E-44	5.87E-42
MAP4	9246.5043	-2.364146232	0.32053	-7.37575	1.63E-13	6.16E-12
TMA7	1475.30509	2.019956222	0.430453	4.692626	2.70E-06	2.97E-05
PFKFB4	452.68047	-2.161677629	0.378561	-5.71024	1.13E-08	2.08E-07
IHO1	49.2017087	2.897070286	0.729621	3.970649	7.17E-05	0.000532
RNF123	882.26478	-2.032034676	0.348038	-5.83854	5.27E-09	1.03E-07
CAMKV	78.3355539	5.801578163	0.784507	7.395189	1.41E-13	5.39E-12
SEMA3F	2224.20609	-3.352957979	0.321572	-10.4268	1.87E-25	2.03E-23
SLC38A3	136.078222	2.908875966	0.521167	5.581462	2.39E-08	4.13E-07
GNAI2	12064.5237	-2.423325738	0.359181	-6.74681	1.51E-11	4.42E-10
HYAL1	72.3891334	2.118947949	0.628409	3.371927	0.000746	0.003817
HYAL2	1881.34131	-2.180142284	0.351359	-6.20488	5.47E-10	1.26E-08
GRM2	21.2679496	4.248920198	1.109485	3.829632	0.000128	0.000879
AC115284.2	217.215927	2.969544777	0.563597	5.268919	1.37E-07	2.06E-06
GPR62	158.394211	3.116321145	0.567457	5.49173	3.98E-08	6.65E-07
ACY1	136.435535	2.90918774	0.520211	5.592321	2.24E-08	3.90E-07
STAB1	44.1712651	3.539720845	0.926158	3.821943	0.000132	0.000903
NT5DC2	4271.6726	-2.151055008	0.321215	-6.69663	2.13E-11	6.14E-10
ITIH1	13.8306447	3.893117621	1.185589	3.283698	0.001025	0.004931
SFMBT1	151.048028	2.55990669	0.533438	4.79888	1.60E-06	1.87E-05
AC115282.2	9.87533112	3.936139891	1.281456	3.071616	0.002129	0.008981

DNAH12	50.9710152	4.260850421	0.886201	4.807995	1.52E-06	1.80E-05
FLNB	7575.10142	-2.167159792	0.318838	-6.79706	1.07E-11	3.16E-10
DNASE1L3	16.9069651	4.840474567	1.224076	3.954391	7.67E-05	0.000564
ACOX2	290.697154	-2.147272139	0.423127	-5.07477	3.88E-07	5.31E-06
FAM107A	74.630574	6.267535686	0.872634	7.182321	6.85E-13	2.38E-11
CFAP20DC	67.9842691	2.320245127	0.675396	3.435383	0.000592	0.003145
CFAP20DC-AS1	14.3271038	3.586344654	1.180276	3.038564	0.002377	0.009841
AC126121.3	16.0664483	4.83022072	1.222086	3.95244	7.74E-05	0.000568
FEZF2	10.4698423	4.061968818	1.273658	3.189215	0.001427	0.006475
SNTN	14.6314203	4.722293368	1.227933	3.845727	0.00012	0.000832
PRICKLE2-AS1	11.6852234	4.08769102	1.275511	3.204748	0.001352	0.006218
ADAMTS9-AS1	29.6861583	5.208729535	1.089474	4.78096	1.74E-06	2.02E-05
TAFA1	12.9767455	4.551258675	1.238839	3.673809	0.000239	0.001482
TAFA4	41.3846688	5.465590494	0.95591	5.717682	1.08E-08	2.00E-07
EOGT	343.876145	-2.572657952	0.40109	-6.41417	1.42E-10	3.58E-09
SAMMSON	42.2659449	6.385429304	1.110547	5.749805	8.93E-09	1.68E-07
MDFIC2	11.7496528	4.203681987	1.266356	3.31951	0.000902	0.004457
EIF4E3	550.248883	2.895426821	0.389693	7.430013	1.09E-13	4.21E-12
GPR27	155.796545	2.599977169	0.538458	4.828564	1.38E-06	1.64E-05
LINC00877	41.0523067	3.638425306	0.84274	4.317377	1.58E-05	0.000143
LINC00870	32.4611617	4.936332153	1.038987	4.751101	2.02E-06	2.30E-05
ROBO2	183.097968	7.669649962	0.798821	9.601213	7.90E-22	6.83E-20
LINC02027	12.8478869	4.474722687	1.24585	3.591704	0.000329	0.001942
AC129807.1	13.9988387	4.466753428	1.25076	3.571231	0.000355	0.002065
LINC02008	20.6028906	5.290566444	1.186956	4.457255	8.30E-06	8.07E-05
LINC00971	26.5362906	5.617956939	1.167274	4.812888	1.49E-06	1.76E-05
CADM2	105.416107	2.867528781	0.580788	4.937311	7.92E-07	1.01E-05
CSNKA2IP	14.4117735	4.684735938	1.230972	3.80572	0.000141	0.000956
EPHA3	116.91046	4.674694753	0.614056	7.61281	2.68E-14	1.10E-12
PROS2P	12.2797346	4.224539264	1.26635	3.335996	0.00085	0.004242
LINC00879	17.1646824	5.001603559	1.207508	4.142088	3.44E-05	0.000281
AC109782.1	11.3484295	4.282003863	1.258062	3.403651	0.000665	0.003466
EPHA6	29.6117518	4.724164716	1.016181	4.648939	3.34E-06	3.60E-05
GABRR3	11.4128589	4.32259367	1.254608	3.445374	0.00057	0.003052
OR5H1	15.6007958	4.833628509	1.219919	3.962255	7.42E-05	0.000548
OR5H14	15.5100076	4.860577653	1.216627	3.995124	6.47E-05	0.000485
OR5K1	10.6250598	4.049542664	1.275176	3.175673	0.001495	0.006735
LINC00973	166.247118	-4.659543636	0.554067	-8.40971	4.11E-17	2.28E-15
AC107029.2	9.25446109	3.938692024	1.279145	3.07916	0.002076	0.008797
FILIP1L	1593.90053	-3.03571528	0.334667	-9.07087	1.18E-19	8.51E-18
LNP1	79.2060439	2.445347184	0.657467	3.719345	0.0002	0.00128
ADGRG7	25.5229731	5.782663023	1.143151	5.058528	4.23E-07	5.74E-06
ABI3BP	122.503931	4.309664274	0.593816	7.25757	3.94E-13	1.42E-11
NFKBIZ	689.317909	-2.486522204	0.384492	-6.46703	9.99E-11	2.58E-09

AC063938.1	9.69375473	4.046019533	1.27217	3.180407	0.001471	0.006648
LINC00882	110.783284	2.035964778	0.617853	3.295225	0.000983	0.004779
LINC00635	40.1983354	6.277109677	1.120161	5.603755	2.10E-08	3.69E-07
MORC1	19.2205804	5.070910042	1.207347	4.200045	2.67E-05	0.000225
DPPA2	9.62932542	4.002668209	1.275446	3.138251	0.0017	0.0075
LINC01205	21.4316959	4.967355779	1.224853	4.05547	5.00E-05	0.00039
CD96	31.5449098	4.2434573	0.97585	4.348471	1.37E-05	0.000126
ZBED2	66.5134404	-3.37209044	0.70826	-4.76109	1.93E-06	2.20E-05
PHLDB2	3669.56632	-3.344024667	0.318706	-10.4925	9.35E-26	1.02E-23
CD200	218.431878	-3.079176527	0.466764	-6.59686	4.20E-11	1.16E-09
SIDT1	29.1603408	5.744739437	1.159479	4.954585	7.25E-07	9.29E-06
GRAMD1C	41.7676732	3.136501234	0.817038	3.83887	0.000124	0.000851
DRD3	13.8172623	4.603767001	1.236903	3.722012	0.000198	0.001268
TIGIT	13.3135394	4.460011267	1.248997	3.570873	0.000356	0.002065
ZBTB20-AS1	17.4812359	3.868397134	1.15815	3.340153	0.000837	0.004189
AC026341.1	23.4210013	3.491749083	1.019427	3.425209	0.000614	0.003246
AC026341.3	11.3865	4.372633274	1.249852	3.498521	0.000468	0.00259
GAP43	81.1921188	3.315937403	0.649111	5.108429	3.25E-07	4.53E-06
LSAMP	1012.04963	5.851626522	0.389559	15.02117	5.34E-51	2.83E-48
IGSF11	20.014498	4.113712093	1.129525	3.641983	0.000271	0.001647
UPK1B	57.2480207	-2.767257831	0.800412	-3.45729	0.000546	0.00295
CD80	22.4244945	5.49816096	1.16819	4.706563	2.52E-06	2.80E-05
PLA1A	118.193475	8.044174897	0.992871	8.101936	5.41E-16	2.73E-14
COX17	1606.97071	2.014875933	0.377747	5.333927	9.61E-08	1.48E-06
CFAP91	70.9233037	5.250605833	0.805344	6.519703	7.04E-11	1.87E-09
NR1I2	15.5363665	4.79754536	1.223534	3.921056	8.82E-05	0.000637
FSTL1	17812.0765	-2.628482145	0.301721	-8.71163	3.00E-18	1.87E-16
HGD	15.6186258	3.821095944	1.141897	3.34627	0.000819	0.004112
STXBP5L	36.1570872	5.144369108	1.010753	5.089641	3.59E-07	4.95E-06
ARGFX	18.8457161	5.042775528	1.209151	4.170509	3.04E-05	0.000252
FBXO40	16.3768833	4.818032405	1.224528	3.934605	8.33E-05	0.000606
ILDR1	12.6663105	4.53535658	1.23918	3.659967	0.000252	0.001551
CD86	10.7802773	4.027023489	1.277437	3.152423	0.001619	0.007214
CASR	29.0051233	5.75053486	1.158289	4.964682	6.88E-07	8.87E-06
PARP15	25.8105105	3.949919991	1.058386	3.732024	0.00019	0.001228
SEMA5B	37.4261175	3.712337297	0.97533	3.806238	0.000141	0.000955
ROPN1	10.3146248	4.067189627	1.272691	3.195739	0.001395	0.006369
ITGB5	10292.4238	-4.004629064	0.309852	-12.9243	3.28E-38	7.62E-36
MUC13	11.0643534	4.176955156	1.266227	3.298742	0.000971	0.004726
HEG1	5063.65713	-2.002961178	0.316928	-6.31993	2.62E-10	6.34E-09
SLC12A8	646.788186	-3.756369205	0.412548	-9.1053	8.60E-20	6.28E-18
ROPN1B	12.62824	4.428525514	1.249417	3.544475	0.000393	0.00225
ALDH1L1	54.7078272	4.64886623	0.852231	5.454937	4.90E-08	8.04E-07
KLF15	66.049757	3.541116759	0.740635	4.781193	1.74E-06	2.02E-05

CFAP100	22.7293365	4.250822242	1.127252	3.770959	0.000163	0.001077
C3orf22	16.1572365	4.774355185	1.228275	3.887041	0.000101	0.000721
LINC01471	26.1614262	5.59867	1.168475	4.791435	1.66E-06	1.93E-05
KBTBD12	31.7874236	3.278101367	0.882239	3.71566	0.000203	0.001294
SEC61A1	9637.05578	-2.210906332	0.341356	-6.47684	9.37E-11	2.43E-09
EFCC1	12.7190282	4.360408778	1.25604	3.471552	0.000517	0.002815
RAB43	97.5426665	-2.609845002	0.612473	-4.26116	2.03E-05	0.000178
RHO	10.2501954	3.994010754	1.278276	3.124529	0.001781	0.007777
COL6A4P2	32.391951	3.545668429	0.914809	3.875859	0.000106	0.000747
COL6A5	33.9955067	5.822823595	1.162805	5.007567	5.51E-07	7.28E-06
NUDT16P1	59.0137974	2.403285179	0.669955	3.587236	0.000334	0.001965
NPHP3-AS1	17.6364534	3.862700594	1.161702	3.325035	0.000884	0.004388
BFSP2	10.6250598	4.049542664	1.275176	3.175673	0.001495	0.006735
TF	908.21841	6.946141173	0.487396	14.25153	4.39E-46	1.63E-43
SLCO2A1	20.1255266	5.114027585	1.205651	4.241713	2.22E-05	0.000192
AMOTL2	6705.58102	-3.97539438	0.34366	-11.5678	6.00E-31	8.88E-29
KY	72.4035778	5.828462761	0.844121	6.904773	5.03E-12	1.55E-10
AC092969.1	25.9215391	4.52526237	1.089471	4.153634	3.27E-05	0.000269
NME9	47.1517936	2.318530982	0.762337	3.041346	0.002355	0.009769
LINC01391	14.6577792	4.630645934	1.237395	3.742253	0.000182	0.001185
AC110716.2	10.9999241	4.102900639	1.272157	3.225153	0.001259	0.005856
CLSTN2	8366.78149	-2.550484752	0.336432	-7.58098	3.43E-14	1.39E-12
SPSB4	19.1034333	5.181849806	1.194228	4.339081	1.43E-05	0.000131
AC010184.1	17.5659056	4.960923812	1.213556	4.087923	4.35E-05	0.000345
PLS1	691.751105	2.11049144	0.40991	5.148675	2.62E-07	3.72E-06
AC072028.1	10.5342716	4.124847874	1.268711	3.25121	0.001149	0.005437
AC021074.3	17.7211231	4.954259176	1.214849	4.078087	4.54E-05	0.000359
PAQR9	133.061506	2.079746683	0.509481	4.082091	4.46E-05	0.000353
PAQR9-AS1	61.3959926	4.237694157	0.765148	5.538398	3.05E-08	5.20E-07
CHST2	1296.01658	-3.608681658	0.33508	-10.7696	4.79E-27	5.74E-25
SLC9A9	99.93804	3.373114085	0.57916	5.824154	5.74E-09	1.11E-07
PLOD2	5553.94858	-2.417237194	0.327627	-7.37801	1.61E-13	6.07E-12
AC069528.2	9.00845539	3.949293107	1.277459	3.091523	0.001991	0.008536
LINC02010	10.2501954	3.994010754	1.278276	3.124529	0.001781	0.007777
ZIC4	23.0717234	5.461781762	1.175049	4.64813	3.35E-06	3.61E-05
CPB1	18.8254757	3.994370265	1.144729	3.489359	0.000484	0.002662
CP	51.9985426	4.754948165	0.861659	5.518365	3.42E-08	5.77E-07
CPHL1P	12.2797346	4.224539264	1.26635	3.335996	0.00085	0.004242
TM4SF1	5541.75845	-3.548750052	0.340682	-10.4166	2.08E-25	2.25E-23
ANKUB1	20.0991677	5.203998622	1.195333	4.353597	1.34E-05	0.000124
RNF13	2490.369	2.093282322	0.40514	5.166814	2.38E-07	3.40E-06
CLRN1-AS1	8.94402608	3.914238868	1.279942	3.058139	0.002227	0.009318
CLRN1	11.3484295	4.282003863	1.258062	3.403651	0.000665	0.003466
IGSF10	57.6662611	4.209728706	0.807148	5.215557	1.83E-07	2.68E-06

AADACP1	19.5397912	5.345730665	1.176603	4.543359	5.54E-06	5.63E-05
MBNL1-AS1	108.782005	-2.135632125	0.647459	-3.29848	0.000972	0.00473
P2RY1	794.226223	6.389379267	0.420458	15.19622	3.75E-52	2.08E-49
ARHGEF26	918.379755	-2.62013134	0.341963	-7.66203	1.83E-14	7.63E-13
GPR149	11.8140821	4.278206312	1.260145	3.395012	0.000686	0.003556
MME	1434.34972	-2.277385652	0.365126	-6.23725	4.45E-10	1.04E-08
PLCH1	80.7795017	3.249074064	0.641937	5.061358	4.16E-07	5.66E-06
C3orf33	96.5865255	2.042287882	0.579002	3.527253	0.00042	0.002369
KCNAB1	406.830016	4.359903742	0.441459	9.876121	5.28E-23	4.93E-21
TIPARP-AS1	116.008188	3.76147849	0.562476	6.687353	2.27E-11	6.50E-10
LINC00881	13.5331862	4.511043366	1.24495	3.623474	0.000291	0.001749
SHOX2	15.3872675	3.660638162	1.178764	3.105488	0.0019	0.008199
LXN	973.591049	-3.152741009	0.360146	-8.75407	2.06E-18	1.31E-16
IQCJ-SCHIP1	375.071129	-3.291402645	0.421566	-7.80756	5.83E-15	2.56E-13
IQCJ	14.1540562	4.436031606	1.254048	3.537369	0.000404	0.002294
IL12A-AS1	44.4797739	3.075140849	0.924427	3.326537	0.000879	0.004368
C3orf80	9.62932542	4.002668209	1.275446	3.138251	0.0017	0.0075
SCARNA7	222.150094	5.848183379	0.731066	7.999532	1.25E-15	6.08E-14
PPM1L	557.065298	2.55336648	0.414681	6.157427	7.39E-10	1.67E-08
LINC01192	33.0114839	6.005783836	1.137101	5.281664	1.28E-07	1.93E-06
SI	27.4412367	5.64927285	1.166203	4.844159	1.27E-06	1.53E-05
SLITRK3	57.0645412	5.960913532	0.909679	6.552766	5.65E-11	1.53E-09
LINC01322	46.2667125	5.409138577	1.013521	5.33698	9.45E-08	1.46E-06
WDR49	18.728569	5.152460706	1.196239	4.307217	1.65E-05	0.000149
LRRC77P	19.6218036	4.955015753	1.221047	4.058005	4.95E-05	0.000386
EGFEM1P	23.8539295	4.319946218	1.121995	3.850236	0.000118	0.000819
AC008040.1	29.2045298	4.726109391	1.067916	4.425546	9.62E-06	9.20E-05
CLDN11	2347.86644	-2.410566501	0.317349	-7.59594	3.06E-14	1.25E-12
SLC7A14-AS1	26.3142335	4.008293165	1.044049	3.839181	0.000123	0.00085
SLC2A2	24.4694926	3.66108529	1.036588	3.531861	0.000413	0.002335
TMEM212	13.9080505	4.552167927	1.242358	3.664136	0.000248	0.001529
LINC01209	9.56489611	3.948402431	1.27948	3.085944	0.002029	0.008665
PEX5L	56.6322975	4.295050523	0.777181	5.526446	3.27E-08	5.53E-07
LINC02053	13.8553328	4.695897067	1.227583	3.82532	0.000131	0.000891
SOX2-OT	84.016233	4.118704706	0.794913	5.181326	2.20E-07	3.17E-06
AC068308.1	16.2860951	4.867545546	1.218917	3.993337	6.51E-05	0.000489
LINC01206	74.5718891	6.205196447	0.933135	6.649839	2.93E-11	8.29E-10
MCF2L2	59.0260796	3.582301507	0.840448	4.262372	2.02E-05	0.000177
HTR3D	10.2501954	3.994010754	1.278276	3.124529	0.001781	0.007777
HTR3C	11.8785114	4.334365593	1.255325	3.452784	0.000555	0.002989
HTR3E	9.25446109	3.938692024	1.279145	3.07916	0.002076	0.008797
VWA5B2	32.603069	3.358083697	0.875348	3.836282	0.000125	0.000858
PSMD2	13118.9128	-2.6358333	0.324763	-8.11618	4.81E-16	2.44E-14
LINC02054	13.5331862	4.511043366	1.24495	3.623474	0.000291	0.001749

LIPH	135.200873	-2.729085307	0.568017	-4.80458	1.55E-06	1.82E-05
ETV5	758.287447	-4.463584833	0.456485	-9.77817	1.40E-22	1.27E-20
LINC02020	9.73182516	4.116361029	1.266495	3.250199	0.001153	0.005447
AC068631.1	37.096921	6.084840405	1.137781	5.347991	8.89E-08	1.38E-06
HRG	10.9354948	3.987525477	1.280888	3.113095	0.001851	0.008019
KNG1	10.3790541	4.122386704	1.268347	3.250204	0.001153	0.005447
MASP1	101.251551	4.345260658	0.628016	6.919023	4.55E-12	1.41E-10
AC007920.2	13.7001152	4.686594183	1.227929	3.816665	0.000135	0.000921
SST	1739.42867	7.883635428	0.554287	14.22303	6.59E-46	2.43E-43
BCL6	639.916508	-2.035187741	0.421543	-4.82794	1.38E-06	1.64E-05
P3H2	3576.97303	-3.812568573	0.313847	-12.1479	5.89E-34	1.06E-31
CLDN1	1158.05107	-4.961894066	0.37211	-13.3345	1.46E-40	4.09E-38
OSTN	13.7909034	4.669103484	1.230113	3.795669	0.000147	0.000987
AC073365.1	107.260191	2.012215058	0.542758	3.707388	0.000209	0.001328
FGF12	102.802315	2.009157584	0.561429	3.578651	0.000345	0.002019
ATP13A5	22.5100955	3.709370437	1.032539	3.592474	0.000328	0.001939
ATP13A4	44.0474739	4.440496133	0.865624	5.129821	2.90E-07	4.07E-06
LINC02028	41.5767564	3.341431745	0.876168	3.81369	0.000137	0.00093
LINC02036	14.1276973	4.598912982	1.238579	3.713055	0.000205	0.001303
LINC02037	11.4772882	4.356824519	1.251683	3.480772	0.0005	0.002731
LINC02048	322.878015	9.333566124	0.937869	9.951887	2.47E-23	2.36E-21
FAM43A	244.357557	-2.441010352	0.495567	-4.92569	8.41E-07	1.06E-05
LINC01968	26.7239855	4.381342846	1.135195	3.859552	0.000114	0.000792
MUC4	73.1636258	5.558472802	0.85352	6.512414	7.40E-11	1.96E-09
TCTEX1D2	168.682465	2.356048087	0.473955	4.971034	6.66E-07	8.61E-06
ANKRD18DP	12.4349521	4.180902917	1.270392	3.291035	0.000998	0.004829
AC079140.2	285.957807	4.038157935	0.485095	8.324475	8.47E-17	4.62E-15
TMEM271	10.1330484	4.143717258	1.26561	3.274087	0.00106	0.005082
AC116565.1	10.0949779	4.005107431	1.276891	3.136609	0.001709	0.007522
PDE6B-AS1	204.185354	4.804810786	0.582194	8.25294	1.55E-16	8.25E-15
ATP5ME	1786.34108	2.403808883	0.324149	7.415753	1.21E-13	4.66E-12
RNF212	84.8904631	3.158156375	0.611119	5.167822	2.37E-07	3.39E-06
AC092535.3	12.367587	4.559473152	1.23582	3.68943	0.000225	0.00141
SPON2	23567.4029	7.254568353	0.366943	19.77029	5.37E-87	1.01E-83
AC092535.5	374.356302	8.295605215	0.743202	11.16199	6.26E-29	8.52E-27
MXD4	6232.45927	-2.035836033	0.328085	-6.20521	5.46E-10	1.26E-08
ZFYVE28	368.152559	-2.356115492	0.423575	-5.56245	2.66E-08	4.58E-07
LINC02600	3038.63512	8.69588195	0.431923	20.13296	3.80E-90	9.19E-87
ADRA2C	14481.4443	8.298131151	0.435405	19.05842	5.59E-81	9.48E-78
OTOP1	12.6545989	4.269665777	1.263756	3.378552	0.000729	0.00374
NSG1	81.5208604	3.133404608	0.671127	4.668872	3.03E-06	3.30E-05
CYTL1	5691.34073	2.2848103	0.410239	5.569461	2.56E-08	4.42E-07
STK32B	1340.47074	4.19795367	0.335357	12.51788	5.96E-36	1.20E-33
LINC01587	10.4434834	4.166374392	1.26483	3.29402	0.000988	0.004787

C4orf50	66.4509556	5.90073914	0.938588	6.286826	3.24E-10	7.70E-09
LINC02495	36.8013803	5.246584627	0.983527	5.334461	9.58E-08	1.48E-06
AC113615.1	10.5342716	4.124847874	1.268711	3.25121	0.001149	0.005437
PPP2R2C	37.4720494	2.622403276	0.851803	3.078651	0.002079	0.008809
MAN2B2	2329.41807	-2.872990434	0.322067	-8.92048	4.64E-19	3.19E-17
LINC02447	57.5341881	4.533664709	0.770927	5.880795	4.08E-09	8.16E-08
ABLIM2	41.2095311	2.510397847	0.825707	3.040301	0.002363	0.009795
GPR78	27.5244275	3.284196377	1.000461	3.282682	0.001028	0.004947
AC005674.1	86.4803907	-2.284157487	0.593328	-3.84974	0.000118	0.000821
WDR1	21601.3089	-2.856826988	0.334494	-8.54073	1.33E-17	7.82E-16
CLNK	21.1918088	3.893008327	1.193632	3.261481	0.001108	0.005276
AC006230.1	37.4338201	2.71900899	0.801598	3.391986	0.000694	0.003589
AC025539.1	18.889905	4.028507423	1.136718	3.54398	0.000394	0.002253
AC005699.1	24.1120525	3.863137845	1.002209	3.854623	0.000116	0.000807
AC007370.2	10.4698423	4.061968818	1.273658	3.189215	0.001427	0.006475
AC007126.1	12.0981582	4.381361397	1.251818	3.499999	0.000465	0.002588
LINC00504	38.4267604	5.355706311	0.963906	5.556254	2.76E-08	4.74E-07
CPEB2	1026.77143	2.600897718	0.337164	7.714048	1.22E-14	5.16E-13
C1QTNF7	9.47410791	3.993729441	1.275601	3.130862	0.001743	0.007649
BST1	114.654869	-2.201292905	0.539245	-4.08217	4.46E-05	0.000353
FGFBP1	37.1988268	-2.942070444	0.877694	-3.35205	0.000802	0.004049
PROM1	93.6935438	3.893150696	0.608497	6.39798	1.57E-10	3.96E-09
LDB2	144.93786	2.300949223	0.497172	4.628074	3.69E-06	3.94E-05
AC104656.1	9.53853722	4.032853085	1.27268	3.168788	0.001531	0.006875
AC024230.1	10.9735652	4.22904493	1.261362	3.35276	0.0008	0.00404
KCNIP4-IT1	19.4782977	5.209734792	1.192367	4.369239	1.25E-05	0.000116
AC096719.1	14.9682142	4.603296853	1.241181	3.708802	0.000208	0.001321
GBA3	13.8087335	3.636901763	1.15929	3.137181	0.001706	0.007522
AC097512.1	33.2047718	6.060235527	1.130394	5.361171	8.27E-08	1.29E-06
PPARGC1A	643.75475	4.849921378	0.415755	11.66534	1.92E-31	2.98E-29
SOD3	212.577069	2.590458215	0.465621	5.563451	2.64E-08	4.56E-07
LGI2	18.0583228	3.790062553	1.129509	3.355496	0.000792	0.004019
SLC34A2	34.3214006	4.039936534	1.039684	3.885736	0.000102	0.000724
SEL1L3	461.665922	-2.505934692	0.389799	-6.42879	1.29E-10	3.28E-09
AC097480.2	15.950972	4.060012386	1.177487	3.448033	0.000565	0.00303
AC016687.2	14.2829148	4.592011422	1.239841	3.703711	0.000212	0.001343
DTHD1	23.200582	5.50864123	1.169688	4.709495	2.48E-06	2.76E-05
AL136537.1	17.5014763	4.91466879	1.218333	4.03393	5.49E-05	0.000422
NWD2	26.2522144	5.559090045	1.173605	4.736765	2.17E-06	2.45E-05
C4orf19	477.935464	3.690314157	0.410391	8.992196	2.42E-19	1.70E-17
TLR1	11.7232939	4.332937102	1.254862	3.45292	0.000555	0.002989
KLB	23.5147253	3.424027329	1.051608	3.255993	0.00113	0.005369
N4BP2	144.027026	2.422913972	0.500005	4.845777	1.26E-06	1.52E-05
RHOH	16.4521014	3.395944421	1.117732	3.038246	0.00238	0.009848

NSUN7	101.287962	3.066765662	0.580565	5.282385	1.28E-07	1.92E-06
APBB2	1180.56513	-2.747105802	0.346367	-7.9312	2.17E-15	1.02E-13
UCHL1	26822.4479	-2.136695612	0.400838	-5.33057	9.79E-08	1.51E-06
LINC00682	14.063268	4.542184092	1.243891	3.651593	0.000261	0.001594
BEND4	20.254791	4.049942856	1.091967	3.708852	0.000208	0.001321
AC024022.1	12.0337289	4.332064733	1.25612	3.448766	0.000563	0.003025
ATP8A1	1553.19025	4.203905373	0.338678	12.4127	2.23E-35	4.37E-33
AC080132.1	76.7890915	5.818880204	0.806574	7.214318	5.42E-13	1.91E-11
AC114757.1	23.4582993	5.581676473	1.161388	4.806041	1.54E-06	1.81E-05
YIPF7	9.0728847	3.978599865	1.275392	3.119511	0.001812	0.007878
GABRG1	19.0917218	4.954989948	1.219411	4.063428	4.84E-05	0.000378
RAC1P2	114.806319	2.965745328	0.655448	4.52476	6.05E-06	6.09E-05
GABRA4	31.6850741	4.932483698	1.031964	4.779704	1.76E-06	2.03E-05
GABRB1	23.8660468	3.864703862	0.996617	3.877824	0.000105	0.000742
AC107398.3	20.4359616	5.083261705	1.210034	4.200924	2.66E-05	0.000225
ATP10D	1342.66358	-2.326178938	0.331828	-7.0102	2.38E-12	7.66E-11
OCIAD2	2786.13811	-2.106842865	0.36171	-5.82468	5.72E-09	1.11E-07
CWH43	21.2501196	5.190539354	1.200823	4.322484	1.54E-05	0.00014
AC118282.4	11.0907123	3.911858271	1.286693	3.040241	0.002364	0.009795
LINC01618	10.7275595	4.247693306	1.258756	3.374517	0.000739	0.003787
AC124017.1	13.1846808	4.268440808	1.265487	3.372963	0.000744	0.003804
GSX2	630.22568	7.014553469	0.527403	13.30019	2.31E-40	6.33E-38
PDGFRA	91.879123	5.818352063	0.745942	7.800007	6.19E-15	2.70E-13
KIT	108.747064	3.589612746	0.596372	6.019086	1.75E-09	3.73E-08
NMU	242.648932	3.502966529	0.487318	7.188252	6.56E-13	2.29E-11
SPINK2	22.7173464	5.558473657	1.162624	4.780972	1.74E-06	2.02E-05
IGFBP7	14080.7356	-2.145855018	0.356328	-6.02213	1.72E-09	3.68E-08
LINC02380	11.3484295	4.282003863	1.258062	3.403651	0.000665	0.003466
ADGRL3	480.140717	3.52979246	0.398336	8.861354	7.90E-19	5.22E-17
TECRL	19.1942215	5.152043895	1.198046	4.300371	1.71E-05	0.000152
LINC02232	28.2378115	5.946899517	1.130286	5.26141	1.43E-07	2.14E-06
EPHA5	176.246872	3.81523083	0.520219	7.333893	2.24E-13	8.27E-12
EPHA5-AS1	58.8173011	4.149767203	0.756234	5.487408	4.08E-08	6.80E-07
TMPRSS11D	16.9713944	4.89148208	1.218905	4.013014	5.99E-05	0.000456
TMPRSS11A	11.9429407	4.379058258	1.251429	3.499245	0.000467	0.002588
TMPRSS11F	9.56489611	3.948402431	1.27948	3.085944	0.002029	0.008665
TMPRSS11BNL	18.6904986	5.050527958	1.207739	4.181803	2.89E-05	0.000241
UGT2B10	8.94402608	3.914238868	1.279942	3.058139	0.002227	0.009318
UGT2A1	10.7275595	4.247693306	1.258756	3.374517	0.000739	0.003787
HTN1	10.5987009	4.173911508	1.264765	3.300148	0.000966	0.004718
PRR27	18.2512049	4.975026463	1.214478	4.096431	4.20E-05	0.000334
AMBN	10.9354948	3.987525477	1.280888	3.113095	0.001851	0.008019
ENAM	19.1095519	4.058272087	1.13189	3.585395	0.000337	0.001975
GC	9.84897224	4.056445929	1.271884	3.189321	0.001426	0.006475

ALB	14.7019681	3.625532786	1.176744	3.080986	0.002063	0.008762
RASSF6	421.194431	2.987187606	0.434013	6.882717	5.87E-12	1.80E-10
CXCL6	142.431151	3.096481788	0.55003	5.62966	1.81E-08	3.21E-07
PF4V1	15.1585663	4.910093696	1.210214	4.057211	4.97E-05	0.000387
CXCL1	139.307273	2.385854128	0.516483	4.619427	3.85E-06	4.08E-05
CXCL2	39.4572407	2.939380181	0.790682	3.717524	0.000201	0.001285
AC093677.2	14.3209853	4.716160301	1.227336	3.842598	0.000122	0.000839
EREG	20.2280263	5.264234866	1.188713	4.428518	9.49E-06	9.09E-05
BTC	203.658379	3.284939015	0.471721	6.96373	3.31E-12	1.05E-10
PARM1	2260.5405	4.774319737	0.333909	14.29827	2.24E-46	8.64E-44
PARM1-AS1	15.9493012	4.922814214	1.211523	4.063326	4.84E-05	0.000378
CDKL2	203.0281	2.545816421	0.450742	5.648058	1.62E-08	2.91E-07
PPEF2	12.2006579	4.500321563	1.240794	3.626968	0.000287	0.001729
ART3	9.84897224	4.056445929	1.271884	3.189321	0.001426	0.006475
FAM47E	30.9238809	4.3861049	0.964342	4.548286	5.41E-06	5.53E-05
SHROOM3-AS1	14.3209853	4.716160301	1.227336	3.842598	0.000122	0.000839
SOWAHB	27.1753965	4.049496843	0.982981	4.11961	3.80E-05	0.000307
ANXA3	2889.27009	-3.747624728	0.343325	-10.9157	9.70E-28	1.23E-25
LINC00989	18.6260693	5.004139642	1.212623	4.126708	3.68E-05	0.000298
FGF5	1227.15921	-2.732809344	0.353149	-7.73841	1.01E-14	4.30E-13
RASGEF1B	85.6953368	2.652028928	0.604001	4.390771	1.13E-05	0.000106
AC079160.1	10.8447066	4.116553698	1.270511	3.240076	0.001195	0.005595
CDS1	220.297669	2.608652394	0.463189	5.63194	1.78E-08	3.17E-07
WDFY3-AS2	287.898974	2.931316811	0.462683	6.335479	2.37E-10	5.78E-09
MAPK10	349.059964	3.184674728	0.425277	7.488471	6.97E-14	2.74E-12
PTPN13	1080.64961	2.000931297	0.357679	5.594211	2.22E-08	3.87E-07
HSD17B11	616.66769	2.441458392	0.424942	5.745386	9.17E-09	1.72E-07
DSPP	10.2238366	4.116439207	1.268272	3.245707	0.001172	0.005504
HERC5	61.4387663	3.327417956	0.688876	4.83021	1.36E-06	1.63E-05
NAP1L5	746.940417	2.49600746	0.437595	5.703923	1.17E-08	2.15E-07
FAM13A-AS1	48.3590908	3.080418142	0.817835	3.766554	0.000166	0.001094
FAM13A	1332.19727	2.70811492	0.35504	7.627635	2.39E-14	9.87E-13
AC097478.4	14.8891375	4.83302621	1.217134	3.970824	7.16E-05	0.000532
MMRN1	15.1149029	3.741440246	1.154394	3.241043	0.001191	0.005585
CCSER1	80.2620127	4.876573057	0.718083	6.7911	1.11E-11	3.29E-10
TMSB4XP8	1893.0772	-2.413875698	0.437948	-5.51179	3.55E-08	5.98E-07
AC110774.1	17.906288	3.425159008	1.043668	3.281846	0.001031	0.004958
GRID2	71.3928575	2.433046956	0.666892	3.648335	0.000264	0.001613
BMPR1B	260.461335	3.316851209	0.492303	6.737412	1.61E-11	4.68E-10
UNC5C	27.2747135	4.42456824	1.074754	4.116821	3.84E-05	0.00031
STPG2-AS1	19.8648735	5.331247559	1.179251	4.520875	6.16E-06	6.19E-05
ADH1B	28.009389	5.749373478	1.155368	4.976225	6.48E-07	8.41E-06
ADH7	13.5448977	4.676080843	1.228401	3.80664	0.000141	0.000954
C4orf17	14.2038382	4.771328413	1.221032	3.90762	9.32E-05	0.000668

MTTP	19.2003401	4.023246537	1.142341	3.52193	0.000428	0.002411
DAPP1	13.701786	3.826164826	1.196225	3.198533	0.001381	0.006333
EMCN	23.5232541	3.741823044	1.097155	3.410477	0.000648	0.003409
SLC39A8	744.68536	2.427060285	0.373588	6.496618	8.21E-11	2.16E-09
LINC02428	13.5595451	4.310027042	1.263134	3.412169	0.000644	0.00339
TACR3	11.2914474	3.761507092	1.208414	3.112763	0.001853	0.008024
CXXC4	93.153035	2.12463551	0.56639	3.751186	0.000176	0.001151
CXXC4-AS1	28.7210472	5.695808823	1.164276	4.892146	9.97E-07	1.23E-05
EEF1A1P9	172.668276	2.269312579	0.495241	4.582242	4.60E-06	4.78E-05
NPNT	243.415707	4.191927453	0.496799	8.43788	3.23E-17	1.82E-15
CYP2U1-AS1	28.29925	3.251132899	0.933207	3.483827	0.000494	0.002706
LEF1	142.499383	3.692731284	0.547866	6.740212	1.58E-11	4.60E-10
LEF1-AS1	44.1299803	3.915453281	0.820045	4.774678	1.80E-06	2.08E-05
CFI	854.707313	3.406371373	0.456175	7.467251	8.19E-14	3.21E-12
RRH	18.7022101	5.196411724	1.190962	4.363205	1.28E-05	0.000119
EGF	237.207515	-2.073938836	0.46086	-4.50015	6.79E-06	6.75E-05
ENPEP	35.3405189	4.103315791	0.956784	4.288652	1.80E-05	0.00016
PITX2	24.2583354	3.922568256	1.041243	3.767198	0.000165	0.001092
AC109347.1	24.9208645	3.25791326	0.948324	3.435443	0.000592	0.003144
TIFA	233.883209	2.531585832	0.443797	5.704381	1.17E-08	2.14E-07
ANK2	634.678198	2.186920415	0.376832	5.803444	6.50E-09	1.25E-07
AC004057.1	354.729515	3.087376739	0.506682	6.093326	1.11E-09	2.42E-08
ARSJ	703.98509	-2.457978534	0.354369	-6.93622	4.03E-12	1.26E-10
UGT8	255.673224	3.648820883	0.501343	7.278086	3.39E-13	1.23E-11
AC093765.2	12.4466636	4.49720804	1.242071	3.620732	0.000294	0.001764
LINC02263	25.8509912	5.607625271	1.166362	4.807792	1.53E-06	1.80E-05
LINC01378	10.7802773	4.027023489	1.277437	3.152423	0.001619	0.007214
NDST3	114.513534	5.606128898	0.669105	8.378553	5.36E-17	2.96E-15
TMEM155	116.111486	2.045463536	0.616215	3.319399	0.000902	0.004458
TRPC3	27.0400135	5.691275949	1.159682	4.907619	9.22E-07	1.15E-05
ADAD1	13.1583219	4.468559565	1.247619	3.58167	0.000341	0.002
IL21	10.7802773	4.027023489	1.277437	3.152423	0.001619	0.007214
IL21-AS1	82.6734405	-2.411087513	0.712189	-3.38546	0.000711	0.003663
FGF2	5262.32648	-2.22900175	0.311169	-7.16331	7.87E-13	2.71E-11
LINC01091	45.8452489	4.839541048	0.968776	4.995521	5.87E-07	7.70E-06
LINC02379	9.56489611	3.948402431	1.27948	3.085944	0.002029	0.008665
AC093591.2	12.7307398	4.561535224	1.236825	3.688101	0.000226	0.001416
LINC02466	14.8571857	3.625897229	1.179074	3.075206	0.002104	0.008903
LINC02465	21.5873193	4.052617455	1.108554	3.65577	0.000256	0.001573
LINC02377	10.5987009	4.173911508	1.264765	3.300148	0.000966	0.004718
SNHG27	11.9692996	4.268166641	1.261583	3.383183	0.000717	0.003686
AC105383.1	44.6434028	3.063249164	0.965841	3.171589	0.001516	0.006822
PCDH10	940.515256	-3.06485465	0.367632	-8.33675	7.64E-17	4.17E-15
AC015631.1	32.5721902	5.969924296	1.140557	5.234217	1.66E-07	2.44E-06

PCDH18	449.713121	4.183605299	0.469556	8.9097	5.12E-19	3.50E-17
SLC7A11	246.502687	2.040887541	0.442337	4.613872	3.95E-06	4.18E-05
LINC00499	52.6511176	5.657941076	0.981152	5.766632	8.09E-09	1.53E-07
NOCT	235.204397	-2.095917042	0.466996	-4.48808	7.19E-06	7.11E-05
PPP1R14BP3	554.23012	-2.646390085	0.451791	-5.85755	4.70E-09	9.26E-08
SCOC-AS1	71.4590518	2.741230246	0.641245	4.274855	1.91E-05	0.000169
MGAT4D	20.0347384	5.167627166	1.199299	4.308874	1.64E-05	0.000148
LINC02432	29.8619913	2.967196618	0.970292	3.058045	0.002228	0.00932
GAB1	385.8949	2.140586445	0.386047	5.544884	2.94E-08	5.02E-07
AC107223.1	44.8570094	3.409477087	0.89461	3.811131	0.000138	0.000938
FREM3	26.1994967	5.677772367	1.158647	4.900345	9.57E-07	1.18E-05
GYPE	58.1451702	2.460738465	0.722318	3.406723	0.000657	0.003443
POU4F2	11.6705761	4.433451561	1.24521	3.560405	0.00037	0.00214
NR3C2	212.893639	2.290477903	0.459822	4.981225	6.32E-07	8.23E-06
AC002460.2	18.5488467	3.772758109	1.096131	3.441885	0.000578	0.003086
MAB21L2	532.228282	2.024662879	0.370244	5.468459	4.54E-08	7.51E-07
AC097375.5	15.498296	4.616383185	1.241704	3.71778	0.000201	0.001284
AC023424.2	11.7496528	4.203681987	1.266356	3.31951	0.000902	0.004457
FHDC1	61.9866562	2.372881672	0.690229	3.437819	0.000586	0.00312
AC106865.1	14.9682142	4.603296853	1.241181	3.708802	0.000208	0.001321
SFRP2	16.6528364	3.303393995	1.059545	3.117749	0.001822	0.007921
DCHS2	59.8426393	5.44571522	0.919801	5.920534	3.21E-09	6.54E-08
FGB	12.3441639	4.315094906	1.258772	3.428019	0.000608	0.003216
LRAT	3189.20729	7.025060616	0.42125	16.67669	1.94E-62	1.49E-59
RBM46	14.423485	4.801424275	1.218695	3.939809	8.15E-05	0.000595
AC107208.1	12.62824	4.428525514	1.249417	3.544475	0.000393	0.00225
ASIC5	9.88704267	4.137612642	1.265241	3.270216	0.001075	0.005136
GLRB	695.710863	2.419671753	0.39936	6.058879	1.37E-09	2.95E-08
GRIA2	380.892002	7.738598807	0.627484	12.33275	6.03E-35	1.15E-32
TMEM144	323.413402	2.422950781	0.444212	5.454492	4.91E-08	8.06E-07
RXFP1	18.2365576	5.179353955	1.191165	4.348141	1.37E-05	0.000126
FNIP2	1510.85885	2.733736442	0.328957	8.310307	9.55E-17	5.17E-15
FSTL5	57.1204417	5.362714642	0.850717	6.303758	2.91E-10	6.99E-09
AC106872.12	10.3790541	4.122386704	1.268347	3.250204	0.001153	0.005447
AC106872.5	9.72011361	3.945326647	1.280246	3.081694	0.002058	0.008746
AC106872.6	20.4931907	4.777535874	1.107215	4.314913	1.60E-05	0.000144
CPE	7462.22861	3.374973119	0.328621	10.27011	9.61E-25	9.96E-23
AC080079.2	27.8541715	5.75180873	1.154563	4.981808	6.30E-07	8.20E-06
SPOCK3	617.757268	5.10365865	0.418449	12.1966	3.24E-34	5.97E-32
PALLD	5582.28194	-2.460756872	0.308542	-7.97543	1.52E-15	7.29E-14
LINC02275	22.6558529	5.573301966	1.15959	4.80627	1.54E-06	1.81E-05
MFAP3L	1020.36367	4.59179627	0.359324	12.77898	2.15E-37	4.64E-35
LINC02504	13.5331862	4.511043366	1.24495	3.623474	0.000291	0.001749
SCRG1	18.9543344	4.057469103	1.129907	3.590975	0.000329	0.001946

LINC02269	16.1308776	4.8690777	1.218154	3.997097	6.41E-05	0.000482
AC106895.2	15.0326435	4.668138074	1.235019	3.779812	0.000157	0.001042
LINC02268	24.4046575	4.300018782	1.076741	3.993552	6.51E-05	0.000488
HPGD	31.3392259	6.112658691	1.117806	5.468444	4.54E-08	7.51E-07
GLRA3	25.5024858	5.519527922	1.176067	4.69321	2.69E-06	2.97E-05
GPM6A	1200.27599	5.797193739	0.443037	13.08513	4.00E-39	1.01E-36
MARK2P4	11.0643534	4.176955156	1.266227	3.298742	0.000971	0.004726
WDR17	217.640781	3.000752461	0.453765	6.613004	3.77E-11	1.05E-09
TENM3	1406.95949	-2.458631216	0.349792	-7.02884	2.08E-12	6.77E-11
ENPP6	20.3954809	3.469937839	1.136987	3.051871	0.002274	0.009494
AC107222.2	9.25446109	3.938692024	1.279145	3.07916	0.002076	0.008797
LINC02427	15.0707139	4.794131131	1.222066	3.922974	8.75E-05	0.000633
CASP3	2358.92831	-2.072967788	0.381482	-5.43399	5.51E-08	8.96E-07
AC112243.1	9.62932542	4.002668209	1.275446	3.138251	0.0017	0.0075
LINC02436	21.455119	5.427435842	1.173431	4.625272	3.74E-06	3.98E-05
ANKRD37	100.048383	-2.372680543	0.571196	-4.15388	3.27E-05	0.000269
CCDC110	51.0911948	2.368939581	0.719841	3.290921	0.000999	0.004829
AC106897.1	41.5801616	3.184329588	0.792823	4.016443	5.91E-05	0.00045
PDLIM3	261.28337	2.616728469	0.43174	6.060896	1.35E-09	2.92E-08
SORBS2	890.08334	3.62375561	0.408644	8.867762	7.46E-19	4.96E-17
FAM149A	142.333986	2.049875001	0.508727	4.029424	5.59E-05	0.000429
RPSAP70	9.9895424	4.177336101	1.262442	3.308932	0.000937	0.004611
CYP4V2	584.952642	2.852100792	0.378571	7.533854	4.93E-14	1.97E-12
F11	10.6250598	4.049542664	1.275176	3.175673	0.001495	0.006735
AC097521.1	33.1930602	5.95295246	1.144521	5.20126	1.98E-07	2.87E-06
LINC01060	21.4673561	3.670506564	1.086165	3.379328	0.000727	0.003732
AC093909.4	10.6250598	4.049542664	1.275176	3.175673	0.001495	0.006735
DUX4L9	78.5665381	6.257966808	0.935727	6.687812	2.27E-11	6.50E-10
LRRC14B	13.3779687	4.51654038	1.243835	3.63114	0.000282	0.001704
ZDHHC11B	590.226677	2.746653137	0.424114	6.476215	9.41E-11	2.44E-09
ZDHHC11	54.6204943	2.371393615	0.702384	3.376207	0.000735	0.003769
AC116351.1	14.9860443	3.689134002	1.165607	3.164989	0.001551	0.006955
AC116351.2	10.2238366	4.116439207	1.268272	3.245707	0.001172	0.005504
SLC6A19	20.4476731	5.290551296	1.186383	4.459396	8.22E-06	8.01E-05
SLC6A18	10.8447066	4.116553698	1.270511	3.240076	0.001195	0.005595
TERT	28.3677604	3.532733015	0.997577	3.541314	0.000398	0.00227
SLC6A3	14.5467506	3.62241693	1.175044	3.082793	0.002051	0.008733
IRX4	9.87533112	3.936139891	1.281456	3.071616	0.002129	0.008981
AC124852.1	45.8216034	2.94164978	0.849433	3.463075	0.000534	0.002895
AC094105.2	10.3146248	4.067189627	1.272691	3.195739	0.001395	0.006369
LINC01019	11.7877232	4.373743725	1.25132	3.495305	0.000474	0.002615
AC106799.2	16.1308776	4.8690777	1.218154	3.997097	6.41E-05	0.000482
LINC01020	12.4085932	4.379167019	1.253201	3.494385	0.000475	0.002618
LINC02145	14.566991	4.684070234	1.231647	3.803095	0.000143	0.000964

AC093307.1	16.6186246	4.260137005	1.175312	3.624686	0.000289	0.001743
LINC02196	16.672671	5.029546155	1.202387	4.182968	2.88E-05	0.00024
ADCY2	59.2256278	6.778547509	1.092391	6.205241	5.46E-10	1.26E-08
C5orf49	234.442396	3.720371552	0.453193	8.209246	2.23E-16	1.17E-14
LINC02199	34.1624357	6.001688723	1.140871	5.260618	1.44E-07	2.14E-06
LINC02112	11.5036471	4.283732053	1.258499	3.403843	0.000664	0.003466
CCT5	4402.06374	-2.067231997	0.324035	-6.37966	1.77E-10	4.42E-09
DAP	4260.19832	-2.018040435	0.372297	-5.42052	5.94E-08	9.60E-07
AC016576.1	10.5342716	4.124847874	1.268711	3.25121	0.001149	0.005437
OTULINL	77.6451871	4.793997527	0.717478	6.681735	2.36E-11	6.75E-10
AC092335.1	9.56489611	3.948402431	1.27948	3.085944	0.002029	0.008665
AC024588.1	24.413553	3.92713167	1.042329	3.76765	0.000165	0.00109
BASP1-AS1	118.574111	3.657167964	0.565134	6.471329	9.71E-11	2.52E-09
LINC02217	23.2532998	5.393042027	1.183989	4.554976	5.24E-06	5.37E-05
LINC02218	8.78880857	3.89750018	1.280684	3.043295	0.00234	0.009716
LINC02223	54.0918437	3.31825294	0.720855	4.603219	4.16E-06	4.38E-05
LINC02241	26.1467789	5.747647383	1.149429	5.000439	5.72E-07	7.53E-06
AC093274.1	11.9429407	4.379058258	1.251429	3.499245	0.000467	0.002588
AC138951.1	49.3564153	5.469870923	1.016756	5.379727	7.46E-08	1.18E-06
AC138951.2	11.5300059	4.129467712	1.271744	3.247089	0.001166	0.005489
CDH12	46.3835809	6.704119975	1.075614	6.232832	4.58E-10	1.07E-08
AC091938.1	10.5606305	3.934530818	1.283673	3.065058	0.002176	0.009142
PRDM9	10.3146248	4.067189627	1.272691	3.195739	0.001395	0.006369
CDH10	247.337422	-2.713124243	0.435624	-6.22813	4.72E-10	1.10E-08
LINC02211	16.9511541	3.839203187	1.157915	3.315617	0.000914	0.00451
AC113370.1	13.0031044	4.473281904	1.246584	3.588433	0.000333	0.001957
CDH9	10.572342	4.23381264	1.259403	3.361761	0.000774	0.003938
AC008825.1	10.9735652	4.22904493	1.261362	3.35276	0.0008	0.00404
LINC02109	20.6997974	4.132996576	1.133384	3.6466	0.000266	0.001622
LINC02064	15.3430785	4.639636316	1.238938	3.74485	0.000181	0.001174
AC099517.1	8.78880857	3.89750018	1.280684	3.043295	0.00234	0.009716
PDZD2	1143.73882	5.425160385	0.379983	14.27739	3.03E-46	1.15E-43
NPR3	202.992178	-2.234420298	0.615883	-3.628	0.000286	0.001724
LINC02160	13.6884037	4.501752988	1.246408	3.611783	0.000304	0.001818
ADAMTS12	1208.94971	-2.475037193	0.333929	-7.41186	1.25E-13	4.79E-12
AC138409.1	24.8674939	3.835168118	1.085744	3.532295	0.000412	0.002333
AC026801.2	62.6517679	2.723129701	0.758201	3.591569	0.000329	0.001942
AGXT2	18.1401764	3.968443119	1.141889	3.475331	0.00051	0.002783
PRLR	44.8899952	6.46123056	1.106433	5.839697	5.23E-09	1.02E-07
SPEF2	333.659216	2.530627878	0.402736	6.283584	3.31E-10	7.84E-09
IL7R	108.468122	-2.297172874	0.69319	-3.31392	0.00092	0.004536
UGT3A1	28.1646065	5.746369684	1.156241	4.969873	6.70E-07	8.66E-06
UGT3A2	35.8756682	6.322107532	1.102341	5.735164	9.74E-09	1.82E-07
RANBP3L	23.5975409	4.906176217	1.107624	4.429459	9.45E-06	9.06E-05

AC008957.1	12.3441639	4.315094906	1.258772	3.428019	0.000608	0.003216
NIPBL-DT	369.542443	2.075024377	0.394561	5.259069	1.45E-07	2.15E-06
LINC02119	12.0717993	4.448565762	1.245315	3.57224	0.000354	0.002061
LIFR-AS1	36.6166627	2.68023812	0.832812	3.218299	0.00129	0.005982
OSMR	636.845955	-2.59839508	0.371794	-6.9888	2.77E-12	8.84E-11
DAB2	2856.38308	-2.989355877	0.332407	-8.99306	2.40E-19	1.69E-17
C7	42.122439	6.454792913	1.100518	5.865234	4.49E-09	8.87E-08
MROH2B	26.5160502	4.56873275	1.083868	4.215213	2.50E-05	0.000213
C6	27.8278126	5.793791592	1.149039	5.042293	4.60E-07	6.20E-06
PLCXD3	18.5997104	5.092663295	1.202655	4.234516	2.29E-05	0.000198
GHR	100.853331	2.147334124	0.580963	3.696165	0.000219	0.001378
CCDC152	164.861322	2.65334182	0.506713	5.236381	1.64E-07	2.42E-06
SELENOP	110.643071	2.730266674	0.584645	4.669958	3.01E-06	3.29E-05
NIM1K	100.927442	3.154194928	0.568929	5.544092	2.95E-08	5.04E-07
NNT-AS1	690.220235	2.603771923	0.353735	7.360798	1.83E-13	6.82E-12
FGF10	19.2825995	3.524761843	1.096369	3.21494	0.001305	0.006036
HCN1	29.1137416	4.748764749	1.060219	4.479043	7.50E-06	7.37E-05
AC010478.1	20.4335513	3.569971451	1.103189	3.236047	0.001212	0.005665
PELO	1257.86379	-2.009055535	0.390994	-5.13833	2.77E-07	3.90E-06
FST	120.938382	-2.092884418	0.58767	-3.56132	0.000369	0.002134
LINC02105	11.3103591	4.03853363	1.278167	3.159629	0.00158	0.007069
LINC01033	15.6007958	4.833628509	1.219919	3.962255	7.42E-05	0.000548
HSPB3	398.812832	2.893255912	0.504496	5.734947	9.75E-09	1.82E-07
ESM1	12.00737	4.41656174	1.248133	3.538534	0.000402	0.002286
AC034238.1	12.821528	4.544094362	1.238932	3.667753	0.000245	0.001511
CDC20B	15.3228382	3.610121128	1.18962	3.034684	0.002408	0.009949
MCIDAS	30.7352326	2.746469995	0.895429	3.067213	0.002161	0.009093
CCNO	502.860111	2.9942533	0.433673	6.904404	5.04E-12	1.55E-10
DDX4	28.4665127	4.774430154	1.045021	4.56874	4.91E-06	5.06E-05
LINC01948	33.7963471	6.211258256	1.111518	5.588089	2.30E-08	3.99E-07
MAP3K1	448.98363	2.745427657	0.397526	6.906286	4.98E-12	1.54E-10
ACTBL2	342.980875	-5.721371614	0.552298	-10.3592	3.80E-25	4.06E-23
LINCR-0003	12.4085932	4.379167019	1.253201	3.494385	0.000475	0.002618
PLK2	12240.3225	-5.223782685	0.315938	-16.5342	2.08E-61	1.57E-58
RAB3C	27.2130611	4.684861555	1.057699	4.429296	9.45E-06	9.06E-05
PDE4D	469.344093	4.348734572	0.394502	11.02336	2.95E-28	3.87E-26
PART1	36.2564042	6.089448876	1.135017	5.365074	8.09E-08	1.27E-06
C5orf64	62.857124	6.903816502	1.081587	6.383044	1.74E-10	4.33E-09
AC026746.1	10.3790541	4.122386704	1.268347	3.250204	0.001153	0.005447
AC092353.1	10.2501954	3.994010754	1.278276	3.124529	0.001781	0.007777
CD180	10.1330484	4.143717258	1.26561	3.274087	0.00106	0.005082
AC112206.2	20.8752552	5.165395553	1.202415	4.29585	1.74E-05	0.000155
AC093523.1	18.7168575	4.92681021	1.221153	4.034555	5.47E-05	0.000421
SMN2	110.30661	-2.713121912	0.619733	-4.37789	1.20E-05	0.000112

PMCHL2	8.69802037	3.906713832	1.279695	3.052849	0.002267	0.009467
ZNF366	12.0337289	4.332064733	1.25612	3.448766	0.000563	0.003025
LINC01333	12.4466636	4.49720804	1.242071	3.620732	0.000294	0.001764
ENC1	8234.08686	-5.953102163	0.313019	-19.0183	1.20E-80	1.94E-77
ANKDD1B	29.3901006	3.461312327	0.892371	3.878781	0.000105	0.00074
SV2C	35.5711048	6.084793251	1.133804	5.366706	8.02E-08	1.26E-06
IQGAP2	541.799634	7.242099025	0.520957	13.90153	6.20E-44	2.04E-41
F2RL2	714.010713	-2.080093369	0.378195	-5.50006	3.80E-08	6.36E-07
ZBED3-AS1	76.8556341	2.130583812	0.621938	3.425718	0.000613	0.003241
OTP	12.4993814	4.297396672	1.260864	3.408295	0.000654	0.003427
ACTBP2	175.28404	-2.287073182	0.494266	-4.62721	3.71E-06	3.95E-05
SCAMP1-AS1	517.714499	2.056851058	0.422038	4.873617	1.10E-06	1.34E-05
DMGDH	65.5369356	2.055383448	0.642355	3.199762	0.001375	0.00631
BHMT	11.7232939	4.332937102	1.254862	3.45292	0.000555	0.002989
CMYA5	57.7507499	2.936980904	0.759565	3.86666	0.00011	0.000773
THBS4-AS1	16.1045187	4.927957808	1.211573	4.067406	4.75E-05	0.000373
CKMT2	67.1069151	3.03797218	0.662921	4.582703	4.59E-06	4.78E-05
ACOT12	11.1551416	4.079209622	1.274555	3.200497	0.001372	0.006296
VCAN	2067.64014	-4.257246884	0.441218	-9.64885	4.97E-22	4.35E-20
EDIL3	2893.12965	-2.502531259	0.359619	-6.95884	3.43E-12	1.08E-10
RPL5P17	160.062998	2.275343446	0.512307	4.441368	8.94E-06	8.63E-05
AC114971.1	22.0642775	5.284973018	1.192732	4.430982	9.38E-06	9.02E-05
LINC00461	67.2529962	6.922264516	1.086479	6.371281	1.87E-10	4.66E-09
MEF2C-AS2	11.9048703	4.177926253	1.269005	3.292284	0.000994	0.004813
MEF2C-AS1	30.8135759	3.169903123	1.037744	3.054611	0.002254	0.009418
AC113167.1	15.7560133	4.834659718	1.220416	3.961487	7.45E-05	0.000549
ADGRV1	169.07446	3.779740859	0.579146	6.526401	6.74E-11	1.79E-09
AC114316.1	23.7477936	3.285098146	1.065615	3.082819	0.00205	0.008733
AC106818.2	10.7539184	4.178531017	1.264949	3.303319	0.000955	0.004683
POU5F2	19.0009336	5.032568275	1.210814	4.156352	3.23E-05	0.000266
AC008534.1	11.4655766	3.961938055	1.28417	3.085213	0.002034	0.008681
FAM81B	28.8516096	2.991633955	0.919856	3.252285	0.001145	0.005429
RFESD	52.6600461	2.66958406	0.703088	3.796941	0.000146	0.000983
ELL2	727.603181	-2.250353981	0.350705	-6.41665	1.39E-10	3.53E-09
PCSK1	27.7379557	3.639020018	0.985125	3.693968	0.000221	0.001389
AC122697.1	12.1245171	4.251364794	1.263572	3.36456	0.000767	0.003905
DDX18P4	10.9354948	3.987525477	1.280888	3.113095	0.001851	0.008019
AC021086.1	11.1287827	4.232433809	1.261651	3.354678	0.000795	0.004025
ST8SIA4	35.5455184	4.156833815	0.932308	4.458649	8.25E-06	8.03E-05
SLCO4C1	64.3363093	6.15079697	0.893277	6.885656	5.75E-12	1.76E-10
SLCO6A1	14.2184855	4.527395237	1.245853	3.633973	0.000279	0.001689
AC099520.1	14.2946264	4.76152773	1.222429	3.895135	9.81E-05	0.0007
LINC01023	114.618536	3.093951448	0.564952	5.476486	4.34E-08	7.20E-07
TMEM232	58.7484496	2.468817833	0.691171	3.571937	0.000354	0.002062

TSLP	182.502284	-2.506961376	0.495885	-5.05553	4.29E-07	5.82E-06
RPS3AP21	165.990042	3.802325326	0.553111	6.874439	6.22E-12	1.90E-10
EPB41L4A	133.362479	2.259747896	0.587866	3.843986	0.000121	0.000836
AC104126.1	33.9875109	4.154178672	0.999805	4.154988	3.25E-05	0.000267
APC	1160.43657	2.057736343	0.335961	6.124918	9.07E-10	2.01E-08
AC010226.1	40.445886	3.537298464	0.901396	3.924246	8.70E-05	0.000631
CDO1	1688.08615	4.604143084	0.404449	11.38375	5.04E-30	7.17E-28
AC093295.1	14.2363156	3.611303113	1.172747	3.079355	0.002074	0.008797
LINC00992	14.6726735	3.744445612	1.149023	3.258807	0.001119	0.005322
LINC02208	13.0938926	4.404207729	1.253385	3.513852	0.000442	0.002474
PTMAP2	1321.48883	4.103532196	0.482703	8.50115	1.88E-17	1.09E-15
MIR1244-2	1418.12408	3.801898244	0.414531	9.171576	4.66E-20	3.53E-18
LOX	2401.18535	-2.611395388	0.328925	-7.93919	2.04E-15	9.59E-14
SNCAIP	251.172868	4.573403741	0.501868	9.112756	8.03E-20	5.90E-18
LINC01170	23.6281641	5.415111023	1.182595	4.579008	4.67E-06	4.84E-05
LINC02240	18.3800636	5.060486706	1.20549	4.197869	2.69E-05	0.000227
AC116362.1	16.1369961	3.731453725	1.172713	3.181898	0.001463	0.006621
AC011416.4	17.5659056	4.960923812	1.213556	4.087923	4.35E-05	0.000345
MEGF10	36.2803528	4.475856887	1.005082	4.453226	8.46E-06	8.21E-05
SLC12A2	1222.07521	2.137991465	0.338029	6.324881	2.53E-10	6.16E-09
FBN2	4877.00632	-2.724524266	0.349826	-7.78824	6.80E-15	2.96E-13
AC008679.1	12.317805	4.427294542	1.248333	3.546565	0.00039	0.002237
ADAMTS19	73.6970126	2.638756493	0.642429	4.107467	4.00E-05	0.000321
AC008591.1	23.8275706	4.392775405	1.102045	3.986022	6.72E-05	0.000502
ACSL6	46.7526053	6.410220096	1.117508	5.736175	9.68E-09	1.81E-07
PDLIM4	3392.1677	-2.09395614	0.368599	-5.68085	1.34E-08	2.44E-07
LEAP2	34.2566621	3.162297768	0.836096	3.782217	0.000155	0.001035
FSTL4	28.6315962	3.305983249	0.963465	3.431347	0.000601	0.003187
AC010307.3	13.131963	4.556748427	1.238911	3.678026	0.000235	0.001464
VDAC1	5211.41656	-2.165956401	0.343743	-6.30109	2.96E-10	7.09E-09
TIFAB	10.2882659	4.156236444	1.265118	3.285256	0.001019	0.004906
CXCL14	1907.29282	4.087269348	0.355901	11.4843	1.58E-30	2.32E-28
SLC25A48	31.3982211	4.628953567	1.060106	4.366501	1.26E-05	0.000117
TGFBI	37207.0751	-7.103282688	0.322243	-22.0432	1.11E-107	7.52E-104
SPOCK1	4935.834	-2.665401664	0.337513	-7.89719	2.85E-15	1.31E-13
AC106791.1	47.4122156	2.865786204	0.781317	3.66789	0.000245	0.001511
LRRTM2	32.7686609	5.070350447	0.999364	5.073578	3.90E-07	5.34E-06
AC011379.1	11.8140821	4.278206312	1.260145	3.395012	0.000686	0.003556
PFDN1	1365.40381	-2.081463404	0.384751	-5.4099	6.31E-08	1.01E-06
HBEGF	467.144836	-4.109919887	0.401156	-10.2452	1.24E-24	1.28E-22
AC116353.6	9.72011361	3.945326647	1.280246	3.081694	0.002058	0.008746
PCDHA5	11.3484295	4.282003863	1.258062	3.403651	0.000665	0.003466
PCDHA9	20.1212622	4.589712659	1.14372	4.012968	6.00E-05	0.000456
PCDHB1	17.7855524	4.996609759	1.210433	4.127953	3.66E-05	0.000297

AC244517.1	10.8447066	4.116553698	1.270511	3.240076	0.001195	0.005595
FCHSD1	1670.42166	-2.227081008	0.330187	-6.7449	1.53E-11	4.46E-10
ARAP3	891.361359	-2.251331807	0.347	-6.48799	8.70E-11	2.28E-09
FGF1	573.834624	-3.989933128	1.041771	-3.82995	0.000128	0.000878
SH3RF2	2363.22021	-3.411854071	0.321816	-10.6019	2.92E-26	3.32E-24
GPR151	11.4392177	4.228541376	1.263141	3.347639	0.000815	0.004096
DPYSL3	8725.40593	-4.953729447	0.334091	-14.8275	9.73E-50	4.71E-47
C5orf46	398.67276	-3.864781379	0.60197	-6.42022	1.36E-10	3.46E-09
AC011352.3	101.774267	-5.420584098	0.713994	-7.59192	3.15E-14	1.28E-12
HTR4	19.1297922	5.118109717	1.201709	4.259027	2.05E-05	0.000179
SH3TC2	227.417143	2.602077082	0.524	4.965796	6.84E-07	8.83E-06
PPARGC1B	98.4477041	2.389473573	0.637481	3.748306	0.000178	0.001161
CDX1	44.6511275	3.925777607	0.878751	4.46745	7.92E-06	7.74E-05
CAMK2A	859.639816	-2.654954814	0.349542	-7.59552	3.07E-14	1.25E-12
ARSI	14379.8572	-2.79865117	0.303666	-9.21621	3.08E-20	2.34E-18
NDST1	8579.59257	-3.588153935	0.319943	-11.215	3.44E-29	4.78E-27
SYNPO	4821.87867	-2.539738376	0.314986	-8.06301	7.44E-16	3.72E-14
TNIP1	4685.66254	-2.585202549	0.338365	-7.64028	2.17E-14	8.97E-13
SLC36A3	14.3651742	3.662844521	1.162012	3.152158	0.001621	0.007218
SLC36A2	12.5374518	4.470435694	1.245048	3.590572	0.00033	0.001948
FAT2	47.5041881	5.104437205	0.996944	5.120084	3.05E-07	4.27E-06
GLRA1	10.9735652	4.22904493	1.261362	3.35276	0.0008	0.00404
LINC01933	74.1237881	6.358694318	0.877173	7.249075	4.20E-13	1.51E-11
AC008571.2	11.7496528	4.203681987	1.266356	3.31951	0.000902	0.004457
GRIA1	100.865787	4.02425894	0.627391	6.41428	1.41E-10	3.58E-09
AC010476.2	19.6715856	5.278916846	1.184845	4.455364	8.38E-06	8.14E-05
SGCD	768.289589	7.531683296	0.479484	15.70788	1.34E-55	8.54E-53
ITK	40.1112555	4.249044901	0.961338	4.419927	9.87E-06	9.42E-05
FAM71B	14.6841381	4.427132779	1.256444	3.523543	0.000426	0.0024
ADAM19	3302.20749	-4.691053501	0.339628	-13.8123	2.15E-43	6.74E-41
LINC02227	10.1594073	4.067721584	1.272091	3.197666	0.001385	0.006342
AC091939.1	11.1287827	4.232433809	1.261651	3.354678	0.000795	0.004025
EBF1	32.9695242	2.944111288	0.94557	3.113585	0.001848	0.008016
LINC02202	10.2501954	3.994010754	1.278276	3.124529	0.001781	0.007777
AC008691.1	44.9004197	2.886604778	0.851163	3.391365	0.000695	0.003596
LINC01845	13.1846808	4.268440808	1.265487	3.372963	0.000744	0.003804
LINC01847	27.5762139	4.601300796	1.086325	4.235657	2.28E-05	0.000197
CCNJL	401.308176	-2.097351486	0.390744	-5.36758	7.98E-08	1.25E-06
AC112191.2	155.878251	3.606185918	0.593109	6.080142	1.20E-09	2.62E-08
GABRB2	1168.08326	7.227759174	1.114214	6.486866	8.76E-11	2.29E-09
GABRA6	13.0675337	4.51829105	1.242467	3.636549	0.000276	0.001677
GABRA1	186.354219	5.41837035	0.566025	9.572675	1.04E-21	8.83E-20
LINC01202	17.8997637	5.183276474	1.189434	4.357768	1.31E-05	0.000122
GABRG2	57.6734528	6.812584962	1.084749	6.280332	3.38E-10	8.00E-09

AC113414.1	32.7452378	4.966251631	1.032662	4.809173	1.52E-06	1.79E-05
AC109466.1	31.6145263	6.014450742	1.131771	5.314195	1.07E-07	1.64E-06
AC026403.1	1062.1476	2.645643096	0.402612	6.571192	4.99E-11	1.36E-09
CTB-178M22.2	77.1898223	3.34917503	0.742369	4.511467	6.44E-06	6.44E-05
SLIT3	5755.53997	-4.271975647	0.325226	-13.1354	2.06E-39	5.34E-37
LCP2	24.7908275	5.572384882	1.167244	4.773969	1.81E-06	2.08E-05
KCNIP1	11.2576413	4.313999964	1.254792	3.438019	0.000586	0.003119
KCNMB1	16.8693005	3.661932311	1.144724	3.198966	0.001379	0.006327
GABRP	11.9048703	4.177926253	1.269005	3.292284	0.000994	0.004813
FGF18	451.009168	-3.371770444	0.389094	-8.66569	4.49E-18	2.75E-16
SMIM23	11.0907123	3.911858271	1.286693	3.040241	0.002364	0.009795
STK10	1120.42842	-2.405473705	0.335669	-7.1662	7.71E-13	2.66E-11
SH3PXD2B	1746.02204	-2.520847798	0.393097	-6.41279	1.43E-10	3.61E-09
DUSP1	7094.64852	-4.596842725	0.308569	-14.8973	3.43E-50	1.74E-47
ERGIC1	5387.9414	-2.480643703	0.354938	-6.98896	2.77E-12	8.84E-11
STC2	4808.38634	-6.352486966	0.350642	-18.1167	2.35E-73	2.85E-70
LINC01484	20.4801506	4.100150428	1.139116	3.599414	0.000319	0.001895
LINC01411	25.7986792	4.50177237	1.037336	4.339742	1.43E-05	0.000131
DRD1	77.4270192	7.44649489	1.027527	7.247005	4.26E-13	1.53E-11
HRH2	61.3615022	5.017079708	0.779871	6.433216	1.25E-10	3.19E-09
CPLX2	27.5700953	5.706603032	1.159427	4.921917	8.57E-07	1.08E-05
THOC3	813.642626	-2.276869224	0.354743	-6.41837	1.38E-10	3.50E-09
HIGD2A	3035.65925	2.059204176	0.315579	6.525168	6.79E-11	1.81E-09
RNF44	1362.87952	-2.364408717	0.328842	-7.19012	6.47E-13	2.26E-11
GPRIN1	314.194071	-2.449635448	0.411549	-5.95223	2.65E-09	5.48E-08
EIF4E1B	13.8172623	4.603767001	1.236903	3.722012	0.000198	0.001268
LINC01574	10.1711188	4.201461847	1.260808	3.332356	0.000861	0.004292
SLC34A1	24.2490342	5.373128416	1.18947	4.517244	6.26E-06	6.29E-05
DBN1	18297.0889	-3.41621515	0.340866	-10.0222	1.22E-23	1.18E-21
AC145098.2	272.397559	-3.653622688	0.520376	-7.02112	2.20E-12	7.12E-11
PDLIM7	14310.599	-4.701352665	0.354862	-13.2484	4.61E-40	1.25E-37
FAM153CP	54.2794335	5.71830007	0.972858	5.877836	4.16E-09	8.28E-08
NHP2	1202.49837	-2.176616949	0.43179	-5.04091	4.63E-07	6.24E-06
COL23A1	16.7253887	4.937215118	1.212992	4.070278	4.70E-05	0.000369
AACSP1	14.256556	4.682592502	1.230583	3.805184	0.000142	0.000958
ADAMTS2	1633.31403	-4.263554098	0.35862	-11.8888	1.35E-32	2.26E-30
HMGB3P22	19.7429451	3.074010777	1.009557	3.04491	0.002327	0.009678
TBC1D9B	3705.51946	-2.27888957	0.313214	-7.27582	3.44E-13	1.25E-11
RASGEF1C	16.622889	4.718421594	1.235477	3.81911	0.000134	0.000912
BTNL9	20.8488963	5.255990436	1.191943	4.409598	1.04E-05	9.83E-05
AL512308.1	9.69375473	4.046019533	1.27217	3.180407	0.001471	0.006648
LINC01622	23.5256644	5.159475123	1.210851	4.261031	2.03E-05	0.000178
AL033381.1	10.6894891	4.123736642	1.269366	3.248658	0.00116	0.005468
FOXF2	280.682059	-2.201423937	0.415925	-5.29284	1.20E-07	1.83E-06

TUBB2A	1038.68005	-2.318535197	0.414031	-5.59991	2.14E-08	3.77E-07
TUBB2B	649.514575	-2.460844095	0.389896	-6.31154	2.76E-10	6.68E-09
AL033523.1	34.3076455	3.215808258	0.92217	3.487219	0.000488	0.002677
PXDC1	1152.0408	-3.342819027	0.340123	-9.82828	8.51E-23	7.88E-21
FAM217A	12.0981582	4.381361397	1.251818	3.499999	0.000465	0.002588
RNA5SP202	52.5427462	5.812012352	0.928336	6.260678	3.83E-10	9.04E-09
NRN1	16.168948	4.95255636	1.209082	4.096129	4.20E-05	0.000335
F13A1	23.8214521	5.506387158	1.172149	4.697686	2.63E-06	2.91E-05
LY86-AS1	24.2724573	5.64192654	1.15659	4.87807	1.07E-06	1.31E-05
BMP6	2979.04215	3.403330054	0.356236	9.553581	1.25E-21	1.06E-19
HULC	152.735919	3.287780982	0.593735	5.537454	3.07E-08	5.22E-07
OFCC1	26.6270788	5.578272218	1.172423	4.757899	1.96E-06	2.23E-05
LINC00518	30.8696842	2.92655449	0.897179	3.261953	0.001106	0.005268
GCNT2	835.897464	-2.368132592	0.347063	-6.82335	8.89E-12	2.66E-10
MAK	74.8087953	3.324221166	0.649136	5.120994	3.04E-07	4.25E-06
GCM2	11.6852234	4.08769102	1.275511	3.204748	0.001352	0.006218
ELOVL2	233.104068	3.51790144	0.476392	7.384463	1.53E-13	5.81E-12
NEDD9	628.818144	-2.849333104	0.438711	-6.49478	8.32E-11	2.18E-09
AL022098.1	8.94402608	3.914238868	1.279942	3.058139	0.002227	0.009318
HIVEP1	675.239834	-2.023162451	0.40996	-4.93502	8.01E-07	1.02E-05
EDN1	424.991206	-4.220167864	1.099582	-3.83797	0.000124	0.000853
AL138720.1	12.4085932	4.379167019	1.253201	3.494385	0.000475	0.002618
STMND1	15.044355	4.841515625	1.216835	3.978778	6.93E-05	0.000516
RNF144B	315.33618	-2.209062724	0.417719	-5.28839	1.23E-07	1.87E-06
ID4	3180.03156	-4.060735425	0.331597	-12.246	1.77E-34	3.30E-32
MBOAT1	44.2800277	2.279255074	0.739627	3.081628	0.002059	0.008746
AL139093.1	14.1276973	4.598912982	1.238579	3.713055	0.000205	0.001303
DCDC2	258.55283	2.871825567	0.437304	6.567111	5.13E-11	1.40E-09
AL022170.1	9.62932542	4.002668209	1.275446	3.138251	0.0017	0.0075
SLC17A4	16.8425358	4.775281338	1.230546	3.880621	0.000104	0.000735
SLC17A1	12.0600878	4.135111753	1.272919	3.248527	0.00116	0.005468
SLC17A3	11.0643534	4.176955156	1.266227	3.298742	0.000971	0.004726
U91328.1	75.5853534	2.253858905	0.6281	3.588375	0.000333	0.001957
H4C1	24.2871046	5.497062047	1.174874	4.678852	2.88E-06	3.17E-05
H4C2	22.2575655	5.388374503	1.181148	4.561979	5.07E-06	5.21E-05
H4C3	87.4717714	2.873854187	0.750176	3.830909	0.000128	0.000875
H2BC4	91.1260661	-2.613979921	0.724002	-3.61046	0.000306	0.001825
H2AC6	681.704368	-2.099862848	0.363909	-5.7703	7.91E-09	1.50E-07
H1-4	71.2150175	4.942223642	0.829566	5.957605	2.56E-09	5.32E-08
H2BC5	186.963749	-2.150827631	0.493528	-4.35806	1.31E-05	0.000122
H4C5	159.176648	3.18772286	0.652103	4.88837	1.02E-06	1.25E-05
H1-3	34.6280882	5.983557152	1.144461	5.228275	1.71E-07	2.51E-06
H4C6	15.3345497	3.771717038	1.150165	3.279284	0.001041	0.005001
LINC00240	25.201352	4.005697803	1.024943	3.908217	9.30E-05	0.000666

H4C9	116.881557	-2.320742685	0.536202	-4.32811	1.50E-05	0.000137
H2BC12	455.769889	-3.773970737	0.397417	-9.49625	2.18E-21	1.80E-19
H2AC12	10.572342	4.23381264	1.259403	3.361761	0.000774	0.003938
POM121L2	12.5638107	4.372264827	1.254408	3.485521	0.000491	0.00269
ZNF204P	172.272127	5.094536289	0.599109	8.503528	1.84E-17	1.07E-15
AL009179.1	64.5262728	3.734048708	0.689261	5.417471	6.04E-08	9.75E-07
H1-5	26.070638	5.630664171	1.164193	4.836539	1.32E-06	1.58E-05
GPX6	12.2533757	4.381831723	1.252369	3.498834	0.000467	0.002588
OR14J1	26.8203667	5.669263405	1.161761	4.879887	1.06E-06	1.30E-05
OR2H1	13.0938926	4.404207729	1.253385	3.513852	0.000442	0.002474
LINC01015	11.7496528	4.203681987	1.266356	3.31951	0.000902	0.004457
OR2I1P	723.945396	6.74183244	1.122482	6.006184	1.90E-09	4.02E-08
MOG	377.395715	5.800710922	0.490468	11.8269	2.83E-32	4.57E-30
HLA-F	327.239486	5.82130515	0.541021	10.75985	5.33E-27	6.36E-25
HLA-F-AS1	27.3917017	4.792602946	1.026341	4.669601	3.02E-06	3.29E-05
HLA-V	9.3188904	3.98167413	1.276003	3.120427	0.001806	0.007857
HLA-H	170.537625	2.874790194	0.604794	4.753336	2.00E-06	2.27E-05
TRIM31	11.4392177	4.228541376	1.263141	3.347639	0.000815	0.004096
TRIM10	10.1594073	4.067721584	1.272091	3.197666	0.001385	0.006342
AL662873.1	12.8742457	4.341881084	1.258229	3.450788	0.000559	0.003008
PPP1R18	3257.18121	-4.170560796	0.331141	-12.5945	2.26E-36	4.65E-34
IER3	1841.31668	-3.748150375	0.424482	-8.82994	1.05E-18	6.83E-17
DDR1	5228.46379	-3.574322611	0.30878	-11.5756	5.48E-31	8.18E-29
MUC21	9.53853722	4.032853085	1.27268	3.168788	0.001531	0.006875
MUC22	20.3451734	5.151379363	1.202217	4.284899	1.83E-05	0.000162
PSORS1C1	72.22939	-3.236118095	0.672073	-4.81513	1.47E-06	1.74E-05
AL662844.4	65.357816	2.78704475	0.693842	4.016826	5.90E-05	0.00045
HLA-B	16827.2532	2.988344243	0.358437	8.337144	7.61E-17	4.17E-15
HCP5	603.614364	3.176933433	0.404311	7.857643	3.91E-15	1.75E-13
MICB	290.997592	-2.302666993	0.415842	-5.53736	3.07E-08	5.22E-07
LY6G5B	88.708784	-2.012111593	0.616452	-3.26402	0.001098	0.005233
SLC44A4	25.078612	3.530759579	1.036974	3.404866	0.000662	0.003463
C4B	98.9517438	2.233546358	0.569868	3.919411	8.88E-05	0.00064
TNXB	83.3303556	2.365787811	0.661491	3.576447	0.000348	0.002034
AGPAT1	982.479136	-2.439130702	0.413721	-5.8956	3.73E-09	7.51E-08
GPSM3	75.1719029	-2.043947798	0.613186	-3.33333	0.000858	0.00428
NOTCH4	89.4443859	2.654906475	0.590672	4.49472	6.97E-06	6.90E-05
TSBP1-AS1	47.8453736	5.571178173	0.925192	6.021644	1.73E-09	3.68E-08
HLA-DQA1	10.5342716	4.124847874	1.268711	3.25121	0.001149	0.005437
PSMB9	424.894502	3.486545749	0.504474	6.911249	4.80E-12	1.49E-10
HLA-DOA	11.6705761	4.433451561	1.24521	3.560405	0.00037	0.00214
HLA-DPB1	15.0033488	4.889037769	1.21198	4.033925	5.49E-05	0.000422
RXRB	2095.11883	-2.310727332	0.354085	-6.5259	6.76E-11	1.80E-09
TAPBP	3827.78955	-2.553264607	0.310745	-8.2166	2.09E-16	1.11E-14

ITPR3	1007.69942	-2.908447521	0.477839	-6.08667	1.15E-09	2.52E-08
IP6K3	374.673971	2.07101641	0.413126	5.013037	5.36E-07	7.10E-06
LINC01016	15.4075078	4.704549513	1.232708	3.816434	0.000135	0.000921
AL138889.1	54.5076091	6.670779244	1.098249	6.074015	1.25E-09	2.71E-08
GRM4	50.1004312	5.45880326	0.972832	5.61125	2.01E-08	3.55E-07
HMGA1	20461.4511	-2.729973109	0.324647	-8.40905	4.13E-17	2.29E-15
AL451165.2	95.099282	2.07420037	0.672648	3.083634	0.002045	0.008718
TEAD3	1305.01949	-3.081832196	0.337869	-9.12137	7.42E-20	5.47E-18
TULP1	552.360258	7.102205426	0.532117	13.34708	1.23E-40	3.48E-38
BNIP5	10.6748417	4.296235077	1.254311	3.425174	0.000614	0.003246
KCTD20	2428.81272	-2.535668993	0.316207	-8.01901	1.07E-15	5.21E-14
STK38	2804.40224	-2.280552027	0.356189	-6.40265	1.53E-10	3.85E-09
CDKN1A	10342.0143	-4.0231776	0.304498	-13.2125	7.43E-40	1.98E-37
CPNE5	331.949637	5.205367376	0.453738	11.4722	1.82E-30	2.66E-28
FGD2	27.5173776	5.793171635	1.148098	5.045888	4.51E-07	6.10E-06
AL096712.2	9.47410791	3.993729441	1.275601	3.130862	0.001743	0.007649
DNAH8	63.2319884	6.910912423	1.08126	6.391535	1.64E-10	4.11E-09
GLP1R	20.7961786	5.360298474	1.179233	4.54558	5.48E-06	5.58E-05
KCNK5	284.815649	7.302538418	0.629444	11.60157	4.05E-31	6.13E-29
KIF6	271.039141	2.047861676	0.42595	4.807752	1.53E-06	1.80E-05
AL592158.1	22.855824	3.426673596	1.092796	3.135694	0.001714	0.007545
DAAM2-AS1	80.2079443	3.324506532	0.694797	4.78486	1.71E-06	1.99E-05
TREML4	11.6588646	4.283776559	1.259077	3.402316	0.000668	0.003478
FOXP4	2542.585	-2.298229471	0.340355	-6.75245	1.45E-11	4.26E-10
PGC	10.0949779	4.005107431	1.276891	3.136609	0.001709	0.007522
PTCRA	10.4698423	4.061968818	1.273658	3.189215	0.001427	0.006475
PEX6	2715.18689	2.105999719	0.339575	6.201867	5.58E-10	1.29E-08
PPP2R5D	710.880894	-2.231803518	0.407121	-5.48192	4.21E-08	7.00E-07
PTK7	8540.48048	-3.16804441	0.30649	-10.3365	4.82E-25	5.13E-23
SRF	2870.99983	-2.588297438	0.321707	-8.04552	8.59E-16	4.25E-14
SLC22A7	11.696935	4.398045908	1.248667	3.522193	0.000428	0.002409
LRRC73	192.533658	2.640661785	0.482264	5.475554	4.36E-08	7.23E-07
YIPF3	3195.16693	-2.0255485	0.357135	-5.67166	1.41E-08	2.56E-07
RSPH9	115.287912	2.056629867	0.589812	3.486926	0.000489	0.002679
AL109615.2	67.6746847	3.58124606	0.810563	4.41822	9.95E-06	9.48E-05
TMEM63B	2163.81138	-2.013753031	0.322536	-6.24351	4.28E-10	1.00E-08
AL035701.1	18.7053929	4.173145278	1.095343	3.809897	0.000139	0.000942
ENPP5	494.864096	2.187550178	0.446888	4.89507	9.83E-07	1.21E-05
CYP39A1	205.956917	2.147936806	0.458722	4.682437	2.83E-06	3.12E-05
TDRD6	32.5536537	2.991150612	0.89535	3.34076	0.000835	0.004184
ADGRF5	26.1614262	5.59867	1.168475	4.791435	1.66E-06	1.93E-05
ADGRF1	33.3746366	5.877441576	1.15458	5.090544	3.57E-07	4.93E-06
AL451166.1	8.94402608	3.914238868	1.279942	3.058139	0.002227	0.009318
ADGRF2	17.6947642	5.033756987	1.205926	4.174185	2.99E-05	0.000249

OPN5	21.3916211	3.223830859	1.035156	3.114344	0.001844	0.008
TFAP2B	22.3323777	4.632202322	1.154318	4.012935	6.00E-05	0.000456
PKHD1	62.4822597	6.896674269	1.081916	6.374498	1.84E-10	4.57E-09
PAQR8	384.167758	2.301892215	0.393663	5.847361	4.99E-09	9.80E-08
AL109918.1	859.08171	2.249155643	0.346768	6.486045	8.81E-11	2.30E-09
GSTA8P	19.6305794	5.354360849	1.175791	4.553836	5.27E-06	5.39E-05
GSTA2	18.2219103	5.229303968	1.185444	4.411263	1.03E-05	9.77E-05
GSTA1	107.530345	7.904360919	1.001598	7.891751	2.98E-15	1.36E-13
GCM1	12.1245171	4.251364794	1.263572	3.36456	0.000767	0.003905
AL033397.1	19.9175913	5.26276199	1.187724	4.430964	9.38E-06	9.02E-05
MLIP	38.7198909	4.714287112	0.934496	5.044738	4.54E-07	6.13E-06
TINAG	11.0116356	4.317624376	1.253511	3.444425	0.000572	0.00306
HCRTR2	13.2491101	4.384912975	1.255665	3.492104	0.000479	0.002637
GFRAL	10.8447066	4.116553698	1.270511	3.240076	0.001195	0.005595
HMGCLL1	135.502473	2.743003148	0.519887	5.276149	1.32E-07	1.98E-06
AL445250.1	36.5234227	4.602771097	0.953631	4.826575	1.39E-06	1.65E-05
KHDRBS2	34.0310471	4.984719755	0.985018	5.060537	4.18E-07	5.68E-06
AL355347.1	15.5100076	4.860577653	1.216627	3.995124	6.47E-05	0.000485
FKBP1C	274.192042	-3.418912956	0.44217	-7.73212	1.06E-14	4.51E-13
LGSN	11.696935	4.398045908	1.248667	3.522193	0.000428	0.002409
RPL7AP34	19.3030867	3.511486447	1.108566	3.167594	0.001537	0.006901
EYS	58.5299541	4.332724708	0.897704	4.826452	1.39E-06	1.65E-05
AL646090.2	10.3790541	4.122386704	1.268347	3.250204	0.001153	0.005447
AL391807.1	22.5481659	3.753277895	1.015383	3.696416	0.000219	0.001376
ADGRB3	38.0694792	5.248529598	0.995498	5.272264	1.35E-07	2.02E-06
LMBRD1	2055.63805	2.307209149	0.404357	5.705868	1.16E-08	2.13E-07
COL19A1	44.3979838	6.496271535	1.100379	5.90367	3.56E-09	7.17E-08
COL9A1	40.8664574	5.312650053	0.948964	5.598369	2.16E-08	3.79E-07
AL583856.1	159.552532	-2.086390217	0.485429	-4.29803	1.72E-05	0.000154
RPL39P3	921.919022	-2.712710113	0.407195	-6.66195	2.70E-11	7.64E-10
COL12A1	1800.667	-3.114604208	0.350931	-8.87527	6.98E-19	4.65E-17
IMPG1	16.0551425	3.559925923	1.157878	3.074525	0.002108	0.008923
AL590426.1	13.9344094	4.350352124	1.260838	3.450364	0.00056	0.003011
HMGN3	2743.15734	2.600251538	0.411262	6.322621	2.57E-10	6.24E-09
LCAL1	15.2522903	4.713246257	1.231253	3.828008	0.000129	0.000883
AL391840.1	34.9008586	4.822966187	1.038479	4.644261	3.41E-06	3.66E-05
ELOVL4	441.85279	2.804319591	0.437985	6.40278	1.53E-10	3.85E-09
LINC02542	12.5755223	4.549478553	1.237415	3.676599	0.000236	0.001469
SNAP91	229.330473	3.878303964	0.481363	8.056919	7.82E-16	3.89E-14
RIPPLY2	12.9503866	4.596834275	1.234147	3.724705	0.000196	0.001258
LINC02857	15.2903607	4.82792795	1.219301	3.959585	7.51E-05	0.000553
MRAP2	60.4442884	4.091258952	0.749129	5.461354	4.73E-08	7.78E-07
CNR1	41.6777993	4.859184093	0.908345	5.349492	8.82E-08	1.37E-06
AL139042.1	14.5025617	4.638184454	1.236066	3.752377	0.000175	0.001146

GABRR1	20.2807441	5.100412324	1.207667	4.223361	2.41E-05	0.000206
RRAGD	1651.62377	4.786976158	0.351693	13.61124	3.43E-42	1.04E-39
AL132996.1	30.2205045	5.763407375	1.160215	4.967533	6.78E-07	8.75E-06
EPHA7	55.896588	3.126663664	0.716636	4.362971	1.28E-05	0.000119
AL034348.1	18.3537047	5.123141281	1.19821	4.275661	1.91E-05	0.000168
FUT9	42.8340972	6.439825354	1.10442	5.830959	5.51E-09	1.07E-07
UFL1-AS1	15.6007958	4.833628509	1.219919	3.962255	7.42E-05	0.000548
GPR63	118.778104	2.216685527	0.534215	4.149425	3.33E-05	0.000273
AL589740.1	50.6780436	4.396989541	0.927469	4.740848	2.13E-06	2.41E-05
POU3F2	12.9503866	4.596834275	1.234147	3.724705	0.000196	0.001258
PRDM13	14.6314203	4.722293368	1.227933	3.845727	0.00012	0.000832
AL357139.2	12.6018811	4.505303377	1.241896	3.627762	0.000286	0.001725
LIN28B-AS1	13.6620448	4.602670925	1.236404	3.722627	0.000197	0.001267
BVES	269.038235	2.129632118	0.426093	4.998047	5.79E-07	7.61E-06
BVES-AS1	21.8717621	3.361965242	1.04158	3.227754	0.001248	0.005817
CRYBG1	397.474877	4.285136862	0.445252	9.624063	6.33E-22	5.51E-20
LINC02532	56.68106	5.549909919	0.985972	5.628874	1.81E-08	3.22E-07
CD24	2162.70045	-4.697930947	0.331339	-14.1786	1.24E-45	4.48E-43
SOBP	155.445081	2.469122581	0.497459	4.963465	6.92E-07	8.92E-06
SCML4	20.0878619	3.943193305	1.11783	3.527543	0.000419	0.002368
OSTM1-AS1	14.4381324	4.581379308	1.241445	3.69036	0.000224	0.001405
NR2E1	260.784387	4.026780073	0.476757	8.446192	3.01E-17	1.71E-15
AL139106.1	9.93976043	4.008663455	1.276073	3.141406	0.001681	0.007444
SLC16A10	82.7303394	2.821052684	0.622256	4.533586	5.80E-06	5.86E-05
LAMA4	1350.82755	-3.660387415	0.370434	-9.88134	5.02E-23	4.70E-21
AL049695.1	12.7190282	4.360408778	1.25604	3.471552	0.000517	0.002815
RPS27AP11	32.6562476	4.445919499	0.99979	4.446854	8.71E-06	8.44E-05
FRK	54.1726633	2.706391451	0.693394	3.903109	9.50E-05	0.000679
AL357141.1	18.1665353	3.884717528	1.163365	3.339207	0.00084	0.004202
COL10A1	18.3156342	5.021387416	1.209627	4.151185	3.31E-05	0.000271
GPRC6A	12.8699813	3.870463439	1.207164	3.206244	0.001345	0.006194
RFX6	21.1329725	5.315164782	1.185948	4.481787	7.40E-06	7.29E-05
ROS1	37.7481528	2.983105908	0.849814	3.510303	0.000448	0.002503
DCBLD1	988.620633	-2.8014275	0.344925	-8.12184	4.59E-16	2.33E-14
AL450405.1	1003.32157	-2.099569852	0.465268	-4.51261	6.40E-06	6.40E-05
FAM184A	193.879582	3.333026085	0.521363	6.392909	1.63E-10	4.08E-09
PKIB	1169.66898	2.062445844	0.442725	4.658524	3.18E-06	3.46E-05
Z99129.3	16.7404419	3.594617491	1.1594	3.100412	0.001933	0.008316
FABP7	13.659109	4.716731858	1.225233	3.84966	0.000118	0.000821
CLVS2	35.2343109	6.13097256	1.126704	5.441512	5.28E-08	8.62E-07
TRDN	53.7168742	6.782119608	1.081037	6.273719	3.53E-10	8.33E-09
TRDN-AS1	24.2226753	5.465033226	1.178552	4.637074	3.53E-06	3.78E-05
AL445259.1	13.2491101	4.384912975	1.255665	3.492104	0.000479	0.002637
AL450332.1	11.9692996	4.268166641	1.261583	3.383183	0.000717	0.003686

AL356534.1	121.129139	4.111983599	0.741328	5.546777	2.91E-08	4.98E-07
RSPO3	50.1091764	2.606881145	0.740254	3.521602	0.000429	0.002413
C6orf58	16.6364547	3.56344187	1.127178	3.161383	0.00157	0.007032
LAMA2	583.002915	5.068750353	0.41856	12.10998	9.35E-34	1.67E-31
ENPP3	18.3800636	5.060486706	1.20549	4.197869	2.69E-05	0.000227
AL117378.1	10.2238366	4.116439207	1.268272	3.245707	0.001172	0.005504
CCN2	14553.0562	-5.864476668	0.435193	-13.4756	2.18E-41	6.36E-39
MOXD1	524.525465	-4.012687005	0.428765	-9.3587	8.07E-21	6.39E-19
VNN1	12.2533757	4.381831723	1.252369	3.498834	0.000467	0.002588
VNN3	15.5250607	3.520290557	1.159546	3.035922	0.002398	0.009911
SLC18B1	510.675481	2.010744111	0.401749	5.004979	5.59E-07	7.37E-06
LINC00326	12.8478869	4.474722687	1.24585	3.591704	0.000329	0.001942
TARID	36.7139337	4.531716895	0.930626	4.869538	1.12E-06	1.36E-05
LINC01312	11.6852234	4.08769102	1.275511	3.204748	0.001352	0.006218
LINC01010	24.2343868	5.594308351	1.162549	4.812105	1.49E-06	1.77E-05
AL121970.1	9.47410791	3.993729441	1.275601	3.130862	0.001743	0.007649
LINC00271	44.0569671	2.922348371	0.787766	3.709664	0.000208	0.001318
AL360178.2	9.3188904	3.98167413	1.276003	3.120427	0.001806	0.007857
AL138828.1	34.369337	3.101265677	0.988623	3.136954	0.001707	0.007522
AL023284.4	21.4700133	4.389034576	1.074464	4.084861	4.41E-05	0.000349
MAP7	680.863328	4.216325703	0.408636	10.31805	5.84E-25	6.18E-23
SLC35D3	130.695761	8.127368918	0.994222	8.174604	2.97E-16	1.54E-14
IL20RA	29.9647772	2.972705665	0.872708	3.406302	0.000658	0.003447
AL590617.2	170.833717	2.472856395	0.511801	4.831672	1.35E-06	1.62E-05
TXLNB	36.0897221	5.234928293	0.980213	5.340604	9.26E-08	1.44E-06
CITED2	4217.79073	-2.791775274	0.330075	-8.458	2.72E-17	1.55E-15
LINC01625	16.0781598	4.966801433	1.207122	4.114581	3.88E-05	0.000313
STX11	199.292268	-3.504081561	0.493416	-7.10168	1.23E-12	4.16E-11
AL023283.1	10.9354948	3.987525477	1.280888	3.113095	0.001851	0.008019
FBXO30-DT	75.128182	2.568772801	0.62358	4.119398	3.80E-05	0.000307
ADGB	32.1770855	4.905689531	1.045424	4.692536	2.70E-06	2.97E-05
STXBP5-AS1	220.402599	3.803249996	0.461011	8.249809	1.59E-16	8.45E-15
SAMD5	448.663365	3.096922083	0.397487	7.79125	6.63E-15	2.89E-13
AL031056.1	12.0454404	4.485805374	1.241611	3.612892	0.000303	0.001814
IYD	34.7042291	6.118320714	1.126909	5.429292	5.66E-08	9.17E-07
MTHFD1L	1111.44639	-2.150929414	0.341282	-6.3025	2.93E-10	7.03E-09
CCDC170	100.516531	2.291483687	0.57659	3.974199	7.06E-05	0.000525
ESR1	39.6174376	3.305212452	0.975574	3.387968	0.000704	0.003635
LINC02840	19.5954447	5.09827678	1.205601	4.228826	2.35E-05	0.000202
AL590867.2	237.301056	2.613576506	0.532258	4.910357	9.09E-07	1.13E-05
OPRM1	45.6397239	6.48192566	1.105329	5.864248	4.51E-09	8.92E-08
CNKSR3	357.569825	2.655000057	0.399879	6.63951	3.15E-11	8.86E-10
SYNJ2	596.787553	-2.123717264	0.407981	-5.20543	1.94E-07	2.81E-06
AL360169.3	49.997494	6.802885389	1.070535	6.354661	2.09E-10	5.15E-09

EZR	44742.313	-3.204242367	0.301564	-10.6254	2.27E-26	2.60E-24
C6orf99	569.996058	-5.770740575	0.451022	-12.7948	1.75E-37	3.83E-35
AL356417.3	24.0410989	5.529833581	1.169994	4.726378	2.29E-06	2.56E-05
AL356417.1	14.1276973	4.598912982	1.238579	3.713055	0.000205	0.001303
MAS1	19.1034333	5.181849806	1.194228	4.339081	1.43E-05	0.000131
IGF2R	4814.33751	-2.399823287	0.320035	-7.49863	6.45E-14	2.55E-12
AIRN	10.4698423	4.061968818	1.273658	3.189215	0.001427	0.006475
SLC22A3	683.151618	-5.475410509	0.484872	-11.2925	1.43E-29	2.00E-27
LPA	21.5459072	5.411176017	1.175781	4.602196	4.18E-06	4.40E-05
PLG	16.1953069	4.902893104	1.214708	4.036273	5.43E-05	0.000419
AL109933.5	11.0643534	4.176955156	1.266227	3.298742	0.000971	0.004726
AL139393.1	15.9290609	3.837928704	1.142492	3.359261	0.000782	0.00397
PACRG	82.1835167	2.987640037	0.63489	4.705759	2.53E-06	2.81E-05
PACRG-AS3	30.4137924	5.848925384	1.149961	5.086195	3.65E-07	5.04E-06
PACRG-AS1	24.2871046	5.497062047	1.174874	4.678852	2.88E-06	3.17E-05
AL031121.2	18.9101454	5.085147585	1.204649	4.221268	2.43E-05	0.000208
AL078602.1	21.8709896	5.106628177	1.211859	4.213881	2.51E-05	0.000214
AL450345.1	11.7877232	4.373743725	1.25132	3.495305	0.000474	0.002615
C6orf118	16.7488118	5.085496285	1.196467	4.250427	2.13E-05	0.000186
PDE10A	163.282108	3.90900872	0.558732	6.996215	2.63E-12	8.42E-11
TBXT	12.9123162	4.516215643	1.242058	3.636073	0.000277	0.001678
PRR18	66.692268	4.53053067	0.71877	6.303176	2.92E-10	7.01E-09
AL121956.7	13.6271571	3.632262483	1.157869	3.137024	0.001707	0.007522
MPC1	645.320923	2.125908366	0.481892	4.41159	1.03E-05	9.75E-05
AL022069.1	17.5794713	3.684595062	1.106658	3.329479	0.00087	0.004327
RAMACL	67.5100145	4.093687682	0.710215	5.764015	8.21E-09	1.55E-07
AL023775.2	9.87533112	3.936139891	1.281456	3.071616	0.002129	0.008981
TTLL2	11.3747884	4.154235208	1.269217	3.273069	0.001064	0.00509
TCP10L3	20.163597	5.235881908	1.191835	4.393127	1.12E-05	0.000105
LINC02538	21.0158254	5.383196029	1.17725	4.572688	4.82E-06	4.97E-05
KIF25-AS1	12.1625875	4.422982886	1.248134	3.543676	0.000395	0.002253
FRMD1	27.8805304	5.697638263	1.161524	4.905311	9.33E-07	1.16E-05
CTAGE13P	11.0643534	4.176955156	1.266227	3.298742	0.000971	0.004726
AL606970.1	9.62932542	4.002668209	1.275446	3.138251	0.0017	0.0075
SMOC2	14.7602789	4.784725864	1.221827	3.916041	9.00E-05	0.000647
AL513210.1	11.0643534	4.176955156	1.266227	3.298742	0.000971	0.004726
THBS2	807.569008	-3.976705265	0.471348	-8.43689	3.26E-17	1.83E-15
AL603783.1	20.8986783	5.422074128	1.172022	4.626256	3.72E-06	3.96E-05
DLL1	29.267886	2.764881642	0.909111	3.041302	0.002356	0.009769
AC093627.7	11.2840002	4.232661173	1.262212	3.353368	0.000798	0.004039
AC188617.1	17.5837357	3.980905126	1.130302	3.521984	0.000428	0.002411
GPER1	283.403226	-2.1437817	0.435775	-4.91947	8.68E-07	1.09E-05
MICALL2	597.370882	-2.024037347	0.360146	-5.62005	1.91E-08	3.38E-07
MAFK	1619.83229	-2.997384842	0.32842	-9.12669	7.06E-20	5.23E-18

AC104129.1	13.752833	4.558390099	1.241168	3.672661	0.00024	0.001487
TTYH3	11480.9033	-2.168158847	0.333705	-6.49723	8.18E-11	2.15E-09
RADIL	50.0695549	2.605209486	0.740182	3.519689	0.000432	0.002428
MMD2	10.9091359	4.179411771	1.265451	3.302706	0.000958	0.004688
OR10AH1P	12.7834575	4.424631592	1.250368	3.538662	0.000402	0.002286
ZNF890P	9.42139014	4.068763349	1.269403	3.205257	0.001349	0.006214
AC093620.1	145.579888	4.053955536	0.643211	6.302687	2.93E-10	7.03E-09
AC092171.1	47.2736183	3.301893727	0.789262	4.183518	2.87E-05	0.00024
ACTB	163243.682	-2.72860184	0.338199	-8.06803	7.14E-16	3.57E-14
FSCN1	9742.43169	-2.562353765	0.329176	-7.78415	7.02E-15	3.05E-13
CYTH3	1126.58692	-2.136686362	0.349016	-6.12204	9.24E-10	2.05E-08
DAGLB	1379.10483	-2.345477428	0.375817	-6.241	4.35E-10	1.02E-08
GRID2IP	32.7554704	3.179749104	0.903736	3.51845	0.000434	0.002438
RPA3	881.033643	2.506398572	0.382701	6.549236	5.78E-11	1.56E-09
NXPH1	14.3209853	4.716160301	1.227336	3.842598	0.000122	0.000839
AC060834.2	10.9091359	4.179411771	1.265451	3.302706	0.000958	0.004688
AC004160.1	20.1255266	5.114027585	1.205651	4.241713	2.22E-05	0.000192
THSD7A	199.863925	2.992728931	0.47393	6.314709	2.71E-10	6.55E-09
SCIN	675.941695	3.09339509	0.394931	7.832755	4.77E-15	2.11E-13
AC011287.1	40.3544842	4.139631571	0.964933	4.290071	1.79E-05	0.000159
DGKB	39.4818958	4.14285436	0.8991	4.607781	4.07E-06	4.29E-05
AGMO	9.72011361	3.945326647	1.280246	3.081694	0.002058	0.008746
CRPPA	338.959645	2.391206553	0.408037	5.860275	4.62E-09	9.12E-08
SOSTDC1	321.975587	4.605985519	0.547265	8.416372	3.88E-17	2.16E-15
TMEM196	14.3209853	4.716160301	1.227336	3.842598	0.000122	0.000839
AC005062.1	13.6884037	4.501752988	1.246408	3.611783	0.000304	0.001818
RPL21P75	128.732275	4.699199616	0.584507	8.039592	9.01E-16	4.44E-14
MACC1	36.0018697	5.143535433	1.009478	5.095243	3.48E-07	4.82E-06
ITGB8	1324.93456	2.317596598	0.350181	6.618283	3.63E-11	1.01E-09
ABCB5	823.947023	2.06495902	0.346579	5.958124	2.55E-09	5.31E-08
SP4	252.398095	2.088705146	0.428853	4.870445	1.11E-06	1.36E-05
DNAH11	55.7834107	3.983205117	0.780228	5.105184	3.30E-07	4.60E-06
RAPGEF5	74.5564718	3.050608002	0.672077	4.539077	5.65E-06	5.74E-05
STEAP1B	80.1944753	2.462052829	0.615229	4.00185	6.28E-05	0.000476
IL6	120.149934	2.61332456	0.578671	4.51608	6.30E-06	6.32E-05
TOMM7	7767.16497	2.25819338	0.406998	5.54841	2.88E-08	4.94E-07
KLHL7-DT	35.5938606	3.082495369	0.817784	3.769327	0.000164	0.001083
AC005082.1	30.3232512	4.973769403	1.004675	4.950627	7.40E-07	9.45E-06
AC010677.1	22.5391116	4.28824396	1.059249	4.048383	5.16E-05	0.0004
AC004540.1	58.57186	3.884442872	0.716608	5.420599	5.94E-08	9.60E-07
HOXA3	10.8447066	4.116553698	1.270511	3.240076	0.001195	0.005595
HOXA-AS2	15.8468015	4.790882526	1.22542	3.909584	9.25E-05	0.000663
HOXA-AS3	14.5933499	4.56598022	1.243478	3.671942	0.000241	0.00149
HOXA10	15.9756601	4.868805736	1.217578	3.998762	6.37E-05	0.00048

HOXA13	18.6058289	3.960133775	1.150496	3.442109	0.000577	0.003084
HOTTIP	12.4349521	4.180902917	1.270392	3.291035	0.000998	0.004829
TRIL	169.70591	6.496697514	0.732812	8.865431	7.62E-19	5.04E-17
AC005162.2	12.4730225	4.428932897	1.248782	3.546601	0.00039	0.002237
CPVL	1088.64171	3.358318917	0.39093	8.590597	8.65E-18	5.15E-16
CHN2	21.765554	5.43389933	1.173782	4.629393	3.67E-06	3.92E-05
AC004593.1	10.3790541	4.122386704	1.268347	3.250204	0.001153	0.005447
GHRHR	8.78880857	3.89750018	1.280684	3.043295	0.00234	0.009716
ADCYAP1R1	25.0368332	5.538851586	1.172224	4.72508	2.30E-06	2.58E-05
FKBP9	3238.67008	-2.157098682	0.314609	-6.85645	7.06E-12	2.14E-10
NPSR1-AS1	19.9573325	4.502449329	1.13074	3.98186	6.84E-05	0.00051
AC018647.1	15.1149029	3.741440246	1.154394	3.241043	0.001191	0.005585
AOAH	21.9268901	4.317264482	1.099098	3.928006	8.57E-05	0.000622
ELMO1	48.1865602	3.668031788	0.869826	4.216971	2.48E-05	0.000212
NME8	14.8510671	4.75902198	1.224934	3.885127	0.000102	0.000725
TRG-AS1	29.6065646	3.704568266	0.938875	3.945751	7.96E-05	0.000582
POU6F2	55.1196717	6.963464438	1.057738	6.583353	4.60E-11	1.26E-09
LINC01450	16.1572365	4.774355185	1.228275	3.887041	0.000101	0.000721
INHBA	1515.94493	-3.924438869	0.332234	-11.8123	3.37E-32	5.37E-30
AC005537.1	23.8858814	5.53092654	1.169319	4.730039	2.24E-06	2.52E-05
STK17A	1697.1185	-3.665724851	0.331577	-11.0554	2.06E-28	2.74E-26
NPC1L1	24.5594692	5.337186164	1.19447	4.468247	7.89E-06	7.72E-05
ZMIZ2	3424.24981	-2.08171567	0.325058	-6.40413	1.51E-10	3.82E-09
LINC01952	9.56489611	3.948402431	1.27948	3.085944	0.002029	0.008665
MYO1G	35.8085817	5.102742574	1.020633	4.999586	5.75E-07	7.55E-06
NACAD	410.564106	-2.189380284	0.383784	-5.70472	1.17E-08	2.14E-07
CCDC201	10.1330484	4.143717258	1.26561	3.274087	0.00106	0.005082
IGFBP3	5733.95136	-6.359068894	0.352231	-18.0537	7.38E-73	8.63E-70
C7orf69	43.0759644	-3.699712923	0.911581	-4.05857	4.94E-05	0.000386
SUN3	14.1540562	4.436031606	1.254048	3.537369	0.000404	0.002294
ABCA13	78.510546	2.129793361	0.683476	3.116122	0.001832	0.007957
LINC02838	12.8976688	4.632198292	1.230495	3.764498	0.000167	0.001101
VWC2	35.6355341	6.105420841	1.131244	5.397088	6.77E-08	1.08E-06
IKZF1	39.655508	3.355252962	0.947888	3.539715	0.000401	0.002283
DDC	12.3822343	4.464991456	1.24496	3.586452	0.000335	0.001968
LINC01445	19.5046565	5.145916084	1.199891	4.288653	1.80E-05	0.00016
VSTM2A	18.5733515	5.150495683	1.195872	4.306897	1.66E-05	0.000149
AC006971.1	15.4342725	3.542842726	1.152612	3.07375	0.002114	0.008944
SEPTIN14	13.9258806	3.597573703	1.170967	3.072309	0.002124	0.008977
AC092447.8	14.6577792	4.630645934	1.237395	3.742253	0.000182	0.001185
AC092447.5	16.1308776	4.8690777	1.218154	3.997097	6.41E-05	0.000482
AC118758.2	70.9225628	7.080445496	1.070464	6.614368	3.73E-11	1.04E-09
AC237221.1	14.3473442	4.642711077	1.235021	3.759217	0.00017	0.001119
TNRC18P2	16.5057419	4.903137571	1.215891	4.032546	5.52E-05	0.000423

ZNF727	50.4573066	6.778877134	1.074297	6.310058	2.79E-10	6.74E-09
AC016769.5	25.3051804	4.331490489	1.019538	4.248483	2.15E-05	0.000187
ZNF117	437.234225	-2.982792899	0.389893	-7.6503	2.01E-14	8.32E-13
ERV3-1	266.106483	-2.247556137	0.43641	-5.1501	2.60E-07	3.70E-06
RPL35P5	113.542461	-3.527687011	0.570168	-6.18711	6.13E-10	1.40E-08
LINC02604	100.071511	-2.158087554	0.575314	-3.75115	0.000176	0.001151
AC092100.1	11.7877232	4.373743725	1.25132	3.495305	0.000474	0.002615
CALN1	31.7580322	5.916711194	1.145146	5.166773	2.38E-07	3.40E-06
FKBP6	14.2522916	4.11156024	1.178338	3.489287	0.000484	0.002662
STX1A	180.364818	-2.411680801	0.469529	-5.13639	2.80E-07	3.94E-06
CLDN3	161.694613	2.394198527	0.56346	4.249103	2.15E-05	0.000187
ELN	40623.3264	11.14297432	0.404658	27.53679	6.37E-167	2.16E-162
LIMK1	5500.4879	-2.801411743	0.310191	-9.03126	1.70E-19	1.20E-17
EIF4H	8440.27912	-2.495056216	0.304265	-8.20027	2.40E-16	1.26E-14
AC005081.1	10.5987009	4.173911508	1.264765	3.300148	0.000966	0.004718
YWHAG	5039.46841	-2.076857861	0.310504	-6.68868	2.25E-11	6.46E-10
FGL2	9.3188904	3.98167413	1.276003	3.120427	0.001806	0.007857
MAGI2	368.316149	2.859542732	0.418064	6.839961	7.92E-12	2.38E-10
AC074024.1	12.9767455	4.551258675	1.238839	3.673809	0.000239	0.001482
AC004862.1	14.4381324	4.581379308	1.241445	3.69036	0.000224	0.001405
AC008163.1	25.5141973	5.658504042	1.158798	4.883082	1.04E-06	1.28E-05
HGF	33.0943079	3.849364685	1.089001	3.534765	0.000408	0.002314
CACNA2D1	1155.05427	-2.609911897	0.338621	-7.70748	1.28E-14	5.41E-13
SEMA3D	77.449506	3.201688955	0.660978	4.843868	1.27E-06	1.53E-05
GRM3	12.2533757	4.381831723	1.252369	3.498834	0.000467	0.002588
ABCB1	773.174973	-2.403046297	0.352942	-6.80861	9.85E-12	2.93E-10
ADAM22	190.166996	2.559563098	0.481938	5.310982	1.09E-07	1.67E-06
AC003991.1	20.8403675	4.293225272	1.090853	3.935657	8.30E-05	0.000605
STEAP4	21.4170486	5.367116452	1.180708	4.545677	5.48E-06	5.57E-05
AC002069.2	11.7496528	4.203681987	1.266356	3.31951	0.000902	0.004457
ZNF804B	15.4455783	4.83130926	1.219555	3.961534	7.45E-05	0.000549
STEAP1	37.3966639	3.920711466	0.910997	4.303758	1.68E-05	0.00015
STEAP2	58.6974027	2.421864957	0.687435	3.523048	0.000427	0.002404
FAM237B	10.9999241	4.102900639	1.272157	3.225153	0.001259	0.005856
CYP51A1	369.098111	2.547350735	0.541682	4.702669	2.57E-06	2.85E-05
LRRD1	12.1245171	4.251364794	1.263572	3.36456	0.000767	0.003905
ERVW-1	16.3768833	4.818032405	1.224528	3.934605	8.33E-05	0.000606
CALCR	14.3473442	4.642711077	1.235021	3.759217	0.00017	0.001119
GNGT1	327.724892	9.35742515	0.936399	9.992989	1.64E-23	1.57E-21
GNG11	5050.31568	3.411131508	0.401168	8.502995	1.85E-17	1.07E-15
PEG10	1157.90952	2.176541096	0.356465	6.105898	1.02E-09	2.25E-08
RPS3AP25	46.9417646	3.901655005	0.787497	4.954503	7.25E-07	9.29E-06
PPP1R9A-AS1	14.5025617	4.638184454	1.236066	3.752377	0.000175	0.001146
PON3	22.8637881	5.538736675	1.164683	4.755574	1.98E-06	2.25E-05

ASB4	14.3473442	4.642711077	1.235021	3.759217	0.00017	0.001119
AC002451.1	27.8278126	5.793791592	1.149039	5.042293	4.60E-07	6.20E-06
PDK4	265.736	2.582083644	0.448038	5.763095	8.26E-09	1.56E-07
DLX6-AS1	40.6437476	5.235207025	1.022855	5.118229	3.08E-07	4.31E-06
DLX6	13.6884037	4.501752988	1.246408	3.611783	0.000304	0.001818
RPS3AP26	1163.57434	2.67023738	0.374033	7.139044	9.40E-13	3.21E-11
TMEM130	181.339132	-2.274281023	0.537659	-4.22997	2.34E-05	0.000201
KPNA7	10.1594073	4.067721584	1.272091	3.197666	0.001385	0.006342
PDAP1	4628.65906	-2.332868016	0.344075	-6.78011	1.20E-11	3.54E-10
ATP5MF	1366.94187	-2.061453148	0.459948	-4.48192	7.40E-06	7.29E-05
GAL3ST4	113.250879	-2.523791715	0.569768	-4.42951	9.44E-06	9.06E-05
ZAN	40.579724	4.635804002	0.938015	4.942141	7.73E-07	9.83E-06
EPHB4	2708.0779	-2.323631608	0.326574	-7.11517	1.12E-12	3.79E-11
MUC3A	44.7895721	4.849843181	0.981942	4.939034	7.85E-07	9.98E-06
MUC12	48.2220762	3.47686859	0.892904	3.893888	9.87E-05	0.000703
MUC17	32.6483311	6.075097075	1.126717	5.391859	6.97E-08	1.11E-06
SERPINE1	27131.2282	-8.745068771	0.397656	-21.9915	3.47E-107	1.96E-103
AP1S1	4162.79768	-3.174696874	0.403347	-7.87088	3.52E-15	1.59E-13
PLOD3	3695.95074	-2.398181343	0.335419	-7.1498	8.69E-13	2.97E-11
SLC26A5	10.9091359	4.179411771	1.265451	3.302706	0.000958	0.004688
RELN	897.325907	2.379542414	0.342051	6.956695	3.48E-12	1.10E-10
LHFPL3	18.728569	5.152460706	1.196239	4.307217	1.65E-05	0.000149
CDHR3	102.698563	3.633512932	0.635638	5.716324	1.09E-08	2.02E-07
PRKAR2B	618.072709	3.466377845	0.36865	9.402895	5.31E-21	4.26E-19
SLC26A4	49.1322034	3.101954457	0.784674	3.953177	7.71E-05	0.000567
SLC26A3	16.7738421	4.258077532	1.177274	3.616896	0.000298	0.001788
AC004014.2	18.6260693	5.004139642	1.212623	4.126708	3.68E-05	0.000298
LRRN3	29.9201433	3.282608366	0.966469	3.396495	0.000683	0.003543
AC002463.1	21.0041138	5.251088729	1.193062	4.401353	1.08E-05	0.000102
GPR85	65.6451701	2.728729045	0.649427	4.201749	2.65E-05	0.000224
TES	4026.70622	-2.053714302	0.323885	-6.34087	2.28E-10	5.60E-09
CAV1	1533.29971	-3.69948901	0.330407	-11.1968	4.23E-29	5.80E-27
ST7-AS2	10.9091359	4.179411771	1.265451	3.302706	0.000958	0.004688
CFTR	48.1944131	3.449481792	0.785097	4.393702	1.11E-05	0.000105
ASZ1	13.131963	4.556748427	1.238911	3.678026	0.000235	0.001464
KCND2	78.5867468	7.482215622	1.024248	7.305079	2.77E-13	1.02E-11
AC074085.2	9.21345487	3.996448214	1.275045	3.134359	0.001722	0.007574
PTPRZ1	316.009779	5.78118247	0.489216	11.81724	3.18E-32	5.09E-30
AC004594.1	10.2501954	3.994010754	1.278276	3.124529	0.001781	0.007777
SLC13A1	15.0326435	4.668138074	1.235019	3.779812	0.000157	0.001042
IQUB	90.9690728	2.867217561	0.598627	4.789659	1.67E-06	1.95E-05
ASB15	13.1056041	4.607910412	1.233642	3.735209	0.000188	0.001214
SPAM1	9.56489611	3.948402431	1.27948	3.085944	0.002029	0.008665
AC006148.1	55.1684037	5.37796298	0.960675	5.598111	2.17E-08	3.80E-07

POT1-AS1	93.3805322	2.994372763	0.597937	5.007839	5.50E-07	7.28E-06
GRM8	27.0136546	5.737159152	1.153718	4.972756	6.60E-07	8.54E-06
LEP	8.85323788	3.929277279	1.278481	3.073395	0.002116	0.008948
IMPDH1	1083.05862	-2.015199776	0.362218	-5.5635	2.64E-08	4.56E-07
AC018638.5	180.648396	-2.161297954	0.475361	-4.54665	5.45E-06	5.56E-05
FLNC	20278.4709	-2.90016685	0.337489	-8.59337	8.45E-18	5.04E-16
STRIP2	2499.91949	2.680372005	0.326839	8.200905	2.39E-16	1.25E-14
UBE2H	6590.86093	-2.49538846	0.30999	-8.04991	8.29E-16	4.11E-14
CPA4	15481.8371	-9.081479065	0.427839	-21.2264	5.45E-100	1.85E-96
MEST	4638.74367	-2.886700203	0.364092	-7.92849	2.22E-15	1.04E-13
AUXG01000058.1	168.799087	-2.162793181	0.540223	-4.00352	6.24E-05	0.000473
LINC-PINT	65.920957	2.015682128	0.6425	3.13725	0.001705	0.007522
AC009362.1	17.0182406	5.055804889	1.201815	4.206807	2.59E-05	0.00022
EEF1B2P6	75.2526957	2.591038759	0.634287	4.084964	4.41E-05	0.000349
AKR1B1	11594.0551	-3.484160644	0.3758	-9.27132	1.84E-20	1.41E-18
AKR1B15	13.3516098	4.59562907	1.235878	3.718512	0.0002	0.001281
CALD1	20191.1617	-3.105388249	0.30313	-10.2444	1.25E-24	1.28E-22
SLC23A4P	17.8616933	5.147739868	1.193416	4.31345	1.61E-05	0.000145
AC078845.1	11.2576413	4.313999964	1.254792	3.438019	0.000586	0.003119
AC009264.1	20.1899559	5.160546469	1.200648	4.298133	1.72E-05	0.000154
CHRM2	30.1856168	4.863680994	1.036986	4.690211	2.73E-06	3.01E-05
PTN	2614.80334	6.172485395	0.420296	14.68604	7.92E-49	3.53E-46
DGKI	123.506584	2.281745188	0.51916	4.395071	1.11E-05	0.000105
AKR1D1	9.00845539	3.949293107	1.277459	3.091523	0.001991	0.008536
PTMAP10	59.8458856	5.113529519	0.803569	6.36352	1.97E-10	4.88E-09
SLC37A3	610.8336	-2.195448208	0.358588	-6.12248	9.21E-10	2.04E-08
AC006452.1	19.554964	3.45807456	1.12582	3.071604	0.002129	0.008981
AC005692.3	14.063268	4.542184092	1.243891	3.651593	0.000261	0.001594
WEE2-AS1	31.9704874	3.306719417	0.893104	3.702503	0.000213	0.001349
WEE2	13.1846808	4.268440808	1.265487	3.372963	0.000744	0.003804
MGAM2	35.3533121	4.70688507	1.018191	4.62279	3.79E-06	4.02E-05
TRPV6	35.3993944	3.874001524	0.964475	4.016696	5.90E-05	0.00045
KEL	20.0610973	5.057206537	1.211654	4.173805	3.00E-05	0.000249
TMEM139	99.5214557	-2.735567667	0.57141	-4.7874	1.69E-06	1.96E-05
CLCN1	19.6485683	3.884251549	1.127172	3.446016	0.000569	0.003049
FAM131B	1986.45031	-2.410803676	0.329642	-7.3134	2.60E-13	9.58E-12
AC093673.1	446.99455	-2.720607013	0.410234	-6.63184	3.32E-11	9.31E-10
ZYX	13935.6417	-3.762386735	0.309735	-12.1471	5.94E-34	1.07E-31
EPHA1-AS1	14.1276973	4.598912982	1.238579	3.713055	0.000205	0.001303
OR10AC1	13.3135394	4.460011267	1.248997	3.570873	0.000356	0.002065
OR2F1	11.8785114	4.334365593	1.255325	3.452784	0.000555	0.002989
OR6B1	13.6620448	4.602670925	1.236404	3.722627	0.000197	0.001267
OR2A5	24.5975396	5.486096555	1.177227	4.660187	3.16E-06	3.43E-05
OR2A12	13.6884037	4.501752988	1.246408	3.611783	0.000304	0.001818

OR2A3P	8.85323788	3.929277279	1.278481	3.073395	0.002116	0.008948
CNTNAP2	63.7398339	3.64559375	0.815421	4.470814	7.79E-06	7.63E-05
AC004941.1	9.51217834	4.071977747	1.269403	3.20779	0.001338	0.006166
SSPOP	290.131507	2.182444433	0.479751	4.549115	5.39E-06	5.51E-05
AC004877.1	27.1719437	3.106409021	0.918731	3.381195	0.000722	0.003709
AC005586.1	51.6394257	2.554380483	0.76535	3.337531	0.000845	0.004225
RARRES2	22612.7449	3.877660163	0.407429	9.517399	1.78E-21	1.48E-19
GIMAP8	13.8436212	4.487620238	1.248294	3.595004	0.000324	0.001923
GIMAP6	9.82261335	4.112078605	1.267155	3.245126	0.001174	0.005514
GIMAP2	9.38331971	4.017150993	1.273395	3.154678	0.001607	0.00717
GIMAP1	12.1362286	4.476236461	1.242888	3.60148	0.000316	0.001882
TMEM176B	98.8151472	6.595870133	0.874511	7.542355	4.62E-14	1.86E-12
TMEM176A	108.868713	7.859894614	1.010372	7.779205	7.30E-15	3.15E-13
IQCA1L	10.4434834	4.166374392	1.26483	3.29402	0.000988	0.004787
CHPF2	5108.21256	-2.879151951	0.308335	-9.33775	9.84E-21	7.67E-19
NUB1	1466.74795	-2.008287371	0.330512	-6.0763	1.23E-09	2.67E-08
WDR86	2690.89096	3.140189285	0.339148	9.259044	2.06E-20	1.57E-18
WDR86-AS1	147.122707	3.925254481	0.555876	7.06139	1.65E-12	5.44E-11
AC005996.1	19.3883211	3.286863833	1.079545	3.044676	0.002329	0.009685
CRYGN	100.494878	3.664069496	0.597545	6.131875	8.68E-10	1.94E-08
PRKAG2	2117.7217	-2.11860428	0.323434	-6.55034	5.74E-11	1.55E-09
LINC01287	28.8296655	4.707895698	1.069217	4.403125	1.07E-05	0.000101
DPP6	267.34665	6.896416054	0.603528	11.42683	3.07E-30	4.43E-28
AC006019.1	17.1002531	4.969940569	1.210807	4.10465	4.05E-05	0.000325
AC008060.1	9.62932542	4.002668209	1.275446	3.138251	0.0017	0.0075
AC008060.5	10.0949779	4.005107431	1.276891	3.136609	0.001709	0.007522
MNX1	14.7866378	4.723776122	1.228387	3.845512	0.00012	0.000832
DNAJB6	5093.71666	-2.836972251	0.320556	-8.85015	8.74E-19	5.75E-17
AC006372.3	16.489766	4.171279073	1.190694	3.503232	0.00046	0.002562
PTPRN2	68.7297798	4.090735614	0.69578	5.879348	4.12E-09	8.23E-08
PLCXD1	5831.88711	2.464426038	0.308408	7.990803	1.34E-15	6.51E-14
SHOX	17.4106881	4.965599823	1.212466	4.095453	4.21E-05	0.000335
CRLF2	10.5342716	4.124847874	1.268711	3.25121	0.001149	0.005437
CSF2RA	20.330526	5.348450313	1.178922	4.536731	5.71E-06	5.79E-05
LINC00106	137.047597	-2.109737918	0.515662	-4.09132	4.29E-05	0.000341
ASMTL-AS1	264.437277	-2.516056993	0.481239	-5.22829	1.71E-07	2.51E-06
AC006062.1	340.078346	-5.753681907	0.569437	-10.1042	5.30E-24	5.25E-22
FAM9B	17.4106881	4.965599823	1.212466	4.095453	4.21E-05	0.000335
FRMPD4	37.7096681	4.552356541	0.935723	4.865068	1.14E-06	1.39E-05
TMSB4X	49361.829	-2.140511426	0.416501	-5.13927	2.76E-07	3.89E-06
LINC02154	620.48315	-7.152194032	0.512816	-13.9469	3.29E-44	1.09E-41
LINC01203	171.195089	-3.497051745	0.547783	-6.38401	1.73E-10	4.30E-09
EGFL6	15.9290609	3.837928704	1.142492	3.359261	0.000782	0.00397
GPM6B	141.697756	3.795773368	0.534759	7.098101	1.26E-12	4.25E-11

ACE2	11.9692996	4.268166641	1.261583	3.383183	0.000717	0.003686
CLTRN	112.510417	5.519953685	0.692076	7.975935	1.51E-15	7.27E-14
SCML1	172.958106	2.056972828	0.501945	4.098001	4.17E-05	0.000333
RAI2	28.2866793	2.877143514	0.881376	3.264377	0.001097	0.005228
BEND2	9.75818404	4.081877292	1.269445	3.215482	0.001302	0.006028
AL807742.1	12.3822343	4.464991456	1.24496	3.586452	0.000335	0.001968
KLHL34	63.8890516	4.99969999	0.775876	6.443945	1.16E-10	2.99E-09
DDX53	10.0305486	3.916288499	1.283419	3.051449	0.002277	0.0095
RPL9P7	176.867432	2.220324688	0.533644	4.160687	3.17E-05	0.000262
KLHL15	151.156651	2.007640365	0.48463	4.142624	3.43E-05	0.000281
PCYT1B	52.5447617	2.155978974	0.690189	3.12375	0.001786	0.007795
GK	90.3560642	2.499441633	0.595964	4.193945	2.74E-05	0.00023
AL591501.1	22.7876473	5.408693913	1.180539	4.581547	4.62E-06	4.80E-05
CFAP47	34.4816465	6.235017787	1.110241	5.615915	1.96E-08	3.46E-07
XK	78.7206985	3.738955013	0.652591	5.729397	1.01E-08	1.87E-07
TSPAN7	433.925285	4.427167568	0.544077	8.137031	4.05E-16	2.07E-14
GAPDHP1	299.175564	-3.103939192	0.452174	-6.86448	6.67E-12	2.03E-10
RPS2P55	419.889168	-2.65652846	0.455299	-5.83469	5.39E-09	1.05E-07
NYX	195.673985	6.782383565	0.671371	10.10229	5.40E-24	5.33E-22
MAOA	436.389513	3.590539118	0.43216	8.308365	9.70E-17	5.24E-15
MAOB	127.166026	2.126168872	0.543461	3.912277	9.14E-05	0.000657
EFHC2	73.7735422	3.323834447	0.644079	5.160604	2.46E-07	3.51E-06
PCSK1N	2688.41436	2.826233584	0.409413	6.903133	5.09E-12	1.57E-10
OTUD5	2695.09063	-2.065645748	0.323688	-6.3816	1.75E-10	4.37E-09
KCND1	159.29413	-2.221750678	0.516427	-4.30216	1.69E-05	0.000151
TFE3	2911.93809	-2.773840067	0.340454	-8.14748	3.72E-16	1.91E-14
SYP	132.815394	2.528284967	0.562257	4.496674	6.90E-06	6.85E-05
CACNA1F	30.6568624	6.026913725	1.127093	5.347306	8.93E-08	1.39E-06
DGKK	15.1351432	4.823231696	1.219187	3.956105	7.62E-05	0.00056
LINC01284	10.7802773	4.027023489	1.277437	3.152423	0.001619	0.007214
AL158055.1	31.2973054	3.047912593	0.856005	3.560624	0.00037	0.00214
MAGED4	100.812126	2.739518358	0.667642	4.103273	4.07E-05	0.000326
MAGEH1	2493.25751	3.271992692	0.358704	9.121706	7.40E-20	5.46E-18
USP51	153.962558	3.449842609	0.511144	6.749253	1.49E-11	4.35E-10
AL050309.1	30.9131007	3.338290845	0.895614	3.727376	0.000193	0.001247
KLF8	34.2663448	2.698991298	0.827194	3.262828	0.001103	0.005253
FAAH2	36.9394205	4.583945225	0.916281	5.002771	5.65E-07	7.45E-06
MTMR8	47.9699935	2.427634773	0.770702	3.149899	0.001633	0.007271
MSN	11623.978	-3.156069489	0.309118	-10.2099	1.79E-24	1.83E-22
EDA2R	427.675579	-2.492788078	0.387877	-6.42675	1.30E-10	3.32E-09
EFNB1	1195.84736	-2.668594128	0.335141	-7.96259	1.68E-15	8.03E-14
RPS23P8	176.603945	4.47174651	0.564429	7.922597	2.33E-15	1.08E-13
NHSL2	151.866822	4.401342126	0.566973	7.762881	8.30E-15	3.57E-13
RTL5	633.673974	2.438105904	0.371758	6.558319	5.44E-11	1.48E-09

NAP1L2	70.8568699	4.404504528	0.757121	5.817436	5.98E-09	1.16E-07
TSIX	38.1749148	5.142102908	1.03023	4.99122	6.00E-07	7.84E-06
XIST	666.742814	10.26794667	0.903098	11.3697	5.92E-30	8.39E-28
NEXMIF	17.5922645	4.838231597	1.226637	3.944305	8.00E-05	0.000585
ZDHHC15	78.3368213	3.627147001	0.726605	4.991913	5.98E-07	7.82E-06
MIR325HG	11.0643534	4.176955156	1.266227	3.298742	0.000971	0.004726
PGK1	11965.7395	-2.525788663	0.346002	-7.29993	2.88E-13	1.05E-11
PGAM4	79.5609978	-2.157908088	0.60318	-3.57755	0.000347	0.002027
HMGN5	631.644707	2.116346803	0.356484	5.936727	2.91E-09	5.99E-08
RPS6KA6	232.411362	2.264585528	0.4339	5.219137	1.80E-07	2.63E-06
SATL1	22.7470469	4.314082363	1.053549	4.094808	4.23E-05	0.000336
DACH2	11.1024238	4.303371118	1.255163	3.428536	0.000607	0.003214
KLHL4	569.113992	-2.039061379	0.380652	-5.35675	8.47E-08	1.32E-06
RPSAP15	339.886144	-2.955607934	0.490961	-6.02005	1.74E-09	3.71E-08
PCDH11X	18.3156342	5.021387416	1.209627	4.151185	3.31E-05	0.000271
KRT18P11	74.6795176	-4.422841391	0.716926	-6.16918	6.86E-10	1.56E-08
NAP1L3	673.307099	2.33758953	0.404842	5.774076	7.74E-09	1.47E-07
DIAPH2-AS1	21.001178	5.458578537	1.167964	4.673585	2.96E-06	3.24E-05
SRPX2	425.320221	-4.697301395	0.497126	-9.44892	3.42E-21	2.79E-19
ZMAT1	184.782263	2.43414432	0.499449	4.873662	1.10E-06	1.34E-05
TCEAL2	41.4693385	6.535260392	1.087997	6.006691	1.89E-09	4.01E-08
BEX5	75.5688092	4.847526689	0.757832	6.396571	1.59E-10	4.00E-09
GPRASP1	285.65264	2.238017818	0.427809	5.231346	1.68E-07	2.48E-06
TCEAL5	103.023444	6.82000543	0.85497	7.976891	1.50E-15	7.22E-14
PLP1	22.2575655	5.388374503	1.181148	4.561979	5.07E-06	5.21E-05
NRK	284.659356	-2.044893146	0.426799	-4.79123	1.66E-06	1.93E-05
RADX	151.808926	2.962814301	0.546998	5.416501	6.08E-08	9.79E-07
MORC4	964.172763	-2.722886502	0.340478	-7.99724	1.27E-15	6.19E-14
CLDN2	190.766203	2.63198826	0.498734	5.277339	1.31E-07	1.97E-06
NUP62CL	25.0638449	3.955763286	0.987924	4.004116	6.22E-05	0.000472
PRPS1	3024.93202	-2.308688524	0.364238	-6.33841	2.32E-10	5.68E-09
AL928646.1	18.9101454	5.085147585	1.204649	4.221268	2.43E-05	0.000208
ACSL4	2209.79947	-3.052809912	0.318757	-9.57723	9.97E-22	8.49E-20
CHRDL1	600.924417	-4.144500169	0.501646	-8.2618	1.43E-16	7.67E-15
CAPN6	273.441074	-3.912140573	0.585447	-6.68232	2.35E-11	6.73E-10
DCX	64.7611145	6.063526257	0.882825	6.868323	6.50E-12	1.97E-10
TRPC5	11.0643534	4.176955156	1.266227	3.298742	0.000971	0.004726
XACT	445.403433	6.701593715	1.156017	5.797141	6.75E-09	1.29E-07
HTR2C	12.4993814	4.297396672	1.260864	3.408295	0.000654	0.003427
IL13RA2	109.54809	2.506129461	0.590692	4.242701	2.21E-05	0.000192
LINC01285	17.8033825	4.007734558	1.126369	3.5581	0.000374	0.002156
KIAA1210	15.5744369	4.886440714	1.214046	4.024922	5.70E-05	0.000437
SOWAHD	56.9795061	-2.321755226	0.707319	-3.28247	0.001029	0.004949
TMEM255A	704.670382	2.435624892	0.400115	6.087314	1.15E-09	2.51E-08

GLUD2	142.707455	3.160564128	0.511346	6.180875	6.37E-10	1.45E-08
GRIA3	235.168062	2.797240567	0.437617	6.391977	1.64E-10	4.10E-09
AL121601.1	9.02016693	3.989611823	1.274622	3.130036	0.001748	0.007669
TENM1	103.566927	3.047799699	0.58806	5.182806	2.19E-07	3.15E-06
ELF4	1151.46665	-2.417831306	0.334712	-7.22362	5.06E-13	1.79E-11
Z82195.2	23.9503107	5.553343813	1.166733	4.759738	1.94E-06	2.21E-05
IGSF1	93.0230361	3.047159866	0.593244	5.136435	2.80E-07	3.94E-06
GPC4	905.707309	-2.457817745	0.342512	-7.17586	7.19E-13	2.49E-11
AC002407.1	22.2546297	5.556978148	1.160275	4.789362	1.67E-06	1.95E-05
CCDC160	95.4146157	2.709634802	0.582024	4.655542	3.23E-06	3.49E-05
SMIM10L2A	162.634719	4.330674747	0.529818	8.173889	2.99E-16	1.54E-14
SAGE4P	10.5342716	4.124847874	1.268711	3.25121	0.001149	0.005437
GPR101	12.1625875	4.422982886	1.248134	3.543676	0.000395	0.002253
FGF13	217.33137	4.272080638	0.543756	7.856619	3.95E-15	1.76E-13
FGF13-AS1	56.8100067	5.355024003	0.851331	6.290182	3.17E-10	7.55E-09
MCF2	111.282149	4.334177387	0.630925	6.869562	6.44E-12	1.96E-10
LINC00632	88.9331987	3.315697701	0.60229	5.505147	3.69E-08	6.19E-07
SLITRK2	108.622739	7.932078563	0.998295	7.945622	1.93E-15	9.15E-14
AC016925.3	30.2560449	4.806101941	0.999922	4.806479	1.54E-06	1.81E-05
PASD1	9.62932542	4.002668209	1.275446	3.138251	0.0017	0.0075
PRRG3	23.6281641	5.415111023	1.182595	4.579008	4.67E-06	4.84E-05
BGN	590.285415	-5.051202271	0.411475	-12.2758	1.22E-34	2.31E-32
ABCD1	863.473997	-2.05417239	0.351561	-5.843	5.13E-09	1.00E-07
PLXNB3	2126.22216	-2.607445787	0.376112	-6.93263	4.13E-12	1.29E-10
L1CAM	2513.86481	-4.712597966	0.325686	-14.4698	1.88E-47	7.68E-45
IRAK1	5173.32137	-3.268601696	0.310043	-10.5424	5.51E-26	6.08E-24
FLNA	65676.9319	-3.987517114	0.314372	-12.6841	7.25E-37	1.54E-34
DNASE1L1	407.297607	-3.05918957	0.399568	-7.65625	1.91E-14	7.96E-13
PLXNA3	1804.4742	-3.309976564	0.373731	-8.85658	8.25E-19	5.44E-17
SLC10A3	559.317244	-2.353334926	0.366087	-6.42835	1.29E-10	3.29E-09
G6PD	2447.21482	-3.949646229	0.383987	-10.2859	8.16E-25	8.51E-23
F8A1	73.194155	-2.67308449	0.657508	-4.06548	4.79E-05	0.000376
RAB39B	108.692884	3.561096635	0.574003	6.203974	5.51E-10	1.27E-08
DLGAP2	64.7713788	4.064730033	0.86484	4.699977	2.60E-06	2.88E-05
AC246817.1	22.5035712	5.347605094	1.186874	4.505621	6.62E-06	6.59E-05
CSMD1	75.5741478	5.201170149	0.826799	6.290732	3.16E-10	7.53E-09
AC027251.1	15.1149029	3.741440246	1.154394	3.241043	0.001191	0.005585
AC018398.2	12.8478869	4.474722687	1.24585	3.591704	0.000329	0.001942
ZNF705G	13.5331862	4.511043366	1.24495	3.623474	0.000291	0.001749
PRAG1	1487.85107	-4.560194197	0.35517	-12.8395	9.85E-38	2.20E-35
CLDN23	64.7733602	2.854379992	0.723552	3.944954	7.98E-05	0.000584
MIR124-1HG	21.1066136	5.36488684	1.179834	4.547154	5.44E-06	5.55E-05
PRSS55	11.9048703	4.177926253	1.269005	3.292284	0.000994	0.004813
RP1L1	30.1794983	5.963288111	1.134168	5.257853	1.46E-07	2.17E-06

C8orf74	22.1287068	5.324285971	1.188358	4.480372	7.45E-06	7.33E-05
AC022239.1	12.7968399	3.733708082	1.202441	3.105107	0.001902	0.008205
AC068587.4	40.0448694	2.708120887	0.874672	3.096157	0.00196	0.008422
TRMT9B	49.4779625	3.957722683	0.79501	4.978207	6.42E-07	8.34E-06
DLC1	2665.32958	-3.598054644	0.33711	-10.6732	1.36E-26	1.58E-24
SGCZ	24.2607457	5.55261194	1.167906	4.75433	1.99E-06	2.26E-05
MSR1	27.8805304	5.697638263	1.161524	4.905311	9.33E-07	1.16E-05
AC011586.2	11.4128589	4.32259367	1.254608	3.445374	0.00057	0.003052
FGF20	12.7834575	4.424631592	1.250368	3.538662	0.000402	0.002286
SLC7A2	1385.72367	6.45456592	0.379001	17.03049	4.88E-65	4.13E-62
MTUS1	355.200045	2.187597096	0.40012	5.467357	4.57E-08	7.55E-07
AC027117.2	11.8902229	4.469810761	1.242571	3.597228	0.000322	0.001909
AC027117.1	50.6005425	4.478477269	0.796312	5.62402	1.87E-08	3.31E-07
FGL1	9.75818404	4.081877292	1.269445	3.215482	0.001302	0.006028
ASAH1	11403.2523	2.530374608	0.338998	7.464282	8.38E-14	3.28E-12
SH2D4A	1093.4944	-3.885188453	0.356081	-10.911	1.02E-27	1.29E-25
LPL	112.723374	4.354010415	0.608826	7.151481	8.58E-13	2.94E-11
SLC18A1	11.6705761	4.433451561	1.24521	3.560405	0.00037	0.00214
AC021613.1	42.5420178	4.300115935	0.971473	4.426388	9.58E-06	9.17E-05
DMTN	122.727045	-2.333730736	0.572675	-4.07514	4.60E-05	0.000363
NUDT18	553.115486	-2.046140429	0.428178	-4.77871	1.76E-06	2.04E-05
BMP1	3707.36007	-3.857871248	0.324999	-11.8704	1.69E-32	2.76E-30
PIWIL2	45.2584404	3.342639321	0.779891	4.286031	1.82E-05	0.000161
C8orf58	289.045762	-2.041638374	0.507806	-4.02051	5.81E-05	0.000444
AC105046.1	14.1276973	4.598912982	1.238579	3.713055	0.000205	0.001303
EGR3	20.8606079	5.379585169	1.17711	4.570162	4.87E-06	5.03E-05
AC037441.1	11.5036471	4.283732053	1.258499	3.403843	0.000664	0.003466
TNFRSF10B	4415.56263	-2.078607864	0.315888	-6.58021	4.70E-11	1.29E-09
TNFRSF10D	2727.00895	-4.508952453	0.321461	-14.0264	1.07E-44	3.64E-42
LOXL2	13288.1687	-7.020831794	0.326448	-21.5067	1.35E-102	5.71E-99
ENTPD4	2890.38572	-2.149024877	0.314127	-6.84126	7.85E-12	2.36E-10
STC1	1065.44087	-2.966183984	0.342513	-8.66007	4.71E-18	2.88E-16
ADAM28	41.9341965	3.341403503	0.835094	4.00123	6.30E-05	0.000476
ADAM7	13.752833	4.558390099	1.241168	3.672661	0.00024	0.001487
DOCK5	1147.12717	-2.301880203	0.385499	-5.97117	2.36E-09	4.92E-08
EBF2	28.4750415	5.738288086	1.158229	4.954364	7.26E-07	9.29E-06
ADRA1A	28.8235469	5.807468849	1.150454	5.047981	4.47E-07	6.05E-06
PTK2B	1131.08551	2.680518442	0.333379	8.040446	8.95E-16	4.42E-14
CHRNA2	18.1604167	5.026847727	1.208454	4.159736	3.19E-05	0.000263
CLU	14413.8763	2.256925175	0.418436	5.393719	6.90E-08	1.10E-06
AC013643.3	13.3135394	4.460011267	1.248997	3.570873	0.000356	0.002065
SCARA5	14.6314203	4.722293368	1.227933	3.845727	0.00012	0.000832
FZD3	603.643708	2.439496811	0.387638	6.293241	3.11E-10	7.43E-09
DUSP4	6357.60849	3.139634615	0.306291	10.25049	1.18E-24	1.21E-22

LINC02099	16.1308776	4.8690777	1.218154	3.997097	6.41E-05	0.000482
LINC02209	10.7275595	4.247693306	1.258756	3.374517	0.000739	0.003787
RBPMS-AS1	70.5396441	2.048868184	0.663486	3.088034	0.002015	0.008627
TEX15	49.3063547	6.761591787	1.074159	6.29478	3.08E-10	7.36E-09
AC068672.2	28.7796049	5.975177462	1.128508	5.294758	1.19E-07	1.81E-06
DUSP26	153.415354	4.18734609	0.570845	7.335351	2.21E-13	8.19E-12
AC087343.1	33.6006489	4.509736691	0.951307	4.740568	2.13E-06	2.41E-05
LSM12P1	138.878087	-2.839046901	0.539702	-5.2604	1.44E-07	2.14E-06
AC124290.1	39.513036	6.271030309	1.119245	5.602912	2.11E-08	3.71E-07
AC090809.1	16.7517476	4.85108935	1.222407	3.968473	7.23E-05	0.000536
AC091182.1	14.7983493	4.842510208	1.215751	3.983144	6.80E-05	0.000508
LINC01605	58.9826229	-2.696869955	0.723043	-3.72989	0.000192	0.001237
ZNF703	2830.8084	-2.66100928	0.320826	-8.29424	1.09E-16	5.88E-15
C8orf86	9.78454293	4.008050513	1.275572	3.14216	0.001677	0.007433
AC069120.1	42.5568094	2.605638005	0.77461	3.363805	0.000769	0.003915
TACC1	5491.96435	2.815718901	0.323143	8.713544	2.95E-18	1.85E-16
ADAM3A	12.8478869	4.474722687	1.24585	3.591704	0.000329	0.001942
IDO1	12.8699813	3.870463439	1.207164	3.206244	0.001345	0.006194
IDO2	12.511093	4.525118401	1.239576	3.650538	0.000262	0.0016
TCIM	52.2091424	-2.872552302	0.785817	-3.6555	0.000257	0.001574
NKX6-3	12.7570987	4.511683948	1.241888	3.632924	0.00028	0.001694
ANK1	379.25405	2.92204389	0.395182	7.394178	1.42E-13	5.42E-12
PLAT	1631.3688	-4.724453835	0.363412	-13.0003	1.22E-38	2.99E-36
HGSNAT	832.476908	-2.25027796	0.368947	-6.09919	1.07E-09	2.34E-08
LINC00293	31.356809	5.946450369	1.140064	5.21589	1.83E-07	2.67E-06
AC120036.1	12.1245171	4.251364794	1.263572	3.36456	0.000767	0.003905
AC120036.4	17.3169641	5.141778599	1.192336	4.312357	1.62E-05	0.000146
EFCAB1	58.9367387	4.854652745	0.799451	6.072481	1.26E-09	2.73E-08
SNTG1	61.4379217	4.624720609	0.810532	5.705786	1.16E-08	2.13E-07
ST18	47.5284705	3.231250206	0.87823	3.679277	0.000234	0.001458
NPBWR1	118.095544	-2.56535456	0.55302	-4.63881	3.50E-06	3.75E-05
OPRK1	15.8091368	3.581425122	1.148854	3.117388	0.001825	0.007928
AC084834.1	15.3167196	4.759284449	1.226727	3.879662	0.000105	0.000737
SBF1P1	18.2309646	3.930239579	1.152976	3.408777	0.000653	0.003427
SDR16C5	18.5733515	5.150495683	1.195872	4.306897	1.66E-05	0.000149
AC012349.1	14.256556	4.682592502	1.230583	3.805184	0.000142	0.000958
AC009597.1	10.0305486	3.916288499	1.283419	3.051449	0.002277	0.0095
LINC01606	41.0190177	4.660729267	0.932968	4.995596	5.87E-07	7.70E-06
AC025674.2	11.8785114	4.334365593	1.255325	3.452784	0.000555	0.002989
CYP7A1	10.4698423	4.061968818	1.273658	3.189215	0.001427	0.006475
AC021393.1	16.0664483	4.83022072	1.222086	3.95244	7.74E-05	0.000568
AC068389.1	22.9140956	3.831934977	1.051794	3.643238	0.000269	0.00164
CLVS1	14.8129967	4.619407683	1.239067	3.728133	0.000193	0.001244
KRT8P3	164.424173	-2.828562684	0.497155	-5.6895	1.27E-08	2.33E-07

AC023095.1	10.6367713	4.260259526	1.257293	3.388439	0.000703	0.00363
NKAIN3	90.0064036	2.900068752	0.732073	3.96145	7.45E-05	0.000549
AC090577.1	10.2882659	4.156236444	1.265118	3.285256	0.001019	0.004906
AC011124.1	12.4085932	4.379167019	1.253201	3.494385	0.000475	0.002618
AC011124.2	11.4772882	4.356824519	1.251683	3.480772	0.0005	0.002731
LINC01414	9.09924358	3.9280641	1.279421	3.07019	0.002139	0.009014
LINC01289	12.2270168	4.457657865	1.245054	3.580294	0.000343	0.002009
AC090136.3	13.3135394	4.460011267	1.248997	3.570873	0.000356	0.002065
AC104232.3	10.6894891	4.123736642	1.269366	3.248658	0.00116	0.005468
LINC00251	13.4687569	4.446583579	1.250803	3.554982	0.000378	0.002179
LINC01299	10.6894891	4.123736642	1.269366	3.248658	0.00116	0.005468
DNAJC5B	16.5965301	4.858850005	1.221017	3.979348	6.91E-05	0.000515
RRS1-AS1	23.2865888	3.303720451	0.991598	3.331712	0.000863	0.0043
SGK3	500.458082	3.142681273	0.439024	7.158332	8.17E-13	2.81E-11
MCMDC2	93.4083945	3.324963916	0.58835	5.651338	1.59E-08	2.86E-07
PPP1R42	27.403819	4.124470244	0.958876	4.301358	1.70E-05	0.000152
PREX2	32.6102606	6.029284152	1.132792	5.3225	1.02E-07	1.57E-06
C8orf34	53.7444114	4.475085036	0.79389	5.636908	1.73E-08	3.09E-07
SLCO5A1	30.2927953	2.923135078	0.914039	3.198043	0.001384	0.006341
PRDM14	9.25446109	3.938692024	1.279145	3.07916	0.002076	0.008797
AC022730.4	9.00845539	3.949293107	1.277459	3.091523	0.001991	0.008536
EYA1	41.3006921	2.988564125	0.869055	3.438866	0.000584	0.003113
MSC	187.664633	-3.442166393	0.485148	-7.09509	1.29E-12	4.33E-11
TRPA1	21.230285	3.629543204	1.037478	3.49843	0.000468	0.00259
AC022905.1	14.0369091	4.644853865	1.233596	3.765297	0.000166	0.001098
AC022893.2	10.1974777	4.174297772	1.263219	3.304494	0.000951	0.004675
SBSPON	514.821325	-2.454002638	0.373775	-6.56546	5.19E-11	1.41E-09
MIR2052HG	14.7222085	4.681425272	1.232515	3.798272	0.000146	0.000979
PI15	19.1034333	5.181849806	1.194228	4.339081	1.43E-05	0.000131
CRISPLD1	8387.1463	5.138755727	0.347266	14.79776	1.51E-49	7.13E-47
CASC9	19.8592805	4.11515896	1.127031	3.651327	0.000261	0.001596
LINC01109	10.5342716	4.124847874	1.268711	3.25121	0.001149	0.005437
STMN2	18.0230293	4.034090188	1.122501	3.593842	0.000326	0.001931
AC036214.1	14.7485674	4.543772542	1.246109	3.646367	0.000266	0.001622
AC034114.2	16.7898181	4.969046522	1.209699	4.10767	4.00E-05	0.000321
RPSAP47	246.782429	-4.754213513	1.168809	-4.06757	4.75E-05	0.000373
ZNF704	67.4730905	2.652444611	0.698547	3.797088	0.000146	0.000983
PAG1	340.096542	2.84677366	0.401875	7.083737	1.40E-12	4.66E-11
PMP2	11.696935	4.398045908	1.248667	3.522193	0.000428	0.002409
AC060765.2	16.5965301	4.858850005	1.221017	3.979348	6.91E-05	0.000515
RALYL	20.6536039	3.236301089	1.007745	3.211429	0.001321	0.006099
LRRCC1	343.340358	3.716209225	0.431166	8.618978	6.76E-18	4.08E-16
CA1	10.0041897	4.063829218	1.271846	3.195222	0.001397	0.006372
CA3-AS1	38.5889081	3.890441535	0.848427	4.585473	4.53E-06	4.72E-05

CA2	115.763224	4.300779531	0.655199	6.564082	5.24E-11	1.42E-09
REXO1L1P	85.009254	7.413507447	1.044185	7.099801	1.25E-12	4.20E-11
CNGB3	358.953432	8.055333076	0.67851	11.87209	1.65E-32	2.72E-30
IARS2P1	8.85323788	3.929277279	1.278481	3.073395	0.002116	0.008948
CALB1	30.9047222	4.613797631	0.995328	4.635455	3.56E-06	3.81E-05
AC004083.1	19.8039692	3.576255304	1.056678	3.384432	0.000713	0.003672
LINC00534	38.3461082	5.609856277	1.055775	5.313494	1.08E-07	1.65E-06
NECAB1	540.7778	2.724845982	0.388444	7.014775	2.30E-12	7.43E-11
PIP4P2	415.876511	2.273793412	0.398194	5.71027	1.13E-08	2.08E-07
SLC26A7	50.2800997	3.265635285	0.742523	4.398028	1.09E-05	0.000103
RUNX1T1	113.21001	3.283091786	0.621489	5.282619	1.27E-07	1.92E-06
FLJ46284	21.3097675	3.123738716	1.026902	3.041907	0.002351	0.009758
C8orf87	10.8944886	4.333328375	1.251765	3.461774	0.000537	0.002907
FSBP	203.108817	2.234713162	0.451492	4.949619	7.44E-07	9.50E-06
GDF6	1534.41713	-6.847267337	0.455437	-15.0345	4.36E-51	2.35E-48
MATN2	4301.61579	-2.240602176	0.315355	-7.10501	1.20E-12	4.07E-11
ERICH5	1780.8787	-2.702950582	0.381454	-7.08591	1.38E-12	4.60E-11
POP1	520.209026	-2.394093317	0.372809	-6.42178	1.35E-10	3.43E-09
KCNS2	16.5965301	4.858850005	1.221017	3.979348	6.91E-05	0.000515
OSR2	15.6713436	3.735941404	1.16466	3.207752	0.001338	0.006166
COX6C	6659.78604	2.192535881	0.351396	6.239496	4.39E-10	1.03E-08
RGS22	35.3730355	3.905790351	0.948709	4.116954	3.84E-05	0.00031
FBXO43	42.2133715	2.958547094	0.776125	3.811946	0.000138	0.000936
RPS26P6	58.8406521	3.453775526	0.775832	4.451706	8.52E-06	8.26E-05
GRHL2	20.6819673	4.917701782	1.227765	4.00541	6.19E-05	0.00047
NCALD	197.476702	3.984542233	0.509315	7.823331	5.14E-15	2.26E-13
RPL5P24	16.5028061	5.059724344	1.198617	4.221302	2.43E-05	0.000208
BAALC	536.863004	7.235856001	0.523755	13.81534	2.06E-43	6.59E-41
CTHRC1	359.16136	3.484705271	0.443179	7.86297	3.75E-15	1.68E-13
RIMS2	298.219752	4.294457293	0.503223	8.533897	1.41E-17	8.28E-16
DPYS	109.715102	7.937205178	0.999227	7.943348	1.97E-15	9.30E-14
DCSTAMP	12.1245171	4.251364794	1.263572	3.36456	0.000767	0.003905
TAGLN2P1	115.08814	-2.855161778	0.55473	-5.14694	2.65E-07	3.75E-06
AC025508.1	12.4847341	4.555767595	1.236451	3.684551	0.000229	0.001433
RSPO2	16.0198491	3.821896172	1.148166	3.328697	0.000873	0.004335
TMEM74	34.395537	3.36655292	0.868399	3.876736	0.000106	0.000745
PKHD1L1	78.5318203	2.487237002	0.702024	3.542953	0.000396	0.002257
KCNV1	15.9173493	3.690366086	1.179381	3.129069	0.001754	0.007688
LINC01608	43.0457799	4.447332003	0.988748	4.497945	6.86E-06	6.81E-05
LINC01609	13.0938926	4.404207729	1.253385	3.513852	0.000442	0.002474
LINC02237	12.4730225	4.428932897	1.248782	3.546601	0.00039	0.002237
AARD	306.140574	5.637707563	0.534536	10.54693	5.25E-26	5.81E-24
SLC30A8	86.0600541	5.382245442	0.758009	7.100499	1.24E-12	4.19E-11
AC027419.2	18.5821272	5.222475319	1.188353	4.394718	1.11E-05	0.000105

EXT1	5040.45544	-2.760287902	0.307961	-8.9631	3.16E-19	2.19E-17
MAL2	1558.42861	-2.348622089	0.399518	-5.87864	4.14E-09	8.26E-08
AC021733.4	13.4160391	4.626058036	1.233038	3.751756	0.000176	0.001148
ENPP2	8889.13714	4.290509767	0.374684	11.45101	2.32E-30	3.38E-28
SNTB1	220.01325	2.351600104	0.483153	4.867191	1.13E-06	1.37E-05
HAS2-AS1	15.1995726	4.848958584	1.216648	3.985505	6.73E-05	0.000503
LINC01151	29.1603408	5.744739437	1.159479	4.954585	7.25E-07	9.29E-06
AC016405.3	55.0827881	3.55425764	0.720814	4.930891	8.19E-07	1.04E-05
ZHX1	972.603558	2.202794559	0.364856	6.037428	1.57E-09	3.36E-08
FBXO32	4232.36242	-4.539226374	0.314134	-14.45	2.51E-47	1.01E-44
ANXA13	24.816447	3.639664772	1.042292	3.491982	0.000479	0.002637
FER1L6	27.090321	4.011423484	1.055821	3.799342	0.000145	0.000976
FER1L6-AS2	10.0949779	4.005107431	1.276891	3.136609	0.001709	0.007522
MTSS1	3266.9189	9.148676087	0.452932	20.19877	1.00E-90	2.62E-87
AC100858.2	17.2554706	4.968540486	1.211556	4.100957	4.11E-05	0.000329
LINC00964	41.8764333	6.465046097	1.098434	5.885692	3.96E-09	7.94E-08
ZNF572	36.735719	3.196706758	0.868176	3.682097	0.000231	0.001445
AC091114.1	20.1846098	3.679611245	1.057873	3.47831	0.000505	0.002754
PCAT1	198.76571	2.284761137	0.456387	5.006194	5.55E-07	7.33E-06
CASC19	90.5932586	4.524742481	0.742454	6.094303	1.10E-09	2.41E-08
PRNCR1	27.8442029	2.977961615	0.941942	3.161511	0.00157	0.00703
AC104370.1	14.7602789	4.784725864	1.221827	3.916041	9.00E-05	0.000647
MYC	2333.4708	-3.696487897	0.341754	-10.8162	2.88E-27	3.53E-25
CCDC26	145.878259	5.350522179	0.754277	7.09358	1.31E-12	4.37E-11
HHLA1	18.0696285	5.064439282	1.203874	4.206784	2.59E-05	0.00022
KCNQ3	216.624515	2.705908139	0.495216	5.464097	4.65E-08	7.67E-07
LRRC6	125.099184	2.713352478	0.523039	5.187663	2.13E-07	3.08E-06
SLA	30.3935521	4.800910231	1.058437	4.535849	5.74E-06	5.81E-05
PTCSC1	12.4993814	4.297396672	1.260864	3.408295	0.000654	0.003427
CCN4	20.6028906	5.290566444	1.186956	4.457255	8.30E-06	8.07E-05
LINC02055	68.3488201	5.437676122	0.918842	5.917968	3.26E-09	6.63E-08
AC046195.2	10.2882659	4.156236444	1.265118	3.285256	0.001019	0.004906
FAM135B	203.794336	8.707032706	0.966776	9.006259	2.13E-19	1.50E-17
COL22A1	238.726829	3.133806391	0.448555	6.986455	2.82E-12	8.98E-11
KCNK9	12.9123162	4.516215643	1.242058	3.636073	0.000277	0.001678
C8orf17	10.4698423	4.061968818	1.273658	3.189215	0.001427	0.006475
DENND3	1307.70648	-2.990572066	0.331141	-9.03111	1.70E-19	1.20E-17
AC011676.5	9.38331971	4.017150993	1.273395	3.154678	0.001607	0.00717
PTP4A3	2562.65936	4.423125124	0.378477	11.68664	1.49E-31	2.35E-29
MROH5	17.8499817	5.032859884	1.206619	4.171043	3.03E-05	0.000251
AC100803.4	13.2461743	4.679044588	1.227217	3.812727	0.000137	0.000933
AC025839.1	27.0019431	5.597144353	1.171265	4.778718	1.76E-06	2.04E-05
AC103758.1	10.1330484	4.143717258	1.26561	3.274087	0.00106	0.005082
CYP11B1	14.6314203	4.722293368	1.227933	3.845727	0.00012	0.000832

LY6E	2634.98037	-3.007250687	0.375351	-8.01184	1.13E-15	5.51E-14
LY6H	10.3526952	4.189621029	1.262465	3.318603	0.000905	0.004468
MAFA	61.4143117	2.030204138	0.664746	3.054104	0.002257	0.009432
NAPRT	422.838839	-3.276888987	0.400065	-8.19089	2.59E-16	1.35E-14
GFUS	1659.91841	-2.234519561	0.352074	-6.34673	2.20E-10	5.41E-09
FAM83H	1769.4503	-2.35289348	0.333324	-7.05887	1.68E-12	5.53E-11
EPPK1	378.72447	-3.087749763	0.521394	-5.9221	3.18E-09	6.49E-08
PLEC	17488.8737	-3.464202686	0.326016	-10.6259	2.26E-26	2.60E-24
SCX	584.024589	-3.063815907	0.407357	-7.5212	5.43E-14	2.17E-12
GPT	66.652594	2.102085742	0.644042	3.263896	0.001099	0.005235
AF186192.2	38.216454	4.459859876	0.933272	4.778735	1.76E-06	2.04E-05
AL449043.2	23.7140121	3.498298054	0.962279	3.635429	0.000278	0.001681
DOCK8-AS1	22.4310188	3.777124332	1.003226	3.764977	0.000167	0.001099
DMRT1	11.6588646	4.283776559	1.259077	3.402316	0.000668	0.003478
DMRT2	12.7190282	4.360408778	1.25604	3.471552	0.000517	0.002815
CARM1P1	11.0643534	4.176955156	1.266227	3.298742	0.000971	0.004726
LINC01231	19.3230801	5.208831955	1.191884	4.370251	1.24E-05	0.000116
SLC1A1	355.066608	-3.379708568	0.410642	-8.2303	1.87E-16	9.89E-15
PDCD1LG2	89.783892	-2.163659425	0.674317	-3.20867	0.001334	0.006153
RIC1	732.665642	-2.084485362	0.353608	-5.8949	3.75E-09	7.54E-08
MLANA	37.6365511	4.268572757	0.911372	4.683676	2.82E-06	3.10E-05
AL162411.1	30.4024	3.118572487	0.88296	3.531953	0.000413	0.002335
PTPRD	961.109497	4.018422082	0.369559	10.87355	1.54E-27	1.93E-25
PTPRD-AS1	121.711533	6.076983342	0.72438	8.389224	4.89E-17	2.71E-15
RPS26P3	146.454324	2.626735084	0.655115	4.009576	6.08E-05	0.000462
RN7SL5P	995.11628	7.686474804	0.613485	12.5292	5.17E-36	1.05E-33
TYRP1	4357.75937	7.369586814	0.40437	18.22487	3.28E-74	4.11E-71
AL161449.2	19.5930345	3.534047063	1.099435	3.214422	0.001307	0.006047
AL137017.1	14.5289206	4.474807258	1.251749	3.574843	0.00035	0.002043
FREM1	31.5562155	4.901310293	1.040317	4.711361	2.46E-06	2.74E-05
BNC2-AS1	60.5803485	-2.17616515	0.677061	-3.21413	0.001308	0.006051
AL162725.2	10.9735652	4.22904493	1.261362	3.35276	0.0008	0.00404
CNTLN	372.162088	2.250376365	0.392208	5.737713	9.60E-09	1.79E-07
SH3GL2	330.665578	4.865628339	0.473883	10.26757	9.87E-25	1.02E-22
SAXO1	31.9760804	3.58617825	0.880628	4.072298	4.66E-05	0.000367
AL391834.1	46.2113058	5.030001427	0.88806	5.664033	1.48E-08	2.67E-07
SLC24A2	31.4537158	4.830277451	1.060638	4.554126	5.26E-06	5.39E-05
AL591222.1	10.5987009	4.173911508	1.264765	3.300148	0.000966	0.004718
AL353732.2	20.9081385	3.522226552	1.072918	3.282848	0.001028	0.004945
CDKN2A	5507.18242	-3.052033147	0.390926	-7.80718	5.85E-15	2.56E-13
CDKN2B	2776.89686	-3.084313203	0.315085	-9.78882	1.26E-22	1.15E-20
LINC01241	12.5638107	4.372264827	1.254408	3.485521	0.000491	0.00269
LINC00032	12.7190282	4.360408778	1.25604	3.471552	0.000517	0.002815
AL162388.2	24.2440939	3.53570142	0.95617	3.697776	0.000217	0.001371

ACO1	4358.44063	-2.834080295	0.320966	-8.82986	1.05E-18	6.83E-17
TAF1L	10.5606305	3.934530818	1.283673	3.065058	0.002176	0.009142
TMEM215	9.56489611	3.948402431	1.27948	3.085944	0.002029	0.008665
NOL6	1784.08316	-2.400424593	0.331411	-7.24305	4.39E-13	1.57E-11
ANKRD18B	100.457311	2.860359206	0.5699	5.019055	5.19E-07	6.90E-06
ANXA2P2	3203.14185	-3.437430315	0.399541	-8.60345	7.74E-18	4.65E-16
AL356489.2	26.7797663	4.593613072	1.023027	4.490217	7.12E-06	7.04E-05
AL356489.3	11.076065	4.340625154	1.251652	3.467917	0.000525	0.002849
UBAP1	1471.24959	-2.274983343	0.326364	-6.9707	3.15E-12	1.00E-10
CNTFR	27.0271091	2.995361076	0.906665	3.303713	0.000954	0.004683
AL162231.2	158.577734	2.183634117	0.507849	4.29977	1.71E-05	0.000153
PHF24	12.62824	4.428525514	1.249417	3.544475	0.000393	0.00225
DNAJB5	908.536368	-2.303051775	0.341132	-6.7512	1.47E-11	4.29E-10
AL353795.3	11.1287827	4.232433809	1.261651	3.354678	0.000795	0.004025
FAM214B	2634.51802	-3.675038244	0.341231	-10.7699	4.77E-27	5.74E-25
RUSC2	2255.54482	-2.838781754	0.319494	-8.88524	6.38E-19	4.29E-17
RMRP	342.629887	4.425417571	1.266049	3.495454	0.000473	0.002615
CA9	4073.13833	2.380267186	0.472248	5.040287	4.65E-07	6.26E-06
TPM2	62448.6421	-3.485328347	0.324695	-10.7342	7.04E-27	8.31E-25
TLN1	14905.0836	-3.288899497	0.347304	-9.46981	2.80E-21	2.31E-19
NPR2	432.067625	-2.087213789	0.388623	-5.37079	7.84E-08	1.23E-06
AL450267.2	17.8499817	5.032859884	1.206619	4.171043	3.03E-05	0.000251
PAX5	31.1522154	4.660161302	1.049425	4.44068	8.97E-06	8.66E-05
TMX2P1	186.102265	-2.26538411	0.465185	-4.86986	1.12E-06	1.36E-05
ALDH1B1	4471.25892	-3.482839755	0.321789	-10.8233	2.67E-27	3.29E-25
ANKRD18A	94.3833802	2.317336159	0.595834	3.889232	0.000101	0.000716
AL591543.1	9.53853722	4.032853085	1.27268	3.168788	0.001531	0.006875
VN2R3P	16.7517476	4.85108935	1.222407	3.968473	7.23E-05	0.000536
CNTNAP3	350.199103	2.950375856	0.400095	7.374195	1.65E-13	6.22E-12
AL353763.1	15.950972	4.060012386	1.177487	3.448033	0.000565	0.00303
AL591926.2	9.0728847	3.978599865	1.275392	3.119511	0.001812	0.007878
CNTNAP3C	57.5473796	4.274029599	0.89011	4.801687	1.57E-06	1.85E-05
FAM27C	33.4200039	3.468713599	0.875245	3.963136	7.40E-05	0.000547
LERFS	32.2617552	5.975612648	1.138905	5.246807	1.55E-07	2.30E-06
AL591438.2	14.2038382	4.771328413	1.221032	3.90762	9.32E-05	0.000668
CR786580.1	19.894574	3.838352511	1.141706	3.361943	0.000774	0.003938
AL669942.1	8.69802037	3.906713832	1.279695	3.052849	0.002267	0.009467
ANKRD20A3P	13.0294632	4.313295341	1.261231	3.41991	0.000626	0.003303
ANKRD20A1	11.2049236	4.378018982	1.248723	3.505998	0.000455	0.00254
PIP5K1B	263.840363	2.801166936	0.451673	6.201761	5.58E-10	1.29E-08
FAM189A2	186.956428	3.521724296	0.477125	7.381136	1.57E-13	5.94E-12
C9orf135	12.9461222	3.989267698	1.186613	3.361894	0.000774	0.003938
MAMDC2	1957.62551	-3.627411233	0.337507	-10.7477	6.08E-27	7.20E-25
TRPM3	2786.6711	4.672042253	0.364502	12.8176	1.31E-37	2.88E-35

RPL35AP21	58.0964859	2.959743551	0.747129	3.961492	7.45E-05	0.000549
GDA	27.549855	4.653012174	1.07114	4.343979	1.40E-05	0.000129
TMC1	39.5481877	2.806471672	0.871277	3.221103	0.001277	0.005929
RORB	107.287451	4.432714621	0.62214	7.124943	1.04E-12	3.55E-11
AL137018.1	13.9080505	4.552167927	1.242358	3.664136	0.000248	0.001529
TRPM6	89.894148	4.135570045	0.653365	6.329645	2.46E-10	5.99E-09
PCA3	28.1913712	4.571184229	1.044354	4.377045	1.20E-05	0.000112
AL353637.2	12.0981582	4.381361397	1.251818	3.499999	0.000465	0.002588
LNCARSR	10.3146248	4.067189627	1.272691	3.195739	0.001395	0.006369
NPAP1P6	11.1551416	4.079209622	1.274555	3.200497	0.001372	0.006296
SPATA31D5P	14.4381324	4.581379308	1.241445	3.69036	0.000224	0.001405
SPATA31D2P	13.9988387	4.466753428	1.25076	3.571231	0.000355	0.002065
AL162726.3	9.95147198	4.159442183	1.263635	3.291648	0.000996	0.004821
SPATA31D1	10.0041897	4.063829218	1.271846	3.195222	0.001397	0.006372
SPATA31B1P	12.00737	4.41656174	1.248133	3.538534	0.000402	0.002286
FRMD3	33.6858441	4.036161257	0.961367	4.198357	2.69E-05	0.000227
AL390838.1	12.317805	4.427294542	1.248333	3.546565	0.00039	0.002237
AL157886.1	11.0379945	4.269395787	1.258002	3.393791	0.000689	0.003567
GAS1RR	29.2043795	3.114211508	0.919641	3.386335	0.000708	0.003652
DAPK1	1384.61271	-2.660242995	0.336699	-7.90095	2.77E-15	1.27E-13
AL772337.1	9.82261335	4.112078605	1.267155	3.245126	0.001174	0.005514
SPATA31E1	17.3198999	5.00227436	1.208035	4.140834	3.46E-05	0.000282
SPATA31C1	11.8140821	4.278206312	1.260145	3.395012	0.000686	0.003556
SPATA31C2	12.7190282	4.360408778	1.25604	3.471552	0.000517	0.002815
SHC3	851.737954	-2.378163255	0.401357	-5.92531	3.12E-09	6.38E-08
AL353150.1	215.836116	-3.252832238	0.463139	-7.02344	2.16E-12	7.02E-11
UNQ6494	13.7264741	4.638510683	1.233011	3.761939	0.000169	0.001109
AL161629.1	16.0283779	4.618363928	1.243202	3.714893	0.000203	0.001297
DIRAS2	22.6328356	4.247844437	1.069456	3.971967	7.13E-05	0.00053
AL158071.3	11.7613643	4.425453629	1.246312	3.550839	0.000384	0.002208
LINC00484	16.3505244	4.904046298	1.215189	4.035623	5.45E-05	0.00042
AL158071.4	9.25446109	3.938692024	1.279145	3.07916	0.002076	0.008797
ROR2	553.525657	2.808641914	0.379296	7.404881	1.31E-13	5.04E-12
OGN	9.69375473	4.046019533	1.27217	3.180407	0.001471	0.006648
ASPN	10.4434834	4.166374392	1.26483	3.29402	0.000988	0.004787
IPPK	137.365556	-2.035512703	0.576006	-3.53384	0.00041	0.002321
FGD3	143.920925	-2.417333079	0.55367	-4.36602	1.27E-05	0.000118
NINJ1	1750.08171	-2.763612159	0.373503	-7.39916	1.37E-13	5.24E-12
PTMAP12	44.3047463	2.891035301	0.760225	3.802869	0.000143	0.000964
FBP2	28.0666181	5.293251275	1.057062	5.007515	5.51E-07	7.28E-06
AOPEP	1164.63582	-2.236451087	0.33369	-6.70219	2.05E-11	5.92E-10
LINC00092	12.7570987	4.511683948	1.241888	3.632924	0.00028	0.001694
AL449403.1	11.193212	4.276943502	1.257921	3.400009	0.000674	0.003502
AL449403.2	13.3516098	4.59562907	1.235878	3.718512	0.0002	0.001281

CTSV	389.880235	2.043004389	0.500084	4.085322	4.40E-05	0.000349
ANKRD18CP	13.8553328	4.695897067	1.227583	3.82532	0.000131	0.000891
CCDC180	36.8319009	2.751858714	0.847596	3.246663	0.001168	0.005495
TMOD1	15.0824255	4.887510435	1.21199	4.032634	5.52E-05	0.000423
TBC1D2	3510.9402	-3.016611283	0.312616	-9.64957	4.94E-22	4.33E-20
GABBR2	35.9329926	5.457784829	1.048713	5.20427	1.95E-07	2.83E-06
GALNT12	1523.34233	2.181873138	0.326268	6.68736	2.27E-11	6.50E-10
COL15A1	51.0074955	2.824668893	0.766105	3.68705	0.000227	0.001421
NAMA	15.5363665	4.79754536	1.223534	3.921056	8.82E-05	0.000637
TMEFF1	78.4447233	2.984466494	0.647922	4.60621	4.10E-06	4.32E-05
CAVIN4	142.955514	3.262606921	0.530876	6.145708	7.96E-10	1.79E-08
PLPPR1	21.0956745	3.31373069	1.045227	3.170346	0.001523	0.006847
BAAT	16.9247952	3.894710737	1.143234	3.406747	0.000657	0.003443
ALDOB	13.2227512	4.519365893	1.242964	3.635958	0.000277	0.001678
LINC01492	11.6325057	4.366275091	1.251409	3.489086	0.000485	0.002662
SMC2-AS1	21.3210732	3.614706582	1.045442	3.457585	0.000545	0.002948
TOPORSLP	15.187861	4.656333116	1.236755	3.764961	0.000167	0.001099
ABCA1	923.377722	-2.499277783	0.450532	-5.54739	2.90E-08	4.97E-07
TMEM38B	618.998283	3.178921791	0.365048	8.708221	3.09E-18	1.92E-16
LINC01505	47.7524538	4.166056211	0.892881	4.665859	3.07E-06	3.35E-05
AL807761.4	13.9080505	4.552167927	1.242358	3.664136	0.000248	0.001529
FRRS1L	98.7519571	2.080885756	0.611249	3.40432	0.000663	0.003466
EPB41L4B	1273.66359	4.710129059	0.359844	13.08935	3.79E-39	9.65E-37
PTPN3	838.493665	2.691496901	0.391839	6.868885	6.47E-12	1.97E-10
PALM2AKAP2	2493.11799	-2.29475021	0.319904	-7.17324	7.32E-13	2.53E-11
MUSK	31.2923797	5.926400632	1.142511	5.187173	2.14E-07	3.08E-06
ZNF483	201.95757	2.528812456	0.463655	5.454082	4.92E-08	8.07E-07
SUSD1	576.534011	-2.026332146	0.379699	-5.33668	9.47E-08	1.46E-06
SLC46A2	10.7275595	4.247693306	1.258756	3.374517	0.000739	0.003787
ZFP37	156.056954	2.346800078	0.5114	4.588971	4.45E-06	4.65E-05
RNF183	10.0686191	4.107514473	1.268446	3.238227	0.001203	0.005627
BSPRY	22.1939478	3.515226771	1.050042	3.347701	0.000815	0.004096
TNFSF8	12.0337289	4.332064733	1.25612	3.448766	0.000563	0.003025
TNC	5218.63011	-5.663230773	0.390234	-14.5124	1.01E-47	4.23E-45
PAPPA	1115.39147	-3.494607701	0.398337	-8.77299	1.74E-18	1.12E-16
AL133284.1	20.1052862	4.073326532	1.141265	3.569134	0.000358	0.002078
AL160272.1	30.9236339	4.816000559	1.05947	4.545668	5.48E-06	5.57E-05
GSN-AS1	24.1959827	3.28500287	0.965418	3.402675	0.000667	0.003477
STOM	712.426663	2.124996984	0.371059	5.726843	1.02E-08	1.90E-07
GGTA1	11.7232939	4.332937102	1.254862	3.45292	0.000555	0.002989
MORN5	24.6180269	5.719784819	1.148314	4.981027	6.32E-07	8.23E-06
LHX6	16.5057419	4.903137571	1.215891	4.032546	5.52E-05	0.000423
AC006450.3	92.0360469	2.711907913	0.617488	4.391838	1.12E-05	0.000106
ADGRD2	23.3821584	5.457253157	1.176682	4.637832	3.52E-06	3.77E-05

NR5A1	12.4203048	4.536071614	1.238139	3.66362	0.000249	0.001531
NR6A1	92.7062942	2.129599951	0.575405	3.701043	0.000215	0.001355
OLFML2A	269.164432	-2.975036727	0.581699	-5.11439	3.15E-07	4.40E-06
AL162391.1	11.8140821	4.278206312	1.260145	3.395012	0.000686	0.003556
LMX1B	26.1792563	4.607628267	1.068549	4.312041	1.62E-05	0.000146
ANGPTL2	1615.95215	2.527207129	0.326902	7.730777	1.07E-14	4.55E-13
NIBAN2	10347.3513	-3.669409062	0.360448	-10.1801	2.43E-24	2.47E-22
TTC16	20.5069152	3.541160797	1.056938	3.350396	0.000807	0.004068
ENG	431.504308	-3.656577052	0.417551	-8.7572	2.00E-18	1.28E-16
CERCAM	1928.14383	-2.252785817	0.341337	-6.59988	4.11E-11	1.14E-09
SPTAN1	11381.9582	-2.556259547	0.302853	-8.4406	3.16E-17	1.79E-15
LINC00963	3655.0633	2.220778537	0.312039	7.116982	1.10E-12	3.75E-11
NTMT1	621.590421	-2.188087995	0.379892	-5.75976	8.42E-09	1.59E-07
PTGES	1475.03244	-2.942857134	0.372535	-7.89954	2.80E-15	1.29E-13
NCS1	5831.44708	-3.284870979	0.308621	-10.6437	1.87E-26	2.15E-24
HMCN2	82.9251514	5.239426724	0.823315	6.363818	1.97E-10	4.87E-09
ABL1	3934.8999	-2.123978955	0.319973	-6.63799	3.18E-11	8.94E-10
FIBCD1	142.94429	-2.83617013	0.612899	-4.62747	3.70E-06	3.95E-05
LAMC3	41.346877	4.69697129	0.977166	4.806728	1.53E-06	1.81E-05
AIF1L	241.57307	-2.811643458	0.554209	-5.07326	3.91E-07	5.35E-06
BARHL1	12.6663105	4.53535658	1.23918	3.659967	0.000252	0.001551
ABO	23.0138185	3.767687324	1.01921	3.696674	0.000218	0.001376
ADAMTSL2	48.4364576	2.248534569	0.732486	3.069729	0.002143	0.009027
DBH-AS1	61.7362908	2.316541635	0.762193	3.039313	0.002371	0.009819
COL5A1	2706.66284	-2.431872874	0.371319	-6.54928	5.78E-11	1.56E-09
AL645768.1	14.8510671	4.75902198	1.224934	3.885127	0.000102	0.000725
FCN1	21.4170486	5.367116452	1.180708	4.545677	5.48E-06	5.57E-05
OLFM1	22.1818304	4.10544763	1.102066	3.725228	0.000195	0.001256
LINC01502	10.5079127	4.202889647	1.26189	3.330629	0.000866	0.004313
SOHLH1	14.5025617	4.638184454	1.236066	3.752377	0.000175	0.001146
KCNT1	64.6594189	4.610881379	0.859983	5.361595	8.25E-08	1.29E-06
NACC2	7661.22489	-3.026446578	0.336709	-8.98832	2.51E-19	1.75E-17
LHX3	10.9999241	4.102900639	1.272157	3.225153	0.001259	0.005856
CCDC187	41.451787	6.346776395	1.113829	5.698159	1.21E-08	2.22E-07
SNAPC4	1738.29494	-2.096786833	0.328259	-6.38761	1.69E-10	4.21E-09
EGFL7	1110.34036	-2.76946259	0.419307	-6.60486	3.98E-11	1.10E-09
LCN15	16.6873183	4.793916673	1.228127	3.903437	9.48E-05	0.000678
PTGDS	5117.17355	4.895318428	0.402688	12.1566	5.29E-34	9.64E-32
CLIC3	464.061555	-3.029429554	0.430713	-7.03351	2.01E-12	6.56E-11
MAN1B1	5158.9582	-2.004246786	0.341486	-5.86918	4.38E-09	8.68E-08
LRRC26	61.3408244	4.860113975	0.923524	5.262577	1.42E-07	2.13E-06
TPRN	17057.6644	3.045138571	0.352518	8.638251	5.71E-18	3.45E-16
TOR4A	2160.00122	-3.045259966	0.323085	-9.42558	4.28E-21	3.47E-19
ENTPD8	275.76071	4.572678644	0.538176	8.496619	1.95E-17	1.13E-15

RIC8A	3940.70697	-2.383172219	0.326551	-7.298	2.92E-13	1.07E-11
IFITM2	938.452012	-4.468416248	0.361498	-12.3608	4.26E-35	8.24E-33
B4GALNT4	4599.69875	-2.45544706	0.356481	-6.88801	5.66E-12	1.74E-10
PKP3	135.810056	-2.350903912	0.619026	-3.79775	0.000146	0.00098
RASSF7	2071.19725	-2.956008762	0.38435	-7.69093	1.46E-14	6.13E-13
MIR210HG	728.998439	-3.797969219	0.375003	-10.1278	4.16E-24	4.17E-22
MIR210	45.5754621	-4.828985169	0.892942	-5.40795	6.38E-08	1.02E-06
CDHR5	111.738906	4.448981202	0.605989	7.341682	2.11E-13	7.82E-12
SCT	35.0712014	2.979532153	0.881702	3.379297	0.000727	0.003732
DRD4	1765.9483	2.760333342	0.418376	6.59774	4.17E-11	1.15E-09
EPS8L2	4353.52599	-4.182275036	0.31241	-13.3872	7.19E-41	2.05E-38
AP006621.5	119.390055	-3.713418241	0.566907	-6.55031	5.74E-11	1.55E-09
SLC25A22	1007.0041	-2.011046017	0.363904	-5.52631	3.27E-08	5.53E-07
PNPLA2	2110.5744	-2.646042014	0.352819	-7.49972	6.40E-14	2.53E-12
CD151	16729.8675	-4.155267464	0.355984	-11.6726	1.76E-31	2.75E-29
MUC6	51.1318343	3.448681295	0.852724	4.044309	5.25E-05	0.000407
MUC2	59.820139	6.798067823	1.090791	6.232234	4.60E-10	1.07E-08
MUC5AC	46.234235	6.504796461	1.103544	5.894462	3.76E-09	7.55E-08
MUC5B	60.4479392	4.758369566	0.860984	5.526665	3.26E-08	5.52E-07
AC091196.1	10.5342716	4.124847874	1.268711	3.25121	0.001149	0.005437
TNNI2	24.9343335	5.357811426	1.193223	4.490202	7.12E-06	7.04E-05
LSP1	24.0208585	4.459181345	1.085559	4.107727	4.00E-05	0.000321
TNNT3	44.4200783	5.885372639	1.025807	5.737311	9.62E-09	1.80E-07
IGF2	16.3768833	4.818032405	1.224528	3.934605	8.33E-05	0.000606
C11orf21	16.2860951	4.867545546	1.218917	3.993337	6.51E-05	0.000489
TRPM5	15.6271546	4.749160485	1.228948	3.864411	0.000111	0.000778
KCNQ1OT1	424.064965	2.578651982	0.466629	5.52613	3.27E-08	5.53E-07
CDKN1C	1840.99063	2.074036839	0.372031	5.574899	2.48E-08	4.28E-07
PHLDA2	836.54062	-6.568913417	0.465776	-14.1032	3.63E-45	1.30E-42
MRGPRE	20.0610973	5.057206537	1.211654	4.173805	3.00E-05	0.000249
RHOG	883.150517	-2.620480085	0.418734	-6.2581	3.90E-10	9.18E-09
OR52I2	17.6303349	4.999970901	1.209478	4.13399	3.57E-05	0.00029
OR52I1	14.4381324	4.581379308	1.241445	3.69036	0.000224	0.001405
OR51D1	10.0949779	4.005107431	1.276891	3.136609	0.001709	0.007522
OR51E1	10.8183477	4.222792645	1.261322	3.34791	0.000814	0.004096
OR51E2	230.265638	4.10096674	0.542316	7.561944	3.97E-14	1.61E-12
OR51H2P	12.8742457	4.341881084	1.258229	3.450788	0.000559	0.003008
OR51L1	13.9988387	4.466753428	1.25076	3.571231	0.000355	0.002065
OR52A1	27.6872424	5.594999883	1.173544	4.767609	1.86E-06	2.14E-05
OR51M1	18.872075	4.900025585	1.224394	4.002001	6.28E-05	0.000476
OR51I2	13.9080505	4.552167927	1.242358	3.664136	0.000248	0.001529
UBQLN3	12.0337289	4.332064733	1.25612	3.448766	0.000563	0.003025
TRIM22	2172.92808	-3.045618928	0.342798	-8.8846	6.42E-19	4.31E-17
OR52N5	10.1330484	4.143717258	1.26561	3.274087	0.00106	0.005082

OR52E4	12.2797346	4.224539264	1.26635	3.335996	0.00085	0.004242
OR56A3	12.5901696	4.10169093	1.276939	3.212128	0.001318	0.006086
OR56A1	12.821528	4.544094362	1.238932	3.667753	0.000245	0.001511
DCHS1	151.339965	2.951181913	0.558673	5.282485	1.27E-07	1.92E-06
GVINP1	28.525349	4.066520715	1.057132	3.846747	0.00012	0.000829
OR2AG1	10.2882659	4.156236444	1.265118	3.285256	0.001019	0.004906
OR6A2	17.9026995	4.777245982	1.233689	3.872325	0.000108	0.000756
AC087280.2	13.0896282	3.910856954	1.202442	3.25243	0.001144	0.005427
SYT9	52.4010638	3.556502822	0.866886	4.102617	4.09E-05	0.000327
CYB5R2	1030.69122	2.948173019	0.400896	7.353965	1.92E-13	7.16E-12
TUB	378.933629	2.372594372	0.4278	5.546041	2.92E-08	5.00E-07
RIC3	39.3501013	4.201224965	0.867461	4.84313	1.28E-06	1.53E-05
RIC3-DT	19.8795209	5.173091033	1.198119	4.317676	1.58E-05	0.000143
SCUBE2	29.7270142	3.180441522	0.967888	3.28596	0.001016	0.0049
RPL23AP65	386.307788	6.310404365	0.537459	11.74119	7.84E-32	1.24E-29
AC011979.1	19.2401449	3.76897528	1.053079	3.579004	0.000345	0.002017
LYVE1	10.7539184	4.178531017	1.264949	3.303319	0.000955	0.004683
DKK3	8797.79886	-2.70719948	0.304491	-8.89091	6.06E-19	4.09E-17
MICAL2	2345.28588	-3.90566802	0.468825	-8.33075	8.03E-17	4.38E-15
PARVA	2956.36095	-2.168220422	0.314578	-6.89248	5.48E-12	1.68E-10
RASSF10	98.4798395	-3.360359109	0.705755	-4.76137	1.92E-06	2.20E-05
RRAS2	1586.90219	-2.029920918	0.341301	-5.9476	2.72E-09	5.63E-08
PDE3B	259.001149	2.48973304	0.430088	5.788899	7.08E-09	1.36E-07
CALCB	114.875756	6.942205595	0.823806	8.426988	3.55E-17	1.99E-15
INSC	12.1889464	4.326075881	1.257234	3.440947	0.00058	0.003092
LINC02682	12.0981582	4.381361397	1.251818	3.499999	0.000465	0.002588
SOX6	420.503349	4.498715341	0.410952	10.94705	6.86E-28	8.81E-26
PLEKHA7	589.174715	2.149872417	0.366815	5.86092	4.60E-09	9.09E-08
NCR3LG1	329.064333	3.783803278	0.46473	8.141945	3.89E-16	1.99E-14
AC124798.1	238.373881	3.696245585	0.564787	6.544492	5.97E-11	1.61E-09
ABCC8	49.7251611	6.533973329	1.107205	5.901321	3.61E-09	7.27E-08
USH1C	36.1734383	3.140081047	0.888588	3.533788	0.00041	0.002321
OTOG	54.2008824	4.673289575	0.933841	5.004376	5.60E-07	7.39E-06
KCNC1	45.1425253	4.792587277	0.926388	5.173415	2.30E-07	3.30E-06
SLC25A51P4	11.8140821	4.278206312	1.260145	3.395012	0.000686	0.003556
LDHA	14617.8206	-5.095266436	0.3417	-14.9115	2.77E-50	1.42E-47
IGSF22	68.1369896	2.511541944	0.645075	3.893408	9.88E-05	0.000704
PTPN5	17.3462588	4.923271681	1.21685	4.045917	5.21E-05	0.000404
E2F8	75.7351919	2.443627384	0.634436	3.851651	0.000117	0.000816
NAV2	1347.25012	-2.137958156	0.359352	-5.94948	2.69E-09	5.57E-08
SLC6A5	17.8119113	4.887945199	1.222233	3.999192	6.36E-05	0.00048
NELL1	19.2341461	3.81783789	1.093892	3.490141	0.000483	0.002655
ANO5	542.006599	3.176747796	0.381953	8.317107	9.01E-17	4.90E-15
SLC17A6	12.5638107	4.372264827	1.254408	3.485521	0.000491	0.00269

WIZP1	12.6503345	3.828967905	1.211971	3.159291	0.001582	0.007076
LUZP2	76.3086499	3.735566016	0.667279	5.598207	2.17E-08	3.80E-07
SLC5A12	19.9124041	3.48907181	1.064223	3.278516	0.001044	0.005014
FIBIN	97.4335165	-2.305515608	0.566161	-4.07219	4.66E-05	0.000367
BDNF	872.016198	-3.96817525	0.384727	-10.3143	6.07E-25	6.41E-23
MPPED2	61.9304661	4.33964117	0.743632	5.835739	5.36E-09	1.04E-07
MPPED2-AS1	15.6505777	4.965810036	1.20565	4.118782	3.81E-05	0.000308
AC131571.1	93.3779216	-2.333676131	0.622702	-3.74766	0.000178	0.001164
PAX6	1678.7785	-3.44106483	0.467447	-7.3614	1.82E-13	6.80E-12
WT1	31.18988	5.819928083	1.155883	5.035051	4.78E-07	6.41E-06
WT1-AS	14.3854146	4.744992649	1.224552	3.87488	0.000107	0.000749
CCDC73	19.4138683	5.181459929	1.195445	4.334337	1.46E-05	0.000133
CSTF3-DT	13.3516098	4.59562907	1.235878	3.718512	0.0002	0.001281
LMO2	17.5776171	5.103346337	1.197474	4.261758	2.03E-05	0.000177
ELF5	18.6904986	5.050527958	1.207739	4.181803	2.89E-05	0.000241
CD44	3667.30009	-2.953953551	0.336941	-8.76698	1.84E-18	1.18E-16
SLC1A2	50.8943488	4.97464213	0.911265	5.459048	4.79E-08	7.86E-07
PAMR1	298.177493	-3.69080749	0.552524	-6.6799	2.39E-11	6.82E-10
PRR5L	322.713933	-3.037383388	0.50668	-5.99468	2.04E-09	4.29E-08
AC080100.1	12.0717993	4.448565762	1.245315	3.57224	0.000354	0.002061
LRRC4C	34.1679261	2.806261652	0.901473	3.112973	0.001852	0.00802
LINC02741	10.6894891	4.123736642	1.269366	3.248658	0.00116	0.005468
LINC01499	15.691584	4.795258917	1.224373	3.916503	8.98E-05	0.000647
LINC02740	15.2259314	4.796445605	1.222436	3.923678	8.72E-05	0.000632
HNRNPKP3	9.87533112	3.936139891	1.281456	3.071616	0.002129	0.008981
ALX4	12.8859573	4.572223503	1.236371	3.698098	0.000217	0.00137
CD82	2667.8728	-2.511630155	0.31551	-7.96055	1.71E-15	8.15E-14
TP53I11	824.736516	-2.008678774	0.349375	-5.74935	8.96E-09	1.68E-07
SYT13	15.4342725	3.542842726	1.152612	3.07375	0.002114	0.008944
LINC02696	25.585151	3.973110367	0.985265	4.032528	5.52E-05	0.000423
AC103855.2	22.5035712	5.347605094	1.186874	4.505621	6.62E-06	6.59E-05
AC044839.1	21.8446307	5.250942569	1.195922	4.390708	1.13E-05	0.000106
LINC02716	21.8270476	5.528295615	1.162622	4.755023	1.98E-06	2.26E-05
SLC35C1	1148.0578	-2.324575806	0.348153	-6.67688	2.44E-11	6.95E-10
LARGE2	96.9499997	5.729506664	0.757746	7.561247	3.99E-14	1.61E-12
CREB3L1	1676.80384	-2.479126732	0.326344	-7.59667	3.04E-14	1.24E-12
DGKZ	1932.66362	-2.0676454	0.32713	-6.32057	2.61E-10	6.32E-09
MYBPC3	19.6335152	5.209846833	1.192937	4.367244	1.26E-05	0.000117
SPI1	14.7983493	4.842510208	1.215751	3.983144	6.80E-05	0.000508
C1QTNF4	82.1049912	2.084968916	0.590018	3.533737	0.00041	0.002321
PTPRJ	1875.18405	-2.226883815	0.328755	-6.77369	1.26E-11	3.70E-10
AC027369.1	10.2238366	4.116439207	1.268272	3.245707	0.001172	0.005504
AC118273.1	10.9735652	4.22904493	1.261362	3.35276	0.0008	0.00404
FOLH1	14.1657678	4.710734189	1.227281	3.83835	0.000124	0.000852

TYRL	16.889382	5.065553388	1.200002	4.221286	2.43E-05	0.000208
AC136759.1	12.6545989	4.269665777	1.263756	3.378552	0.000729	0.00374
AC109635.5	14.8129967	4.619407683	1.239067	3.728133	0.000193	0.001244
OR5AS1	23.5637348	5.376121837	1.186994	4.529192	5.92E-06	5.98E-05
OR8J2	9.25446109	3.938692024	1.279145	3.07916	0.002076	0.008797
OR5AK2	9.56489611	3.948402431	1.27948	3.085944	0.002029	0.008665
LRRC55	17.4634059	4.580908549	1.250573	3.663049	0.000249	0.001534
TNKS1BP1	5431.54345	-2.587088918	0.328727	-7.87002	3.55E-15	1.60E-13
P2RX3	17.0358238	4.93378004	1.214557	4.062206	4.86E-05	0.00038
RTN4RL2	80.7798904	4.229660314	0.655026	6.45724	1.07E-10	2.75E-09
SLC43A1	241.784553	3.739005068	0.463819	8.061349	7.55E-16	3.76E-14
SMTNL1	55.4008121	6.972390152	1.057411	6.593832	4.29E-11	1.18E-09
UBE2L6	919.04222	2.831622661	0.432885	6.541279	6.10E-11	1.64E-09
SERPING1	1531.0691	-2.746830426	0.354881	-7.74015	9.93E-15	4.25E-13
OR9Q2	13.0031044	4.473281904	1.246584	3.588433	0.000333	0.001957
GLYATL1	49.4381737	3.245334055	0.804362	4.03467	5.47E-05	0.000421
MPEG1	13.131963	4.556748427	1.238911	3.678026	0.000235	0.001464
OR5AN1	26.9755842	5.665463972	1.162735	4.872533	1.10E-06	1.35E-05
OR5A2	20.8488963	5.255990436	1.191943	4.409598	1.04E-05	9.83E-05
OR5A1	22.9692237	5.320791101	1.191531	4.465509	7.99E-06	7.81E-05
OR4D9	18.3598232	3.998726499	1.137069	3.516695	0.000437	0.002453
STX3	2016.3754	-2.147342636	0.328696	-6.53291	6.45E-11	1.72E-09
OOSP4B	14.2829148	4.592011422	1.239841	3.703711	0.000212	0.001343
MS4A2	19.9966679	4.981668924	1.219425	4.08526	4.40E-05	0.000349
MS4A6A	21.7538425	5.30038449	1.189889	4.454518	8.41E-06	8.17E-05
MS4A7	16.1572365	4.774355185	1.228275	3.887041	0.000101	0.000721
MS4A14	14.7485674	4.543772542	1.246109	3.646367	0.000266	0.001622
MS4A12	9.3188904	3.98167413	1.276003	3.120427	0.001806	0.007857
LINC00301	10.2882659	4.156236444	1.265118	3.285256	0.001019	0.004906
MS4A8	9.47410791	3.993729441	1.275601	3.130862	0.001743	0.007649
AP003721.1	133.969631	3.248859447	0.585833	5.545714	2.93E-08	5.00E-07
SLC15A3	55.9732544	2.925366513	0.708384	4.129634	3.63E-05	0.000295
CD6	24.1816691	5.65142082	1.155049	4.892796	9.94E-07	1.23E-05
CYB561A3	973.603844	-2.615393258	0.349071	-7.49244	6.76E-14	2.66E-12
AP002754.1	12.5755223	4.549478553	1.237415	3.676599	0.000236	0.001469
RAB3IL1	294.995416	3.39105695	0.42675	7.946237	1.92E-15	9.11E-14
BEST1	6426.97502	8.749054986	0.419789	20.84156	1.82E-96	5.60E-93
FTH1	20880.8416	-2.096940747	0.356313	-5.88511	3.98E-09	7.97E-08
ASRGL1	308.510619	2.701393337	0.46231	5.843255	5.12E-09	1.00E-07
GANAB	11616.728	-2.453251776	0.307679	-7.97341	1.54E-15	7.39E-14
CSKMT	238.556677	2.086875472	0.466111	4.477207	7.56E-06	7.43E-05
CHRM1	17.2554706	4.968540486	1.211556	4.100957	4.11E-05	0.000329
SLC22A6	16.9684586	5.108048526	1.194742	4.275441	1.91E-05	0.000168
SLC22A8	55.8430415	6.985124384	1.056122	6.613934	3.74E-11	1.04E-09

SLC22A25	14.8510671	4.75902198	1.224934	3.885127	0.000102	0.000725
PLAAT5	9.97783086	4.128966046	1.266293	3.260673	0.001111	0.005289
PLAAT3	2049.91726	2.457467023	0.432121	5.686992	1.29E-08	2.36E-07
SPINDOC	1155.6836	-2.718917166	0.362525	-7.49994	6.38E-14	2.53E-12
AP000721.2	17.4697713	3.426504936	1.096654	3.124509	0.001781	0.007777
AP006333.2	36.2255108	3.802613124	0.849428	4.476675	7.58E-06	7.44E-05
STIP1	4595.18703	-2.355192766	0.336934	-6.99008	2.75E-12	8.78E-11
PPP1R14B	7161.74185	-2.364113095	0.366966	-6.44232	1.18E-10	3.02E-09
PLCB3	1518.42405	-2.949435091	0.329729	-8.94503	3.72E-19	2.56E-17
SLC22A11	17.5395467	5.033079693	1.205405	4.175427	2.97E-05	0.000248
SLC22A12	12.7307398	4.561535224	1.236825	3.688101	0.000226	0.001416
NRXN2	74.8269175	5.772871341	0.812856	7.101962	1.23E-12	4.16E-11
EHD1	6633.7073	-3.455696143	0.333047	-10.376	3.19E-25	3.42E-23
MIR194-2HG	11.3484295	4.282003863	1.258062	3.403651	0.000665	0.003466
CAPN1	6446.16113	-2.299403796	0.353323	-6.50794	7.62E-11	2.01E-09
LTBP3	21442.9082	-2.448919975	0.355453	-6.88957	5.60E-12	1.72E-10
KCNK7	47.0851117	3.970629397	0.791839	5.014443	5.32E-07	7.05E-06
RELA	1808.09162	-2.189447772	0.356523	-6.14111	8.19E-10	1.84E-08
OVOL1	10.3146248	4.067189627	1.272691	3.195739	0.001395	0.006369
CFL1	26363.9197	-2.428825374	0.379723	-6.39631	1.59E-10	4.00E-09
EFEMP2	6035.78818	-2.961656864	0.364556	-8.12401	4.51E-16	2.30E-14
FOSL1	572.100764	-4.10335118	0.3885	-10.562	4.47E-26	5.00E-24
PACS1	2298.00109	-2.615747854	0.347888	-7.51894	5.52E-14	2.20E-12
RAB1B	2432.47871	-2.26188483	0.357211	-6.33207	2.42E-10	5.90E-09
CNIH2	176.911265	-2.682654352	0.476576	-5.62901	1.81E-08	3.22E-07
DPP3	2233.94138	-2.495936697	0.344917	-7.23634	4.61E-13	1.65E-11
ACTN3	15.4032434	4.18264886	1.177488	3.552179	0.000382	0.002199
RHOD	913.779937	-4.058982796	0.374154	-10.8484	2.03E-27	2.52E-25
SSH3	2002.20576	-2.664736212	0.323718	-8.23166	1.85E-16	9.79E-15
CLCF1	408.258093	-5.191830892	0.447214	-11.6093	3.70E-31	5.64E-29
ALDH3B2	11.9810111	4.463474905	1.243518	3.589394	0.000331	0.001954
LRP5	2080.36833	-2.069093873	0.321426	-6.43723	1.22E-10	3.11E-09
GAL	59.0812763	-2.656067498	0.83687	-3.17381	0.001505	0.006774
CPT1A	1681.10638	2.849249952	0.334519	8.517447	1.63E-17	9.53E-16
CCND1	21161.7861	-3.998750828	0.302867	-13.203	8.43E-40	2.23E-37
ANO1	18.7549279	5.089643378	1.203574	4.228774	2.35E-05	0.000202
CTTN	16634.6987	-2.781435732	0.350356	-7.93888	2.04E-15	9.60E-14
SHANK2	456.019786	2.799906084	0.409724	6.833645	8.28E-12	2.48E-10
SHANK2-AS1	12.62824	4.428525514	1.249417	3.544475	0.000393	0.00225
AP003783.1	12.2914461	4.487496012	1.242404	3.611945	0.000304	0.001818
ACTE1P	13.4687569	4.446583579	1.250803	3.554982	0.000378	0.002179
AP002387.1	65.7350014	-2.085939662	0.653104	-3.19388	0.001404	0.0064
UNC93B6	12.1319642	3.87567405	1.197817	3.235616	0.001214	0.005671
INPPL1	8335.65476	-2.651985399	0.306825	-8.64332	5.46E-18	3.31E-16

PDE2A	27.4510635	3.247466742	1.069285	3.037045	0.002389	0.00988
AP002761.3	45.4325366	-2.50250673	0.781133	-3.20369	0.001357	0.006239
ARHGEF17	2394.77041	-2.416274575	0.336337	-7.18408	6.77E-13	2.36E-11
AP003717.4	11.3484295	4.282003863	1.258062	3.403651	0.000665	0.003466
PPME1	1943.7966	-3.103169964	0.326747	-9.49716	2.16E-21	1.79E-19
P4HA3-AS1	13.0294632	4.313295341	1.261231	3.41991	0.000626	0.003303
KCNE3	58.5652245	3.553095371	0.716595	4.958306	7.11E-07	9.13E-06
CHRDL2	18.9101454	5.085147585	1.204649	4.221268	2.43E-05	0.000208
OR2AT4	17.2571414	4.039233679	1.193324	3.38486	0.000712	0.003668
SLCO2B1	29.6005658	3.617367975	1.033265	3.50091	0.000464	0.002582
SERPINH1	8030.7063	-2.797192078	0.309096	-9.04958	1.44E-19	1.02E-17
MOGAT2	11.6588646	4.283776559	1.259077	3.402316	0.000668	0.003478
WNT11	474.351075	4.635664618	1.247762	3.715185	0.000203	0.001296
LRRC32	600.273532	-4.31939059	0.462509	-9.33904	9.72E-21	7.59E-19
GUCY2EP	10.2238366	4.116439207	1.268272	3.245707	0.001172	0.005504
AP003119.2	66.8483081	2.482976099	0.67512	3.67783	0.000235	0.001464
B3GNT6	12.938675	4.416877878	1.251663	3.528807	0.000417	0.002357
CAPN5	1474.57332	-2.58775545	0.337793	-7.66076	1.85E-14	7.70E-13
MYO7A	1781.90924	3.614658955	0.333137	10.85036	1.99E-27	2.47E-25
GDPD4	13.0675337	4.51829105	1.242467	3.636549	0.000276	0.001677
NDUFC2	1373.79076	2.270667494	0.366803	6.190434	6.00E-10	1.38E-08
MIR4300HG	16.2658548	3.804728599	1.15643	3.290065	0.001002	0.00484
FAM181B	90.9769648	2.782685953	0.595945	4.66937	3.02E-06	3.30E-05
DLG2	130.523702	4.015797599	0.571819	7.022853	2.17E-12	7.04E-11
PRSS23	20339.7197	-3.033261295	0.321949	-9.42157	4.44E-21	3.59E-19
AP001528.2	93.8071072	2.174055804	0.583085	3.728539	0.000193	0.001243
RAB38	1030.74303	2.892468926	0.394779	7.32681	2.36E-13	8.71E-12
GRM5	20.6734385	4.197133543	1.115807	3.761523	0.000169	0.00111
TYR	8668.68561	9.756325302	0.397909	24.519	9.26E-133	1.57E-128
AP000720.1	25.8187607	5.767550603	1.146673	5.029815	4.91E-07	6.57E-06
FOLH1B	9.25446109	3.938692024	1.279145	3.07916	0.002076	0.008797
NAALAD2	262.56825	3.339757856	0.447255	7.467232	8.19E-14	3.21E-12
DEUP1	23.3246593	3.329685692	0.980025	3.397552	0.00068	0.003531
SESN3	5334.85037	2.224030816	0.31359	7.092167	1.32E-12	4.40E-11
PGR	112.737363	2.962571473	0.624667	4.742643	2.11E-06	2.39E-05
TRPC6	18.8635462	4.077298056	1.123232	3.629971	0.000283	0.001712
ANGPTL5	13.1072749	3.740916722	1.204663	3.105363	0.0019	0.008201
CEP126	171.476271	2.051504519	0.476336	4.306846	1.66E-05	0.000149
BIRC3	190.995119	2.190036192	0.473581	4.624415	3.76E-06	3.99E-05
MMP8	11.193212	4.276943502	1.257921	3.400009	0.000674	0.003502
MMP1	161.463684	-2.373777983	0.505736	-4.69371	2.68E-06	2.96E-05
MMP13	15.1234317	4.58060031	1.243877	3.682518	0.000231	0.001442
PDGFD	734.108291	2.016437718	0.360453	5.59418	2.22E-08	3.87E-07
LINC02552	47.4496162	6.516040378	1.104746	5.898222	3.67E-09	7.40E-08

GRIA4	30.9659688	5.258867744	1.087491	4.83578	1.33E-06	1.59E-05
HSPD1P13	10.6250598	4.049542664	1.275176	3.175673	0.001495	0.006735
ELMOD1	14.6228915	3.737090485	1.147504	3.256711	0.001127	0.005356
C11orf65	45.4187033	2.468286794	0.748116	3.299338	0.000969	0.004726
AP003049.2	19.1974043	4.213014305	1.090357	3.863884	0.000112	0.00078
C11orf87	15.4370495	3.831505064	1.136342	3.371788	0.000747	0.003818
LINC02715	14.7075611	4.846670339	1.214966	3.989142	6.63E-05	0.000497
LINC02732	67.8094053	7.270733665	1.037368	7.008831	2.40E-12	7.73E-11
COLCA1	21.0685432	5.28508789	1.189297	4.443876	8.84E-06	8.54E-05
POU2AF1	33.1859077	3.233024614	1.010857	3.198302	0.001382	0.006337
AP002008.3	9.62932542	4.002668209	1.275446	3.138251	0.0017	0.0075
AP002008.2	27.5093743	3.630662813	1.04117	3.4871	0.000488	0.002678
LAYN	2123.54246	-2.039479613	0.329949	-6.18119	6.36E-10	1.45E-08
CRYAB	668.410188	-2.399370766	0.391086	-6.13515	8.51E-10	1.90E-08
IL18	3488.00175	-2.157605003	0.332898	-6.48128	9.10E-11	2.37E-09
LINC02763	9.56489611	3.948402431	1.27948	3.085944	0.002029	0.008665
AP003100.2	14.3473442	4.642711077	1.235021	3.759217	0.00017	0.001119
AP000880.1	24.6104294	3.232609646	0.95511	3.384541	0.000713	0.003672
DRD2	19.8912324	5.302633597	1.182821	4.48304	7.36E-06	7.25E-05
HTR3B	17.4106881	4.965599823	1.212466	4.095453	4.21E-05	0.000335
ZBTB16	38.1951551	6.17638958	1.128569	5.47276	4.43E-08	7.35E-07
NNMT	529.962035	-2.365223771	0.424257	-5.57498	2.48E-08	4.28E-07
AP002518.1	12.8742457	4.341881084	1.258229	3.450788	0.000559	0.003008
C11orf71	121.980039	2.130404337	0.597671	3.564511	0.000365	0.002112
REXO2	1925.22252	-2.233947921	0.380246	-5.875	4.23E-09	8.40E-08
NXPE1	12.9123162	4.516215643	1.242058	3.636073	0.000277	0.001678
AP000997.3	19.7623738	5.260421909	1.187416	4.430141	9.42E-06	9.04E-05
APOA5	11.0379945	4.269395787	1.258002	3.393791	0.000689	0.003567
TAGLN	26840.0774	-8.085608807	0.378787	-21.346	4.24E-101	1.60E-97
DSCAML1	31.9376042	3.911445819	0.933843	4.188546	2.81E-05	0.000235
FXYD6	32.6166261	4.472778456	0.953642	4.690209	2.73E-06	3.01E-05
TMPRSS13	22.3634068	4.031594809	1.123093	3.589725	0.000331	0.001953
IL10RA	23.1337425	3.85134168	1.048319	3.673827	0.000239	0.001482
TMPRSS4	24.4865111	4.46490858	1.089675	4.097469	4.18E-05	0.000333
SCN4B	45.163983	3.820957127	0.90961	4.200656	2.66E-05	0.000225
SCN2B	187.357918	5.845910882	0.57978	10.08298	6.57E-24	6.47E-22
JAML	29.2985007	4.249800665	0.995036	4.271001	1.95E-05	0.000171
MPZL2	21.815336	5.522179928	1.163034	4.748083	2.05E-06	2.33E-05
CD3E	9.93976043	4.008663455	1.276073	3.141406	0.001681	0.007444
CD3G	17.437047	4.857396501	1.224147	3.967986	7.25E-05	0.000537
PHLDB1	5893.34737	-4.416261143	0.380918	-11.5937	4.43E-31	6.68E-29
TREH	17.2352302	3.903588178	1.145587	3.407499	0.000656	0.003436
AP004609.3	75.6583751	2.304715389	0.617134	3.734544	0.000188	0.001217
BCL9L	2419.21016	-3.08046369	0.388356	-7.93206	2.16E-15	1.01E-13

HYOU1	4295.48807	-2.196494534	0.316736	-6.93479	4.07E-12	1.27E-10
RNF26	858.534906	-2.081770708	0.351364	-5.92483	3.13E-09	6.39E-08
MFRP	1997.39799	8.072036651	0.413384	19.52675	6.50E-85	1.16E-81
THY1	1748.86292	-3.960331401	0.32929	-12.0269	2.57E-33	4.51E-31
AP003393.1	21.4029268	3.648332933	1.093194	3.337316	0.000846	0.004227
AP001360.2	10.7802773	4.027023489	1.277437	3.152423	0.001619	0.007214
TRIM29	31.0274708	3.243772823	0.909332	3.567203	0.000361	0.002092
GRIK4	42.778341	3.09787164	0.792647	3.908261	9.30E-05	0.000666
AP004147.1	23.4085173	5.385258369	1.18542	4.542912	5.55E-06	5.64E-05
AP000977.1	9.62932542	4.002668209	1.275446	3.138251	0.0017	0.0075
SORL1	457.533465	2.295486232	0.409308	5.608213	2.04E-08	3.61E-07
AP001977.1	15.1412618	3.694014192	1.166858	3.165778	0.001547	0.006941
UBASH3B	217.207643	-2.248950285	0.495795	-4.53605	5.73E-06	5.81E-05
CRTAM	12.2533757	4.381831723	1.252369	3.498834	0.000467	0.002588
GRAMD1B	692.474098	2.602539003	0.3619	7.191316	6.42E-13	2.25E-11
SCN3B	64.4993003	4.087295226	0.762883	5.357698	8.43E-08	1.32E-06
OR8G1	9.29253152	4.026062753	1.272371	3.16422	0.001555	0.006972
OR8D1	19.7243034	5.177123619	1.197093	4.324746	1.53E-05	0.000139
AP001804.1	10.405413	3.972768242	1.280392	3.102774	0.001917	0.00826
OR8B9P	14.5025617	4.638184454	1.236066	3.752377	0.000175	0.001146
OR8A1	15.7560133	4.834659718	1.220416	3.961487	7.45E-05	0.000549
CCDC15	70.8067163	2.51748673	0.641725	3.923002	8.75E-05	0.000633
PKNOX2	101.401648	3.18746521	0.582802	5.469206	4.52E-08	7.48E-07
DDX25	87.588981	4.388510275	0.68099	6.444305	1.16E-10	2.99E-09
AP001993.1	36.836268	6.245846535	1.115508	5.599103	2.15E-08	3.78E-07
DNAJB6P1	172.359973	-2.425208396	0.576301	-4.20823	2.57E-05	0.000219
LINC02725	12.7834575	4.424631592	1.250368	3.538662	0.000402	0.002286
KCNJ5	29.3158053	4.920263804	1.009665	4.873163	1.10E-06	1.34E-05
BARX2	13.7104982	3.919981071	1.207671	3.245901	0.001171	0.005504
TMEM45B	31.2549706	2.955836582	0.851083	3.473031	0.000515	0.002805
ADAMTS15	2812.96789	-5.501441892	0.36144	-15.2209	2.57E-52	1.48E-49
NTM	1457.11581	-4.961678063	0.397741	-12.4747	1.03E-35	2.02E-33
AP003025.2	22.1287068	5.324285971	1.188358	4.480372	7.45E-06	7.33E-05
OPCML	555.596602	6.056428332	0.430214	14.0777	5.21E-45	1.82E-42
AP003972.1	13.2491101	4.384912975	1.255665	3.492104	0.000479	0.002637
LINC02731	26.6895036	3.648047372	0.955868	3.816476	0.000135	0.000921
GLB1L3	32.2236848	5.902366674	1.148325	5.139979	2.75E-07	3.88E-06
B3GAT1-DT	41.157917	5.058041611	0.922686	5.481864	4.21E-08	7.00E-07
LINC02706	10.7539184	4.178531017	1.264949	3.303319	0.000955	0.004683
LINC00200	15.2786492	4.548576996	1.247368	3.646539	0.000266	0.001622
ADARB2	34.5373	6.015879688	1.140044	5.276882	1.31E-07	1.97E-06
LINC00700	14.0590036	4.032088638	1.191209	3.38487	0.000712	0.003668
AL441943.2	8.94402608	3.914238868	1.279942	3.058139	0.002227	0.009318
LINC02645	11.193212	4.276943502	1.257921	3.400009	0.000674	0.003502

AL713851.1	12.2533757	4.381831723	1.252369	3.498834	0.000467	0.002588
PFKP	8913.93327	-2.848211302	0.325913	-8.73918	2.35E-18	1.49E-16
LINC02668	12.5638107	4.372264827	1.254408	3.485521	0.000491	0.00269
LINC02669	18.4825633	5.170860545	1.193126	4.333876	1.47E-05	0.000134
AC025822.1	10.6894891	4.123736642	1.269366	3.248658	0.00116	0.005468
AC025822.2	9.72011361	3.945326647	1.280246	3.081694	0.002058	0.008746
AKR1C2	27.0112444	4.101530411	1.018107	4.028585	5.61E-05	0.00043
AKR1C8P	11.2840002	4.232661173	1.262212	3.353368	0.000798	0.004039
CALML3-AS1	23.1244412	5.307962375	1.193492	4.447423	8.69E-06	8.42E-05
IL2RA	17.6566938	4.903090935	1.22011	4.018563	5.86E-05	0.000447
PFKFB3	1530.52565	-3.082724646	0.360811	-8.54389	1.30E-17	7.64E-16
PRKCQ	77.1045634	4.917602994	0.769419	6.391316	1.64E-10	4.11E-09
PRKCQ-AS1	190.19906	2.806428727	0.471491	5.952244	2.64E-09	5.48E-08
LINC00707	26.0442791	5.67848686	1.158036	4.90355	9.41E-07	1.17E-05
AL392086.3	41.3934762	5.259887344	1.021475	5.149306	2.61E-07	3.71E-06
SFMBT2	88.507824	2.261711345	0.602473	3.754045	0.000174	0.00114
LINC02642	10.6250598	4.049542664	1.275176	3.175673	0.001495	0.006735
CELF2-DT	22.2956359	5.460755685	1.172394	4.657781	3.20E-06	3.46E-05
AL136452.1	30.4874046	-3.936494555	0.935693	-4.20704	2.59E-05	0.00022
SFTA1P	360.707512	-3.913513888	0.440201	-8.8903	6.09E-19	4.11E-17
LINC00710	32.8585348	2.84397987	0.921619	3.085852	0.00203	0.008665
CELF2-AS2	11.696935	4.398045908	1.248667	3.522193	0.000428	0.002409
CELF2-AS1	13.3516098	4.59562907	1.235878	3.718512	0.0002	0.001281
AC026887.1	10.8183477	4.222792645	1.261322	3.34791	0.000814	0.004096
ECHDC3	659.429343	2.529703378	0.406557	6.222253	4.90E-10	1.14E-08
CAMK1D	394.012676	-2.179202573	0.455593	-4.78323	1.73E-06	2.00E-05
AL731559.1	15.0326435	4.668138074	1.235019	3.779812	0.000157	0.001042
FAM107B	2034.85201	-2.220782659	0.335152	-6.6262	3.44E-11	9.64E-10
ITGA8	23.8012117	4.438965859	1.08862	4.077608	4.55E-05	0.000359
LINC02654	11.4655766	3.961938055	1.28417	3.085213	0.002034	0.008681
AL353576.1	17.0094649	4.999090528	1.207185	4.141114	3.46E-05	0.000282
RSU1	2261.62793	-3.391098635	0.336481	-10.0781	6.90E-24	6.78E-22
VIM	45641.4087	-2.183908742	0.407452	-5.35992	8.33E-08	1.30E-06
TMEM236	16.9450356	4.970285907	1.210168	4.107105	4.01E-05	0.000322
MRC1	25.4761269	5.587649678	1.167626	4.785479	1.71E-06	1.98E-05
SLC39A12	331.637808	8.478021105	0.77437	10.94828	6.77E-28	8.73E-26
SLC39A12-AS1	12.4085932	4.379167019	1.253201	3.494385	0.000475	0.002618
CACNB2	59.4434952	2.292379495	0.670523	3.418793	0.000629	0.003315
AL390783.1	18.6904986	5.050527958	1.207739	4.181803	2.89E-05	0.000241
MALRD1	29.7305697	4.241697414	1.027628	4.127658	3.66E-05	0.000297
AL157895.1	13.752833	4.558390099	1.241168	3.672661	0.00024	0.001487
PLXDC2	1437.98515	-2.033223572	0.334475	-6.07886	1.21E-09	2.64E-08
SPAG6	20.4476731	5.290551296	1.186383	4.459396	8.22E-06	8.01E-05
ARMC3	53.6471074	3.829788429	0.78245	4.894609	9.85E-07	1.22E-05

AL139815.1	11.193212	4.276943502	1.257921	3.400009	0.000674	0.003502
ENKUR	31.5449819	2.925049271	0.85935	3.403791	0.000665	0.003466
LINC01516	9.25446109	3.938692024	1.279145	3.07916	0.002076	0.008797
GPR158	27.7060038	3.232280394	1.032977	3.129094	0.001753	0.007688
LINC00836	30.0506397	5.933491038	1.13775	5.215111	1.84E-07	2.68E-06
GAD2	27.9449597	5.724734193	1.158306	4.942334	7.72E-07	9.82E-06
PTCHD3	13.9197621	4.71952845	1.225366	3.851525	0.000117	0.000816
MKX	75.02914	5.731711024	0.790806	7.247936	4.23E-13	1.52E-11
MPP7	121.931061	2.061697673	0.541912	3.804486	0.000142	0.00096
BAMBI	2814.09006	-3.056493336	0.326101	-9.37285	7.06E-21	5.63E-19
LINC02664	9.87533112	3.936139891	1.281456	3.071616	0.002129	0.008981
RPS4XP11	154.104459	2.669448724	0.566659	4.710853	2.47E-06	2.75E-05
CCDC7	51.5357232	2.990589742	0.775798	3.854856	0.000116	0.000806
ITGB1	23694.897	-4.014023422	0.301637	-13.3075	2.09E-40	5.82E-38
NRP1	943.000308	-3.11542198	0.368267	-8.45967	2.68E-17	1.53E-15
AL353600.2	10.8183477	4.222792645	1.261322	3.34791	0.000814	0.004096
LINC00838	10.2501954	3.994010754	1.278276	3.124529	0.001781	0.007777
PCAT5	11.7496528	4.203681987	1.266356	3.31951	0.000902	0.004457
ANKRD30A	16.9713944	4.89148208	1.218905	4.013014	5.99E-05	0.000456
BX322639.1	134.171057	3.434553053	0.529568	6.485569	8.84E-11	2.31E-09
CAP1P2	63.466653	-2.800817535	0.670553	-4.17688	2.96E-05	0.000246
LINC00840	22.890147	5.506264213	1.168869	4.71076	2.47E-06	2.75E-05
LINC00841	13.8553328	4.695897067	1.227583	3.82532	0.000131	0.000891
AL137026.1	8.67166149	3.922044738	1.278537	3.067603	0.002158	0.009082
CXCL12	1121.72195	4.874363104	0.377831	12.90089	4.45E-38	1.02E-35
TMEM72	17.5131878	5.082003491	1.199693	4.236088	2.27E-05	0.000197
OR6D1P	12.0981582	4.381361397	1.251818	3.499999	0.000465	0.002588
ANTXRLP1	11.5036471	4.283732053	1.258499	3.403843	0.000664	0.003466
ANXA8L1	123.766181	-4.331807687	0.598496	-7.23783	4.56E-13	1.63E-11
LINC00842	52.926649	-2.338703691	0.718426	-3.25531	0.001133	0.005379
PTPN20	27.2985032	3.743967271	0.983042	3.808554	0.00014	0.000947
ANXA8	299.433484	-4.814507392	0.571111	-8.43007	3.45E-17	1.94E-15
FO681492.1	88.7508376	-2.931634676	0.677211	-4.32898	1.50E-05	0.000136
AC245041.2	171.361025	-4.175517414	0.660551	-6.32127	2.59E-10	6.29E-09
FRMPD2	26.9228664	5.755957005	1.150977	5.000931	5.71E-07	7.51E-06
WDFY4	57.5214265	4.072140509	0.822387	4.95161	7.36E-07	9.41E-06
FAM170B-AS1	13.8553328	4.695897067	1.227583	3.82532	0.000131	0.000891
TMEM273	20.0845203	5.352849082	1.177456	4.546113	5.46E-06	5.57E-05
C10orf71	13.9461209	4.675279624	1.230098	3.800737	0.000144	0.000971
CHAT	19.2586508	5.181979812	1.194801	4.337106	1.44E-05	0.000132
SLC18A3	13.5712566	4.633033107	1.232949	3.757685	0.000171	0.001125
C10orf53	16.9977533	4.750623989	1.23351	3.851305	0.000117	0.000817
OGDHL	1209.22007	5.885332518	0.382991	15.36676	2.73E-53	1.60E-50
PRKG1	179.922137	2.234300685	0.496678	4.498492	6.84E-06	6.80E-05

PRKG1-AS1	17.5854065	3.547582809	1.120618	3.165739	0.001547	0.006941
DKK1	186.344538	-2.397661483	0.494061	-4.85297	1.22E-06	1.46E-05
MBL2	11.0643534	4.176955156	1.266227	3.298742	0.000971	0.004726
PCDH15	57.8436208	3.263188202	0.84954	3.841124	0.000122	0.000843
LINC00844	27.4148778	5.709938331	1.158512	4.928685	8.28E-07	1.05E-05
PHYHIPL	91.0664209	5.838857878	0.812831	7.183364	6.80E-13	2.37E-11
AC026391.1	13.1583219	4.468559565	1.247619	3.58167	0.000341	0.002
SLC16A9	64.0815475	2.000192095	0.651727	3.069065	0.002147	0.009043
ANK3	1667.81649	5.877119588	0.348035	16.88658	5.65E-64	4.46E-61
CDK1	892.084874	2.671184688	0.408231	6.543316	6.02E-11	1.62E-09
TMEM26	65.2944988	5.136843548	0.80655	6.368908	1.90E-10	4.72E-09
CABCOCO1	142.92373	2.346821022	0.555661	4.223476	2.41E-05	0.000206
ARID5B	1961.93921	-2.406465396	0.320911	-7.49885	6.44E-14	2.55E-12
LINC02671	9.60296653	4.065518288	1.270238	3.200595	0.001371	0.006295
LINC01515	101.816309	2.642465689	0.557945	4.736068	2.18E-06	2.46E-05
LRRTM3	14.0369091	4.644853865	1.233596	3.765297	0.000166	0.001098
DNAJC12	83.6281074	3.616654249	0.624924	5.787351	7.15E-09	1.37E-07
TET1	75.5910491	2.883601439	0.62802	4.591579	4.40E-06	4.60E-05
STOX1	72.893498	3.873885643	0.681955	5.680558	1.34E-08	2.44E-07
HKDC1	643.746632	-2.398897338	0.359112	-6.68008	2.39E-11	6.81E-10
NPFFR1	4974.79565	12.17766774	0.685988	17.75201	1.66E-70	1.61E-67
ADAMTS14	424.401008	-3.81778345	0.478785	-7.9739	1.54E-15	7.37E-14
SGPL1	1933.31116	-2.80379578	0.321518	-8.72051	2.77E-18	1.74E-16
CDH23	122.083535	3.183280782	0.612812	5.194547	2.05E-07	2.97E-06
VSIR	1426.38416	-2.085333733	0.417447	-4.99544	5.87E-07	7.70E-06
AC073370.1	56.33838	3.254732515	0.769677	4.228701	2.35E-05	0.000202
CHST3	1334.58373	-2.374994679	0.367799	-6.45731	1.07E-10	2.75E-09
AC022400.6	181.671928	-2.756077495	0.507058	-5.43543	5.47E-08	8.90E-07
PLAU	1485.97671	-4.718102928	0.341085	-13.8326	1.62E-43	5.23E-41
VCL	10129.6976	-2.282665304	0.316385	-7.21483	5.40E-13	1.91E-11
AP3M1	756.52641	-2.02940256	0.347971	-5.8321	5.47E-09	1.07E-07
AC018511.6	10.4434834	4.166374392	1.26483	3.29402	0.000988	0.004787
COMTD1	2032.19299	2.130034998	0.364119	5.849826	4.92E-09	9.66E-08
ZNF503-AS1	25.7968337	3.029255231	0.995898	3.041734	0.002352	0.00976
KCNMA1	1780.75475	-2.545666257	0.341855	-7.44663	9.58E-14	3.72E-12
KCNMA1-AS1	14.0369091	4.644853865	1.233596	3.765297	0.000166	0.001098
KCNMA1-AS3	15.9639485	4.451184625	1.257878	3.538646	0.000402	0.002286
LINC00595	30.4099424	2.89082436	0.94151	3.070412	0.002138	0.009013
ZCCHC24	517.359996	-2.03081377	0.372714	-5.44872	5.07E-08	8.30E-07
SFTPA2	14.9682142	4.603296853	1.241181	3.708802	0.000208	0.001321
NUTM2E	18.2280288	4.127020736	1.100222	3.75108	0.000176	0.001151
SFTPD-AS1	10.7539184	4.178531017	1.264949	3.303319	0.000955	0.004683
ANXA11	8628.20181	-2.915426579	0.320115	-9.10744	8.44E-20	6.19E-18
LINC00857	140.600463	-3.288462627	0.55902	-5.88255	4.04E-09	8.08E-08

EIF5AP4	89.8228962	-2.808780464	0.602957	-4.65834	3.19E-06	3.46E-05
MAT1A	14.8129967	4.619407683	1.239067	3.728133	0.000193	0.001244
SH2D4B	16.4149537	4.934555343	1.212074	4.071168	4.68E-05	0.000368
AL096706.1	51.0384684	4.138568743	0.847537	4.883056	1.04E-06	1.28E-05
NRG3	263.886516	5.31835576	0.516046	10.30598	6.62E-25	6.95E-23
CDHR1	250.433734	5.978458859	0.539308	11.08543	1.48E-28	1.99E-26
RGR	275.217591	9.168249278	0.939775	9.755793	1.74E-22	1.57E-20
LINC00858	12.1625875	4.422982886	1.248134	3.543676	0.000395	0.002253
RPS3AP5	128.521177	2.77645343	0.543451	5.108929	3.24E-07	4.52E-06
AL731532.1	15.7823722	4.739937488	1.230461	3.852164	0.000117	0.000815
GRID1-AS1	10.3146248	4.067189627	1.272691	3.195739	0.001395	0.006369
GRID1	19.7746109	3.487853134	1.119696	3.114999	0.001839	0.007983
OPN4	12.4466636	4.49720804	1.242071	3.620732	0.000294	0.001764
SNCG	102.836838	-4.300404358	0.683006	-6.29629	3.05E-10	7.29E-09
PAPSS2	735.633546	-2.726956859	0.35721	-7.63404	2.28E-14	9.40E-13
LIPJ	12.4730225	4.428932897	1.248782	3.546601	0.00039	0.002237
LIPN	10.9091359	4.179411771	1.265451	3.302706	0.000958	0.004688
ACTA2	6127.67495	-7.454558454	0.340134	-21.9165	1.81E-106	8.75E-103
AL353751.1	11.5153586	4.416266	1.246262	3.543608	0.000395	0.002253
LINC00865	28.3746905	2.772210762	0.913077	3.036119	0.002396	0.009906
LINC01374	9.60296653	4.065518288	1.270238	3.200595	0.001371	0.006295
LINC01375	8.94402608	3.914238868	1.279942	3.058139	0.002227	0.009318
AL391704.1	19.3474346	3.153757415	1.01053	3.120895	0.001803	0.007857
ANKRD1	1866.12247	-4.477599685	0.46543	-9.62035	6.56E-22	5.69E-20
PPP1R3C	763.097101	-3.458693171	0.408287	-8.47123	2.43E-17	1.39E-15
SRP9P1	34.0992568	2.750359591	0.828223	3.320794	0.000898	0.004445
CPEB3	85.8566729	2.600406836	0.60737	4.281423	1.86E-05	0.000164
CYP26C1	50.0047027	4.310157815	0.791444	5.445942	5.15E-08	8.42E-07
AL358613.2	19.2440035	5.300108967	1.180679	4.489033	7.15E-06	7.08E-05
CYP26A1	982.96406	10.82763109	0.877775	12.33531	5.85E-35	1.12E-32
MYOF	4965.95335	-3.394494515	0.350048	-9.69722	3.10E-22	2.77E-20
FFAR4	12.4085932	4.379167019	1.253201	3.494385	0.000475	0.002618
LGI1	49.3925204	4.253156349	0.933824	4.55456	5.25E-06	5.38E-05
AL157396.1	19.1415038	5.245648418	1.186801	4.419991	9.87E-06	9.42E-05
PLCE1-AS2	12.4085932	4.379167019	1.253201	3.494385	0.000475	0.002618
CYP2C19	17.3462588	4.923271681	1.21685	4.045917	5.21E-05	0.000404
CYP2C9	11.5036471	4.283732053	1.258499	3.403843	0.000664	0.003466
ENTPD1-AS1	59.7247943	2.961776445	0.745544	3.972639	7.11E-05	0.000528
CC2D2B	26.4642638	3.242433109	0.996353	3.2543	0.001137	0.005396
BLNK	15.0707139	4.794131131	1.222066	3.922974	8.75E-05	0.000633
SLIT1	44.4221243	3.319545623	0.872073	3.806501	0.000141	0.000954
ARHGAP19-SLIT1	10.4434834	4.166374392	1.26483	3.29402	0.000988	0.004787
FRAT1	51.6680102	-2.751389913	0.79304	-3.46942	0.000522	0.002835
PGAM1	9014.90735	-2.374881471	0.318619	-7.45366	9.08E-14	3.54E-12

UBTD1	3088.25225	-2.044911926	0.352551	-5.80033	6.62E-09	1.27E-07
MARVELD1	2455.27648	-2.811590287	0.322984	-8.70505	3.17E-18	1.97E-16
SFRP5	9936.68716	8.73838243	0.360198	24.25994	5.19E-130	5.87E-126
GOLGA7B	271.326367	5.031037104	0.521161	9.653521	4.75E-22	4.18E-20
CRTAC1	702.447411	6.621344542	0.45684	14.49379	1.33E-47	5.48E-45
LOXL4	649.181644	-3.664917416	0.38699	-9.47032	2.79E-21	2.30E-19
HPSE2	21.1066136	5.36488684	1.179834	4.547154	5.44E-06	5.55E-05
ABCC2	33.8937794	4.002963471	0.982132	4.075791	4.59E-05	0.000362
PAX2	22.3060188	4.718792125	1.136641	4.151523	3.30E-05	0.000271
LBX1	9.93976043	4.008663455	1.276073	3.141406	0.001681	0.007444
LDB1	5160.79399	-2.325120944	0.318878	-7.29158	3.06E-13	1.12E-11
TRIM8	2888.69416	-2.018560345	0.320207	-6.30392	2.90E-10	6.99E-09
INA	74.844761	-2.459835219	0.741305	-3.31825	0.000906	0.004473
COL17A1	30.9565172	3.803652083	0.969944	3.921516	8.80E-05	0.000636
SFR1	211.150453	2.384820768	0.495847	4.809589	1.51E-06	1.79E-05
CFAP43	59.3429107	4.35048748	0.776864	5.600063	2.14E-08	3.76E-07
CFAP58-DT	17.5807999	4.064811326	1.109005	3.665277	0.000247	0.001524
SORCS3	37.2218905	3.137777613	0.920331	3.409401	0.000651	0.003421
SORCS1	49.7978577	3.498350782	0.80592	4.340817	1.42E-05	0.00013
LINC01435	37.9900445	3.687183948	0.974986	3.781782	0.000156	0.001036
ADD3-AS1	31.2077101	4.854924982	1.050776	4.620325	3.83E-06	4.06E-05
ADD3	2276.33077	2.370987308	0.365882	6.480193	9.16E-11	2.38E-09
RBM20	42.6057407	2.819945118	0.855576	3.295962	0.000981	0.004768
TECTB	16.0137305	4.946010046	1.209194	4.090337	4.31E-05	0.000342
GUCY2GP	15.6271546	4.749160485	1.228948	3.864411	0.000111	0.000778
AL157786.1	16.9218594	4.002029319	1.1158	3.586689	0.000335	0.001968
HABP2	11.7877232	4.373743725	1.25132	3.495305	0.000474	0.002615
NRAP	29.444417	5.79472157	1.154005	5.021401	5.13E-07	6.83E-06
PLEKHS1	18.2487947	3.418998392	1.11313	3.071518	0.00213	0.008983
ADRB1	222.883451	4.890522435	0.500744	9.766505	1.57E-22	1.42E-20
TDRD1	17.5395467	5.033079693	1.205405	4.175427	2.97E-05	0.000248
ABLIM1	1312.72787	-2.695352345	0.349551	-7.7109	1.25E-14	5.29E-13
ATRNL1	690.905044	2.513800575	0.359076	7.000749	2.55E-12	8.16E-11
GFRA1	966.536734	-3.242115286	0.382447	-8.47729	2.31E-17	1.32E-15
PNLIPRP3	13.0031044	4.473281904	1.246584	3.588433	0.000333	0.001957
PNLIPRP1	16.4210723	3.806333826	1.158376	3.285923	0.001016	0.0049
VAX1	21.2501196	5.190539354	1.200823	4.322484	1.54E-05	0.00014
MIR3663HG	10.4698423	4.061968818	1.273658	3.189215	0.001427	0.006475
EMX2OS	23.2508895	3.812052851	1.066137	3.575576	0.000349	0.002039
CASC2	73.5607375	3.034434456	0.667658	4.544893	5.50E-06	5.59E-05
AL354863.1	13.8172623	4.603767001	1.236903	3.722012	0.000198	0.001268
PRLHR	12.4466636	4.49720804	1.242071	3.620732	0.000294	0.001764
AL157388.1	14.9799257	4.817339829	1.2192	3.951232	7.78E-05	0.00057
NANOS1	10807.7864	6.408963647	0.357066	17.94895	4.89E-72	5.18E-69

BAG3	1699.70483	-2.985366075	0.350857	-8.50878	1.76E-17	1.03E-15
RPL21P16	609.97994	2.276141109	0.438094	5.195553	2.04E-07	2.95E-06
WDR11-AS1	24.7479757	3.360092764	1.000364	3.358871	0.000783	0.003974
FGFR2	594.490873	4.404664693	0.394862	11.15495	6.77E-29	9.18E-27
ATE1-AS1	27.5153731	3.730687689	0.93234	4.001426	6.30E-05	0.000476
DMBT1	29.311294	5.190342902	1.090822	4.758193	1.95E-06	2.23E-05
DMBT1L1	40.9099936	6.23242586	1.127848	5.525945	3.28E-08	5.53E-07
LINC02641	16.7211243	4.365243341	1.155494	3.777816	0.000158	0.00105
GPR26	24.7000393	5.596356402	1.163914	4.808221	1.52E-06	1.80E-05
CPXM2	1278.15535	2.321128502	0.349695	6.637585	3.19E-11	8.96E-10
FAM53B	2250.90922	2.611830761	0.350086	7.460548	8.62E-14	3.37E-12
MMP21	23.0251242	4.42856179	1.081517	4.094769	4.23E-05	0.000336
C10orf90	17.9468885	3.842478816	1.170645	3.282359	0.001029	0.00495
AL359094.1	12.0717993	4.448565762	1.245315	3.57224	0.000354	0.002061
LINC01163	9.87533112	3.936139891	1.281456	3.071616	0.002129	0.008981
EBF3	28.2407473	5.861045816	1.141541	5.134327	2.83E-07	3.98E-06
TCERG1L	27.1308017	5.660938184	1.163792	4.864219	1.15E-06	1.39E-05
DPYSL4	722.800762	-2.077105036	0.383876	-5.41087	6.27E-08	1.01E-06
NKX6-2	11.2195709	4.169021091	1.267451	3.289296	0.001004	0.00485
LINC01168	12.1625875	4.422982886	1.248134	3.543676	0.000395	0.002253
ADGRA1	26.5362906	5.617956939	1.167274	4.812888	1.49E-06	1.76E-05
ADGRA1-AS1	18.2248461	5.063132891	1.20461	4.20313	2.63E-05	0.000223
KNDC1	172.5118	2.059818654	0.474113	4.34457	1.40E-05	0.000128
ADAM8	1962.07565	2.605950348	0.349623	7.453605	9.08E-14	3.54E-12
IQSEC3	381.448045	6.838967447	0.54162	12.62687	1.50E-36	3.14E-34
AC026369.1	130.303751	7.121508916	0.815976	8.727598	2.60E-18	1.64E-16
AC007406.3	27.025772	4.60130911	1.023414	4.496037	6.92E-06	6.86E-05
SLC6A12	93.8750396	3.931712638	0.691156	5.688604	1.28E-08	2.34E-07
SLC6A12-AS1	13.5448977	4.676080843	1.228401	3.80664	0.000141	0.000954
SLC6A13	41.0366889	4.40469369	0.893723	4.928476	8.29E-07	1.05E-05
WNT5B	580.523325	-3.019480053	0.379115	-7.96455	1.66E-15	7.92E-14
CACNA2D4	156.621073	4.214093541	0.528025	7.980856	1.45E-15	7.01E-14
CACNA1C	99.7205014	3.177464684	0.649741	4.890353	1.01E-06	1.24E-05
AC125807.1	37.7531726	5.318331745	0.971125	5.476462	4.34E-08	7.20E-07
PRMT8	23.0733942	4.616049854	1.13478	4.067792	4.75E-05	0.000373
THCAT155	16.2480247	4.685379322	1.237493	3.786186	0.000153	0.001021
CCND2	4074.03468	-2.121223428	0.311345	-6.81309	9.55E-12	2.85E-10
TIGAR	302.545153	-2.305468862	0.417032	-5.52828	3.23E-08	5.48E-07
FGF23	12.3441639	4.315094906	1.258772	3.428019	0.000608	0.003216
RAD51AP1	224.664951	2.060332002	0.456823	4.510134	6.48E-06	6.47E-05
AC005906.2	21.0041138	5.251088729	1.193062	4.401353	1.08E-05	0.000102
KCNA1	21.1974018	5.342100178	1.18294	4.515953	6.30E-06	6.32E-05
KCNA5	30.4756918	4.819021823	0.998489	4.826315	1.39E-06	1.65E-05
LINC02443	15.4075078	4.704549513	1.232708	3.816434	0.000135	0.000921

AC006206.1	12.2270168	4.457657865	1.245054	3.580294	0.000343	0.002009
NTF3	126.690317	-3.413624954	0.598943	-5.69941	1.20E-08	2.20E-07
PLEKHG6	446.954559	2.896401691	0.381629	7.58958	3.21E-14	1.31E-12
TNFRSF1A	4418.73604	-2.412514673	0.326729	-7.38384	1.54E-13	5.83E-12
LTBR	1372.23781	-2.332123494	0.345942	-6.74138	1.57E-11	4.56E-10
VAMP1	645.013272	-3.510849464	0.366282	-9.5851	9.24E-22	7.88E-20
GAPDH	57619.6377	-3.363128494	0.322659	-10.4232	1.94E-25	2.10E-23
GNB3	595.483492	2.67653295	0.424429	6.306201	2.86E-10	6.90E-09
C12orf57	2058.80813	3.105824324	0.321369	9.664353	4.27E-22	3.78E-20
C1R	4449.08676	2.401893361	0.4298	5.588401	2.29E-08	3.98E-07
ACSM4	10.3146248	4.067189627	1.272691	3.195739	0.001395	0.006369
CD163L1	22.8140062	5.331304073	1.189813	4.48079	7.44E-06	7.32E-05
CD163	29.0959115	5.713116458	1.163229	4.911428	9.04E-07	1.13E-05
AC006511.3	19.6954145	4.028490675	1.093104	3.685368	0.000228	0.001429
AC092111.1	22.7319937	5.597166802	1.157105	4.837215	1.32E-06	1.58E-05
CLEC4E	12.7570987	4.511683948	1.241888	3.632924	0.00028	0.001694
A2ML1	39.3535541	5.693279143	1.042199	5.462754	4.69E-08	7.72E-07
M6PR	1191.22297	-2.105291322	0.331064	-6.35917	2.03E-10	5.01E-09
A2M	34.315243	4.417171422	1.002432	4.406456	1.05E-05	9.97E-05
PZP	23.2796587	5.292230692	1.195754	4.425852	9.61E-06	9.19E-05
A2MP1	17.126612	4.883287581	1.220343	4.00157	6.29E-05	0.000476
MIR1244-3	143.945444	5.473168357	0.635518	8.612138	7.17E-18	4.32E-16
PTMAP4	176.988886	2.90931852	0.473873	6.139451	8.28E-10	1.85E-08
LINC02367	25.1397388	4.449730986	1.044195	4.261398	2.03E-05	0.000178
AC092821.3	10.3263363	4.222486144	1.259516	3.352467	0.000801	0.004044
CLEC2B	16.7168599	3.954074536	1.124044	3.517721	0.000435	0.002444
LINC02470	12.0454404	4.485805374	1.241611	3.612892	0.000303	0.001814
CLEC1A	12.2153053	4.056993238	1.279274	3.171325	0.001517	0.006827
TMEM52B	13.7909034	4.669103484	1.230113	3.795669	0.000147	0.000987
GABARAPL1	2283.18668	-2.072231406	0.348563	-5.94508	2.76E-09	5.71E-08
STYK1	11.9165818	4.437809462	1.245737	3.562397	0.000367	0.002126
ETV6	428.67626	-2.581483102	0.396844	-6.50503	7.77E-11	2.05E-09
BCL2L14	22.219495	5.274568791	1.194439	4.41594	1.01E-05	9.58E-05
PTMAP9	68.6008295	2.01878193	0.63758	3.166321	0.001544	0.00693
GPRC5A	11434.7222	-5.499374614	0.310659	-17.7023	4.03E-70	3.79E-67
AC022276.1	9.25446109	3.938692024	1.279145	3.07916	0.002076	0.008797
GRIN2B	87.8223363	4.811455435	0.777966	6.18466	6.22E-10	1.43E-08
PLBD1	297.919223	3.196728433	0.508708	6.284012	3.30E-10	7.82E-09
PLBD1-AS1	38.4431898	3.252059841	0.948292	3.429388	0.000605	0.003207
GUCY2C	17.3843292	5.031663074	1.204961	4.175788	2.97E-05	0.000247
ART4	16.6609594	4.901109753	1.216706	4.028179	5.62E-05	0.000431
ARHGDIB	88.1528798	-2.833725129	0.743583	-3.81091	0.000138	0.000939
RERG	13.7264741	4.638510683	1.233011	3.761939	0.000169	0.001109
PTPRO	68.2816969	2.496875691	0.753559	3.313444	0.000922	0.004543

LMO3	85.9615658	3.408454905	0.609215	5.594834	2.21E-08	3.86E-07
PIK3C2G	26.3669512	3.919636108	1.078771	3.633426	0.00028	0.001692
PLCZ1	17.4812359	3.868397134	1.15815	3.340153	0.000837	0.004189
PDE3A	54.8827204	5.865726391	0.927175	6.32645	2.51E-10	6.10E-09
SLCO1C1	16.1308776	4.8690777	1.218154	3.997097	6.41E-05	0.000482
SLCO1B1	9.62932542	4.002668209	1.275446	3.138251	0.0017	0.0075
SLCO1A2	54.8212354	3.954231018	0.859741	4.599326	4.24E-06	4.45E-05
SPX	134.943022	3.431190788	0.545574	6.289142	3.19E-10	7.59E-09
KCNJ8	148.933681	4.058675835	0.539757	7.519451	5.50E-14	2.20E-12
ABCC9	65.675577	3.239722194	0.75791	4.274548	1.92E-05	0.000169
AC084816.1	16.3947134	3.856943913	1.144919	3.368747	0.000755	0.003856
SOX5	161.401526	3.066276062	0.516708	5.934255	2.95E-09	6.06E-08
AC023796.1	9.38331971	4.017150993	1.273395	3.154678	0.001607	0.00717
BCAT1	5470.08171	-3.027651238	0.309151	-9.79344	1.20E-22	1.10E-20
STK38L	1461.6573	-2.222227888	0.335091	-6.63171	3.32E-11	9.31E-10
PTHLH	212.757279	3.935240724	0.534469	7.362899	1.80E-13	6.73E-12
AC024940.1	40.1563758	3.23875376	0.919834	3.521018	0.00043	0.002418
FGD4	581.404605	2.211559197	0.369557	5.984358	2.17E-09	4.55E-08
YARS2	459.634262	-2.010336387	0.406271	-4.94827	7.49E-07	9.55E-06
SYT10	39.7193335	3.710633764	0.918881	4.038211	5.39E-05	0.000416
ALG10	61.0428195	2.256257485	0.655761	3.440668	0.00058	0.003094
AC140847.2	14.3326968	4.804428726	1.218045	3.944378	8.00E-05	0.000585
DUX4L27	9.3188904	3.98167413	1.276003	3.120427	0.001806	0.007857
ALG10B	189.04956	2.212882239	0.464338	4.765673	1.88E-06	2.16E-05
KIF21A	2548.86671	2.887935992	0.332488	8.685846	3.76E-18	2.32E-16
ABCD2	49.4336147	4.303926059	0.787792	5.463275	4.67E-08	7.70E-07
C12orf40	8.78880857	3.89750018	1.280684	3.043295	0.00234	0.009716
SLC2A13	170.968515	3.669910434	0.509869	7.197746	6.12E-13	2.15E-11
LINC02471	15.7179428	4.6749555	1.236755	3.780019	0.000157	0.001042
LRRK2	52.4736944	4.192965993	0.853331	4.913648	8.94E-07	1.12E-05
MUC19	137.692674	2.227677804	0.690929	3.224177	0.001263	0.005873
CNTN1	1097.95072	3.66095469	0.349846	10.46449	1.26E-25	1.37E-23
ADAMTS20	31.6394454	3.336690594	0.96853	3.445107	0.000571	0.003054
NELL2	2291.48027	5.147096932	0.360913	14.26131	3.81E-46	1.44E-43
DBX2	13.653516	3.622671374	1.160286	3.122224	0.001795	0.007831
AC079950.1	10.7539184	4.178531017	1.264949	3.303319	0.000955	0.004683
SLC38A2	3509.60285	-2.795699283	0.32102	-8.70879	3.07E-18	1.92E-16
AMIGO2	1313.36606	-2.903364053	0.333232	-8.71275	2.97E-18	1.86E-16
PCED1B	70.1269209	-2.501559123	0.649749	-3.85004	0.000118	0.00082
LINC02156	10.4434834	4.166374392	1.26483	3.29402	0.000988	0.004787
RAPGEF3	363.026549	-2.92343831	0.476863	-6.13056	8.76E-10	1.95E-08
SLC48A1	1720.59875	-2.372982859	0.362715	-6.54227	6.06E-11	1.63E-09
HDAC7	4324.79573	-3.311876592	0.331297	-9.99671	1.58E-23	1.52E-21
COL2A1	47.0112539	4.374175052	0.942591	4.640587	3.47E-06	3.72E-05

AC004801.1	55.6514342	-3.021627536	0.731632	-4.12998	3.63E-05	0.000295
ADCY6	1477.64394	-2.75953979	0.332886	-8.28974	1.13E-16	6.10E-15
CACNB3	1117.61167	-2.455791019	0.336568	-7.29656	2.95E-13	1.08E-11
TUBA1B	1342.60065	-2.372701952	0.333971	-7.10452	1.21E-12	4.08E-11
TUBA1C	2554.70191	-2.118632778	0.415345	-5.1009	3.38E-07	4.69E-06
PRPH	46.3187704	3.283237177	0.865226	3.794658	0.000148	0.00099
KCNH3	79.9535455	2.893691907	0.611195	4.734484	2.20E-06	2.47E-05
LINC02395	16.0664483	4.83022072	1.222086	3.95244	7.74E-05	0.000568
AQP2	12.7834575	4.424631592	1.250368	3.538662	0.000402	0.002286
ASIC1	328.770569	-2.699945327	0.406479	-6.64227	3.09E-11	8.70E-10
AC074032.1	34.7157989	2.707196132	0.840107	3.222441	0.001271	0.005906
FAM186A	35.0818789	3.556148678	0.941527	3.777003	0.000159	0.001053
METTL7A	585.985134	4.041281261	0.382133	10.57559	3.87E-26	4.34E-24
GALNT6	4143.25099	2.241718515	0.310304	7.22427	5.04E-13	1.79E-11
TMDD1	18.863952	3.489482199	1.039683	3.356293	0.00079	0.004008
TAMALIN	278.628849	2.588982197	0.471607	5.4897	4.03E-08	6.71E-07
LINC00592	94.4244353	2.714362097	0.617069	4.398798	1.09E-05	0.000103
LINC02874	63.7233094	3.33321599	0.684254	4.871312	1.11E-06	1.35E-05
KRT7-AS	716.96249	-2.812333537	0.374077	-7.51806	5.56E-14	2.21E-12
KRT85	11.4128589	4.32259367	1.254608	3.445374	0.00057	0.003052
KRT84	12.00737	4.41656174	1.248133	3.538534	0.000402	0.002286
KRT75	10.0949779	4.005107431	1.276891	3.136609	0.001709	0.007522
KRT74	14.7485674	4.543772542	1.246109	3.646367	0.000266	0.001622
KRT77	16.479383	4.962719799	1.209182	4.104196	4.06E-05	0.000325
KRT76	9.53853722	4.032853085	1.27268	3.168788	0.001531	0.006875
KRT79	8.69802037	3.906713832	1.279695	3.052849	0.002267	0.009467
KRT8	124132.365	-4.658244179	0.354496	-13.1405	1.93E-39	5.03E-37
KRT18	55876.145	-5.165697522	0.381133	-13.5535	7.55E-42	2.24E-39
TNS2	273.209818	-2.005600377	0.42892	-4.67593	2.93E-06	3.21E-05
IGFBP6	1677.44512	-3.273685138	0.377768	-8.66586	4.48E-18	2.75E-16
AMHR2	19.4025626	3.911717219	1.116844	3.502473	0.000461	0.002568
CALCOCO1	3131.2634	-2.56664338	0.316983	-8.0971	5.63E-16	2.84E-14
HOXC11	11.2195709	4.169021091	1.267451	3.289296	0.001004	0.00485
HOXC6	12.62824	4.428525514	1.249417	3.544475	0.000393	0.00225
LINC02381	19.2007459	3.484910594	1.049364	3.320974	0.000897	0.004444
NFE2	71.0117207	4.854539571	0.744324	6.522077	6.93E-11	1.84E-09
ITGA5	6146.90762	-4.727030719	0.318622	-14.8358	8.59E-50	4.22E-47
NCKAP1L	43.1845045	3.471176741	0.944188	3.676362	0.000237	0.00147
PDE1B	18.0959874	4.984482375	1.2129	4.109557	3.96E-05	0.000319
MUCL1	16.1835954	4.568731868	1.248233	3.660159	0.000252	0.00155
NEUROD4	9.22810221	3.999043102	1.274302	3.138222	0.0017	0.0075
OR6C66P	9.72011361	3.945326647	1.280246	3.081694	0.002058	0.008746
OR6C4	11.9429407	4.379058258	1.251429	3.499245	0.000467	0.002588
OR6U2P	12.511093	4.525118401	1.239576	3.650538	0.000262	0.0016

RDH5	262.552207	2.756157196	0.464709	5.930932	3.01E-09	6.18E-08
TMEM198B	307.933567	-2.5152856	0.451191	-5.57476	2.48E-08	4.29E-07
DGKA	1192.26359	-3.892000879	0.386888	-10.0598	8.32E-24	8.13E-22
PMEL	92973.5538	7.830346499	0.389359	20.11086	5.93E-90	1.34E-86
ESYT1	3158.82713	-2.061817198	0.347091	-5.94027	2.85E-09	5.87E-08
RNF41	1682.5422	-2.151154886	0.326939	-6.57969	4.71E-11	1.29E-09
COQ10A	407.790735	2.065261367	0.461894	4.471285	7.78E-06	7.62E-05
PRIM1	93.5106668	2.244893906	0.602759	3.724363	0.000196	0.00126
MYO1A	20.1899559	5.160546469	1.200648	4.298133	1.72E-05	0.000154
STAT6	2031.98817	-2.031998076	0.323133	-6.28842	3.21E-10	7.62E-09
LRP1	11098.7206	-3.19264044	0.315711	-10.1125	4.86E-24	4.86E-22
GLI1	50.9465826	3.170411705	0.761396	4.163946	3.13E-05	0.000258
ARHGAP9	125.837634	4.532868418	0.665559	6.810622	9.72E-12	2.89E-10
AC083805.1	18.6377808	5.174755574	1.193271	4.336613	1.45E-05	0.000132
GIHCG	354.280855	2.156547887	0.480883	4.484562	7.31E-06	7.21E-05
AC108721.2	22.3483536	5.353585561	1.185639	4.515361	6.32E-06	6.34E-05
SLC16A7	424.000705	4.028646282	0.407336	9.890237	4.59E-23	4.32E-21
AC090629.1	17.1910413	4.929529807	1.215601	4.05522	5.01E-05	0.00039
AVPR1A	16.8278885	5.037007229	1.20215	4.189998	2.79E-05	0.000234
TBC1D30	640.550534	4.760501819	0.381207	12.48797	8.68E-36	1.74E-33
LINC02389	14.9799257	4.817339829	1.2192	3.951232	7.78E-05	0.00057
MSRB3	1510.84937	-2.47994773	0.329004	-7.53774	4.78E-14	1.92E-12
AC026124.1	9.78454293	4.008050513	1.275572	3.14216	0.001677	0.007433
HMGA2	1882.23815	-2.130903476	0.320632	-6.64595	3.01E-11	8.51E-10
TMBIM4	596.15974	2.472871044	0.389469	6.349344	2.16E-10	5.33E-09
IRAK3	28.4321505	3.389733668	1.019034	3.326417	0.00088	0.004369
LINC02408	11.4392177	4.228541376	1.263141	3.347639	0.000815	0.004096
LINC02384	17.5456652	3.905316954	1.149706	3.396797	0.000682	0.00354
CPM	414.75056	2.909584083	0.41073	7.083939	1.40E-12	4.66E-11
AC020656.2	17.9788403	5.092276912	1.200333	4.242387	2.21E-05	0.000192
YEATS4	708.465835	2.100289298	0.42761	4.911699	9.03E-07	1.13E-05
TSPAN8	29.772929	4.320960707	0.974673	4.433239	9.28E-06	8.93E-05
TPH2	18.5733515	5.150495683	1.195872	4.306897	1.66E-05	0.000149
LINC02882	34.3820825	6.020704914	1.138993	5.285991	1.25E-07	1.89E-06
KCNC2	24.6473216	5.663891834	1.155087	4.903433	9.42E-07	1.17E-05
GLIPR1L2	30.9344863	4.058203316	0.91612	4.429771	9.43E-06	9.05E-05
GLIPR1	2542.11336	-6.73636783	0.433283	-15.5473	1.66E-54	1.02E-51
AC107032.1	49.9564657	3.210762973	0.770793	4.165534	3.11E-05	0.000257
NAV3	256.962057	2.498032883	0.443518	5.632318	1.78E-08	3.17E-07
OTOGL	36.6810505	6.245706857	1.115092	5.601069	2.13E-08	3.74E-07
LIN7A	1221.2832	6.411649894	0.414458	15.46996	5.53E-54	3.29E-51
AC069228.1	28.4900946	4.450410137	1.08005	4.120558	3.78E-05	0.000306
TSPAN19	54.479373	4.739035887	0.806186	5.878338	4.14E-09	8.27E-08
LRRIQ1	187.113139	2.251563529	0.457724	4.919041	8.70E-07	1.09E-05

MGAT4C	59.8448992	4.505849123	0.815631	5.524372	3.31E-08	5.58E-07
MKRN9P	11.7496528	4.203681987	1.266356	3.31951	0.000902	0.004457
LINC02458	10.9999241	4.102900639	1.272157	3.225153	0.001259	0.005856
ATP2B1-AS1	56.2103976	2.521350386	0.680814	3.703434	0.000213	0.001344
LINC02822	40.4330353	5.057863664	1.01814	4.96775	6.77E-07	8.75E-06
LUM	1559.19304	-2.956386212	0.326146	-9.06461	1.25E-19	8.98E-18
DCN	797.891355	-2.362513735	0.418272	-5.64827	1.62E-08	2.90E-07
LINC02404	10.2501954	3.994010754	1.278276	3.124529	0.001781	0.007777
LINC01619	26.1382501	4.711570733	1.03609	4.547452	5.43E-06	5.55E-05
AC025164.1	51.7522032	3.832811371	0.764816	5.011415	5.40E-07	7.16E-06
LINC02397	21.0661329	3.677792558	1.075381	3.419989	0.000626	0.003303
RPL41P5	154.059469	2.33806617	0.545811	4.283657	1.84E-05	0.000163
PLXNC1	83.6069039	2.955301088	0.718293	4.11434	3.88E-05	0.000313
AC126615.1	53.8946727	3.120221452	0.747002	4.176989	2.95E-05	0.000246
AC084879.1	10.9735652	4.22904493	1.261362	3.35276	0.0008	0.00404
CCDC38	18.5884046	3.824021826	1.128261	3.389307	0.000701	0.00362
ELK3	1600.81401	-2.61511288	0.336253	-7.77723	7.41E-15	3.19E-13
CFAP54	73.2344377	3.353478496	0.7024	4.774316	1.80E-06	2.08E-05
RMST	27.223444	4.265706904	1.070078	3.98635	6.71E-05	0.000502
PAFAH1B2P2	10.6250598	4.049542664	1.275176	3.175673	0.001495	0.006735
TMPO-AS1	64.9999701	2.926225432	0.676287	4.3269	1.51E-05	0.000137
ANKS1B	68.9666125	3.514321892	0.744367	4.721222	2.34E-06	2.62E-05
GOLGA2P5	140.402338	2.613459165	0.514577	5.078853	3.80E-07	5.21E-06
MYBPC1	40.7547761	6.238526346	1.126675	5.537111	3.08E-08	5.23E-07
AC079907.2	12.6926693	4.473755964	1.245338	3.592403	0.000328	0.001939
HELLPAR	445.449343	5.630430129	0.619759	9.08487	1.04E-19	7.55E-18
LINC00485	15.0326435	4.668138074	1.235019	3.779812	0.000157	0.001042
PAH	20.2807441	5.100412324	1.207667	4.223361	2.41E-05	0.000206
AC068643.1	10.0686191	4.107514473	1.268446	3.238227	0.001203	0.005627
C12orf42	27.6608835	5.673922081	1.163815	4.875279	1.09E-06	1.33E-05
LINC02401	17.5659056	4.960923812	1.213556	4.087923	4.35E-05	0.000345
STAB2	45.5189186	2.718992856	0.837363	3.24709	0.001166	0.005489
AC078819.1	177.693197	-3.20406982	0.490925	-6.5266	6.73E-11	1.79E-09
TXNRD1	7829.42801	-3.316967415	0.306878	-10.8088	3.13E-27	3.81E-25
NUAK1	812.179911	-3.259065884	0.386144	-8.44003	3.17E-17	1.79E-15
RFX4	21.0421843	5.34188285	1.182395	4.517849	6.25E-06	6.27E-05
AC007695.1	14.4982973	4.102613556	1.182837	3.468453	0.000523	0.002843
BTBD11	567.886214	-2.126499783	0.375555	-5.66228	1.49E-08	2.69E-07
AC126177.3	14.373703	4.505843839	1.248403	3.609286	0.000307	0.001832
WSCD2	25.2945505	5.638134693	1.16065	4.85774	1.19E-06	1.43E-05
CMKLR1	20.9894665	5.413532828	1.173411	4.613499	3.96E-06	4.18E-05
LINC01498	21.2735426	5.448725216	1.170116	4.656567	3.22E-06	3.48E-05
SELPLG	112.39448	-4.32660841	0.657446	-6.58094	4.67E-11	1.28E-09
CORO1C	6875.86608	-3.33922303	0.315613	-10.5801	3.68E-26	4.15E-24

SSH1	1720.42509	-2.403140315	0.378499	-6.34914	2.17E-10	5.33E-09
DAO	19.2647694	4.057532165	1.134249	3.577284	0.000347	0.002029
SVOP	20.4096027	5.193907657	1.197602	4.336922	1.44E-05	0.000132
UNG	679.394209	-2.360781842	0.362819	-6.50677	7.68E-11	2.03E-09
ACACB	506.195253	2.167354457	0.372271	5.821982	5.82E-09	1.13E-07
FOXN4	13.9841914	4.740437774	1.223427	3.874722	0.000107	0.00075
KCTD10	2318.89317	-2.39131294	0.317235	-7.53799	4.77E-14	1.92E-12
TRPV4	177.457475	3.022628782	0.516631	5.850658	4.90E-09	9.62E-08
ANKRD13A	1085.6416	-2.301992672	0.338536	-6.79984	1.05E-11	3.11E-10
LINC01405	15.2259314	4.796445605	1.222436	3.923678	8.72E-05	0.000632
CUX2	30.8928273	5.155801239	1.081473	4.767388	1.87E-06	2.14E-05
PHETA1	837.604131	-2.80971647	0.354507	-7.9257	2.27E-15	1.06E-13
RPH3A	31.5825744	4.871683812	1.049612	4.641416	3.46E-06	3.71E-05
TPCN1	2077.84768	-2.614651911	0.32017	-8.16645	3.18E-16	1.64E-14
LHX5	10.0949779	4.005107431	1.276891	3.136609	0.001709	0.007522
LINC01234	39.3051008	6.335525708	1.109834	5.708534	1.14E-08	2.10E-07
TBX5	15.2786492	4.548576996	1.247368	3.646539	0.000266	0.001622
TBX5-AS1	10.8447066	4.116553698	1.270511	3.240076	0.001195	0.005595
LINC02457	10.4434834	4.166374392	1.26483	3.29402	0.000988	0.004787
MAP1LC3B2	117.707721	-2.136590486	0.533958	-4.00142	6.30E-05	0.000476
NOS1	40.3040179	5.386795252	0.970629	5.5498	2.86E-08	4.91E-07
KSR2	47.534589	3.119667218	0.853521	3.655056	0.000257	0.001576
SRRM4	67.6887699	2.215773195	0.653337	3.391471	0.000695	0.003595
CCDC60	22.0472199	3.80356991	1.045973	3.636393	0.000276	0.001678
TMEM233	106.984455	-2.399857554	0.567671	-4.22755	2.36E-05	0.000203
RAB35	1387.85716	-2.053984044	0.32651	-6.29072	3.16E-10	7.53E-09
PXN	1980.287	-3.279309117	0.322773	-10.1598	3.00E-24	3.02E-22
AC004263.2	163.331185	-2.285711347	0.480989	-4.75211	2.01E-06	2.29E-05
HNF1A-AS1	18.3156342	5.021387416	1.209627	4.151185	3.31E-05	0.000271
HNF1A	13.4043276	4.355513885	1.258772	3.460129	0.00054	0.002922
RPL21P1	13.9080505	4.552167927	1.242358	3.664136	0.000248	0.001529
HCAR1	16.933324	4.634096204	1.244413	3.723921	0.000196	0.001262
CDK2AP1	3429.99273	-2.099284969	0.343494	-6.11156	9.87E-10	2.18E-08
DNAH10	69.3352861	4.382227122	0.847715	5.169459	2.35E-07	3.36E-06
CCDC92	1349.36882	-2.281542872	0.347291	-6.56955	5.05E-11	1.38E-09
RFLNA	463.633769	-4.20393454	0.486339	-8.64404	5.43E-18	3.30E-16
AC026358.1	10.4434834	4.166374392	1.26483	3.29402	0.000988	0.004787
AC073592.3	9.51217834	4.071977747	1.269403	3.20779	0.001338	0.006166
SCARB1	4158.47009	-2.385804391	0.343974	-6.93601	4.03E-12	1.26E-10
AC122688.4	10.5342716	4.124847874	1.268711	3.25121	0.001149	0.005437
LINC00939	22.5275198	3.723865301	1.086248	3.42819	0.000608	0.003216
LINC02826	18.5681643	3.376641013	1.077113	3.134901	0.001719	0.007563
LINC02359	25.1012626	5.567321075	1.168917	4.762802	1.91E-06	2.19E-05
LINC02347	18.4562044	5.203525686	1.189164	4.375787	1.21E-05	0.000113

LINC00943	16.9073709	3.728867169	1.128379	3.304623	0.000951	0.004674
LINC00944	13.8172623	4.603767001	1.236903	3.722012	0.000198	0.001268
LINC02405	26.0179203	5.715505881	1.153172	4.956332	7.18E-07	9.22E-06
AC079949.3	10.5987009	4.173911508	1.264765	3.300148	0.000966	0.004718
AC079949.2	24.7944161	3.966905151	0.974186	4.072022	4.66E-05	0.000367
LINC02393	10.5987009	4.173911508	1.264765	3.300148	0.000966	0.004718
LINC00507	15.1234317	4.58060031	1.243877	3.682518	0.000231	0.001442
TMEM132C	22.7824601	3.64909231	1.061804	3.436691	0.000589	0.003131
LINC02418	14.063268	4.542184092	1.243891	3.651593	0.000261	0.001594
FZD10-AS1	21.0421843	5.34188285	1.182395	4.517849	6.25E-06	6.27E-05
PIWIL1	14.1013385	4.679991737	1.230232	3.804153	0.000142	0.000961
RIMBP2	49.8251308	5.598387727	0.930474	6.016708	1.78E-09	3.79E-08
AC063926.3	10.9091359	4.179411771	1.265451	3.302706	0.000958	0.004688
ADGRD1	43.2917465	4.324305513	0.970038	4.457873	8.28E-06	8.06E-05
LINC01257	12.0600878	4.135111753	1.272919	3.248527	0.00116	0.005468
ULK1	2352.77381	-2.617146582	0.319957	-8.17968	2.85E-16	1.48E-14
LINC02361	38.0778285	-3.739016603	0.935149	-3.99831	6.38E-05	0.000481
AC079031.1	10.5987009	4.173911508	1.264765	3.300148	0.000966	0.004718
P2RX2	10.4698423	4.061968818	1.273658	3.189215	0.001427	0.006475
FO681491.1	10.1330484	4.143717258	1.26561	3.274087	0.00106	0.005082
ZNF962P	10.2238366	4.116439207	1.268272	3.245707	0.001172	0.005504
ANKRD20A9P	12.0337289	4.332064733	1.25612	3.448766	0.000563	0.003025
PHF2P2	16.5965301	4.858850005	1.221017	3.979348	6.91E-05	0.000515
ANKRD26P3	20.324933	4.106416371	1.135529	3.616304	0.000299	0.001792
TPTE2	19.8150916	5.133966142	1.202356	4.269923	1.96E-05	0.000172
GJB6	14.373703	4.505843839	1.248403	3.609286	0.000307	0.001832
IL17D	123.449188	2.476858315	0.52052	4.758428	1.95E-06	2.23E-05
LATS2	886.535836	-2.505750005	0.346179	-7.23832	4.54E-13	1.63E-11
ESRRAP2	11.193212	4.276943502	1.257921	3.400009	0.000674	0.003502
LINC00540	13.2227512	4.519365893	1.242964	3.635958	0.000277	0.001678
FTH1P7	431.725459	-2.516079343	0.517062	-4.86611	1.14E-06	1.38E-05
ATP12A	18.4064224	4.962733688	1.216338	4.08006	4.50E-05	0.000356
RNF17	34.8551907	3.335229458	0.948957	3.514627	0.00044	0.00247
AMER2	24.1816691	5.65142082	1.155049	4.892796	9.94E-07	1.23E-05
SHISA2	532.399043	5.686954462	0.474551	11.98386	4.32E-33	7.54E-31
ATP8A2P3	10.5987009	4.173911508	1.264765	3.300148	0.000966	0.004718
CDX2	16.002019	4.784072071	1.226707	3.89993	9.62E-05	0.000687
FLT1	43.5721143	6.378696081	1.114614	5.722783	1.05E-08	1.94E-07
MTUS2	167.974565	3.358763389	0.488847	6.870781	6.39E-12	1.95E-10
MTUS2-AS1	8.91766719	3.955761029	1.276665	3.09851	0.001945	0.008364
LINC00365	18.4064224	4.962733688	1.216338	4.08006	4.50E-05	0.000356
LINC00384	14.2184855	4.527395237	1.245853	3.633973	0.000279	0.001689
LINC00426	21.820126	3.954749822	1.093411	3.616891	0.000298	0.001788
AL161893.1	10.572342	4.23381264	1.259403	3.361761	0.000774	0.003938

TEX26-AS1	28.7336901	3.661109631	0.995679	3.676999	0.000236	0.001468
MEDAG	254.633875	-3.614518036	0.495167	-7.29959	2.89E-13	1.06E-11
RXFP2	15.8468015	4.790882526	1.22542	3.909584	9.25E-05	0.000663
BRCA2	82.7069946	2.82356431	0.661647	4.267477	1.98E-05	0.000174
N4BP2L1	143.443512	2.331256767	0.513028	4.544111	5.52E-06	5.61E-05
AL138820.1	13.8816916	4.642384313	1.233234	3.764399	0.000167	0.001101
KL	32.833496	4.424503593	0.919544	4.811626	1.50E-06	1.77E-05
AL139383.1	29.6733651	4.268748649	0.993236	4.297817	1.72E-05	0.000154
DCLK1	939.379729	4.714538377	0.363479	12.97058	1.80E-38	4.32E-36
AL356274.2	10.0041897	4.063829218	1.271846	3.195222	0.001397	0.006372
POSTN	5759.00867	-4.898653176	0.315304	-15.5363	1.97E-54	1.19E-51
FREM2	67.6533933	4.106982142	0.737683	5.567409	2.59E-08	4.47E-07
AL354809.1	57.6842415	5.382800376	0.926566	5.809408	6.27E-09	1.21E-07
LHFPL6	2387.60995	3.703833415	0.345596	10.71723	8.45E-27	9.94E-25
LINC00598	69.2979552	5.045995489	0.899786	5.607993	2.05E-08	3.61E-07
FOXO1	575.689068	2.007642186	0.361575	5.552498	2.82E-08	4.83E-07
RGCC	959.467939	8.066726408	0.551761	14.61997	2.09E-48	9.10E-46
AL354833.1	14.658185	3.497479764	1.151565	3.037154	0.002388	0.009879
FAM216B	9.72011361	3.945326647	1.280246	3.081694	0.002058	0.008746
ENOX1	90.908969	3.679526602	0.619855	5.936107	2.92E-09	6.00E-08
ENOX1-AS1	11.8140821	4.278206312	1.260145	3.395012	0.000686	0.003556
AL162713.2	20.3338677	3.906703704	1.130236	3.456537	0.000547	0.002957
DGKZP1	173.094065	-2.389456273	0.475355	-5.02667	4.99E-07	6.66E-06
LINC00390	16.9718003	3.748225614	1.124403	3.333524	0.000858	0.004278
AL589745.1	15.0124032	3.623801719	1.181935	3.06599	0.00217	0.009122
CPB2-AS1	63.8065623	3.351732894	0.789116	4.247452	2.16E-05	0.000188
CPB2	16.8603659	3.869951202	1.148705	3.368968	0.000755	0.003853
LRRC63	59.3865435	3.589915332	0.713383	5.032243	4.85E-07	6.50E-06
LINC01198	11.8257936	4.449235975	1.24429	3.575724	0.000349	0.002038
HTR2A	46.8717338	5.248638192	0.885059	5.930268	3.02E-09	6.20E-08
LPAR6	49.5177367	3.02435031	0.726237	4.164412	3.12E-05	0.000258
LINC00462	19.1649268	5.312465256	1.17924	4.504991	6.64E-06	6.61E-05
CYSLTR2	44.0933888	6.634586623	1.080359	6.141093	8.20E-10	1.84E-08
SETDB2	438.059137	2.285591696	0.382635	5.973298	2.33E-09	4.86E-08
DLEU1	138.285925	2.947091878	0.581174	5.070926	3.96E-07	5.41E-06
DLEU7	82.3827618	5.922138859	0.799933	7.403295	1.33E-13	5.09E-12
RNASEH2B-AS1	26.842867	4.270094899	1.00456	4.25071	2.13E-05	0.000186
GUCY1B2	21.1974018	5.342100178	1.18294	4.515953	6.30E-06	6.32E-05
C13orf42	10.8447066	4.116553698	1.270511	3.240076	0.001195	0.005595
SERPINE3	9486.87901	3.181459938	0.306083	10.39411	2.64E-25	2.84E-23
INTS6	4783.66852	3.529713175	0.342584	10.30319	6.82E-25	7.13E-23
NEK5	71.6218997	3.375399313	0.70709	4.773647	1.81E-06	2.08E-05
TPTE2P2	11.3220707	4.345514698	1.25213	3.470498	0.000519	0.002824
THSD1	350.615638	-3.331304336	0.410257	-8.12004	4.66E-16	2.36E-14

AL137058.1	13.4602281	3.568717868	1.17021	3.049638	0.002291	0.009554
CNMD	70.4920133	7.291506939	1.039732	7.012872	2.33E-12	7.52E-11
PCDH8	12.00737	4.41656174	1.248133	3.538534	0.000402	0.002286
LINC00558	13.6884037	4.501752988	1.246408	3.611783	0.000304	0.001818
AL390964.1	15.9554197	3.795216489	1.154078	3.288528	0.001007	0.004862
PCDH17	837.3143	5.668966451	0.382399	14.82473	1.01E-49	4.84E-47
AL159156.1	11.3601411	4.39794495	1.247419	3.525637	0.000422	0.002383
AL359208.1	23.39387	5.565514386	1.16321	4.784616	1.71E-06	1.99E-05
OR7E104P	11.7877232	4.373743725	1.25132	3.495305	0.000474	0.002615
LINC00355	10.5987009	4.173911508	1.264765	3.300148	0.000966	0.004718
PCDH9	345.999666	2.205097542	0.437038	5.045552	4.52E-07	6.11E-06
LINC00550	11.8785114	4.334365593	1.255325	3.452784	0.000555	0.002989
KLHL1	14.5025617	4.638184454	1.236066	3.752377	0.000175	0.001146
ATXN8OS	25.1539803	5.398057643	1.18929	4.538892	5.66E-06	5.74E-05
LINC00347	9.1636729	3.96679767	1.276628	3.107246	0.001888	0.008154
TBC1D4	391.450188	-2.42477004	0.448871	-5.40193	6.59E-08	1.05E-06
LMO7	497.219956	-2.288741067	0.395903	-5.78106	7.42E-09	1.41E-07
AL136441.1	25.1539803	5.398057643	1.18929	4.538892	5.66E-06	5.74E-05
KCTD12	449.651558	2.772142175	0.398316	6.959664	3.41E-12	1.08E-10
SCEL	14.5890855	4.075086939	1.189063	3.427143	0.00061	0.003226
EDNRB	844.476484	7.039773505	0.434591	16.19863	5.16E-59	3.72E-56
OBI1-AS1	17.8184356	3.315436095	1.082781	3.061964	0.002199	0.009222
LINC01069	14.9682142	4.603296853	1.241181	3.708802	0.000208	0.001321
AL158064.1	33.2574896	5.977157354	1.141561	5.23595	1.64E-07	2.42E-06
AL137781.1	18.3537047	5.123141281	1.19821	4.275661	1.91E-05	0.000168
AL590807.1	28.170725	4.643420732	1.080756	4.296454	1.74E-05	0.000155
PTMAP5	2931.93075	4.193509246	0.318938	13.14834	1.74E-39	4.57E-37
AL355481.1	14.5933499	4.56598022	1.243478	3.671942	0.000241	0.00149
SLITRK6	17.0358238	4.93378004	1.214557	4.062206	4.86E-05	0.00038
MIR4500HG	62.1345659	5.141300521	0.895153	5.743486	9.27E-09	1.74E-07
SLITRK5	677.516278	4.096134108	0.365546	11.20553	3.83E-29	5.28E-27
AL445647.1	11.2195709	4.169021091	1.267451	3.289296	0.001004	0.00485
LINC02336	10.2501954	3.994010754	1.278276	3.124529	0.001781	0.007777
LINC00559	16.8425358	4.775281338	1.230546	3.880621	0.000104	0.000735
GPC5	23.649177	3.934291674	1.02511	3.837921	0.000124	0.000853
DCT	16474.0173	14.44958761	0.787209	18.35547	2.98E-75	4.40E-72
SOX21-AS1	14.063268	4.542184092	1.243891	3.651593	0.000261	0.001594
SOX21	11.3747884	4.154235208	1.269217	3.273069	0.001064	0.00509
CLDN10	67.8838057	3.261593983	0.696259	4.684454	2.81E-06	3.09E-05
DNAJC3-DT	112.771616	3.127473517	0.571629	5.471163	4.47E-08	7.41E-07
HS6ST3	48.1117394	5.624409149	0.958258	5.869407	4.37E-09	8.67E-08
OXGR1	28.2292827	4.853377376	1.019068	4.762563	1.91E-06	2.19E-05
MBNL2	1315.25449	-2.316485237	0.328695	-7.04751	1.82E-12	5.97E-11
SLC15A1	15.4897672	3.779089082	1.150784	3.283926	0.001024	0.004929

LINC01232	21.6740264	3.524819534	1.022258	3.448071	0.000565	0.00303
CLYBL	364.217088	3.553937662	0.409654	8.675459	4.12E-18	2.53E-16
AL137139.2	30.7814651	3.244015395	0.902084	3.596135	0.000323	0.001916
ZIC2	10.7539184	4.178531017	1.264949	3.303319	0.000955	0.004683
FGF14	42.5376422	3.319058776	0.821413	4.04067	5.33E-05	0.000412
FGF14-AS2	105.056425	2.717619567	0.589254	4.611964	3.99E-06	4.21E-05
CCDC168	53.2277986	6.649539506	1.098625	6.052602	1.43E-09	3.07E-08
SLC10A2	10.4698423	4.061968818	1.273658	3.189215	0.001427	0.006475
LINC00343	33.8637122	6.105993681	1.126144	5.422036	5.89E-08	9.54E-07
AL138689.2	24.2644931	3.545439729	1.074761	3.298818	0.000971	0.004726
LINC00551	12.8478869	4.474722687	1.24585	3.591704	0.000329	0.001942
MYO16	147.641842	3.729970332	0.538572	6.92567	4.34E-12	1.35E-10
AL161431.1	146.738823	-3.865141169	0.600217	-6.43957	1.20E-10	3.07E-09
COL4A1	21145.7807	-4.013936247	0.332482	-12.0727	1.47E-33	2.61E-31
COL4A2	37796.5602	-4.142841752	0.328562	-12.609	1.88E-36	3.89E-34
SOX1-OT	11.7496528	4.203681987	1.266356	3.31951	0.000902	0.004457
LINC01043	24.6883278	5.44008247	1.183012	4.598502	4.26E-06	4.46E-05
ATP11AUN	11.4392177	4.228541376	1.263141	3.347639	0.000815	0.004096
MCF2L	280.062344	3.553538635	0.450511	7.887801	3.08E-15	1.40E-13
F7	14.7019681	3.625532786	1.176744	3.080986	0.002063	0.008762
KARS1P2	11.1287827	4.232433809	1.261651	3.354678	0.000795	0.004025
LAMP1	10985.533	-3.03282869	0.320869	-9.45192	3.33E-21	2.72E-19
GRK1	12.8742457	4.341881084	1.258229	3.450788	0.000559	0.003008
GAS6	8026.04227	-3.436327702	0.386418	-8.89277	5.96E-19	4.03E-17
GAS6-DT	136.138007	-3.305533724	0.588177	-5.61996	1.91E-08	3.38E-07
RASA3	578.69854	-2.20863739	0.364913	-6.05251	1.43E-09	3.07E-08
CFAP97D2	12.00737	4.41656174	1.248133	3.538534	0.000402	0.002286
AL589743.3	24.8816157	5.543155084	1.171183	4.732953	2.21E-06	2.49E-05
LINC01297	11.6442172	4.449284153	1.243678	3.577521	0.000347	0.002027
MED15P6	10.9354948	3.987525477	1.280888	3.113095	0.001851	0.008019
AL512310.7	12.2914461	4.487496012	1.242404	3.611945	0.000304	0.001818
ARHGAP42P4	15.1615021	4.761005321	1.225946	3.883536	0.000103	0.000729
OR4N2	17.4751174	5.001808518	1.208684	4.138227	3.50E-05	0.000285
OR4M1	14.7222085	4.681425272	1.232515	3.798272	0.000146	0.000979
OR4K2	28.1885551	4.10852204	1.035442	3.967893	7.25E-05	0.000537
OR4Q2	9.93976043	4.008663455	1.276073	3.141406	0.001681	0.007444
OR4K13	13.0031044	4.473281904	1.246584	3.588433	0.000333	0.001957
OR4K17	14.1276973	4.598912982	1.238579	3.713055	0.000205	0.001303
OR4N5	10.5342716	4.124847874	1.268711	3.25121	0.001149	0.005437
KLHL33	16.1750666	3.826444413	1.149447	3.328943	0.000872	0.004333
AL355075.5	10.9354948	3.987525477	1.280888	3.113095	0.001851	0.008019
RNASE9	10.0041897	4.063829218	1.271846	3.195222	0.001397	0.006372
ANG	45.3465874	2.613457228	0.786046	3.324814	0.000885	0.00439
NDRG2	367.593505	3.832611083	0.455409	8.415761	3.90E-17	2.17E-15

ZNF219	2513.83153	-3.240439042	0.317459	-10.2074	1.84E-24	1.87E-22
OR5AU1	19.393628	4.10794267	1.122517	3.659582	0.000253	0.001553
RPGRIP1	33.2954097	3.570500814	0.888783	4.017291	5.89E-05	0.000449
SALL2	1107.03949	-2.292398831	0.334228	-6.85879	6.94E-12	2.11E-10
OR10G3	14.1276973	4.598912982	1.238579	3.713055	0.000205	0.001303
OR4E2	15.498296	4.616383185	1.241704	3.71778	0.000201	0.001284
AC245505.2	12.00737	4.41656174	1.248133	3.538534	0.000402	0.002286
AC244502.1	25.7221326	5.54706044	1.173418	4.727266	2.28E-06	2.55E-05
TRAC	54.5122024	-4.11295867	0.772074	-5.32715	9.98E-08	1.54E-06
OR6J1	10.9091359	4.179411771	1.265451	3.302706	0.000958	0.004688
SLC7A7	569.429542	-3.169354998	0.383079	-8.27337	1.30E-16	6.97E-15
MMP14	33982.036	-2.981138768	0.328451	-9.07636	1.12E-19	8.13E-18
AL132780.1	27.1935932	3.125784651	0.989622	3.158564	0.001585	0.007091
AJUBA	2630.1003	-2.850940143	0.315445	-9.03784	1.60E-19	1.14E-17
AL132780.4	39.0533922	-2.66654457	0.827144	-3.2238	0.001265	0.005879
CDH24	1008.85435	-2.001906789	0.347667	-5.75811	8.51E-09	1.60E-07
CMTM5	12.821528	4.544094362	1.238932	3.667753	0.000245	0.001511
MYH6	35.4158873	6.088368721	1.13291	5.374098	7.70E-08	1.21E-06
MYH7	53.0725811	6.653432113	1.097778	6.060816	1.35E-09	2.92E-08
JPH4	21.9111366	3.698095662	1.093415	3.382151	0.000719	0.003699
AL160237.1	10.2882659	4.156236444	1.265118	3.285256	0.001019	0.004906
AL136419.3	11.4772882	4.356824519	1.251683	3.480772	0.0005	0.002731
CARMIL3	76.8016318	3.45143746	0.636631	5.421407	5.91E-08	9.57E-07
CPNE6	16.3768833	4.818032405	1.224528	3.934605	8.33E-05	0.000606
NRL	80.2952321	2.810320033	0.611863	4.593053	4.37E-06	4.57E-05
TGM1	166.520639	-2.759225795	0.61179	-4.51008	6.48E-06	6.47E-05
ADCY4	27.3805157	3.591298189	1.056399	3.399567	0.000675	0.003507
STXBP6	266.146699	2.084727564	0.433638	4.807534	1.53E-06	1.80E-05
LINC02306	10.2501954	3.994010754	1.278276	3.124529	0.001781	0.007777
AL359396.2	9.3188904	3.98167413	1.276003	3.120427	0.001806	0.007857
AL132633.1	16.0928072	4.709305251	1.23463	3.814347	0.000137	0.000928
LINC02588	36.8704798	4.842360207	0.930034	5.206646	1.92E-07	2.80E-06
AL110292.1	15.6007958	4.833628509	1.219919	3.962255	7.42E-05	0.000548
LINC02293	19.088786	5.290955156	1.181201	4.479303	7.49E-06	7.36E-05
MIR4307HG	11.5036471	4.283732053	1.258499	3.403843	0.000664	0.003466
LINC00645	74.0005225	7.393293891	1.029877	7.178814	7.03E-13	2.44E-11
FOXG1-AS1	14.9418553	4.722241593	1.22915	3.841875	0.000122	0.000841
FOXG1	9.84897224	4.056445929	1.271884	3.189321	0.001426	0.006475
LINC01551	38.4148019	6.193121183	1.126906	5.495684	3.89E-08	6.52E-07
LINC02327	18.1340579	5.093999888	1.200735	4.242401	2.21E-05	0.000192
AL135878.1	23.6925934	5.449234583	1.178709	4.623053	3.78E-06	4.02E-05
AL133166.1	39.6946124	6.225309404	1.125845	5.529456	3.21E-08	5.45E-07
AL158058.2	9.3188904	3.98167413	1.276003	3.120427	0.001806	0.007857
AL161665.2	23.4265943	3.903811763	1.037524	3.762622	0.000168	0.001107

AKAP6	202.42719	2.696637806	0.474431	5.683945	1.32E-08	2.40E-07
AL049781.1	9.87533112	3.936139891	1.281456	3.071616	0.002129	0.008981
NPAS3	80.2774435	2.778054307	0.646382	4.29785	1.72E-05	0.000154
INSM2	10.0686191	4.107514473	1.268446	3.238227	0.001203	0.005627
PAX9	20.6997974	4.132996576	1.133384	3.6466	0.000266	0.001622
TTC6	34.9069772	4.300332756	1.001333	4.294606	1.75E-05	0.000156
AL049828.1	63.9729353	3.057134826	0.724975	4.216883	2.48E-05	0.000212
AL390800.1	16.7517476	4.85108935	1.222407	3.968473	7.23E-05	0.000536
AL121821.2	119.114554	2.285315609	0.561594	4.069336	4.71E-05	0.000371
LRFN5	156.765972	3.864584281	0.524186	7.372542	1.67E-13	6.29E-12
MDGA2	33.8783596	5.955223821	1.146096	5.196095	2.04E-07	2.95E-06
RN7SL1	3311.90004	2.984847026	0.452781	6.592248	4.33E-11	1.19E-09
LINC01588	46.593165	2.957816721	0.832055	3.554834	0.000378	0.00218
PYGL	1300.83762	-2.043917911	0.332522	-6.14671	7.91E-10	1.78E-08
FRMD6	1265.19325	-2.130900993	0.338231	-6.30013	2.97E-10	7.12E-09
AL079307.1	16.1572365	4.774355185	1.228275	3.887041	0.000101	0.000721
COX5AP2	10.118401	4.189169896	1.262074	3.319273	0.000903	0.004459
TXNDC16	200.517436	2.090218488	0.449349	4.651655	3.29E-06	3.55E-05
GPR137C	112.139457	2.59740692	0.545184	4.764273	1.90E-06	2.17E-05
AL365295.1	52.9688885	4.043286043	0.798328	5.064693	4.09E-07	5.57E-06
LGALS3	410.044215	-2.640251531	0.420041	-6.28569	3.26E-10	7.75E-09
PELI2	244.321825	2.365168924	0.428972	5.513577	3.52E-08	5.92E-07
OTX2	4865.66644	2.950941578	0.36245	8.141659	3.90E-16	2.00E-14
OTX2-AS1	189.170472	3.49233375	0.535243	6.524759	6.81E-11	1.81E-09
AL161757.4	12.3939459	4.555758959	1.236144	3.685459	0.000228	0.001429
SLC35F4	15.3284312	4.892293615	1.212417	4.035158	5.46E-05	0.000421
TIMM9	855.29235	2.130628303	0.422319	5.045072	4.53E-07	6.12E-06
LINC01500	27.7897422	5.7282997	1.157365	4.949433	7.44E-07	9.50E-06
AL049873.2	24.4223287	3.988189097	1.020812	3.906881	9.35E-05	0.000669
DAAM1	4502.76115	3.546853116	0.335111	10.58412	3.53E-26	3.99E-24
RTN1	170.586334	-2.396917256	0.523194	-4.58132	4.62E-06	4.80E-05
LRRC9	18.6260693	5.004139642	1.212623	4.126708	3.68E-05	0.000298
SALL4P7	10.6250598	4.049542664	1.275176	3.175673	0.001495	0.006735
SIX1	23.865641	4.458277686	1.083836	4.113425	3.90E-05	0.000314
TMEM30B	16.6143602	3.886086759	1.140676	3.406829	0.000657	0.003443
AL359220.1	17.7653121	3.937337225	1.1446	3.439925	0.000582	0.003102
AL355916.2	9.75818404	4.081877292	1.269445	3.215482	0.001302	0.006028
AL355916.1	60.1125558	-2.773574101	0.728782	-3.80577	0.000141	0.000956
HIF1A	6578.89273	-3.199383324	0.329519	-9.70924	2.75E-22	2.47E-20
SYT16	45.8005038	3.984980604	0.93869	4.245257	2.18E-05	0.00019
LINC00643	16.5352836	3.956920638	1.120664	3.530871	0.000414	0.002341
KCNH5	39.2538399	3.572382658	0.944881	3.780775	0.000156	0.00104
RHOJ	447.243991	-4.082621948	0.424286	-9.62234	6.43E-22	5.59E-20
HSPE1P2	114.075361	-2.023195516	0.649754	-3.11379	0.001847	0.008013

SYNE2	938.546757	3.460416562	0.398283	8.688338	3.68E-18	2.27E-16
ESR2	27.6725951	5.793685251	1.148543	5.044377	4.55E-07	6.14E-06
LINC02324	10.0949779	4.005107431	1.276891	3.136609	0.001709	0.007522
YBX1P1	298.858903	-3.057154435	0.470761	-6.49407	8.35E-11	2.19E-09
LINC02290	18.8076457	4.783820137	1.235635	3.871548	0.000108	0.000758
PLEKHH1	957.844093	3.215514887	0.373941	8.598988	8.04E-18	4.82E-16
AL132640.2	13.5448977	4.676080843	1.228401	3.80664	0.000141	0.000954
AL121820.3	16.5818828	5.038222964	1.201048	4.194857	2.73E-05	0.00023
AL121820.2	52.520262	4.411477056	0.90204	4.890558	1.01E-06	1.24E-05
ACTN1	22184.5766	-4.383507589	0.304419	-14.3996	5.20E-47	2.05E-44
CCDC177	14.9418553	4.722241593	1.22915	3.841875	0.000122	0.000841
SLC8A3	28.7947778	4.20897384	1.003916	4.192555	2.76E-05	0.000232
AC004825.3	10.4171245	4.218020553	1.26022	3.347052	0.000817	0.004102
AC004817.5	14.5289206	4.474807258	1.251749	3.574843	0.00035	0.002043
AC004817.4	11.0907123	3.911858271	1.286693	3.040241	0.002364	0.009795
RGS6	31.2871925	4.175593256	0.999736	4.176696	2.96E-05	0.000246
AL442663.3	24.2060235	3.531934592	0.955741	3.695492	0.000219	0.001381
AC005480.1	10.3790541	4.122386704	1.268347	3.250204	0.001153	0.005447
VRTN	11.5036471	4.283732053	1.258499	3.403843	0.000664	0.003466
SYNDIG1L	12.4085932	4.379167019	1.253201	3.494385	0.000475	0.002618
LTBP2	4519.13767	-5.87212483	0.355438	-16.5208	2.60E-61	1.91E-58
RPS6KL1	251.231604	-2.400064783	0.444847	-5.39526	6.84E-08	1.09E-06
PGF	177.464971	2.26176755	0.477311	4.738561	2.15E-06	2.43E-05
FLVCR2	236.306801	4.257812867	0.651454	6.535858	6.32E-11	1.69E-09
ESRRB	19.3875095	5.232312826	1.189344	4.399328	1.09E-05	0.000103
RPL22P2	78.0392799	-3.514843542	0.7077	-4.96657	6.81E-07	8.79E-06
AHSA1	2451.70274	-2.013981119	0.369644	-5.44843	5.08E-08	8.31E-07
ISM2	16.5701712	4.936887429	1.212424	4.071916	4.66E-05	0.000367
AC008056.3	11.2195709	4.169021091	1.267451	3.289296	0.001004	0.00485
TSHR	32.1446081	6.030466227	1.131232	5.330883	9.77E-08	1.51E-06
AC007262.2	21.3384975	3.62353755	1.101047	3.290994	0.000998	0.004829
LINC02301	19.3381333	3.884924732	1.122889	3.459759	0.000541	0.002926
LINC02328	56.5123735	4.245344223	0.762965	5.564271	2.63E-08	4.54E-07
AL359237.1	9.3188904	3.98167413	1.276003	3.120427	0.001806	0.007857
GPR65	10.6250598	4.049542664	1.275176	3.175673	0.001495	0.006735
LINC01146	21.3789782	5.275992178	1.191457	4.428185	9.50E-06	9.11E-05
KCNK10	18.8574276	5.200648692	1.191063	4.366394	1.26E-05	0.000117
EML5	43.7487675	2.591666223	0.834401	3.106019	0.001896	0.008185
AL121768.1	8.94402608	3.914238868	1.279942	3.058139	0.002227	0.009318
AL139193.2	9.4096786	3.945690492	1.279149	3.084622	0.002038	0.008693
AL122020.1	25.5679736	3.403423944	1.008381	3.375139	0.000738	0.003782
LINC02321	17.6015658	3.417240681	1.099529	3.107914	0.001884	0.008137
RPS6KA5	196.933511	3.704361841	0.475913	7.783698	7.04E-15	3.06E-13
AL133373.3	13.7706631	3.589067597	1.170453	3.066392	0.002167	0.009114

CATSPERB	38.1456201	5.300527459	0.978882	5.414881	6.13E-08	9.87E-07
AL121839.2	39.2470369	4.771013037	0.916549	5.205411	1.94E-07	2.81E-06
TC2N	488.177237	4.494616156	0.408606	10.99987	3.83E-28	4.99E-26
SLC24A4	56.185707	6.8413776	1.07769	6.348184	2.18E-10	5.36E-09
RIN3	524.503958	-2.364480179	0.369975	-6.39092	1.65E-10	4.12E-09
ITPK1-AS1	12.3441639	4.315094906	1.258772	3.428019	0.000608	0.003216
TMEM251	218.343329	2.005677195	0.495726	4.045943	5.21E-05	0.000404
PRIMA1	16.8425358	4.775281338	1.230546	3.880621	0.000104	0.000735
AL132642.2	10.405413	3.972768242	1.280392	3.102774	0.001917	0.00826
FAM181A-AS1	17.0885415	4.549678346	1.252339	3.632945	0.00028	0.001694
ASB2	22.3195845	3.787247908	1.057822	3.580231	0.000343	0.002009
PPP4R4	150.300886	3.26846185	0.525831	6.215802	5.11E-10	1.18E-08
SERPINA10	10.9354948	3.987525477	1.280888	3.113095	0.001851	0.008019
SERPINA1	740.349425	5.31296802	0.488837	10.86859	1.63E-27	2.03E-25
SERPINA5	85.2976778	2.095361465	0.616672	3.397853	0.000679	0.003528
AL121612.2	10.9091359	4.179411771	1.265451	3.302706	0.000958	0.004688
LINC02318	9.56489611	3.948402431	1.27948	3.085944	0.002029	0.008665
TCL6	25.7903093	3.585589398	1.084582	3.305964	0.000947	0.004655
AK7	369.327471	2.888509136	0.399775	7.22534	5.00E-13	1.77E-11
LINC02325	27.0400135	5.691275949	1.159682	4.907619	9.22E-07	1.15E-05
CYP46A1	109.459403	2.239897509	0.537672	4.165916	3.10E-05	0.000256
WARS1	1322.45381	-2.053718384	0.363035	-5.65709	1.54E-08	2.77E-07
BEGAIN	173.190904	-2.532067874	0.58299	-4.34324	1.40E-05	0.000129
LINC00523	12.2533757	4.381831723	1.252369	3.498834	0.000467	0.002588
DLK1	50.7762483	3.241936947	0.877839	3.693087	0.000222	0.001393
AL355836.2	11.4392177	4.228541376	1.263141	3.347639	0.000815	0.004096
AL355032.1	37.4526366	2.749189405	0.879062	3.127412	0.001764	0.007728
RCOR1	1719.65062	-2.140819534	0.324436	-6.59859	4.15E-11	1.15E-09
LBHD2	21.6864774	5.503728502	1.164823	4.724948	2.30E-06	2.58E-05
LINC00605	159.298921	6.653760524	0.702756	9.468088	2.85E-21	2.34E-19
RPL10AP1	34.4658063	3.312034831	0.918242	3.60693	0.00031	0.001846
TDRD9	47.2617795	3.308085952	0.816231	4.052878	5.06E-05	0.000394
ASPG	117.052756	5.412539094	0.669245	8.087527	6.09E-16	3.07E-14
TMEM179	33.5434834	4.769890736	0.942136	5.062845	4.13E-07	5.62E-06
C14orf180	10.5606305	3.934530818	1.283673	3.065058	0.002176	0.009142
CEP170B	2624.0938	-2.461805198	0.318727	-7.72387	1.13E-14	4.80E-13
PLD4	12.1362286	4.476236461	1.242888	3.60148	0.000316	0.001882
AHNAK2	1472.7705	-4.047865243	0.483231	-8.37667	5.44E-17	3.01E-15
JAG2	1069.10366	-2.150039396	0.378277	-5.68377	1.32E-08	2.40E-07
MTA1	2811.04353	-2.077455432	0.320835	-6.47514	9.47E-11	2.46E-09
CRIP2	1809.33622	-2.679139413	0.394869	-6.78488	1.16E-11	3.43E-10
CRIP1	27.1200693	-3.868676937	0.993449	-3.89419	9.85E-05	0.000702
IGHE	11.0379945	4.269395787	1.258002	3.393791	0.000689	0.003567
IGHGP	13.6620448	4.602670925	1.236404	3.722627	0.000197	0.001267

IGHG3	13.2871805	4.560426404	1.239161	3.680253	0.000233	0.001453
FAM30A	31.2162389	5.733811377	1.166539	4.915235	8.87E-07	1.11E-05
AC246787.1	148.819175	2.001925232	0.528212	3.790001	0.000151	0.001006
LINC00221	13.2491101	4.384912975	1.255665	3.492104	0.000479	0.002637
AC087386.2	12.8098164	4.225515716	1.267898	3.332692	0.00086	0.004288
RHPN2P1	29.5352052	5.761750111	1.158449	4.973675	6.57E-07	8.50E-06
CHEK2P2	19.0917218	4.954989948	1.219411	4.063428	4.84E-05	0.000378
AC026495.1	16.0664483	4.83022072	1.222086	3.95244	7.74E-05	0.000568
AC131280.1	22.8671297	4.126608978	1.104731	3.735398	0.000187	0.001214
HERC2P2	555.394675	-2.509649711	0.366183	-6.85354	7.20E-12	2.17E-10
AC138649.1	32.5428955	6.148349254	1.1164	5.507301	3.64E-08	6.12E-07
GOLGA6L2	12.1889464	4.326075881	1.257234	3.440947	0.00058	0.003092
MAGEL2	99.3330212	2.352831212	0.557536	4.220053	2.44E-05	0.000209
PWRN4	19.748252	3.537646295	1.101305	3.212231	0.001317	0.006085
PWRN1	85.9608079	5.092518661	0.834371	6.103422	1.04E-09	2.28E-08
NPAP1	16.6143602	3.886086759	1.140676	3.406829	0.000657	0.003443
LINC02250	17.9407699	4.991530811	1.211566	4.119901	3.79E-05	0.000307
LINC02346	31.8289859	4.273068593	0.967891	4.414823	1.01E-05	9.62E-05
GABRG3	34.6387267	3.226557755	0.871035	3.704278	0.000212	0.001341
GABRG3-AS1	9.22810221	3.999043102	1.274302	3.138222	0.0017	0.0075
AC144833.1	13.3135394	4.460011267	1.248997	3.570873	0.000356	0.002065
AC021979.3	15.187861	4.656333116	1.236755	3.764961	0.000167	0.001099
OCA2	182.13016	3.056913998	0.50684	6.031324	1.63E-09	3.48E-08
RPL41P2	116.835458	3.431466626	0.579596	5.920448	3.21E-09	6.54E-08
APBA2	996.219056	-2.043640973	0.338798	-6.03203	1.62E-09	3.47E-08
AC107980.1	13.8172623	4.603767001	1.236903	3.722012	0.000198	0.001268
CHRFAM7A	25.0936651	3.189969091	0.988131	3.228284	0.001245	0.00581
GOLGA8UP	11.2312825	4.357474461	1.250674	3.484102	0.000494	0.002704
HERC2P10	16.2658548	3.804728599	1.15643	3.290065	0.001002	0.00484
AC009562.1	201.859421	6.787439999	0.660142	10.28179	8.51E-25	8.85E-23
TRPM1	6245.00235	7.16961544	0.378669	18.9337	6.02E-80	9.27E-77
MIR211	11.4128589	4.32259367	1.254608	3.445374	0.00057	0.003052
OTUD7A	69.3974165	3.470761502	0.717895	4.834639	1.33E-06	1.59E-05
CHRNA7	116.608466	4.892132278	0.66323	7.376225	1.63E-13	6.15E-12
SCG5	3040.96642	2.636142072	0.42223	6.243385	4.28E-10	1.00E-08
GREM1	1139.0489	-2.522729498	0.383227	-6.58286	4.61E-11	1.27E-09
NUTM1	13.0031044	4.473281904	1.246584	3.588433	0.000333	0.001957
AC025678.3	51.9717009	-3.59198472	0.761273	-4.71839	2.38E-06	2.66E-05
AC018868.1	21.6868832	4.229803159	1.063571	3.976983	6.98E-05	0.00052
AC087516.2	18.0051992	5.030607371	1.207457	4.166283	3.10E-05	0.000256
AC068875.1	10.1594073	4.067721584	1.272091	3.197666	0.001385	0.006342
TMCO5A	19.2798225	3.350340568	1.103003	3.037472	0.002386	0.00987
LINC02694	27.2596603	5.712656679	1.157668	4.934624	8.03E-07	1.02E-05
AC109630.1	9.56489611	3.948402431	1.27948	3.085944	0.002029	0.008665

THBS1	2111.05313	-2.395986475	0.366936	-6.52971	6.59E-11	1.76E-09
FSIP1	51.2005525	2.503050051	0.727963	3.438431	0.000585	0.003116
BMF	1436.04106	2.963297085	0.332839	8.903084	5.43E-19	3.70E-17
AC021755.1	12.0600878	4.135111753	1.272919	3.248527	0.00116	0.005468
CCDC9B	3585.43368	-3.129718381	0.333957	-9.37163	7.14E-21	5.68E-19
CHST14	1176.00981	-2.592897031	0.369057	-7.02574	2.13E-12	6.91E-11
SPINT1	1115.42287	-4.391208299	0.351759	-12.4836	9.18E-36	1.82E-33
EXD1	17.9849589	3.967854596	1.139796	3.481196	0.000499	0.002729
LTK	16.6496537	3.629430666	1.149582	3.157175	0.001593	0.007121
SPTBN5	301.735498	2.843830617	0.415372	6.846469	7.57E-12	2.28E-10
PLA2G4E	18.8193572	5.123549075	1.199937	4.269849	1.96E-05	0.000172
PLA2G4D	24.369364	4.518759533	1.072421	4.213605	2.51E-05	0.000214
PLA2G4F	28.3465887	4.572274702	1.045692	4.372487	1.23E-05	0.000115
CAPN3	82.4113817	3.126425371	0.64935	4.814696	1.47E-06	1.75E-05
EPB42	11.0643534	4.176955156	1.266227	3.298742	0.000971	0.004726
TGM7	11.5417175	4.386168201	1.249182	3.511234	0.000446	0.002496
CKMT1B	10.9472063	4.290824649	1.255708	3.417056	0.000633	0.003334
PATL2	36.3028531	3.941383104	0.909691	4.332662	1.47E-05	0.000134
SORD2P	98.644324	2.835654512	0.60929	4.654029	3.26E-06	3.52E-05
DUOX2	38.0806102	3.836944162	0.97252	3.945365	7.97E-05	0.000583
GATM	113.6561	2.143443568	0.539139	3.975678	7.02E-05	0.000522
SEMA6D	532.178145	4.614920677	0.397059	11.62276	3.16E-31	4.86E-29
AC092078.3	12.2533757	4.381831723	1.252369	3.498834	0.000467	0.002588
SLC24A5	206.684803	4.875128445	0.526284	9.2633	1.98E-20	1.52E-18
SLC12A1	46.588859	5.484441471	0.992472	5.52604	3.28E-08	5.53E-07
FGF7	71.8264384	2.437005343	0.640652	3.803943	0.000142	0.000961
ATP8B4	43.9271441	4.769450412	0.922099	5.172382	2.31E-07	3.32E-06
HDC	10.5987009	4.173911508	1.264765	3.300148	0.000966	0.004718
CYP19A1	27.3358012	5.821064288	1.143823	5.089132	3.60E-07	4.96E-06
GLDN	20.2016674	5.310072643	1.183103	4.48826	7.18E-06	7.10E-05
DMXL2	372.374445	2.081723753	0.397786	5.233271	1.67E-07	2.45E-06
TMOD3	1257.17473	-2.029154804	0.329165	-6.16454	7.07E-10	1.61E-08
AC009754.2	9.69375473	4.046019533	1.27217	3.180407	0.001471	0.006648
ONECUT1	26.6534376	5.476239171	1.184578	4.622944	3.78E-06	4.02E-05
WDR72	37.3587523	3.853573408	0.891394	4.323084	1.54E-05	0.000139
AC084759.3	10.0949779	4.005107431	1.276891	3.136609	0.001709	0.007522
AC084759.2	10.4698423	4.061968818	1.273658	3.189215	0.001427	0.006475
UNC13C	47.2709756	6.217188542	1.142965	5.439528	5.34E-08	8.70E-07
RAB27A	1250.76349	2.549184083	0.367485	6.936834	4.01E-12	1.26E-10
DNAAF4	56.0035905	3.72168291	0.765814	4.859771	1.18E-06	1.42E-05
PRTG	319.935708	2.404637642	0.403856	5.9542	2.61E-09	5.42E-08
MNS1	169.937492	2.34955026	0.502828	4.672676	2.97E-06	3.25E-05
AC090518.1	11.8785114	4.334365593	1.255325	3.452784	0.000555	0.002989
GCOM1	20.5887688	3.570776002	1.105904	3.228829	0.001243	0.0058

ALDH1A2	44.2450348	3.344887313	0.844114	3.9626	7.41E-05	0.000548
LIPC	19.4138683	5.181459929	1.195445	4.334337	1.46E-05	0.000133
MYO1E	2142.153	-2.572046267	0.334123	-7.6979	1.38E-14	5.82E-13
FAM81A	43.4025686	3.695911148	0.806526	4.582505	4.59E-06	4.78E-05
AC092079.1	10.9354948	3.987525477	1.280888	3.113095	0.001851	0.008019
AC037433.1	9.44774903	4.046930126	1.271214	3.183517	0.001455	0.006587
ANXA2	66561.7608	-3.364205792	0.395663	-8.5027	1.85E-17	1.07E-15
AC126323.2	9.25446109	3.938692024	1.279145	3.07916	0.002076	0.008797
GOLGA2P11	16.2480247	4.685379322	1.237493	3.786186	0.000153	0.001021
TPM1	48222.362	-4.626345719	0.314874	-14.6927	7.18E-49	3.24E-46
TPM1-AS	30.413779	-3.648043517	0.982285	-3.71384	0.000204	0.001302
RPS27L	4229.08786	-2.189149098	0.367549	-5.95608	2.58E-09	5.37E-08
CA12	458.003114	-3.396124281	0.431621	-7.86831	3.59E-15	1.62E-13
AC090543.2	41.0912132	3.081023406	0.838042	3.676457	0.000236	0.00147
AC103691.1	12.1889464	4.326075881	1.257234	3.440947	0.00058	0.003092
KBTBD13	13.5331862	4.511043366	1.24495	3.623474	0.000291	0.001749
IGDCC4	164.514387	4.514879587	0.530955	8.503325	1.84E-17	1.07E-15
MEGF11	68.3864787	3.140692617	0.67151	4.67706	2.91E-06	3.19E-05
LCTL	140.622161	-2.092736591	0.51625	-4.05373	5.04E-05	0.000392
SMAD3	4732.23192	-2.093618226	0.314006	-6.66745	2.60E-11	7.39E-10
ITGA11	521.391424	-2.820522121	0.541369	-5.20998	1.89E-07	2.75E-06
AC027088.5	10.0949779	4.005107431	1.276891	3.136609	0.001709	0.007522
AC026992.2	24.4657452	5.681160601	1.152226	4.930594	8.20E-07	1.04E-05
PAQR5	231.728642	3.121724202	0.454206	6.872926	6.29E-12	1.92E-10
AC027237.3	135.980025	-2.175954432	0.519353	-4.18974	2.79E-05	0.000234
DRAIC	99.9021474	4.588167981	0.760801	6.03071	1.63E-09	3.49E-08
AC021818.1	10.405413	3.972768242	1.280392	3.102774	0.001917	0.00826
LARP6	4090.62836	-3.210377119	0.362066	-8.86683	7.53E-19	4.99E-17
LRRC49	398.305513	2.088561835	0.401773	5.198369	2.01E-07	2.91E-06
THSD4-AS1	18.2248461	5.063132891	1.20461	4.20313	2.63E-05	0.000223
HMGB1P6	905.339255	2.494733014	0.360576	6.918751	4.56E-12	1.41E-10
PKM	79765.2664	-3.570953891	0.364218	-9.80444	1.08E-22	9.95E-21
ADPGK-AS1	9.75818404	4.081877292	1.269445	3.215482	0.001302	0.006028
PML	2085.58963	-2.519845131	0.318785	-7.90453	2.69E-15	1.24E-13
GOLGA6A	10.3790541	4.122386704	1.268347	3.250204	0.001153	0.005447
ISLR2	18.5352811	5.056349239	1.206527	4.190831	2.78E-05	0.000233
AC010931.1	10.5079127	4.202889647	1.26189	3.330629	0.000866	0.004313
CCDC33	102.164447	3.595910971	0.612857	5.867458	4.43E-09	8.76E-08
AC023300.3	12.4730225	4.428932897	1.248782	3.546601	0.00039	0.002237
PPIAP46	10.0686191	4.107514473	1.268446	3.238227	0.001203	0.005627
SEMA7A	882.86287	-5.593104427	0.426043	-13.128	2.27E-39	5.84E-37
CYP1A1	55.1331262	4.155361959	0.763095	5.445405	5.17E-08	8.45E-07
AC091230.1	13.8787558	4.742935546	1.223378	3.876917	0.000106	0.000745
FAM219B	353.335884	-2.004896821	0.403761	-4.96556	6.85E-07	8.83E-06

RPP25	842.399313	2.041570437	0.409556	4.984841	6.20E-07	8.08E-06
SNX33	1259.28003	-2.239469573	0.331443	-6.75673	1.41E-11	4.15E-10
CSPG4	842.026102	-3.061384433	0.355703	-8.60658	7.53E-18	4.53E-16
LINGO1	28.9420312	3.308703855	0.969811	3.4117	0.000646	0.003395
LINGO1-AS1	11.9429407	4.379058258	1.251429	3.499245	0.000467	0.002588
CSPG4P13	15.3430785	4.639636316	1.238938	3.74485	0.000181	0.001174
TBC1D2B	2305.02735	-2.058890671	0.393808	-5.22815	1.71E-07	2.51E-06
ACSBG1	15.8735662	3.605570759	1.143834	3.152181	0.001621	0.007218
CHRNA5	209.749993	2.337598722	0.463119	5.047509	4.48E-07	6.06E-06
CHRNA3	4416.69049	3.445110201	0.342321	10.06399	7.97E-24	7.81E-22
CHRNB4	155.687717	3.905413304	0.555645	7.028614	2.09E-12	6.78E-11
AC067863.1	16.6580236	5.076719299	1.197226	4.240401	2.23E-05	0.000193
AC022748.2	11.2576413	4.313999964	1.254792	3.438019	0.000586	0.003119
CTSH	15706.7166	2.986432643	0.381302	7.832199	4.79E-15	2.11E-13
ANKRD34C-AS1	31.2246174	3.637733272	0.973829	3.735496	0.000187	0.001213
ANKRD34C	19.1622696	3.822718059	1.188963	3.21517	0.001304	0.006033
LINC00927	17.126612	4.883287581	1.220343	4.00157	6.29E-05	0.000476
AC016705.2	239.431087	2.317349128	0.438507	5.28464	1.26E-07	1.90E-06
AC016705.1	9.13731401	4.003118936	1.27369	3.142931	0.001673	0.007426
CFAP161	11.8785114	4.334365593	1.255325	3.452784	0.000555	0.002989
IL16	56.1922314	5.180915834	0.874942	5.921438	3.19E-09	6.51E-08
STARD5	104.442309	-3.031274872	0.579171	-5.23382	1.66E-07	2.45E-06
AC104041.1	34.0777661	4.941462814	1.053295	4.691434	2.71E-06	2.99E-05
MEX3B	313.952645	-2.071647205	0.409885	-5.05422	4.32E-07	5.86E-06
AC243919.1	2318.72828	-2.689522352	0.318283	-8.4501	2.91E-17	1.65E-15
CPEB1	119.737756	2.351728388	0.54568	4.309721	1.63E-05	0.000147
AP3B2	50.7062481	6.650184298	1.093279	6.082788	1.18E-09	2.58E-08
FSD2	16.362236	5.012156132	1.20314	4.165897	3.10E-05	0.000256
HOMER2	2787.27104	3.811383028	0.329271	11.57522	5.50E-31	8.18E-29
TM6SF1	11.0379945	4.269395787	1.258002	3.393791	0.000689	0.003567
BNC1	2177.45136	-2.274578684	0.333496	-6.82042	9.08E-12	2.71E-10
SLC28A1	16.5057419	4.903137571	1.215891	4.032546	5.52E-05	0.000423
LINC01584	11.3865	4.372633274	1.249852	3.498521	0.000468	0.00259
AGBL1	39.196491	3.852771318	0.958468	4.019717	5.83E-05	0.000445
NTRK3	113.60345	3.786506091	0.652713	5.801179	6.59E-09	1.27E-07
ISG20	343.876779	4.082569456	0.451107	9.050105	1.43E-19	1.02E-17
ACAN	45.0335012	6.352953923	1.121365	5.665377	1.47E-08	2.65E-07
HAPLN3	146.413205	-2.026255417	0.495618	-4.08834	4.34E-05	0.000345
ABHD2	3071.38364	2.413798583	0.341906	7.059825	1.67E-12	5.50E-11
RLBP1	934.229665	4.766156175	0.41009	11.62222	3.18E-31	4.87E-29
MIR9-3HG	30.0125692	5.879593224	1.144761	5.136087	2.81E-07	3.94E-06
AC124248.1	9.53853722	4.032853085	1.27268	3.168788	0.001531	0.006875
FURIN	3837.63022	-2.901997561	0.312967	-9.27252	1.82E-20	1.40E-18
SV2B	464.413016	8.95893624	0.752636	11.90342	1.14E-32	1.92E-30

LINC00930	14.8627787	4.859395158	1.214185	4.002186	6.28E-05	0.000475
AC091544.2	9.62932542	4.002668209	1.275446	3.138251	0.0017	0.0075
HMGN1P38	154.805842	-2.968178343	0.538024	-5.51682	3.45E-08	5.81E-07
RGMA	318.101272	2.375011128	0.404194	5.875915	4.21E-09	8.36E-08
AC091078.1	33.7875714	5.987317566	1.141712	5.244159	1.57E-07	2.32E-06
LINC01579	32.6828851	4.052905377	0.949011	4.270662	1.95E-05	0.000172
LINC02207	26.9260492	4.772020194	1.027198	4.645669	3.39E-06	3.64E-05
LINC01580	12.1625875	4.422982886	1.248134	3.543676	0.000395	0.002253
LINC01581	11.4392177	4.228541376	1.263141	3.347639	0.000815	0.004096
AC009432.2	9.38331971	4.017150993	1.273395	3.154678	0.001607	0.00717
AC087633.2	105.450336	2.350934961	0.566874	4.147191	3.37E-05	0.000275
LINC01197	36.2300453	6.135497008	1.128847	5.435189	5.47E-08	8.91E-07
LINC00924	13.9080505	4.552167927	1.242358	3.664136	0.000248	0.001529
AC015574.1	25.4648211	4.344679234	1.07676	4.034957	5.46E-05	0.000421
AC024337.1	11.5417175	4.386168201	1.249182	3.511234	0.000446	0.002496
LINC02253	9.25446109	3.938692024	1.279145	3.07916	0.002076	0.008797
LINC02254	24.8171864	5.512964664	1.174677	4.693176	2.69E-06	2.97E-05
AC026523.2	20.5384613	5.262181298	1.190099	4.421634	9.80E-06	9.35E-05
LINC00923	53.4021834	4.05333989	0.844279	4.800947	1.58E-06	1.85E-05
LUNAR1	19.0863757	3.585101342	1.070477	3.349068	0.000811	0.004086
AC015660.1	33.8043675	3.021056557	0.902662	3.34683	0.000817	0.004104
DNM1P46	24.7526068	3.087650043	0.956668	3.227503	0.001249	0.005819
AC022710.1	9.62932542	4.002668209	1.275446	3.138251	0.0017	0.0075
CERS3	17.8739303	3.387644371	1.116322	3.034649	0.002408	0.009949
AC015712.1	34.4451687	2.603961974	0.825771	3.153369	0.001614	0.0072
AC015712.6	63.841004	4.711959144	0.79372	5.936551	2.91E-09	5.99E-08
PCSK6	63.1875464	3.199349365	0.764406	4.185407	2.85E-05	0.000238
LINC02348	15.5805554	3.759291709	1.157359	3.248163	0.001162	0.005473
DNM1P47	31.612116	4.369116398	0.98173	4.450424	8.57E-06	8.31E-05
RHBDF1	1329.04324	-2.016166065	0.354327	-5.69013	1.27E-08	2.32E-07
HBQ1	82.3444579	-2.068863366	0.596115	-3.47058	0.000519	0.002824
RGS11	435.472059	-3.190343027	0.389643	-8.18785	2.66E-16	1.38E-14
ARHGDIG	218.465348	-4.479122637	0.536952	-8.34176	7.32E-17	4.01E-15
PDIA2	169.235674	-3.504442195	0.511116	-6.85645	7.06E-12	2.14E-10
Z97634.1	119.907754	3.407183508	0.565109	6.029246	1.65E-09	3.52E-08
NME4	5873.91773	-2.567337636	0.358245	-7.16643	7.70E-13	2.65E-11
DECR2	755.031838	-2.301927588	0.377656	-6.0953	1.09E-09	2.40E-08
MSLN	138.178428	-2.086167423	0.596294	-3.49856	0.000468	0.00259
CEROX1	2758.49365	4.717034637	0.347129	13.58871	4.67E-42	1.40E-39
SOX8	3911.49758	4.714971918	0.317413	14.85439	6.52E-50	3.25E-47
SSTR5-AS1	10.4698423	4.061968818	1.273658	3.189215	0.001427	0.006475
PRSS29P	9.09924358	3.9280641	1.279421	3.07019	0.002139	0.009014
CCDC154	96.2447411	3.019669438	0.599927	5.033395	4.82E-07	6.46E-06
PTX4	12.5901696	4.10169093	1.276939	3.212128	0.001318	0.006086

HAGH	1576.33584	-2.099311198	0.342485	-6.12965	8.81E-10	1.96E-08
AL031722.1	20.2035852	3.892800243	1.060472	3.67082	0.000242	0.001496
ZNF598	1054.0795	-2.20593261	0.353942	-6.23248	4.59E-10	1.07E-08
NPW	1081.01636	3.432995403	0.473225	7.254465	4.03E-13	1.45E-11
SLC9A3R2	1192.49315	-2.455646339	0.37105	-6.6181	3.64E-11	1.01E-09
PRSS33	7795.40159	7.762294091	0.4243	18.29436	9.18E-75	1.24E-71
PRSS21	20.2551968	3.358573094	1.069974	3.138929	0.001696	0.0075
PRSS22	18.4593872	4.144229077	1.100536	3.765645	0.000166	0.001098
PAQR4	392.275765	2.617256933	0.395042	6.625257	3.47E-11	9.68E-10
LINC00514	28.1076329	3.023651172	0.970542	3.115425	0.001837	0.007974
CLDN6	43.1705636	-2.648480404	0.866329	-3.05713	0.002235	0.009345
TNFRSF12A	4663.91291	-5.37117979	0.340794	-15.7608	5.79E-56	3.77E-53
AC108134.3	9.47410791	3.993729441	1.275601	3.130862	0.001743	0.007649
SRL	28.1594193	3.9959209	1.022071	3.909632	9.24E-05	0.000663
GLIS2	2164.34998	-2.276166524	0.318174	-7.15384	8.44E-13	2.90E-11
CDIP1	2093.67542	-2.391436585	0.377052	-6.34247	2.26E-10	5.54E-09
UBALD1	1390.2182	-4.193105137	0.395014	-10.6151	2.54E-26	2.89E-24
MGRN1	3425.79048	-2.949076906	0.344181	-8.56839	1.05E-17	6.21E-16
PPL	3169.13362	-3.239241614	0.322764	-10.036	1.06E-23	1.03E-21
RBFOX1	53.1633693	6.632475099	1.100829	6.024984	1.69E-09	3.61E-08
RPL21P119	12.8830215	4.561980136	1.23838	3.683829	0.00023	0.001437
LINC02177	17.2554706	4.968540486	1.211556	4.100957	4.11E-05	0.000329
AC012178.1	22.1667773	5.415994766	1.177458	4.599734	4.23E-06	4.44E-05
AC099489.1	115.452972	4.161130448	0.706917	5.886305	3.95E-09	7.92E-08
COX6CP1	46.8377468	2.987444371	0.773286	3.863311	0.000112	0.000781
SHISA9	28.7940775	3.416962585	0.960014	3.559283	0.000372	0.002148
LINC02185	14.7602789	4.784725864	1.221827	3.916041	9.00E-05	0.000647
MYH11	79.2820403	4.097296306	0.681411	6.012959	1.82E-09	3.87E-08
NOMO3	203.132626	-2.233234122	0.467441	-4.77757	1.77E-06	2.05E-05
TMC5	109.394128	3.247912947	0.571464	5.683493	1.32E-08	2.40E-07
GPR139	10.4698423	4.061968818	1.273658	3.189215	0.001427	0.006475
GP2	14.0369091	4.644853865	1.233596	3.765297	0.000166	0.001098
UMOD	13.1583219	4.468559565	1.247619	3.58167	0.000341	0.002
ACSM5	15.691584	4.795258917	1.224373	3.916503	8.98E-05	0.000647
ACSM2A	20.6292495	5.225652702	1.194707	4.374004	1.22E-05	0.000114
ACSM2B	27.7633833	5.774508055	1.151339	5.015472	5.29E-07	7.03E-06
REXO5	148.523641	2.106459102	0.528866	3.982973	6.81E-05	0.000508
DNAH3	64.9327063	5.143038318	0.913261	5.631511	1.79E-08	3.18E-07
ZP2	13.0031044	4.473281904	1.246584	3.588433	0.000333	0.001957
ANKS4B	12.1625875	4.422982886	1.248134	3.543676	0.000395	0.002253
OTOA	21.7538425	5.30038449	1.189889	4.454518	8.41E-06	8.17E-05
AC130466.1	12.3441639	4.315094906	1.258772	3.428019	0.000608	0.003216
SCNN1G	13.7791919	4.396401629	1.256379	3.499265	0.000467	0.002588
SCNN1B	18.2892754	5.094848856	1.201233	4.241351	2.22E-05	0.000192

PRKCB	585.686834	2.453185842	0.364347	6.733104	1.66E-11	4.81E-10
AC008938.1	13.2491101	4.384912975	1.255665	3.492104	0.000479	0.002637
SLC5A11	21.1879502	3.418990383	1.028606	3.323907	0.000888	0.004402
IL4R	290.367448	-2.558885476	0.432196	-5.92067	3.21E-09	6.53E-08
GSG1L	18.9745747	5.12148459	1.200752	4.26523	2.00E-05	0.000175
EIF3CL	407.338216	2.582126897	0.422491	6.11168	9.86E-10	2.18E-08
SULT1A1	856.38128	2.308087044	0.444952	5.18727	2.13E-07	3.08E-06
RABEP2	1301.55613	-2.229744324	0.371771	-5.99763	2.00E-09	4.22E-08
MVP	9314.63114	-2.463776302	0.34735	-7.09307	1.31E-12	4.38E-11
ALDOA	673.464708	-2.013225712	0.386635	-5.20705	1.92E-07	2.80E-06
SULT1A3	106.460628	3.678621077	0.606619	6.064141	1.33E-09	2.87E-08
ITGAL	48.2870225	4.161413911	0.911614	4.564885	5.00E-06	5.14E-05
FBRS	2203.69685	-2.210663121	0.348736	-6.33908	2.31E-10	5.66E-09
FBXL19	1971.66658	-2.560708692	0.320479	-7.99026	1.35E-15	6.53E-14
BCKDK	1793.40509	-2.092786184	0.357594	-5.8524	4.85E-09	9.52E-08
PYCARD	120.997029	-2.269325463	0.535628	-4.23676	2.27E-05	0.000196
TRIM72	40.6671706	5.360032616	0.983077	5.452302	4.97E-08	8.14E-07
ITGAM	27.8222196	4.555529864	1.101432	4.136007	3.53E-05	0.000288
ITGAD	17.0155834	3.87277951	1.1503	3.366757	0.000761	0.003881
TGFB1I1	2239.20568	-4.319988614	0.336233	-12.8482	8.81E-38	1.98E-35
AC136428.4	10.6894891	4.123736642	1.269366	3.248658	0.00116	0.005468
AC023824.6	10.9472063	4.290824649	1.255708	3.417056	0.000633	0.003334
FRG2DP	10.7802773	4.027023489	1.277437	3.152423	0.001619	0.007214
KIF18BP1	13.442398	4.562290166	1.239586	3.680494	0.000233	0.001452
ANKRD26P1	26.0249701	3.203106331	1.006876	3.181233	0.001466	0.006635
AC092368.3	52.9076016	-2.669353117	0.838471	-3.18359	0.001455	0.006587
MYLK3	59.5319342	4.2659621	0.75389	5.6586	1.53E-08	2.75E-07
LINC02133	10.8564181	4.301121925	1.254435	3.428732	0.000606	0.003213
ABCC12	29.4180581	5.844023343	1.147565	5.092542	3.53E-07	4.88E-06
ABCC11	41.1586259	3.87436631	0.924291	4.191718	2.77E-05	0.000232
AC007611.1	44.7343574	3.29321421	0.767772	4.289311	1.79E-05	0.000159
AC044798.3	12.7834575	4.424631592	1.250368	3.538662	0.000402	0.002286
AC007614.1	57.570953	6.751958784	1.092983	6.177552	6.51E-10	1.48E-08
AC007614.2	9.78454293	4.008050513	1.275572	3.14216	0.001677	0.007433
LINC02179	12.5638107	4.372264827	1.254408	3.485521	0.000491	0.00269
ZNF423	49.0098777	2.362648874	0.766302	3.083181	0.002048	0.008729
AC007493.1	11.2576413	4.313999964	1.254792	3.438019	0.000586	0.003119
NKD1	39.0741482	5.119409546	0.990217	5.169987	2.34E-07	3.35E-06
AC007608.4	9.84897224	4.056445929	1.271884	3.189321	0.001426	0.006475
LINC02128	19.7623738	5.260421909	1.187416	4.430141	9.42E-06	9.04E-05
SALL1	13.1963923	4.589335614	1.235892	3.713379	0.000205	0.001303
AC087564.1	15.2522903	4.713246257	1.231253	3.828008	0.000129	0.000883
C16orf97	12.0337289	4.332064733	1.25612	3.448766	0.000563	0.003025
CASC22	10.0305486	3.916288499	1.283419	3.051449	0.002277	0.0095

TOX3	37.4254331	4.691068113	0.927006	5.06045	4.18E-07	5.68E-06
AC026462.4	11.7496528	4.203681987	1.266356	3.31951	0.000902	0.004457
AC007342.3	13.2227512	4.519365893	1.242964	3.635958	0.000277	0.001678
LINC02183	14.4762028	4.720153283	1.227539	3.845216	0.00012	0.000832
IRX6	13.2227512	4.519365893	1.242964	3.635958	0.000277	0.001678
MMP2	6525.3478	-3.313343792	0.320942	-10.3238	5.50E-25	5.84E-23
SLC6A2	20.9575146	4.241883794	1.107264	3.830959	0.000128	0.000875
CES1P1	15.7823722	4.739937488	1.230461	3.852164	0.000117	0.000815
CES5A	19.4402272	5.106787881	1.204105	4.241147	2.22E-05	0.000192
AC040168.1	26.9111549	5.636921505	1.166097	4.834007	1.34E-06	1.60E-05
MT1F	442.743957	5.88163157	0.54479	10.79614	3.59E-27	4.33E-25
MT1G	99.9246871	7.707298592	1.022215	7.539799	4.71E-14	1.89E-12
MT1H	11.1639174	4.231154291	1.262941	3.35024	0.000807	0.00407
MT1X	150.142148	3.945557053	0.595516	6.625444	3.46E-11	9.68E-10
CX3CL1	27.8629472	5.926009604	1.131862	5.235628	1.64E-07	2.43E-06
ADGRG1	317.051688	-2.369273588	0.504322	-4.69794	2.63E-06	2.91E-05
KIFC3	2254.36898	-3.021707886	0.323296	-9.34658	9.05E-21	7.09E-19
CNGB1	30.425504	5.950249265	1.136693	5.234701	1.65E-07	2.44E-06
USB1	1267.24332	-2.667981335	0.336977	-7.91741	2.43E-15	1.12E-13
MMP15	7834.83763	-3.473558136	0.3175	-10.9404	7.39E-28	9.42E-26
NDRG4	2861.70846	-3.441876882	0.318761	-10.7977	3.53E-27	4.29E-25
LINC02141	19.5573743	4.839096753	1.232386	3.926608	8.62E-05	0.000625
AC009081.2	12.0600878	4.135111753	1.272919	3.248527	0.00116	0.005468
LINC02165	13.1846808	4.268440808	1.265487	3.372963	0.000744	0.003804
CDH11	4083.31077	-3.820349451	0.332595	-11.4865	1.54E-30	2.27E-28
CDH5	19.9660447	4.589758378	1.142233	4.018234	5.86E-05	0.000447
CDH16	18.9101454	5.085147585	1.204649	4.221268	2.43E-05	0.000208
CBFB	763.464608	-2.070639823	0.353656	-5.85495	4.77E-09	9.39E-08
HSF4	406.248197	2.86365332	0.524406	5.460752	4.74E-08	7.80E-07
KCTD19	29.1785767	4.171602858	0.966953	4.314174	1.60E-05	0.000145
TPPP3	94.7381533	3.284303413	0.671543	4.890684	1.00E-06	1.24E-05
RIPOR1	2484.79658	-2.448857202	0.319227	-7.67122	1.70E-14	7.13E-13
EDC4	1228.55039	-2.121449107	0.435934	-4.86644	1.14E-06	1.38E-05
LCAT	435.817036	-2.290337451	0.409162	-5.59764	2.17E-08	3.80E-07
SLC12A4	3667.07215	-2.240522202	0.314998	-7.11282	1.14E-12	3.85E-11
DPEP2	10.6894891	4.123736642	1.269366	3.248658	0.00116	0.005468
PLA2G15	1357.58758	-2.905265004	0.333003	-8.72444	2.67E-18	1.69E-16
SMPD3	1699.68404	2.885127681	0.335979	8.587216	8.91E-18	5.30E-16
AC099521.2	35.5639132	3.466602837	0.879334	3.942304	8.07E-05	0.000589
CDH1	340.959317	2.307636965	0.402858	5.728168	1.02E-08	1.89E-07
NQO1	3190.13312	-2.267790141	0.372353	-6.09043	1.13E-09	2.46E-08
IL34	44.5629745	2.5513388	0.832837	3.06343	0.002188	0.009183
HYDIN	121.491888	2.94570778	0.640868	4.596436	4.30E-06	4.50E-05
CALB2	88.5737934	-3.281450426	0.657313	-4.99222	5.97E-07	7.81E-06

TAT	18.5352811	5.056349239	1.206527	4.190831	2.78E-05	0.000233
LINC01572	39.1319114	3.008451986	0.850818	3.535952	0.000406	0.002304
LINC01568	26.9228664	5.755957005	1.150977	5.000931	5.71E-07	7.51E-06
AC092114.1	10.2501954	3.994010754	1.278276	3.124529	0.001781	0.007777
AC138627.1	28.2327441	3.700605471	1.02282	3.618041	0.000297	0.001781
CHST6	40.8066371	2.575441698	0.820596	3.1385	0.001698	0.0075
SYCE1L	1158.36854	2.13029596	0.350496	6.077944	1.22E-09	2.65E-08
VAT1L	1715.19823	5.923108307	0.369233	16.04165	6.54E-58	4.62E-55
LINC01229	31.8354711	3.353582496	0.968413	3.462966	0.000534	0.002896
ARLNC1	16.7404419	3.594617491	1.1594	3.100412	0.001933	0.008316
PKD1L2	188.646411	2.155565949	0.455952	4.727611	2.27E-06	2.55E-05
BCO1	460.684209	5.263676652	1.206034	4.364451	1.27E-05	0.000118
AC131888.1	29.96597	4.847345233	1.03962	4.662614	3.12E-06	3.40E-05
SDR42E1	33.156327	3.48225682	0.96879	3.594439	0.000325	0.001927
CDH13	221.563964	-2.256752283	0.588214	-3.83662	0.000125	0.000857
AC099506.1	15.5100076	4.860577653	1.216627	3.995124	6.47E-05	0.000485
AC009063.2	13.3516098	4.59562907	1.235878	3.718512	0.0002	0.001281
SLC38A8	10.7539184	4.178531017	1.264949	3.303319	0.000955	0.004683
AC040169.1	109.706738	2.260700311	0.540267	4.184414	2.86E-05	0.000239
ADAD2	89.4607164	7.606479608	1.022863	7.436457	1.03E-13	4.01E-12
KCNG4	20.8752552	5.165395553	1.202415	4.29585	1.74E-05	0.000155
WFDC1	83.7355304	6.550836215	0.860764	7.610492	2.73E-14	1.12E-12
MEAK7	1222.66146	-3.122074109	0.344761	-9.05576	1.36E-19	9.72E-18
COTL1	32349.563	-3.261868901	0.339974	-9.59446	8.44E-22	7.24E-20
CRISPLD2	854.545143	-3.927830784	0.412677	-9.51792	1.77E-21	1.47E-19
ZDHHC7	3095.01663	-2.4870253	0.315424	-7.8847	3.15E-15	1.43E-13
CIBAR2	12.0981582	4.381361397	1.251818	3.499999	0.000465	0.002588
AC026469.1	11.1287827	4.232433809	1.261651	3.354678	0.000795	0.004025
AC092127.1	8.94402608	3.914238868	1.279942	3.058139	0.002227	0.009318
AC135012.1	27.7897422	5.7282997	1.157365	4.949433	7.44E-07	9.50E-06
LINC00917	14.1921267	4.645323689	1.234155	3.76397	0.000167	0.001102
FENDRR	14.4762028	4.720153283	1.227539	3.845216	0.00012	0.000832
FOXF1	15.1234317	4.58060031	1.243877	3.682518	0.000231	0.001442
FOXC2	748.789374	-5.738581472	0.40464	-14.1819	1.19E-45	4.32E-43
FOXL1	254.327356	-2.379403809	0.488482	-4.87102	1.11E-06	1.35E-05
AC093519.2	14.1276973	4.598912982	1.238579	3.713055	0.000205	0.001303
MAP1LC3B	3298.75005	-2.905059151	0.356323	-8.15289	3.55E-16	1.83E-14
AC092720.1	15.9375897	4.727108273	1.232314	3.835959	0.000125	0.000859
ZNF469	403.455964	-2.342787685	0.566637	-4.13455	3.56E-05	0.00029
PABPN1L	11.1668532	4.332430351	1.252741	3.45836	0.000543	0.00294
CBFA2T3	15.8472073	3.636652484	1.135687	3.20216	0.001364	0.006265
AC092384.3	11.0643534	4.176955156	1.266227	3.298742	0.000971	0.004726
CDH15	31.7588133	2.809880684	0.893083	3.14627	0.001654	0.007349
CPNE7	1975.504	-5.023934102	0.335465	-14.976	1.05E-50	5.49E-48

VPS9D1-AS1	139.293378	-2.422638707	0.512676	-4.72548	2.30E-06	2.57E-05
DBNDD1	1367.26392	-2.839705815	0.334848	-8.48057	2.24E-17	1.29E-15
PRDM7	15.6271546	4.749160485	1.228948	3.864411	0.000111	0.000778
DOC2B	217.640705	4.629246302	0.486415	9.517065	1.78E-21	1.48E-19
TRARG1	9.93976043	4.008663455	1.276073	3.141406	0.001681	0.007444
MYO1C	7687.48143	-3.50599634	0.320418	-10.942	7.26E-28	9.29E-26
SERPINF2	749.864885	3.993822726	1.294209	3.085917	0.002029	0.008665
SERPINF1	55533.078	5.755149657	0.367545	15.65836	2.91E-55	1.83E-52
AC005696.4	14.5406321	4.751032377	1.224537	3.879859	0.000105	0.000737
OR1D2	20.9396845	5.211849478	1.197373	4.352736	1.34E-05	0.000124
OR1A1	12.4085932	4.379167019	1.253201	3.494385	0.000475	0.002618
OR3A3	8.78880857	3.89750018	1.280684	3.043295	0.00234	0.009716
ASPA	17.2671821	5.089799908	1.197821	4.249216	2.15E-05	0.000187
P2RX1	17.2554706	4.968540486	1.211556	4.100957	4.11E-05	0.000329
SPNS3	238.260726	2.616842036	0.440918	5.934988	2.94E-09	6.04E-08
MYBBP1A	1989.3652	-2.131530524	0.319829	-6.6646	2.65E-11	7.53E-10
ALOX15	9.11095513	4.012369049	1.272984	3.15194	0.001622	0.007223
AC091153.1	28.6006943	3.434201793	0.982331	3.495974	0.000472	0.002613
PLD2	643.652292	-2.664119334	0.360149	-7.39727	1.39E-13	5.31E-12
MINK1	3571.91184	-2.431602704	0.317326	-7.66279	1.82E-14	7.59E-13
CAMTA2	898.84647	-2.20605331	0.341124	-6.46702	1.00E-10	2.58E-09
KIF1C	9452.45885	-2.183218229	0.318467	-6.85541	7.11E-12	2.15E-10
AC055839.2	1526.13325	-2.114721218	0.331878	-6.37198	1.87E-10	4.64E-09
WSCD1	960.239693	2.489199485	0.344278	7.230195	4.82E-13	1.72E-11
AIPL1	16.3768833	4.818032405	1.224528	3.934605	8.33E-05	0.000606
PITPNM3	234.487053	-2.244814288	0.521295	-4.30623	1.66E-05	0.000149
SLC13A5	16.0664483	4.83022072	1.222086	3.95244	7.74E-05	0.000568
XAF1	12.4993814	4.297396672	1.260864	3.408295	0.000654	0.003427
TEKT1	14.9682142	4.603296853	1.241181	3.708802	0.000208	0.001321
ALOX12P2	21.5195483	5.444529837	1.171548	4.647295	3.36E-06	3.62E-05
BCL6B	15.1149029	3.741440246	1.154394	3.241043	0.001191	0.005585
ASGR2	10.2501954	3.994010754	1.278276	3.124529	0.001781	0.007777
SLC2A4	104.960508	5.39453637	0.691917	7.796504	6.36E-15	2.78E-13
EIF5A	13710.5796	-2.270759259	0.38095	-5.96077	2.51E-09	5.23E-08
KCTD11	554.271606	-2.221137985	0.365687	-6.07387	1.25E-09	2.71E-08
TMEM256	1413.60493	2.97195863	0.3347	8.879458	6.72E-19	4.49E-17
NLGN2	3900.87226	-2.451699056	0.322736	-7.5966	3.04E-14	1.24E-12
FGF11	28.9539102	-3.241244029	0.90411	-3.58501	0.000337	0.001978
AC113189.3	27.0353996	-4.483367846	1.029337	-4.35559	1.33E-05	0.000123
POLR2A	4131.56047	-2.141165883	0.312299	-6.85614	7.07E-12	2.14E-10
TNFSF12	79.191449	-2.931619806	0.624732	-4.69261	2.70E-06	2.97E-05
ATP1B2	70.5155402	4.690855998	0.726678	6.455202	1.08E-10	2.78E-09
TP53	2675.90089	-2.959688744	0.367886	-8.04513	8.62E-16	4.26E-14
DNAH2	81.5527793	3.676358451	0.737506	4.984852	6.20E-07	8.08E-06

CHD3	5249.8429	-2.022003129	0.307226	-6.58149	4.66E-11	1.28E-09
KCNAB3	116.170458	2.494636439	0.562927	4.431549	9.36E-06	9.00E-05
GUCY2D	16.2174014	4.282533342	1.166722	3.67057	0.000242	0.001497
ALOX15B	13.0675337	4.51829105	1.242467	3.636549	0.000276	0.001677
RANGRF	74.3329127	2.279224228	0.671517	3.39414	0.000688	0.003566
ARHGEF15	23.0072941	5.434744384	1.178136	4.613002	3.97E-06	4.19E-05
NDEL1	631.122277	-2.014454747	0.356254	-5.65454	1.56E-08	2.81E-07
PIK3R6	19.0187637	4.079122886	1.125003	3.625878	0.000288	0.001736
PIK3R5	25.2564801	5.563669993	1.169882	4.755752	1.98E-06	2.25E-05
AC005695.1	16.1953069	4.902893104	1.214708	4.036273	5.43E-05	0.000419
MYH8	71.971015	7.009784012	1.081915	6.479051	9.23E-11	2.40E-09
MYH4	30.1033574	5.856080782	1.148104	5.100652	3.38E-07	4.70E-06
MYH2	22.6968591	5.438814739	1.176551	4.622675	3.79E-06	4.02E-05
PIRT	10.6631302	4.213999006	1.261505	3.340455	0.000836	0.004186
SHISA6	27.5964542	5.642286273	1.167534	4.832651	1.35E-06	1.61E-05
DNAH9	59.3912919	6.276224438	0.981583	6.393979	1.62E-10	4.06E-09
LINC00670	10.8447066	4.116553698	1.270511	3.240076	0.001195	0.005595
ARHGAP44	185.488631	2.624101297	0.46546	5.637654	1.72E-08	3.08E-07
HS3ST3A1	460.777551	-3.049260677	0.414346	-7.35921	1.85E-13	6.90E-12
HS3ST3B1	331.440875	-2.546743433	0.43502	-5.85432	4.79E-09	9.42E-08
AC005224.3	87.1407504	-2.130980776	0.687742	-3.09852	0.001945	0.008364
PMP22	790.38329	2.261431605	0.35298	6.40669	1.49E-10	3.76E-09
TRIM16	724.880015	-2.690415313	0.364216	-7.38687	1.50E-13	5.71E-12
LINC02087	9.78454293	4.008050513	1.275572	3.14216	0.001677	0.007433
TRPV2	203.159428	-2.972878908	0.471517	-6.30492	2.88E-10	6.95E-09
CCDC144A	17.5014763	4.91466879	1.218333	4.03393	5.49E-05	0.000422
TBC1D27P	14.0369091	4.644853865	1.233596	3.765297	0.000166	0.001098
TNFRSF13B	10.6631302	4.213999006	1.261505	3.340455	0.000836	0.004186
RPL13P12	6861.49193	3.413673022	0.430044	7.937966	2.06E-15	9.66E-14
TOM1L2	2556.83816	-2.553958351	0.317861	-8.03483	9.37E-16	4.60E-14
MYO15A	105.681709	3.234249447	0.676399	4.781574	1.74E-06	2.02E-05
FLII	5784.64004	-2.7009271	0.324524	-8.32275	8.59E-17	4.68E-15
AC107983.1	17.8942343	4.006955951	1.10127	3.638487	0.000274	0.001667
TBC1D28	11.1024238	4.303371118	1.255163	3.428536	0.000607	0.003214
TRIM16L	837.618636	-2.81860221	0.362314	-7.77944	7.28E-15	3.14E-13
SNORD3B-1	42.8811768	2.329110073	0.761455	3.058764	0.002223	0.009314
MFAP4	107.334988	-3.398736996	0.609168	-5.57931	2.41E-08	4.18E-07
CCDC144NL	88.1268681	4.940946909	0.696528	7.093677	1.31E-12	4.37E-11
LINC01563	15.4987018	3.566877684	1.147671	3.107926	0.001884	0.008137
NATD1	705.390975	-3.172107629	0.386796	-8.20099	2.38E-16	1.25E-14
MAP2K3	2088.00833	-2.336008794	0.322136	-7.25162	4.12E-13	1.48E-11
AC068418.3	27.6556963	3.927506004	1.040503	3.774621	0.00016	0.001063
AC068418.2	11.1024238	4.303371118	1.255163	3.428536	0.000607	0.003214
KCNJ18	9.69375473	4.046019533	1.27217	3.180407	0.001471	0.006648

LINC02002	9.62932542	4.002668209	1.275446	3.138251	0.0017	0.0075
AC138761.1	15.1234317	4.58060031	1.243877	3.682518	0.000231	0.001442
AC132825.3	17.1910413	4.929529807	1.215601	4.05522	5.01E-05	0.00039
TBC1D3P5	9.95147198	4.159442183	1.263635	3.291648	0.000996	0.004821
NOS2P1	12.4993814	4.297396672	1.260864	3.408295	0.000654	0.003427
NOS2	19.4242513	4.3486907	1.184187	3.672301	0.00024	0.001489
LINC01992	13.0411748	4.581508302	1.236059	3.706546	0.00021	0.001331
TNFAIP1	2082.77615	-3.055944428	0.365283	-8.36596	5.96E-17	3.29E-15
SARM1	786.938432	-2.264884214	0.404642	-5.59726	2.18E-08	3.81E-07
VTN	623.003892	-3.646311917	0.387155	-9.41823	4.59E-21	3.70E-19
SLC13A2	15.6609607	4.279009673	1.161326	3.684591	0.000229	0.001433
FOXN1	15.4075078	4.704549513	1.232708	3.816434	0.000135	0.000921
SEZ6	46.3143873	2.697854201	0.766537	3.519537	0.000432	0.002429
AC138207.3	34.3751293	4.797815761	0.975651	4.917553	8.76E-07	1.10E-05
AC138207.2	151.083296	3.491084293	0.534892	6.526714	6.72E-11	1.79E-09
RHBDL3	31.123852	3.526747523	0.895375	3.93885	8.19E-05	0.000597
TMEM98	40196.9042	2.601852321	0.371958	6.995019	2.65E-12	8.49E-11
ASIC2	308.248301	-4.297092639	0.494546	-8.68896	3.66E-18	2.26E-16
AC024610.2	14.2184855	4.527395237	1.245853	3.633973	0.000279	0.001689
AC123769.1	15.2522903	4.713246257	1.231253	3.828008	0.000129	0.000883
TMEM132E	19.0009336	5.032568275	1.210814	4.156352	3.23E-05	0.000266
UNC45B	26.8062449	3.966515106	1.068334	3.712805	0.000205	0.001303
SLFN13	52.9946607	2.796564528	0.778789	3.590913	0.00033	0.001946
SLFN12L	12.1625875	4.422982886	1.248134	3.543676	0.000395	0.002253
SLFN14	15.0970728	4.718984564	1.230081	3.836321	0.000125	0.000858
RASL10B	1192.14667	-2.357923581	0.33241	-7.09341	1.31E-12	4.37E-11
MMP28						
