# Supplementary material for: Global Transcriptional and Epigenetic Reconfiguration during Chemical Reprogramming of Human Retinal Pigment Epithelial Cells into Photoreceptor-like Cells
Source: Cells. 2022 Oct 6;11(19):3146. doi: 10.3390/cells11193146 (PMC9564162; doi:10.3390/cells11193146)
Supplement: Supplementary file 1 [file cells-11-03146-s001.zip › Supplementary Table S3.pdf]

Supplementary Materials

Supplementary Table S3:

Summary of sequence libraries generated in this study

Sample name	Cell types	Time	Treatment	Library type
RPE_ctrl_H1a	RPE cells	Day 0	None	Bulk RNA-seq
RPE_ctrl_H1b	RPE cells	Day 0	None	Bulk RNA-seq
RPE_D3_5F_H2a	RPE derived reprogramming intermediate	Day 3	5F	Bulk RNA-seq
RPE_D3_5F_H2b	RPE derived reprogramming intermediate	Day 3	5F	Bulk RNA-seq
RPE_D3_5FA_H3a	RPE derived reprogramming intermediate	Day 3	5FA	Bulk RNA-seq
HDF_ctrl_S6	HDF	Day 0	None	Bulk RNA-seq
HDF_ctrl_DR13	HDF	Day 0	None	Bulk RNA-seq
RPE_d10_5FA_x1	RPE derived CiPCs	Day 10	5FA	Bulk RNA-seq
RPE_d10_5FA_x10	RPE derived CiPCs	Day 10	5FA	Bulk RNA-seq
RPE_d10_only_ASO_x2	RPE with ASO treatment	Day 10	ASO only	Bulk RNA-seq
RPE_d10_5F_x3	RPE derived CiPCs	Day 10	5F	Bulk RNA-seq
HDF_d10_5FA_x4	HDF derived CiPCs	Day 10	5FA	Bulk RNA-seq
HDF_d10_5F_x5	HDF derived CiPCs	Day 10	5F	Bulk RNA-seq
HDF_d10_only_ASO_x6	HDF with ASO treatment	Day 10	ASO only	Bulk RNA-seq
RPE_d10_5FA_x7	RPE derived CiPCs	Day 10	5FA	Bulk RNA-seq
RPE_d10_only_ASO_x8	RPE with ASO treatment	Day 10	ASO only	Bulk RNA-seq
RPE_d10_5F_x9	RPE derived CiPCs	Day 10	5F	Bulk RNA-seq
RPE_ctrl_M5	RPE	Day 0	None	EM-seq
HDF 5F d10_em5	HDF derived CiPCs	Day 10	5F	EM-seq
HDF ASO+5F d10_em6	HDF derived CiPCs	Day 10	5FA	EM-seq
RPE 5F d10_em8	RPE derived CiPCs	Day 10	5F	EM-seq
RPE ASO+5F d10_em9	RPE derived CiPCs	Day 10	5FA	EM-seq
RPE_ctrl	RPE	Day 0	None	scRNA-seq
RPE_d3_5F	RPE derived reprogramming intermediate	Day 3	5F	scRNA-seq
RPE_d3_5FA	RPE derived reprogramming intermediate	Day 3	5FA	scRNA-seq
RPE_d10_5FA	RPE derived CiPCs	Day 10	5FA	scRNA-seq
