# Supplementary material for: Global Transcriptional and Epigenetic Reconfiguration during Chemical Reprogramming of Human Retinal Pigment Epithelial Cells into Photoreceptor-like Cells
Source: Cells. 2022 Oct 6;11(19):3146. doi: 10.3390/cells11193146 (PMC9564162; doi:10.3390/cells11193146)
Supplement: Supplementary file 1 [file cells-11-03146-s001.zip › Supplementary Table S4.pdf]

Supplementary Materials

Supplementary Table S4: qPCR primers and antibodies used in this study.

qPCR primers used in this study

Gene	Forward primer	Reverse primer
GAPDH	CATGAGAAGTATGACAACAGCCT	AGTCCTTCCACGATACCAAAGT
RHO	TCTCAACTACATCCTGCTCAACC	GGACCACAGGGCAATTTCAC
RCVRN	GGTAACGGGACCATCAGCAA	TCTTCTCGGCTCGCTTTTCC
CRX	GCCCCACTATTCTGTCAACGC	CTTGAACCAAACCTGAACCCTG
CHX10	AGCTAGAGGAGCTGGAGAAG	CATGATGCCATCCTTGGCTG
OTX2	AGCCCTCACTCGCCACATCT	CACGGAGGGGTGCAGCAAGTC
ASCL1	CGGCCAACAAGAAGATGAGT	TGGAGTAGTTGGGGGAGATG
PTBP1	CTCCGCCATTTTGTGAGTCTA	CGCTTTGTACCAACGGCTAT
GRM4	TGAGGGTGCTGTCACGATCC	ACGTGGCTGCCCTTCTTGAG
GLUD2	CACTCTGCCTTGGCATACAC	CTCAGGTCCAATCCCAGGTT
ACTB	CATCGAGCACGGCATCGTCA	TAGCACAGCCTGGATAGCAAC
BMP4	CTTTACCGGCTTCAGTCTG	GGGATGCTGCTGAGGTTAAA
SMAD3	CATCGAGCCCCAGAGCAATA	GTGGTTCATCTGGTGGTCACT
COL1A1	AGGGCCAAGACATC	AGATCACGTCATCGCACAACA
SMAD7	GAATCTTACGGGAAGATCAACCC	CGCAGAGTCGGCTAAGGTG
TGFB1	CCCTGGACACCAACTATTGC	TGCGGAAGTCAATGTACAGC

Antibodies used in this study

Antibodies	Species	Dilution	Manufacturer	Catalogue Number
TUJ1	Rabbit	1:500	Millipore	MAB1637
MAP2	Rabbit	1:1000	Chemicon	AB5622
RHODOPSIN	Mouse	1:200	Millipore	MAB5316
RHODOPSIN	Rabbit	1:100	Abcam	ab155097
RECOVERIN	Rabbit	1:1000	Millipore	AB5585
RECOVERIN	Rabbit	1:100	Assaypro	12151-05061
CRX	Sheep	1:100	R&D Systems	AF7085
Human Nuclear Antigen (HNA)	Mouse	1:200	Novus Biologicals	NBP2-34342
PTBP1	Rabbit	1:500	ABclonal	A607
OPN1SW	Goat	1:200	Santa Cruz Biotechnology	sc-14363
Beta Actin	Rabbit	1:1000	Abcam	Ab8227
Cy™2 AffiniPure Goat Anti- Rabbit IgG	Goat	1:600	Jackson ImmunoResearch Labs	111-225-144
Cy™2 AffiniPure Goat Anti-Mouse IgG	Goat	1:600	Jackson ImmunoResearch Labs	115-225-146
Cy™3 AffiniPure Donkey Anti-Rabbit IgG	Donkey	1:600	Jackson ImmunoResearch Labs	711-165-152
Cy™3 AffiniPure Donkey Anti-Mouse IgG	Donkey	1:600	Jackson ImmunoResearch Labs	715-165-151
Cy™3 AffiniPure Donkey Anti-Sheep IgG	Donkey	1:600	Jackson ImmunoResearch Labs	713-165-147
Cy™3 AffiniPure Donkey Anti-Goat IgG	Donkey	1:600	Jackson ImmunoResearch Labs	705-165-003
HRP Goat Anti-Rabbit IgG	Goat	1:10000	Jackson ImmunoResearch Labs	111-035-003
HRP Goat Anti-Mouse IgG	Goat	1:10000	Jackson ImmunoResearch Labs	115-035-003
