# Supplementary material for: Global Transcriptional and Epigenetic Reconfiguration during Chemical Reprogramming of Human Retinal Pigment Epithelial Cells into Photoreceptor-like Cells
Source: Cells. 2022 Oct 6;11(19):3146. doi: 10.3390/cells11193146 (PMC9564162; doi:10.3390/cells11193146)
Supplement: Supplementary file 1 [file cells-11-03146-s001.zip › Supplementary Table S5.pdf]

Supplementary Materials

Supplementary Table S5:

Different mediums and reagents used in this study

Cell type	Basic medium components	Additional molecules/components	Reason	Reference
RPE	Growth medium:<br>α-MEM<br>2 mmol/L L-glutamine<br>1X N1 supplement<br>1X non-essential amino acids<br>0.25 mg/mL taurine<br>0.013 μg/L Triiodo-thyronine<br>20 μg/L Hydrocortisone<br>1X penicillin-streptomycin<br>5% fetal bovine serum			[1]
CiPCs (D1-D10)	Photoreceptor induction medium:<br>DMEM/F12<br>10% knockout serum replacement<br>2% B27<br>5 ng/mL Noggin<br>5 ng/mL IGF-1	0.5 mM VPA<br>4.8 μM CHIR99021<br>2 μM Repsox<br>10 μM Forskolin<br>10 μM IWR1<br>3 nM Sonic hedgehog<br>100 μM Taurine<br>1 μM Retinoic acid	1.VPA: facilitates photoreceptor differentiation; promotes DNA demethylation; epithelial-mesenchymal transition (EMT) inhibition; regulation of glutamate signaling pathway	VPA[2-5]
			2.CHIR99021: promotes neuronal reprogramming	CHIR99021[6, 7]
			3.Repsox: promotes neuronal reprogramming; assisting mesenchymal-epithelial transition (MET)	Repsox [8, 9]
			4.Forskolin: promotes neuronal reprogramming	Forskolin [10, 11]
			5.IWR1: promotes photoreceptor differentiation	IWR1[12]
			6.Sonic hedgehog: facilitates rod photoreceptor differentiation	Sonic hedgehog [13]
			7.Taurine: promotes retinal cell differentiation	Taurine [14]
			8.Retinoic acid: promotes photoreceptor differentiation	Retinoic acid [15]

CiPCs (D11~)	Photoreceptor differentiation medium:<br>DMEM/F12<br>2% B27<br>1% N2<br>10 ng/mL Noggin<br>10 ng/mL IGF1<br>5 ng/mL bFGF	0.5 mM VPA<br>4.8 μM CHIR99021<br>2 μM Repsox<br>10 μM Forskolin<br>10 μM IWR1	9.bFGF: promotes retinal regeneration from RPE	bFGF [16, 17]
HDF	Growth medium:<br>DMEM with glutamine<br>1X non-essential amino acids<br>1X penicillin-streptomycin<br>10% fetal bovine serum			[12]

Reference

1. Maminishkis, A., S. Chen, S. Jalickee, T. Banzon, G. Shi, F. E. Wang, T. Ehalt, J. A. Hammer, and S. S. Miller. "Confluent Monolayers of Cultured Human Fetal Retinal Pigment Epithelium Exhibit Morphology and Physiology of Native Tissue." Invest Ophthalmol Vis Sci 47, no. 8 (2006): 3612-24.
2. Mello, M. L. S. "Sodium Valproate-Induced Chromatin Remodeling." Front Cell Dev Biol 9 (2021): 645518.
3. Chen, L., A. Alam, A. Pac-Soo, Q. Chen, Y. Shang, H. Zhao, S. Yao, and D. Ma. "Pretreatment with Valproic Acid Alleviates Pulmonary Fibrosis through Epithelial-Mesenchymal Transition Inhibition in Vitro and in Vivo." Lab Invest 101, no. 9 (2021): 1166-75.
4. Osakada, F., S. Ooto, T. Akagi, M. Mandai, A. Akaike, and M. Takahashi. "Wnt Signaling Promotes Regeneration in the Retina of Adult Mammals." J Neurosci 27, no. 15 (2007): 4210-9.
5. Park, H. J., W. S. Kang, J. W. Paik, and J. W. Kim. "Effect of Valproic Acid through Regulation of Nmda Receptor-Erk Signaling in Sleep Deprivation Rats." J Mol Neurosci 47, no. 3 (2012): 554-8.
6. Yang, Y., R. Chen, X. Wu, Y. Zhao, Y. Fan, Z. Xiao, J. Han, L. Sun, X. Wang, and J. Dai. "Rapid and Efficient Conversion of Human Fibroblasts into Functional Neurons by Small Molecules." Stem Cell Reports 13, no. 5 (2019): 862-76.
7. Ladewig, J., J. Mertens, J. Kesavan, J. Doerr, D. Poppe, F. Glaue, S. Herms, P. Wernet, G. Kogler, F. J. Muller, P. Koch, and O. Brustle. "Small Molecules Enable Highly Efficient Neuronal Conversion of Human Fibroblasts." Nat Methods 9, no. 6 (2012): 575-8.
8. Hu, W., B. Qiu, W. Guan, Q. Wang, M. Wang, W. Li, L. Gao, L. Shen, Y. Huang, G. Xie, H. Zhao, Y. Jin, B. Tang, Y. Yu, J. Zhao, and G. Pei. "Direct Conversion of Normal and Alzheimer's Disease Human Fibroblasts into Neuronal Cells by Small Molecules." Cell Stem Cell 17, no. 2 (2015): 204-12.
9. Liu, X., H. Sun, J. Qi, L. Wang, S. He, J. Liu, C. Feng, C. Chen, W. Li, Y. Guo, D. Qin, G. Pan, J. Chen, D. Pei, and H. Zheng. "Sequential Introduction of Reprogramming Factors Reveals a Time-Sensitive Requirement for Individual Factors and a Sequential Emt-Met Mechanism for Optimal Reprogramming." Nat Cell Biol 15, no. 7 (2013): 829-38.
10. Li, X., X. Zuo, J. Jing, Y. Ma, J. Wang, D. Liu, J. Zhu, X. Du, L. Xiong, Y. Du, J. Xu, X. Xiao, J. Wang, Z. Chai, Y. Zhao, and H. Deng. "Small-Molecule-Driven Direct Reprogramming of Mouse Fibroblasts into Functional Neurons." Cell Stem Cell 17, no. 2 (2015): 195-203.
11. Liu, M. L., T. Zang, Y. Zou, J. C. Chang, J. R. Gibson, K. M. Huber, and C. L. Zhang. "Small Molecules Enable Neurogenin 2 to Efficiently Convert Human Fibroblasts into Cholinergic Neurons." Nat Commun 4 (2013): 2183.
12. Mahato, B., K. D. Kaya, Y. Fan, N. Sumien, R. A. Shetty, W. Zhang, D. Davis, T. Mock, S. Batabyal, A. Ni, S. Mohanty, Z. Han, R. Farjo, M. J. Forster, A. Swaroop, and S. H. Chavala. "Pharmacologic Fibroblast Reprogramming into Photoreceptors Restores Vision." Nature 581, no. 7806 (2020): 83-88.
13. Levine, E. M., H. Roelink, J. Turner, and T. A. Reh. "Sonic Hedgehog Promotes Rod Photoreceptor Differentiation in Mammalian Retinal Cells in Vitro." J Neurosci 17, no. 16 (1997): 6277-88.
14. Altshuler, D., J. J. Lo Turco, J. Rush, and C. Cepko. "Taurine Promotes the Differentiation of a Vertebrate Retinal Cell Type in Vitro." Development 119, no. 4 (1993): 1317-28.

15. Kelley, M. W., J. K. Turner, and T. A. Reh. "Retinoic Acid Promotes Differentiation of Photoreceptors in Vitro." Development 120, no. 8 (1994): 2091-102.
16. Pittack, C., M. Jones, and T. A. Reh. "Basic Fibroblast Growth Factor Induces Retinal Pigment Epithelium to Generate Neural Retina in Vitro." Development 113, no. 2 (1991): 577-88.
17. Luz-Madrigal, A., E. Grajales-Esquivel, A. McCorkle, A. M. DiLorenzo, K. Barbosa-Sabanero, P. A. Tsonis, and K. Del Rio-Tsonis. "Reprogramming of the Chick Retinal Pigmented Epithelium after Retinal Injury." BMC Biol 12 (2014): 28.
